# Substituent effects in *N*-acetylated phenylazopyrazole photoswitches

**DOI:** 10.3762/bjoc.21.66

**Published:** 2025-04-25

**Authors:** Radek Tovtik, Dennis Marzin, Pia Weigel, Stefano Crespi, Nadja A Simeth

**Affiliations:** 1 Institute of Organic and Biomolecular Chemistry, Georg-August-University, Tammannstraße 2, 37077 Goettingen, Germany; 2 Department of Chemistry, Ångström laboratory, Uppsala University, Box 523, 751 20 Uppsala, Sweden; 3 Cluster of Excellence “Multiscale Bioimaging: from Molecular Machines to Networks of Excitable Cells” (MBExC), University of Göttingen, 37075 Göttingen, Germany

**Keywords:** azobenzenes, azopyrazoles, photochromism, photoswitches, substituent effects

## Abstract

Phenylazopyrazole photoswitches proved to be valuable structural motifs for various applications ranging from materials science to medicine. Despite their potential, their structural diversity is still limited and a larger pool of substitution patterns remains to be systematically investigated. This is paramount as electronic effects play a crucial role in the behavior of photoswitches and a deeper understanding enables their straightforward development for specific applications. In this work, we synthesized novel *N*-acylpyrazole-based photoswitches and conducted a comparative study with 33 phenylazopyrazoles, comparing their photoswitching properties and the impact of electronic effects. Using UV–vis and NMR spectroscopy, we discovered that simple acylation of the pyrazole moiety leads to increased quantum yields of isomerization, long *Z*-isomer life-times, good spectral separation, and high photostability.

## Introduction

Organic photoswitches are molecules reversibly changing their optical and chemical properties upon irradiation. These features offer easy, precise, and reversible control over the system they are embedded in and make them attractive modulators for diverse applications. In the last decades, many classes of photoswitches have been described and extensively studied [1–2]. Among these, azobenzenes belong to the most common ones [3]. They were firstly explored almost 200 years ago [4] and initially used mainly as dyes or pigments [5]. Relatively recently, azobenzenes started to become an important part of state-of-the-art technologies ranging from energy-storage materials [6–7] to pharmacology [8–11], materials chemistry [12–13], control of peptides structure [14–15] or proteins [16], as antibacterial agents [17–18], smart coating [19], or multivalent photoresponsive systems [20–21], to name only a few examples.

Azobenzene and its derivatives show two characteristic absorption bands, namely a π→π* transition around 330 nm and an n→π* one around 450 nm, respectively [22]. The molecule can populate the thermodynamically metastable *Z* isomer by addressing these transitions in the thermally stable *E* form. The relative position of the absorption bands in the azobenzene derivatives depends on the substitution pattern on the aromatic rings, which can act as a handle to affect the absorption properties of the compound class [3]. For instance, push–pull systems or the introduction of tetra-*ortho* substituents were reported to either bathochromically shift the UV–vis absorption spectrum or lead to a better separation of the n→π* bands of the two photoisomers and allow for visible-light-responsive switches [23–26].

In recent years, heteroaryl azobenzene derivatives have revealed superior properties to classical azobenzenes. Heterocyclic rings offer, for example, enhanced polarity, electron pairs for metal coordination [27], better water-solubility, and variable p*K*_a_ [28–29]. Special attention has been given to 5-membered *N*-heterocyclic azobenzenes, which not only maintain the azobenzenes properties but often show higher quantum yields and increased thermal half-life of the metastable state. For the half-life, the choice of the heterocycle is crucial, as revealed through density functional theory (DFT) calculations, which showed that a 5-membered ring promotes the stability of the *Z* isomer [30]. Within these, azo-photoswitches based on a 1,3,5-trimethylpyrazole ring (phenylazopyrazole; **PAP**) became particularly popular, showing almost quantitative back and forward photoswitching and high thermal stability [31]. Moreover, 1*H*-pyrazole derivatives [32] and arylazopyrazolium [33] compounds were investigated in detail and allowed to correlate thermal relaxation rates and steric or electronic effects as well as mechanistic peculiarities, which has been in the spotlight recently for different classes of azobenzenes [34].

Despite these studies, the variety of substitution patterns in **PAP**s is limited compared to classical azobenzenes and remains to be better understood. Interestingly, Leistner et al*.* recently reported that introducing a formyl group in the *para* position of tetra-*ortho*-fluoro or -chloro-substituted azobenzenes leads to the decrease of the HOMO–LUMO gap resulting in a significant 50 nm bathochromic shift of the n*→*π* absorption band further moving their UV–vis absorption spectrum towards the therapeutic window [35]. Moreover, a similar modification in stilbene-based photoswitches and molecular motors showed an increase in performance and photoisomerization quantum yield [36–37].

Consequently, we were interested to study the photoswitching properties of eleven novel *N*-acetylated analogous **NAc**-**PAP**s and the influence of substituent effects. Furthermore, we compared their photochromism with a set of 22 known *N*-methylated (**NMe**-) and unfunctionalized (**NH**-) **PAP**s in moderate to good yields (66–85%).

## Results and Discussion

### Synthesis

**Scheme 1:**
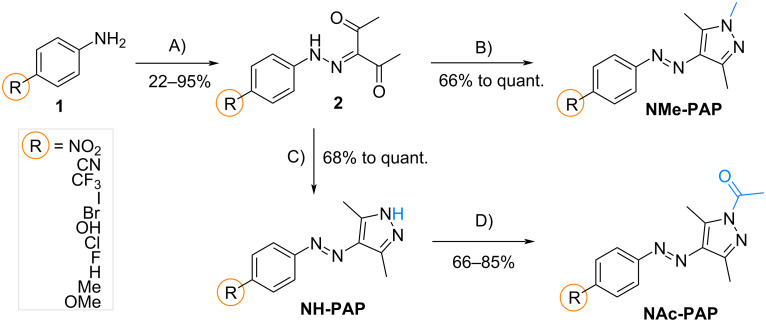
Reaction pathway for synthesizing *N*H-substituted, methylated-, and acetylated arylazopyrazoles. Conditions: A) NaNO_2_, AcOH + HCl at 0 °C, then, 2,4-pentanedione, NaOAc in EtOH + H_2_O, reflux; B) MeNHNH_2_, EtOH, reflux; C) NH_2_NH_2_, EtOH, reflux; D) AcCl, NaOAc in DCM, 0 °C to rt.

The **PAP**s in this study were obtained in a straightforward, three-step metal-free synthesis from commercially available aniline derivatives adapting known procedures [31,38]. An overview of the compounds used in this study and their synthesis is displayed in Scheme 1. First, a diazotization of a given aniline **1** and reaction with 2,4-pentanedione gave intermediate **2**, with yields which strongly depended on the residue in the *para*-position. Specifically, the residues bearing an electron-donating group (EDG) such as -OMe or -OH showed low yields because of the poor reactivity of the diazonium salt. When strong electron-withdrawing groups (EWGs) were introduced, the yield was also reduced, likely due to the low nucleophilicity of the aniline derivative and the ineffective formation of a diazonium salt. An annulation reaction of compound **2** was performed with either hydrazine or methylhydrazine to yield **NH-PAP**s or **NMe-PAP**s, respectively, in high to quantitative yields. The **NAc-PAP**s were synthesized via an acetylation reaction of **NH-PAP**s with acyl chloride, forming the novel **NAc-PAP**s. While the molecules could be extracted and purified by column chromatography without further issue, we observed some instability at acidic pH during analysis. Thus, we tested the stability of **NAc-PAP-H** (1 mM, MeOH, ambient conditions, 2 h) at pH 2, 12, and in the presence of DBU (10^−2^ M) and found that the acetyl group was lost (cf. Supporting Information File 1, section 2.4).

### UV–vis absorption spectroscopy

With a set of photoswitches at hand, we studied the obtained **PAP**s through UV–vis absorption spectroscopy. The photochemical properties are summarized in Table 1. All compounds show absorption maxima in the range of 324–368 nm in CH_3_CN, which corresponds to the strongly allowed π→π* transition, while the weaker n→π* band shows absorption maxima in the range of 410–451 nm in CH_3_CN.

**Table 1:** Photophysical properties of synthesized **PAP** derivatives in CH_3_CN.

R:	**NH-PAP**			**NMe-PAP**			**NAc-PAP**		
	λ_max_ [nm]		ε (π→π*) 10^3^ [L/(mol·cm)]	λ_max_ [nm]		ε (π→π*) 10^3^ [L/(mol·cm)]	λ_max_ [nm]		ε (π→π*) 10^3^ [L/(mol·cm)]
	π→π*	n→π*		π→π*	n→π*		π→π*	n→π*	

OH	342	415*	16.8 ± 0.5	345	^a^	18.0 ± 0.3	344	443	13.0 ± 0.1
OMe	344	410	18.9 ± 0.8	345	^a^	19.4 ± 1.1	344	447	28.3 ± 0.4
Me	336	413*	19.1 ± 0.9	338	434	21.8 ± 1.3	333	425	24.1 ± 0.4
H	330	440	18.9 ± 0.1	337	433	19.5 ± 1.1	327	427	24.5 ± 0.4
F	332	437	17.2 ± 0.7	335	435	17.3 ± 0.5	327	426	20.2 ± 0.1
Cl	336	441	20.9 ± 0.6	341	447	22.1 ± 1.1	333	414	26.9 ± 0.3
Br	341	450	17.8 ± 0.5	343	451	27.1 ± 0.1	334	429	25.2 ± 0.2
I	343	451	24.2 ± 0.1	347	445	22.1 ± 1.3	340	422	29.1 ± 0.6
CF_3_	335	442	16.3 ± 0.1	341	444	23.3 ± 0.1	324	435	23.5 ± 0.5
CN	345	448	24.1 ± 1.4	352	451	13.8 ± 0.2	333	444	27.3 ± 0.5
NO_2_	361	449	21.3 ± 1.0	368	^a^	13.8 ± 0.1	345	448	26.4 ± 0.5

^a^Transition band appears as shoulder.

**Figure 1:**
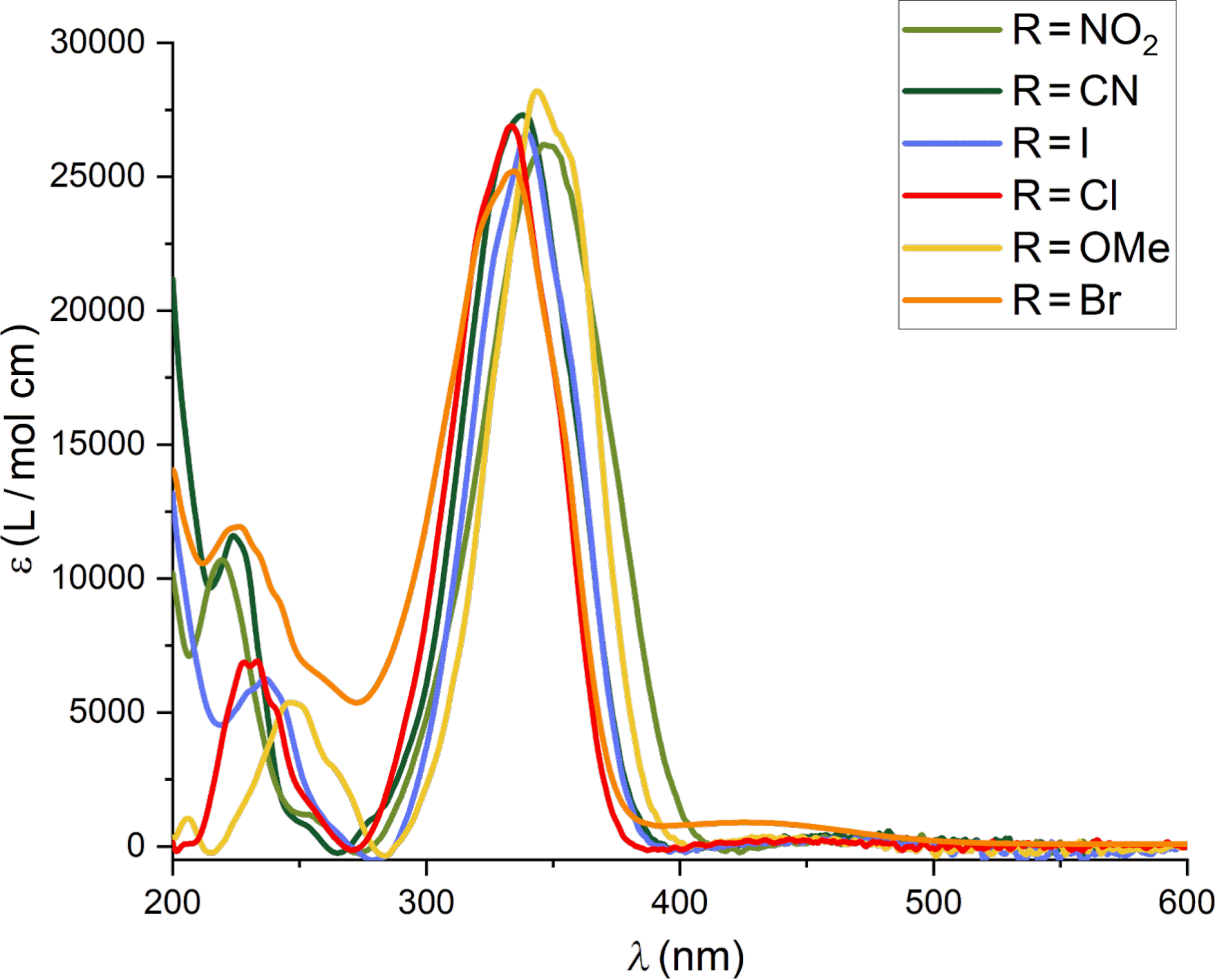
UV–vis absorption spectra of selected **NAc-PAP** derivatives in CH_3_CN. The strong π→π* can be observed in the region of 330–360 nm.

The introduction of methyl and acyl groups on one nitrogen of the pyrazole ring led to a hyperchromic effect and a minor bathochromic shift. Also, the introduction of an EDG or EWG on the R position led to minor hyperchromic effects and a bathochromic shift (exemplary the UV–vis absorption spectra of **NAc-PAP**s are displayed in Figure 1 and a full set is available in Supporting Information File 1, section 3.5). In the case of EWGs, we observed positive hyperchromic effects while for EDGs negative hyperchromic effects compared for **NH-** and **NMe-PAP-H**.

### Photochemical isomerization

**Figure 2:**
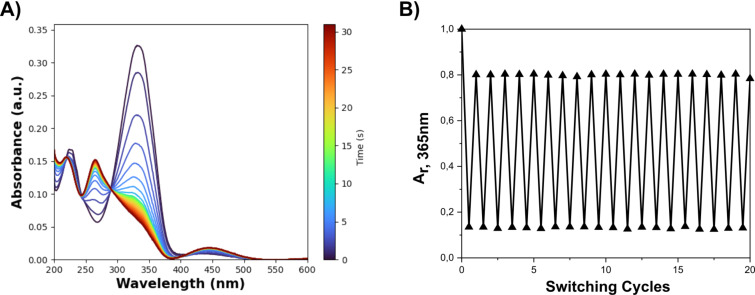
A) Time-resolved UV–vis absorption spectra of **NAc-PAP-CN** upon 365 nm irradiation (12.5 µM in CH_3_CN, at 25 °C). B) Absorbance of the same sample at 365 nm (A_r, 365nm_) after reaching PSS^365^ or PSS^455^, respectively, to show the recyclability.

Upon irradiation with a 365 nm LED (exemplarily displayed for **NMe**-**PAP**-**CN** in Figure 2), we observed a decrease of the π→π* band for the *E* isomers. Simultaneously the π→π* bands around 253–369 nm and the n→π* bands around 426–457, respectively, of the *Z* isomers increased until reaching the photostationary state (PSS).

The UV–vis absorption spectra of the *Z* isomers show a more intense n→π* transition and less intense π→π* absorption band compared to the *E* isomers, because of the loss of the molecule’s planarity [30,39]. This change in color can also be seen by naked eye with the solution changing from pale to dark yellow (cf. Supporting Information File 1, Figure S2). In Table 2, the absorption maxima of *Z*–**PAP** derivatives in CH_3_CN are summarized. For EWGs, such as CF_3_, CN, or NO_2_, the absorption maxima are redshifted, while for EDGs, such as OH or OMe, they are shifted towards the blue. Especially for **NAc-PAP**s, we observe a higher spectral separation of the π→π* and the n→π* transitions of the *Z* isomers compared to **NMe-PAP**s and **NH-PAP**s. For instance, the separation for the π→π* transition band and the n→π* is 176 nm for **NAc-PAP-CN**, while for **NMe-PAP-CN** 146 nm and for **NH-PAP-CN** 102 nm are found.

**Table 2:** UV–vis absorption maxima of *Z*-**PAP** derivatives in CH_3_CN (obtained through 365 nm irradiation to the corresponding PSS^365^).

R	**NH-PAP**		**NMe-PAP**		**NAc-PAP**	
	λ_max_ [nm]		λ_max_ [nm]		λ_max_ [nm]	
	π→π*	n→π*	π→π*	n→π*	π→π*	n→π*

OH	304	436	307	435	309	445
OMe	307	426	307	411	307	445
Me	331	457	301	456	279	439
H	300	433	358	455	280	442
F	334	441	299	445	280	442
Cl	358	454	302	456	277	437
Br	366	452	302	457	275	435
I	366	455	366	456	279	445
CF_3_	369	453	304	442	253	440
CN	361	463	311	457	265	441
NO_2_	350	463	372	448	280	448

Subsequent illumination with a 445 nm LED led to the formation of the *E* isomer, which is accompanied by an increase of its π→π* band until reaching the PSS^445^. However, judging from the intensity of the *E* isomers’ π→π* band, the photostationary distribution (PSD) of the initial dark state could not be fully regenerated by photochemical means for all 33 **PAP**s (vide infra, ex-situ NMR measurements and thermal half-lifes).

The recyclability of **NH-PAP**s was previously studied by Rustler et al. by altered irradiation with 365 and 420 or 455 nm light, showing a great photostability of these compounds [32]. We performed the same experiment on our **NAc-PAP**s (see Table 3 and section 3.2 in Supporting Information File 1) and did not observe any fatigue after 10–20 cycles of photoswitching, showing high photostability also of **NAc-PAP**s.

**Table 3:** PSS distribution of various **NAc-PAP** derivatives.^a^

R:	365 nm *E:Z* UV–vis	365 nm *E:Z* NMR	445 nm *E:Z* UV–vis	445 nm *E:Z* NMR

OH	8:92	^a^	81:19	^a^
OMe	10:90	5:95	76:24	78:22
Me	4:96	4:96	77:23	77:23
H	22:78	22:78	81:19	81:19
F	8:92	8:92	79:21	79:21
Cl	16:84	4:96	73:27	75:25
Br	2:98	4:96	77:23	75:25
I	6:94	6:94	75:25	75:25
CF_3_	17:83	17:83	78:22	78:22
CN	15:85	5:95	78:22	78:22
NO_2_	14:86	27:73	84:16	88:12

^a^Not determined; relaxation towards the *Z* isomer is too fast.

To quantify the extent of photoisomerization, the isomer distributions of **NAc-PAP**s at the PSS^365^ and PSS^445^ were investigated using UV–vis and NMR spectroscopy. For the ex-situ ^1^H NMR irradiation experiments, we irradiated our samples in a cuvette until the PSS was reached and measured immediately afterwards the ^1^H NMR spectrum. We compared these results to simply estimating the isomer distribution at the PSS (cf. Supporting Information File 1, section 3.1) from the UV–vis spectra and observed good agreement, meaning the results from UV–vis spectroscopy can be used for rough PSD estimation, due to good spectral separation of the isomers. Since we encountered fast back isomerization only for **NAc-PAP-OH** (vide infra), the PSDs could only be determined from the UV–vis spectra.

**NAc-PAP**s showed high to quantitative formation of the *Z*-configurated isomers. For example, **NAc-PAP-Br** showed an isomerization distribution of 98% *Z* by UV–vis and 96% *Z* by ^1^H NMR. EWGs, such as **NAc-PAP-CF_3_**, on the other hand, showed a lower PSD for the *Z* isomer of 83% by both UV–vis and ^1^H NMR spectroscopy. For **NMe-** and **NAc-PAP**s, we observed increased *Z* content to almost quantitative photoisomerization after 365 nm illumination for most cases compared to **NH-PAP**s. For **NMe-PAP**s, West et al. attributed this to a pronounced spectral separation between the π→π* bands of *E* and *Z*-isomers, which we also found for **NAc-PAP**s [31].

For the back isomerization with 445 nm light, favoring the *E* isomer, we observed that the amount of *E* isomer present in the dark state could not be reached for any of the **NAc-PAP**s. For example, for **NAc-PAP-Cl** 73% of the *E* isomer could be reformed and for **NAc-PAP-NO_2_** 84%. Since we observed for **NAc-PAP**s a decrease of spectral separation of the n→π* bands for the *E* and *Z* isomers, a non-quantitative PSD upon irradiation with 445 nm can be explained by the presence of a competing *E→Z* isomerization at the same wavelength. In general, **NAc-PAP**s show only a minor substitution effect on the PSD upon irradiation with 365 or 445 nm LED light.

Next, we studied the photoisomerization quantum yields (QYs) Φ_E→Z_ and Φ_Z→E_ and the impact of substitution effects on the *para* position, using two types of LEDs (365 nm and 445 nm, details see Supporting Information File 1, section 3.5). The values determined are provided in Table 4.

**Table 4:** Quantum yield values for the *E***→***Z* and *Z***→***E* photoisomerization of **PAP** derivatives with the corresponding 365 nm and 445 nm LED in CH_3_CN.^a^

R:	X = H	X = Me	X = Ac	X = Ac
	π→π* [%]			n→π* [%]

OH	33	44	56	^a^
OMe	29	38	65	28
Me	18	45	44	31
H	11	22	14	30
F	29	56	72	61
Cl	26	36	59	42
Br	21	32	55	39
I	29	40	45	32
CF_3_	25	27	69	30
CN	25	26	26	10
NO_2_	21	18	16	20

^a^Not determined.

For Φ_E→Z_, we could observe for nearly all **NAc-PAP** derivatives, higher values compared to **NMe-PAP**s, followed by **NH-PAP**s in a descending order. For example, for **NH-PAP-CF_3_** we found a QY of 25%, which went towards 27% for the **NMe-PAP-CF_3_** and finally to 69% for the **NAc-PAP-CF_3_**. Especially for EDGs and weak EWGs, the **NAc-PAP**s showed higher Φ_E→Z_ compared to **NMe-PAP**s and **NH-PAP**s, while strong EWGs show a higher to equal Φ_E→Z_. Interestingly, for R = Me and H, we recorded higher QYs in **NMe-PAP**s than in **NAc-PAP**s (22% and 45% for R = H and R = Me in **NMe-PAP** vs 14% and 44% for the acetylated ones).

In contrast to the excitation of the π→π* state in **NAc-PAP**s, the isomerization process varies in efficiency when the n→π* transition is selectively addressed, with similar or markedly reduced quantum yields. A deviation from Kasha's rule with the opposite outcome (viz. the quantum yield of isomerization proceeding from the excitation of the π→π* state is lower than the one from the n→π*) is reported in various studies on azobenzene [32,40]. Another interesting aspect, that could point to a more complex picture in the excited state landscape of these switches, is that the QYs of **NAc-PAP**s with R = NO_2_ and R = H are lower for the π→π* than for the n→π* transition, while for the other substituents the opposite was found. We thus suspect that the substituents play a crucial role in in the population of the respective exited state and we can at this state not rule out a contribution also from the triplet state.

Moreover, we could not find a quantitative correlation between the R-substituents and Φ_E→Z_, however, some trends can be observed; EWG and EDG lead to higher Φ_E→Z_ for the π→π* transition, while the opposite can be seen for the n→π* transition. We also observed increased numbers for the Φ_E→Z_ of π→π* for **NMe-PAP**s compared to **NH-PAP**s. However, the highest values were observed for our newly synthesized **NAc-PAP**s. For example, for **NH-PAP-CF_3_** we observed that the Φ_E→Z_ has a value of 25%, for **NMe-PAP-CF_3_** 27%, while for **NAc-PAP-CF_3_** we observed an increase of Φ_E→Z_ to 69%.

### Thermal half-lifes

The metastable *Z* isomers can be converted back to the thermodynamically favored *E* form by thermal means. Four mechanisms were predicted using quantum chemistry to describe the *Z*→*E* isomerization in azobenzenes, namely: rotation, inversion, inversion-assisted rotation, and concerted inversion depending on the structure of the azobenzene [40–44]. For **PAP**s, Calbo et al. showed by DFT calculations that the inversion mechanism is one of the fastest relaxation mechanism for heterocyclic azobenzenes (typically for most of the azo dyes [45]). However, the nature of the mechanism is still a matter of current debate, and additional factors, such as the presence of tautomerizable groups [46–47], and the involvement of the triplet state [48], appear to play a role.

Specifically, the rotation mechanism does not explain the low activation entropy observed in azobenzene systems [49] sparking new discussions on the possibility of alternative isomerization pathways [34]. Recently, Reimann et al. computationally showed that the involvement of a triplet state mechanism, which crosses the transition state for the *Z→E* relaxation, could explain the low values of the activation entropy. The same authors also showed experimental evidence for this proposal by an external heavy atom effect on *Z→E* isomerization.

To understand the thermal *Z→E* isomerization in our newly synthesized **NAc-PAP**s, we recorded the process by time-resolved UV–vis absorption spectroscopy and calculated the thermal half-lifes of back isomerization (Table 5 and Supporting Information File 1, section 3.6). EDGs and weakly EWGs, such as halogen substituents, exhibited thermal half-lifes for thermal back isomerization in the range of days. For instance, **NAc-PAP-Cl** or -**Br** converts back to the form with half-lifes of roughly 1.5 days. In contrast, **NAc-PAP-H** or **-Me** demonstrated significantly longer thermal half-lifes, ranging from 9 days to 21.5 days. Notably, **NAc-PAP-OMe**, with a thermal half-life of 4.38 days, extends the thermal half-life significantly compared to the 19.7 minutes observed for **NH-PAP-OMe**.

**Table 5:** Overview over thermal half-lifes for **NAc-PAPs** in CH_3_CN at 30 °C.

R:	τ_1/2_ [d]

OMe	4.38
Me	8.97
H	21.5
F	13.3
Cl	1.51
Br	1.57
I	3.26

R	τ_1/2_ [s]

OH	19.0
CF_3_	1992
CN	696
NO_2_	608

**Figure 3:**
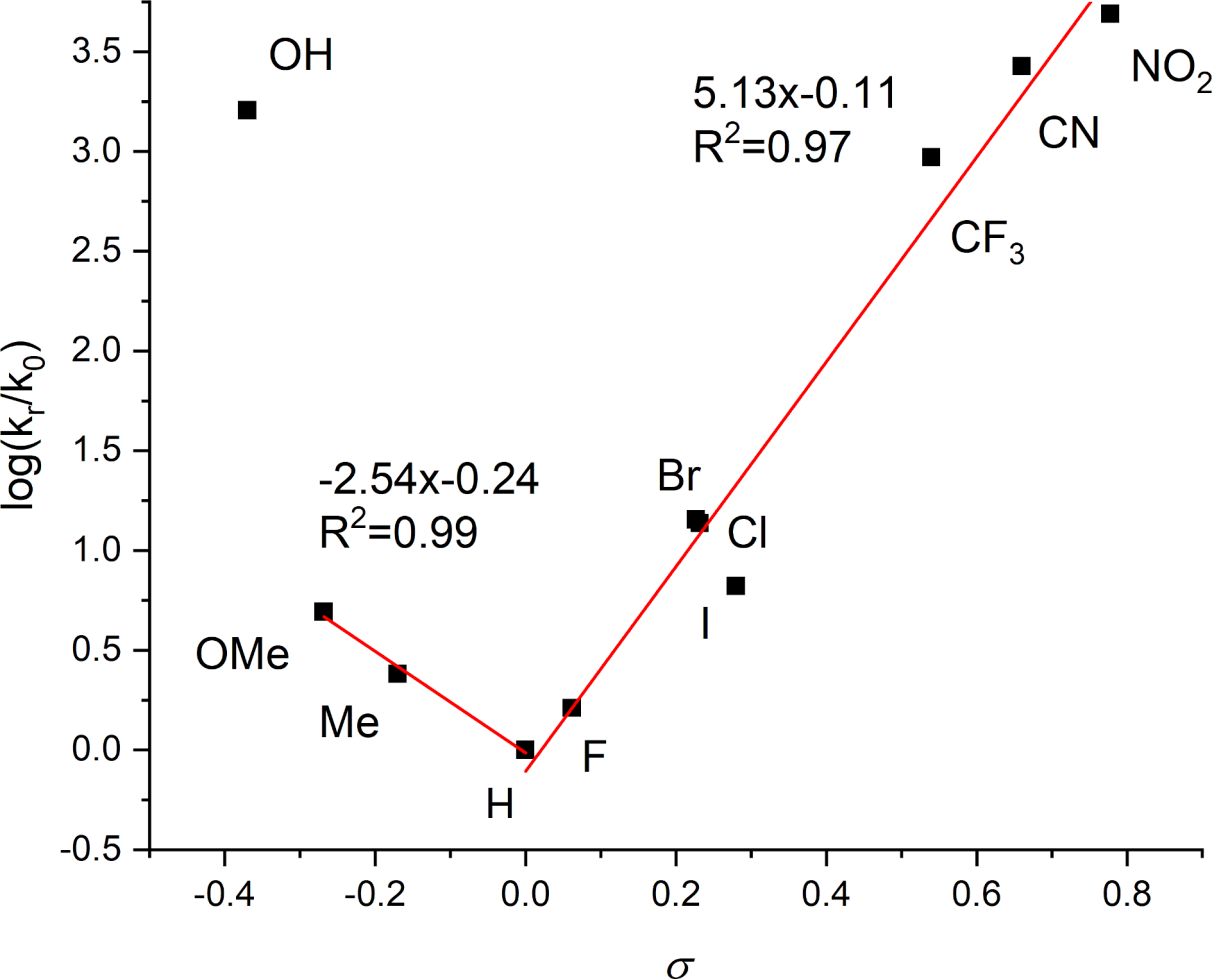
Hammett plot of **NAc-PAP** derivatives.

We subsequently analyzed the electronic effects on the thermal relaxation rates in our **NAc**-**PAP**s using Hammett parameters for the substituents in the *para*-position and found a trending behavior (cf. Figure 3). Specifically, the Hammett plot shows linear trends for both EWGs and EDGs, with a minimum for electron-neutral R = H and R = OH as an outlier, likely due to a contribution of tautomerization [50]. Both linear fits show high slope values, indicating a great dependency on the nature of the substituent. The observed trend behavior indicates an apparent change of mechanism for thermal relaxation to the *E* isomer. This was previously observed for **NH-PAP**s [32] **N-PEG-PAP**s [20], and for azopyrazolium salts [33].

**Figure 4:**
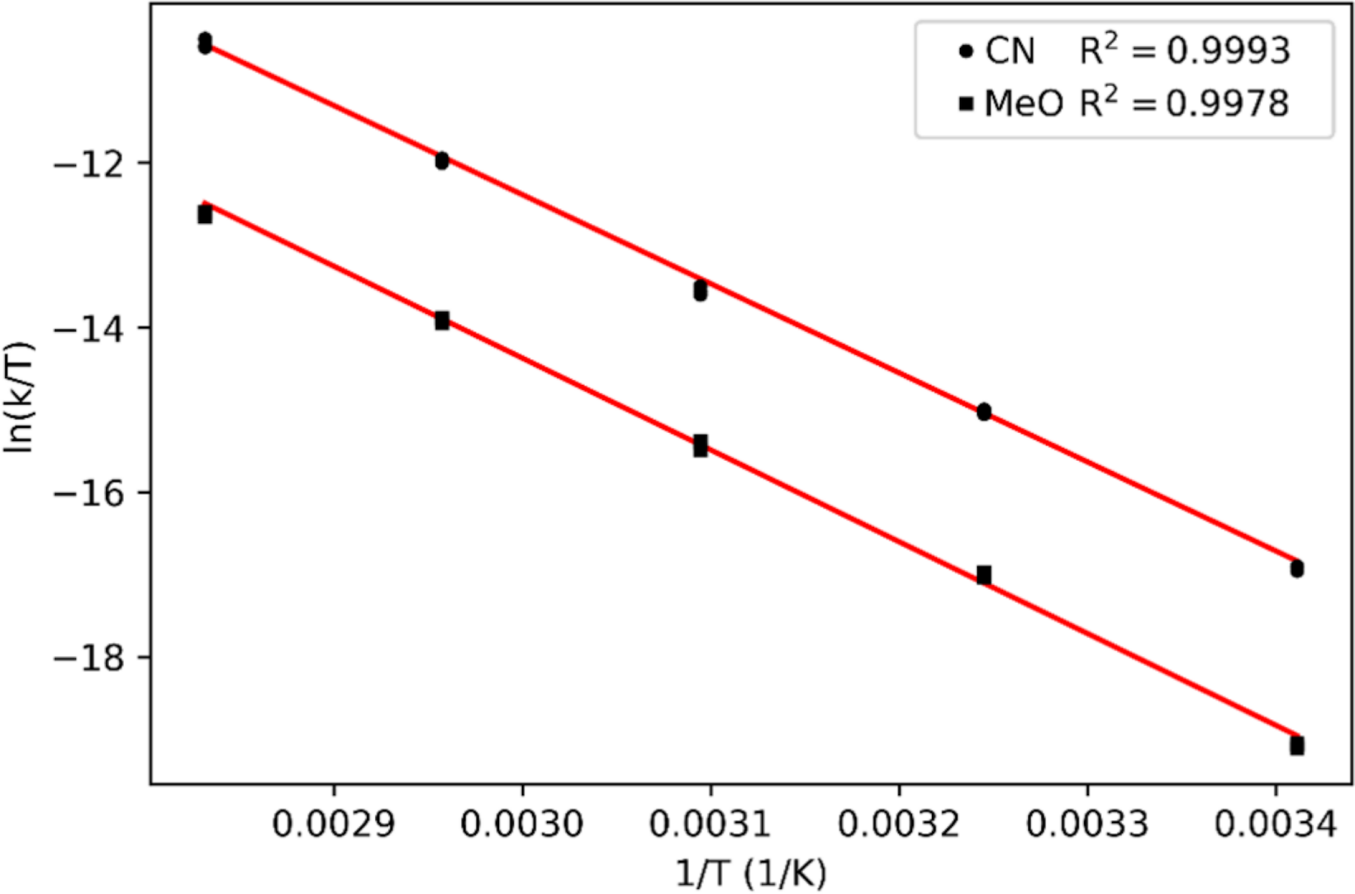
Eyring plots for **NAc-PAP-CN** and **NAc-PAP-OMe**.

To obtain a deeper understanding of the transition states thermodynamic properties, we decided to perform an Eyring analysis of two representative **PAP**s. We chose **NAc-PAP-CN** and **NAc-PAP-OMe**, which lie on two different ends of the Hammett plot and hence should reveal the difference in relaxation mechanism. We measured the temperature-dependency of their relaxation rates in toluene to access higher temperatures (Table 6 and Supporting Information File 1, section 3.7). The Eyring plot for **NAc-PAP-CN** and **NAc-PAP-OMe** are depicted in Figure 4.

**Table 6:** Eyring analysis of **NAc-PAP-CN** and **NAc-PAP-OMe** determined in toluene (for details see Supporting Information File 1, section 3.7).

	**NAc-PAP-CN**	**NAc-PAP-OMe**

Δ*G*^‡^ / kJ/mol	99.1 ± 0.07^a^	104.3 ± 0.1^a^
Δ*H*^‡^ / kJ/mol	90.0 ± 0.7	93.0 ± 1.0
Δ*S*^‡^ / J/(mol·K)	−30.0 ± 2.0	−39.0 ± 4.0

^a^At 298 K.

Counterintuitively, the calculated thermodynamic data of the transition states show, within the error margin, similar values of activation enthalpy Δ*H***^‡^** and Δ*S*^‡^. In particular, **NAc-PAP-CN** showed a negative Δ*S***^‡^** = −30.0 ± 2.0 J/(mol·K), while **NAc-PAP-OMe** showed a relatively similar value (−39.0 ± 4.0 J/(mol·K)), hinting towards the same mechanism of relaxation operating for both compounds. Comparing these values to the calculations for azobenzene, we hypothesize that both compounds undergo isomerization via triplet intermediacy [34].

Contrary to what was observed by Reimann et al. [34], however, we did not observe any heavy atom effect. This could be explained by the different ways in which the heavy atom is introduced: in our case as a substituent, in the case of the literature example by adding tetrabutylammonium iodide to the solution.

## Conclusion

In this study, we synthesized and systematically investigated various **PAP** derivatives, including **NAc-PAP**s, **NMe-PAP**s, and **NH-PAP**s. As similar functional groups were reported as highly beneficial for the photochemical properties in other classes of photoswitches, our focus was on the novel **NAc-PAP**s, which exhibit an acetyl group on one of the pyrazole nitrogens. The functional group could be installed easily via acetylation from the corresponding **NH-PAP**s in high yields to result in a set of eleven novel compounds that we could compare to a set of 22 **NMe-PAP**s and **NH-PAP**s.

We then analyzed the molecules' photophysical and photochemical properties and studied the metastable isomers' thermal relaxation mechanism. In particular, photophysical studies highlighted the impact of structural modifications on the π→π* and n→π* transitions, showing that substitution of nitrogen with methyl or acetyl groups resulted in a small bathochromic shift and hyperchromic effects.

Anti-Kasha behavior was observed with distinct trends in the π→π* and n→π* transitions when studying the quantum yields (Φ_E→Z_ and Φ_Z→E_ ). Strong EWGs or EDGs enhanced the quantum yields for the π→π* transitions, whereas the n→π* transitions exhibited no clear correlation with substitution patterns. Notably, the acetylation of nitrogen significantly increased the Φ_E→Z_ for π→π* transitions in almost all compounds studied (excluding **NAc-PAP-H** and **-Me**), even surpassing the effects of methylation.

Hammett analysis showed that the thermal population of the triplet state seem to be preferred as the thermal relaxation mechanisms of the back isomerization. EWGs and EDGs accelerated the relaxation dynamics compared to **NAc-PAP-H**. Acylation of the pyrazole moiety led to an enhanced metastable half-life compared to the **NH-PAPs**. For **NAc-PAP-H**, we observed increased half-lifes (21.5 days, 30 °C), compared to the reported **NH-PAP-H** (0.066 days; 25 °C [51]) or **NMe-PAP-H** (10 days; 25 °C [31], all in CH_3_CN). In the presence of OH as substituent, tautomerism can become feasible and result in particularly fast relaxation rates. These results highlight the complex interplay between electronic effects and thermal isomerization pathways in this class of compounds.

To summarize, this work provides a comprehensive understanding of how structural modifications affect the synthesis, photochemical, and thermal behavior of **PAP** derivatives introducing **NAc-PAPs** as novel compound set with enhanced photochemical performance. Our findings provide valuable guidance for designing functional **PAP**s with tailor-made photochemistry and photophysical properties, which may broaden their application in areas such as molecular switches, photodynamic materials, and optoelectronics.

## Supporting Information

File 1 Materials and methods, analytical equipment, experimental procedures, compound characterization, UV–vis spectra at different concentrations, photochemical experiments, thermal isomerization analysis, and NMR spectra.

## Data Availability

All data that supports the findings of this study is available in the published article and/or the supporting information of this article.
