# Supplementary material for: Substituent effects in N-acetylated phenylazopyrazole photoswitches
Source: Beilstein J Org Chem. 2025 Apr 25;21:830–8. doi: 10.3762/bjoc.21.66 (PMC12035873; doi:10.3762/bjoc.21.66)
Supplement: File 1 — Materials and methods, analytical equipment, experimental procedures, compound characterization, UV–vis spectra at different concentrations, photochemical experiments, thermal isomerization analysis, and NMR spectra. [file Beilstein_J_Org_Chem-21-830-s001.pdf]

BEILSTEIN JOURNAL OF ORGANIC CHEMISTRY

Supporting Information

for

Substituent effects in *N*-acetylated phenylazopyrazole photoswitches

Radek Tovtik, Dennis Marzin, Pia Weigel, Stefano Crespi and Nadja A. Simeth

Beilstein J. Org. Chem. **2025,** *21,* 830–838. doi:10.3762/bjoc.21.66

Materials and methods, analytical equipment, experimental procedures, compound characterization, UV–vis spectra at different concentrations, photochemical experiments, thermal isomerization analysis, and NMR spectra

License and Terms: This is a supporting information file under the terms of the Creative Commons Attribution License (https://creativecommons.org/licenses/by/4.0). Please note that the reuse, redistribution and reproduction in particular requires that the author(s) and source are credited and that individual graphics may be subject to special legal provisions.
The license is subject to the *Beilstein Journal of Organic Chemistry* terms and conditions: (https://www.beilstein-journals.org/bjoc/terms)

Table of contents

1. Materials and methods S2

2. Synthesis S3

2.1 General procedure 1 to synthesize 3-(2-phenylhydrazono)pentane-2,4-diones S3

2.2 General procedure 2 to synthesize 1,3-dimethyl-arylazopyrazoles S6

2.3 General procedure 3 to synthesize (*E*)-1-(4-(diazenyl-3,5-dimethyl-1*H*-pyrazol-1-yl)ethan-1-one S11

2.4 pH-Dependent stability of **NAc-PAP-H** S15

3. Photochemical and photophysical studies S17

3.1 Determination of photostationary state distribution by ^{1}H NMR S17

3.2 Cyclic irradiation to study fatigue resistance S23

3.3 Determination of molar extinction coefficients S27

3.3.1 Absorbance vs. concentrations of **NAc-PAP** derivatives S27

3.3.2 Absorbance vs. concentrations of **NMe-PAP** derivatives S31

3.3.3 Absorbance vs. concentrations of **NH-PAP** derivatives S35

3.4 Chemical actinometry S39

3.5 Determination of quantum yields S39

3.5.1 Irradiation of **NAc-PAP** derivatives and evaluation of the kinetic traces S40

3.5.2 Irradiation of **NH-PAP** derivatives and evaluation of the kinetic traces S48

3.5.3 Irradiation of **NMe-PAP** derivatives and evaluation of the kinetic traces S52

3.6 Determination of thermal half-lives S56

3.7 Hammett correlation of thermal half-lives of **NAc-PAP** derivatives S60

3.7.1 Thermal relaxation of **NAc-PAP-CN** at different temperatures S62

3.7.2 Thermal relaxation of **NAc-PAP-OMe** at different temperatures S65

4. NMR Spectra S68

5. References S160

1. Materials and methods

Reagents and solvents: Reagents and solvents were purchased in the highest grade of purity available. Dry solvents were purchased from Sigma-Aldrich (Taufkirchen, Germany). Technical solvents were distilled before use. HPLC-grade solvents have been used for photoswitching and kinetics studies. For column chromatography, distilled solvents have been utilized. Solvents for the NMR measurements were supplied by Deuterio (Kastellaun, Germany) or Eurisotop (Saarbrücken, Germany).

Chromatography: TLC was performed on 0.25 mm silica-gel 60 F plates with a 254 nm fluorescence indicator from Merck (Darmstadt, Germany). The substance detection took place by light with wavelengths of 254 nm and 360 nm. Non-UV-active substances have been visualized by the following TLC stains: ninhydrin solution (1.5 g ninhydrin, 3 mL acetic acid in 100 mL *n*-butanol) or potassium permanganate solution (3.0 g $KMnO_4$, 20 g K_2CO_3 and 2.5 mL NaOH (10%) in 400 mL water) and gentle heating afterwards.

Flash column chromatography was performed using silica-gel of the type Geduran® Si 60 (40–63 µm mesh ASTM) purchased from Merck (Darmstadt, Germany). Columns were packed with wet silica gel and the samples were loaded as a concentrated solution or as a silica pad.

NMR spectroscopy: ^{1}H, ^{13}C, and ^{19}F NMR spectra were recorded on 300 or 400 MHz spectrometers; ^{13}C NMR spectra were obtained on 101 or 75 MHz instruments. ^{1}H chemical shifts (δ) are reported in parts per million (ppm) relative to DMSO-d_6 (δ= 2.50 ppm), CD_3OD (δ= 3.31 ppm), $CDCl_3$ (δ= 7.26 ppm), or CD_3CN (δ= 1.94 ppm) as internal references. ^{13}C δ are reported in ppm with DMSO-d_6 (δ= 77.67 ppm), CD_3CN (δ= 118.26, 1.32 ppm) as internal references. ^{19}F NMR spectra were measured without any internal standard to qualitatively confirm the structure and purity of the desired product.

MS/HRMS: Electron spray ionization mass spectrometry (ESI-MS) and high-resolution ESI (HRMS) were performed on a maXis or MicroTOF spectrometer from Bruker (Bremen Germany).

UV–vis spectroscopy: UV–vis absorption spectroscopy was performed on a Specord S600 or Jasco V-670 in quartz cuvettes (path 1.00 cm) at a controlled temperature of 20 °C. Molar extinction coefficients (ε) were determined by fitting the slope of absorbance dependency to the concentration taken from at least three separate dilutions. Photoisomerization was

measured under 365 (at concentration 12.5 µM) or 445 nm (at concentration 50 µM) irradiation at 25% or 10% intensity.

2. Synthesis

2.1 General procedure 1 to synthesize 3-(2-phenylhydrazono)pentane-2,4-diones

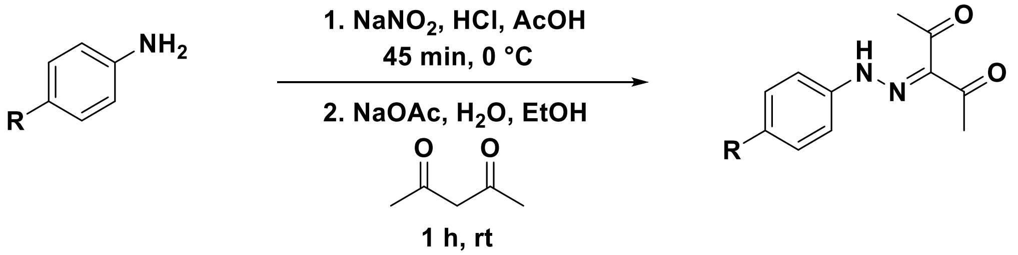

The synthesis was adopted from Weston et al.[1] The starting compound (10.74 mmol, 1.0 equiv) was dissolved in AcOH (16.1 mL) and HCl (12 M, 2.5 mL) and in water dissolved $NaNO_2$ (0.89 g, 12.9 mmol, 1.3 equiv) was added after cooling to 0 °C. The mixture was stirred for 45 minutes and then added dropwise to a solution of pentane-2,4-dione (1.4 mL, 14.0 mmol, 1.3 equiv) and NaOAc (2.64 g, 32.22 mmol, 3.0 equiv) in water (6.4 mL) and EtOH (10.7 mL). After stirring for 1 hour the solution was vacuum filtrated to collect the yellow solid. It was washed with water, water/EtOH 1:1, and hexane and dried in vacuo.

3-(2-Phenylhydrazono)pentane-2,4-dione

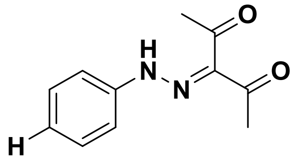

Yield: 0.85 g (4.15 mmol, 38 %). **^{1}H NMR** (400 MHz, DMSO−d_6): δ (ppm) = 14.05 (s, 1H), 7.59 – 7.54 (m, 2H), 7.46 7.39 (m, 2H), 7.22 – 7.16 (m, 1H), 2.44 (s, 6H). **^{13}C NMR** (101 MHz, DMSO−d_6): δ (ppm) = 196.5, 141.8, 133.3, 129.6, 125.4, 116.3, 31.2, 26.5. **MS(EI) m/z**: 205.1 $[M+H]^+$, 227.1 $[M+Na]^+$. The spectral data are in accordance with the literature.[2,3]

3-(2-(4-Fluorophenyl)hydrazono)pentane-2,4-dione

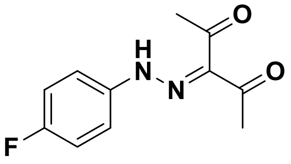

Yield: 2.16 g (9.71 mmol, 90 %). **^{1}H NMR** (400 MHz, DMSO−d_6): δ (ppm) = 14.03 (s, 1H), 7.65 – 7.59 (m, 2H), 7.30– 7.23 (m, 2H), 2.43 (s, 6H). **^{13}C NMR** (101 MHz, DMSO−d_6): δ (ppm) = 196.4, 160.9, 158.5, 138.5, 138.4, 133.3, 118.2, 118.1, 116.4, 116.2, 31.0, 26.4. **^{19}F NMR** (282 MHz, DMSO-d_6): δ

(ppm) = -117.4. **MS(EI) m/z**: 223.1 [M+H]$^+$, 245.1 [M+Na]$^+$. The spectral data are in accordance with the literature.[3]

3-(2-(4-Hydroxyphenyl)hydrazono)pentane-2,4-dione

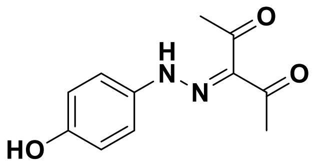

Yield: 0.60 g (2.72 mmol, 25 %). **^{1}H NMR** (300 MHz, DMSO–d$_6$): δ (ppm) = 14.49 (s, 1H), 9.65 (s, 1H), 7.46 – 7.40 (m, 2H), 6.87 – 6.79 (m, 2H), 2.45 (s, 3H), 2.37 (s, 3H). **^{13}C NMR** (75 MHz, DMSO–d$_6$): δ (ppm) = 196.0, 155.9, 133.7, 132.1, 118.1, 116.1, 31.1, 26.4. **MS(EI) m/z**: 243.1 [M+Na]$^+$. The spectral data are in accordance with the literature.[3]

3-(2-(4-Bromophenyl)hydrazono)pentane-2,4-dione

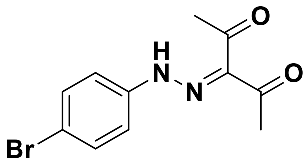

Yield: 2.57 g (9.09 mmol, 85 %) **^{1}H NMR** (400 MHz, CDCl$_3$): δ (ppm) = 14.65 (s, 1H), 7.52 – 7.48 (m, 2H), 7.27 (d, J = 7.0 Hz, 2H), 2.59 (s, 3H), 2.47 (s, 3H). **^{13}C NMR** (101 MHz, CDCl$_3$): δ (ppm) = 198.3, 197.0, 140.8, 133.6, 132.8, 118.7, 117.8, 31.8, 26.7. **MS(EI) m/z**: 283.0, 285.0 [M+Na]$^+$. The spectral data are in accordance with the literature.[3]

3-(2-(*p*-Tolyl)hydrazono)pentane-2,4-dione

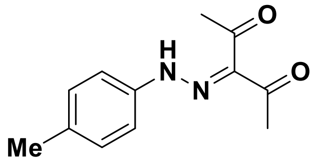

Yield: 1.74 g (7.98 mmol, 74 %). **^{1}H NMR** (400 MHz, DMSO–d$_6$): δ (ppm) = 14.17 (s, 1H), 7.48 – 7.44 (m, 2H), 7.26 – 7.21 (m, 2H), 2.43 (s, 6H), 2.30 (s, 3H). **^{13}C NMR** (101 MHz, DMSO–d$_6$): δ (ppm) = 196.3, 139.4, 134.9, 132.9, 130.0, 116.3, 31.1, 26.3, 20.5. **MS(EI) m/z**: 219.1 [M+H]$^+$, 241.1 [M+Na]$^+$. The spectral data are in accordance with the literature.[2,3]

3-(2-(4-Iodophenyl)hydrazono)pentane-2,4-dione

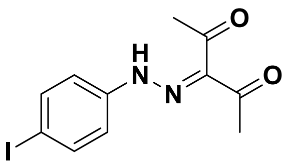

Yield: 3.15 g (9.56 mmol, 89 %). **^{1}H NMR** (400 MHz, DMSO–d$_6$): δ (ppm) = 13.81 (s, 1H), 7.76 – 7.71 (m, 2H), 7.41 – 7.37 (m, 2H), 2.43 (s, 6H). **^{13}C NMR** (101 MHz, DMSO–d$_6$): δ (ppm) = 196.8, 141.8, 138.1, 133.8, 118.4, 89.3, 31.2, 26.3. **MS(EI) m/z**: 331.0 [M+H]$^+$. The spectral data are in accordance with the literature.[3]

3-(2-(4-(Trifluoromethyl)phenyl)hydrazono)pentane-2,4-dione

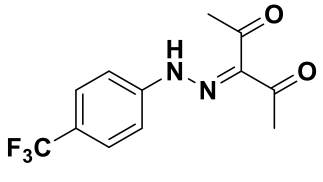

Yield: 2.76 g (10,16 mmol, 95 %). **^{1}H NMR** (300 MHz, $CDCl_3$): δ (ppm) = 14.58 (s, 1H), 7.66 (d, J = 8.3 Hz, 2H), 7.48 (d, J = 8.4 Hz, 2H), 2.62 (s, 3H), 2.50 (s, 3H). **^{13}C NMR** (75 MHz, $CDCl_3$): δ (ppm) = 198.6, 197.1, 144.4, 134.3, 127.7, 127.1, 127.1, 116.1, 31.9, 26.8. **^{19}F NMR** (282 MHz, $CDCl_3$): δ (ppm) = -66.54. The spectral data are in accordance with the literature.[2,3]

3-(2-(4-Chlorophenyl)hydrazono)pentane-2,4-dione

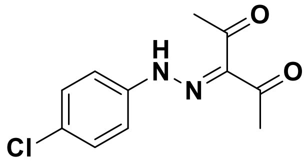

Yield: 2.17 g (9.10 mmol, 85 %). **^{1}H NMR** (400 MHz, DMSO–d_6): δ (ppm) = 13.84 (s, 1H), 7.62 – 7.57 (m, 2H), 7.49 – 7.43 (m, 2H), 2.43 (s, 6H). **^{13}C NMR** (101 MHz, DMSO–d_6): δ (ppm) = 196.5, 140.9, 133.8, 129.4, 129.0, 117.9, 31.2, 26.4. **MS(EI) m/z**: 239.06 $[M+H]^+$, 261.04 $[M+Na]^+$. The spectral data are in accordance with the literature. [3]

4-(2-(2,4-Dioxopentan-3-ylidene)hydrazineyl)benzonitrile

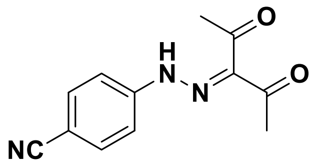

Yield: 1.24 g (5.39 mmol, 50 %). **^{1}H NMR** (300 MHz, DMSO–d_6): δ (ppm) = 13.39 (s, 1H), 7.88 – 7.81 (m, 2H), 7.73 – 7.66 (m, 2H), 2.47 (s, 3H), 2.42 (s, 3H). **^{13}C NMR** (75 MHz, DMSO–d_6): δ (ppm) = 197.6, 196.5, 145.9, 135.8, 133.8, 119.0, 116.4, 106.1, 31.3, 26.3. **MS(EI) m/z**: 252.1 $[M+Na]^+$. The spectral data are in accordance with the literature.[2]

3-(2-(4-Methoxyphenyl)hydrazono)pentane-2,4-dione

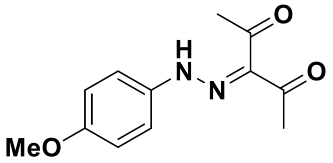

Yield: 0.55 g (2.36 mmol, 22 %). **^{1}H NMR** (300 MHz, DMSO–d_6): δ (ppm) = 14.36 (s, 1H), 7.58 – 7.51 (m, 2H), 7.05 – 6.97 (m, 2H), 3.77 (s, 3H), 2.48 – 2.35 (m, 6H). **^{13}C NMR** (75 MHz, DMSO–d_6): δ (ppm) = 196.1, 157.4, 135.2, 132.5, 117.9, 114.9, 55.4, 31.1, 26.4. **MS(EI) m/z**: 257.1 $[M+Na]^+$. The spectral data are in accordance with the literature.[2,3]

3-(2-(4-Nitrophenyl)hydrazono)pentane-2,4-dione

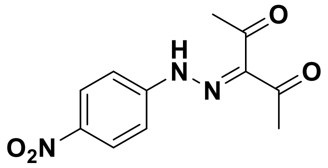

Yield: 1.61 g (6.47 mmol, 60%). **^{1}H NMR** (400 MHz, CD_2Cl_2): δ (ppm) = 14.43 (s, 1H), 8.36 – 8.16 (m, 2H), 7.73 – 7.43 (m, 2H), 2.59 (s, 3H), 2.49 (s, 3H). (s, 3H. **^{13}C NMR** (101 MHz, CD_2Cl_2): (ppm) =

199.0, 197.1, 147.3, 144.8, 135.5, 126.0, 116.2, 31.9, 26.8. **MS(EI) m/z**: 272.1 [M+Na]⁺. The spectral data are in accordance with the literature.[2,3]

2.2 General procedure 2 to synthesize 1,3-dimethyl-arylazopyrazoles

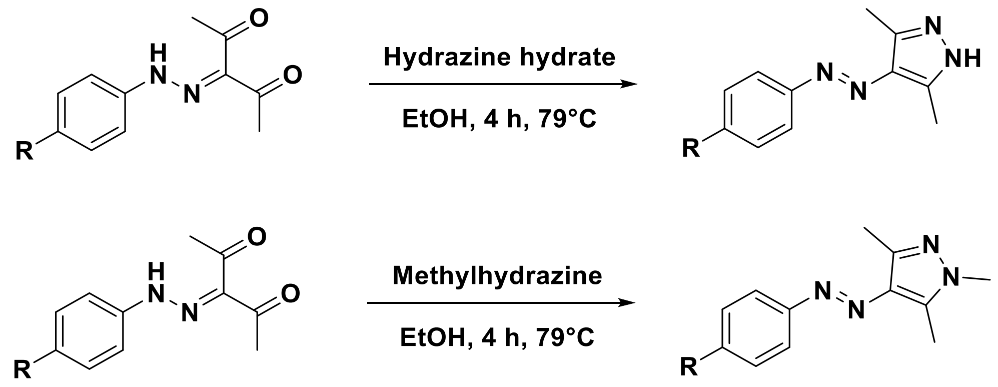

The synthesis was adopted from Patel et al.[4] The 3-(2-phenylhydrazono)pentane-2,4-dione (0.5 mmol, 1 equiv) was dissolved in EtOH (7 mL) and then the hydrazine hydrate/methylhydrazine (0.5 mmol, 1 equiv) was added. The solution was refluxed for 4 hours, and the solvent was removed in vacuo.

(*E*)-3,5-Dimethyl-4-(phenyldiazenyl)-1*H*-pyrazole

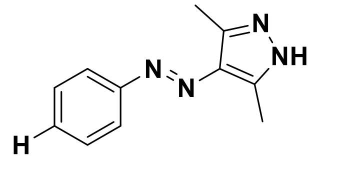

Yield: 101,5 mg (quant.) ^{1}H NMR (400 MHz, $CDCl_3$) δ (ppm) = 7.74 – 7.69 (m, 2H), 7.54 – 7.48(m, 2H), 7.44 – 7.40 (m, 1H), 2.6 (s, 6H). **^{13}C NMR** (101 MHz, $CDCl_3$): δ (ppm) = 153.7, 141.7, 134.9, 129.7, 129.1, 122.0, 12.3. **MS(EI) m/z**: 201.1 [M+H]⁺, 223.1 [M+Na]⁺. The spectral data are in accordance with the literature.[5]

(*E*)-1,3,5-Trimethyl-4-(phenyldiazenyl)-1*H*-pyrazole

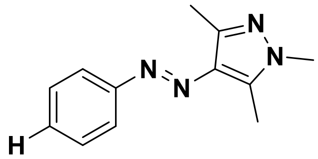

Yield: 88.0mg (0.41 mmol, 82 %). **^{1}H NMR** (400 MHz, DMSO-d_6): δ (ppm) = 7.75 - 7.70 (m, 2H), 7.54 - 7.48 (m, 2H), 7.46 - 7.39 (m, 1H), 3.74 (s, 3H), 2.55 (s, 3H), 2.37 (s, 3H). **^{13}C NMR** (101 MHz, DMSO-d_6): δ (ppm) = 153.0, 140.3, 139.6, 134.4, 129.5, 129.2, 121.4, 36.0, 13.8, 9.5. **MS(EI) m/z**: 215.13 [M+H]⁺, 237.11 [M+Na]⁺. The spectral data are in accordance with the literature.[1]

(*E*)-4-((4-Fluorophenyl)diazenyl)-3,5-dimethyl-1*H*-pyrazole

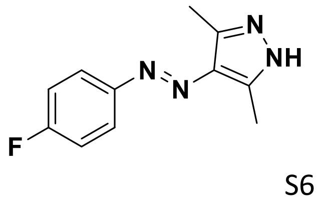

Yield: 111.4mg (quant.). **^{1}H NMR** (400 MHz, DMSO-d_6): δ (ppm) = 12.84 (s, 1H), 7.80 - 7.73 (m, 2H), 7.36 - 7.29 (m, 2H), 2.44 (s, 6H).

^{13}**C NMR** (101 MHz, DMSO-d_6): δ (ppm) = 163.8, 161.3, 149.8, 134.0, 123.4, 123.3, 116.1, 115.9, 13.7, 10.0. **MS(EI) m/z**: 219.10 $[M+H]^+$, 241.08 $[M+Na]^+$. The spectral data are in accordance with the literature.[5,6]

(*E*)-4-((4-Fluorophenyl)diazenyl)-1,3,5-trimethyl-1*H*-pyrazole

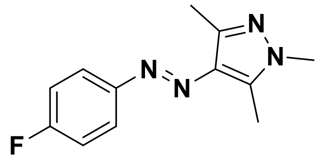

Yield: 157.7mg (quant.). ^{1}**H NMR** (300 MHz, DMSO-d_6): δ (ppm) = 7.78 (dd, J = 9.2, 5.3 Hz, 2H), 7.34 (t, J = 8.9 Hz, 2H), 3.74 (s, 3H), 2.54 (s, 3H), 2.36 (s, 3H). ^{13}**C NMR** (75 MHz, DMSO-d_6): δ (ppm) = 161.0, 149.7, 140.3, 139.7, 123.3, 116.2, 115.9, 36.0, 13.8, 9.5. ^{19}**F NMR** (282 MHz, DMSO-d_6): δ (ppm) = -112.4. **MS(EI) m/z**: 233.12 $[M+H]^+$, 255,10 $[M+Na]^+$.

(*E*)-4-((3,5-Dimethyl-1*H*-pyrazol-4-yl)diazenyl)phenol

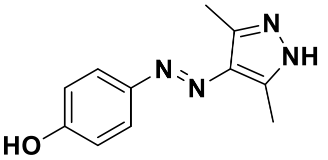

Yield: 105.1mg (0.49 mmol, 98 %). ^{1}**H NMR** (300 MHz, DMSO-d_6): δ (ppm) 12.67 (s, 1H), 9.91 (s, 1H), 7.65 – 7.56 (m, 2H), 6.92 – 6.83 (m, 2H), 2.48 – 2.34 (m, 6H). ^{13}**C NMR** (75 MHz, DMSO-d_6): δ (ppm) = 159.1, 146.1, 133.7, 123.1, 115.6, 31.1, 26.4. **MS(EI) m/z**: 217.1 $[M+H]^+$, 215.1 $[M-H]^-$. The spectral data are in accordance with the literature.[6]

(*E*)-4-((1,3,5-Trimethyl-1*H*-pyrazol-4-yl)diazenyl)phenol

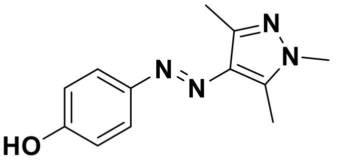

Yield: 75.5mg (0.33 mmol, 66 %). ^{1}**H NMR** (300 MHz, DMSO-d_6): δ (ppm) = 9.93 (s, 1H), 7.65 - 7.57 (m, 2H), 6.90 - 6.83 (m, 2H), 3.71 (s, 3H), 2.51 (s, 3H), 2.34 (s, 3H). ^{13}**C NMR** (75 MHz, DMSO-d_6): δ (ppm) = 196.0, 159.1, 155.9, 146.1, 139.9, 138.2, 134.0, 123.2, 118.1, 116.14, 115.6, 35.9, 31.1, 13.7, 9.4. **MS(EI) m/z**: 231.1 $[M+H]^+$, 253.1 $[M+Na]^+$. The spectral data are in accordance with the literature.[7]

(*E*)-4-((4-Bromophenyl)diazenyl)-3,5-dimethyl-1*H*-pyrazole

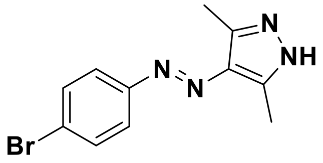

Yield: 144.0mg (0.49 mmol, 98 %). ^{1}**H NMR** (300 MHz, DMSO-d_6): δ (ppm) = 12.89 (s, 1H), 7.71 - 7.62 (m, 4H), 2.43 (d, J = 7.3 Hz, 6H). ^{13}**C NMR** (75 MHz, DMSO-d_6): δ (ppm) = 196.5, 151.9, 141.4, 134.2, 132.3, 123.3, 122.4, 13.6, 10.1. **MS(EI) m/z**: 279.0 $[M+H]^+$, 281.0 $[M+H]^+$. The spectral data are in accordance with the literature.[5,6]

(*E*)-4-((4-Bromophenyl)diazenyl)-1,3,5-trimethyl-1*H*-pyrazole

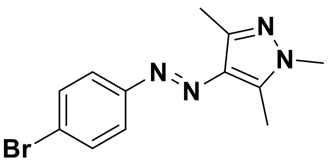

Yield: 147.6mg (quant.). **^{1}H NMR** (300 MHz, DMSO-d$_6$): δ (ppm) = 7.73 - 7.64 (m, 4H), 3.74 (s, 3H), 2.54 (s, 3H), 2.36 (s, 3H). **^{13}C NMR** (75 MHz, DMSO-d$_6$): δ (ppm) = 151.9, 140.5, 140.1, 134.4, 132.2, 123.3, 122.4, 36.0, 13.8, 9.5. **MS(EI) m/z**: 293.0 [M+H]$^+$, 295.0 [M+H]$^+$, 315.0 [M+Na]$^+$, 317.0 [M+Na]$^+$.

(*E*)-3,5-Dimethyl-4-(*p*-tolyldiazenyl)-1*H*-pyrazole

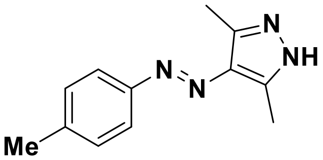

Yield: 113.9mg (quant.). **^{1}H NMR** (300 MHz, DMSO-d$_6$): δ (ppm) = 12.79 (s, 1H), 7.65 - 7.60 (m, 2H), 7.33 - .28 (m, 2H), 2.44 (s, 6H), 2.36 (s, 3H), 2.30 (s, 3H). **^{13}C NMR** (75 MHz, DMSO-d$_6$): δ (ppm) = 151.0, 139.2, 134.0, 129.7, 121.3, 20.9, 20.5. **MS(EI) m/z**: 215.13 [M+H]$^+$, 237.11 [M+Na]$^+$. The spectral data are in accordance with the literature. [5,6]

(*E*)-1,3,5-Trimethyl-4-(*p*-tolyldiazenyl)-1*H*-pyrazole

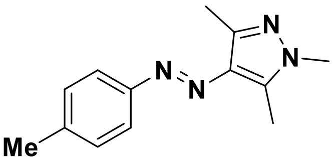

Yield: 126.2mg (quant.). **^{1}H NMR** (300 MHz, DMSO−d$_6$): δ (ppm) = 7.63 (d, J = 8.4 Hz, 2H), 7.30 (d, J = 8.7Hz, 2H), 3.73 (s, 3H), 2.53 (s, 3H), 2.36 (s, 6H). **^{13}C NMR** (75 MHz, DMSO-d$_6$): δ (ppm) = 151.0, 140.2, 139.3, 130.0, 129.7, 121.3, 116.3, 35.9, 20.9, 13.7, 9.4. **MS(EI) m/z**: 229.1 [M+H]$^+$, 251.1 [M+Na]$^+$. The spectral data are in accordance with the literature.[1]

(*E*)-4-((4-Iodophenyl)diazenyl)-3,5-dimethyl-1*H*-pyrazole

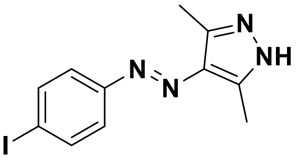

Yield: 166.5mg (0.35 mmol, 70 %). **^{1}H NMR** (400 MHz, DMSO-d$_6$): δ (ppm) = 12.89 (s, 1H), 7.90 - 7.84 (m, 2H), 7.53 - 7.48 (m, 2H), 2.49 (s, 3H), 2.39 (d, J = 5.9 Hz, 3H). **^{13}C NMR** (101 MHz, DMSO−d$_6$): δ (ppm) = 196.3, 152.4, 138.0, 123.4, 95.8, 26.4, 10.1. **MS(EI) m/z**: 327.0 [M+H]$^+$. The spectral data are in accordance with the literature.[8]

(*E*)-4-((4-Iodophenyl)diazenyl)-1,3,5-trimethyl-1*H*-pyrazole

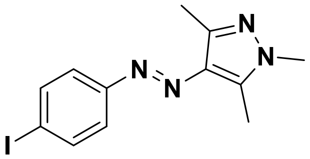

Yield: 168.3mg (0.49 mmol, 98 %). **^{1}H NMR** (300 MHz, DMSO-d$_6$): δ (ppm) = 7.91 - 7.82 (m, 2H), 7.56 - 7.47 (m, 2H), 3.73 (s, 3H), 2.53 (s, 3H), 2.35 (s, 3H). **^{13}C NMR** (75 MHz, DMSO−d$_6$): δ (ppm) = 152.3,

140.5, 140.0, 138.0, 134.4, 123.4, 95.9, 36.0, 13.8, 9.5. **MS(EI) m/z**: 341.0 $[M+H]^+$, 363.0 $[M+Na]^+$. The spectral data are in accordance with the literature.[9,10]

(*E*)-3,5-Dimethyl-4-((4-(trifluoromethyl)phenyl)diazenyl)-1*H*-pyrazole

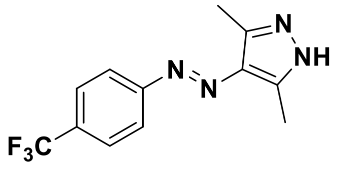

Yield: 125.3mg (0.34 mmol, 68 %). **^{1}H NMR** (400 MHz, DMSO-d$_6$): δ (ppm) = 12.99 (s, 1H), 7.89 - 7.82 (m, 4H), 2.45 (s, 6H). **^{13}C NMR** (101 MHz, DMSO–d$_6$): δ (ppm) = 155.3, 134.6, 126.4, 126.4, 121.9, 116.4. **^{19}F NMR** (282 MHz, DMSO–d$_6$): δ (ppm) = -60.77. **MS(EI) m/z**: 269.1 $[M+H]^+$. The spectral data are in accordance with the literature.[6]

(*E*)-1,3,5-Trimethyl-4-((4-(trifluoromethyl)phenyl)diazenyl)-1*H*-pyrazole

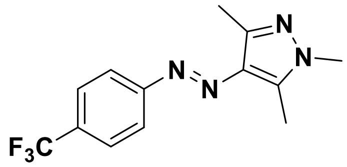

Yield: 130.3mg (0.46 mmol, 92%). **^{1}H NMR** (300 MHz, DMSO-d$_6$): δ (ppm) = 7.87 (d, J = 1.8 Hz, 4H), 3.75 (s, 3H), 2.57 (s, 3H), 2.38 (s, 3H). **^{13}C NMR** (75 MHz, DMSO–d$_6$): δ (ppm) = 155.3, 140.8, 134.8, 126.4, 121.9, 36.0, 13.8, 9.5. **^{19}F NMR** (282 MHz, $CDCl_3$): δ (ppm) = -62.34. **MS(EI) m/z**: 283.1 $[M+H]^+$.

(*E*)-4-((4-Chlorophenyl)diazenyl)-3,5-dimethyl-1*H*-pyrazole

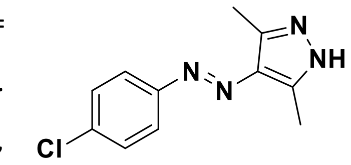

Yield: 118.6mg (quant.). **^{1}H NMR** (300 MHz, DMSO–d$_6$): δ (ppm) = 12.89 (s, 1H), 7.75 - 7.71 (m, 2H), 7.58 - 7.53 (m, 2H), 2.43 (s, 6H). **^{13}C NMR** (75 MHz, DMSO–d$_6$): δ (ppm) = 151.6, 134.2, 133.7, 129.4, 129.3, 123.0, 117.9. **MS(EI) m/z**: 235.1 $[M+H]^+$. The spectral data are in accordance with the literature.[6]

(*E*)-4-((4-Chlorophenyl)diazenyl)-1,3,5-trimethyl-1*H*-pyrazole

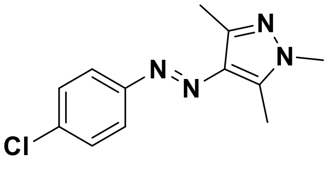

Yield: 123.6mg (quant.). **^{1}H NMR** (300 MHz, DMSO–d$_6$): δ (ppm) = 7.76 - 7.70 (m, 2H), 7.59 - 7.52 (m, 2H), 3.73 (s, 3H), 2.54 (s, 3H), 2.36 (s, 3H). **^{13}C NMR** (75 MHz, DMSO–d$_6$): δ (ppm) = 151.6, 140.5, 140.0, 134.4, 133.7, 129.2, 123.0, 117.9, 36.0, 13.8, 9.5. **MS(EI) m/z**: 249.1 $[M+H]^+$, 271.1 $[M+Na]^+$.

(*E*)-4-((3,5-Dimethyl-1*H*-pyrazol-4-yl)diazenyl)benzonitrile

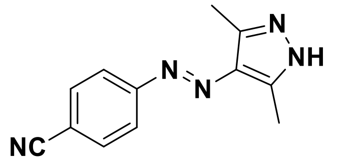

Yield: 114.9mg (quant.). **^{1}H NMR** (400 MHz, DMSO−d$_6$): δ (ppm) = 13.03 (s, 1H), 7.98 - 7.92 (m, 2H), 7.86 - 7.79 (m, 2H), 2.45 (s, 6H). **^{13}C NMR** (101 MHz, DMSO−d$_6$): δ (ppm) = 155.3, 134.8, 133.8, 133.6, 122.1, 111.1, 106.0, 30.9, 25.9. **MS(EI) m/z**: 226.1 [M+H]$^+$, 248.1 [M+Na]$^+$. The spectral data are in accordance with the literature.[2]

(*E*)-4-((1,3,5-Trimethyl-1*H*-pyrazol-4-yl)diazenyl)benzonitrile

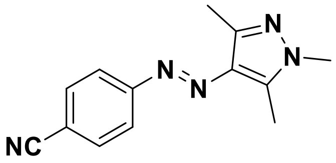

Yield: 121.0mg (quant.). **^{1}H NMR** (300 MHz, CDCl$_3$): δ (ppm) = 7.86 - 7.80 (m, 2H), 7.76 - 7.71 (m, 2H), 3.79 (s, 3H), 2.59 (s, 3H), 2.48 (s, 3H). **^{13}C NMR** (75 MHz, CDCl$_3$): δ (ppm) = 155.9, 143.0, 140.5, 135.8, 133.2, 122.5, 119.0, 116.4, 112.1, 36.3, 14.1, 10.2. **MS(EI) m/z**: 240.1 [M+H]$^+$, 262.1 [M+Na]$^+$.

(*E*)-4-((4-Methoxyphenyl)diazenyl)-3,5-dimethyl-1*H*-pyrazole

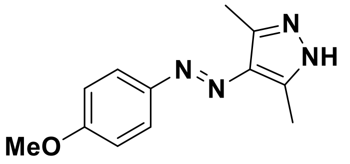

Yield: 115.3mg (quant.). **^{1}H NMR** (300 MHz, DMSO−d$_6$): δ (ppm) = 12.73 (s, 1H), 7.70 (d, J = 9.0 Hz, 2H), 7.04 (dd, J = 9.1, 2.3 Hz, 2H), 3.82 (s, 3H), 2.38 (s, 6H). **^{13}C NMR** (75 MHz, DMSO−d$_6$): δ (ppm) = 160.4, 147.2, 133.8, 122.9, 117.9, 114.9, 114.3, 55.4, 13.7, 10.0. **MS(EI) m/z**: 231.1 [M+H]$^+$. The spectral data are in accordance with the literature.[5]

(*E*)-4-((4-Methoxyphenyl)diazenyl)-1,3,5-trimethyl-1*H*-pyrazole

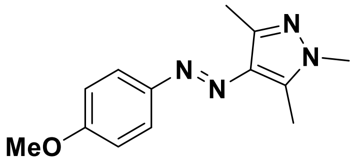

Yield: 122.2mg (quant.). **^{1}H NMR** (300 MHz, DMSO−d$_6$): δ (ppm) = 7.74 - 7.67 (m, 2H), 7.04 (dd, J = 9.1, 2.2 Hz, 2H), 3.82 (s, 3H), 3.72 (s, 3H), 2.52 (s, 3H), 2.35 (s, 3H). **^{13}C NMR** (75 MHz, DMSO−d$_6$): δ (ppm) = 196.1, 160.4, 147.1, 140.1, 138.6, 123.0, 114.3, 55.5, 35.9, 13.7, 9.4. **MS(EI) m/z**: 245.1 [M+H]$^+$, 267.1 [M+Na]$^+$. The spectral data are in accordance with the literature.[4]

(*E*)-3,5-Dimethyl-4-((4-nitrophenyl)diazenyl)-1*H*-pyrazole

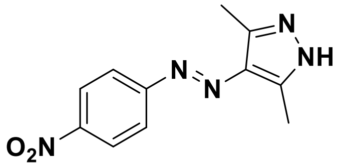

Yield: 116.3mg (0.47 mmol, 94 %). **^{1}H NMR** (400 MHz, DMSO-d$_6$): δ (ppm) = 13.07 (s, 1H), 8.38 - 8.32 (m, 2H), 7.91 - 7.86 (m, 2H), 2.47 (s, 6H). **^{13}C NMR** (101 MHz, DMSO-d$_6$):

δ (ppm) = 156.7, 147.0, 135.1, 124.9, 122.2, 30.7. **MS(EI) m/z**: 246.1 $[M+H]^+$. The spectral data are in accordance with the literature.[5,6]

(*E*)-1,3,5-Trimethyl-4-((4-nitrophenyl)diazenyl)-1*H*-pyrazole

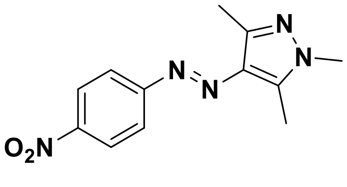

Yield: 129.5mg (quant.). **^{1}H NMR** (400 MHz, DMSO−d$_6$): δ (ppm) = 8.36 - .31 (m, 2H), 7.91 - 7.86 (m, 2H), 3.75 (s, 3H), 2.57 (s, 3H), 2.38 (s, 3H). **^{13}C NMR** (101 MHz, DMSO−d$_6$): δ (ppm) = 156.6, 147.0, 141.5, 141.0, 135.2, 124.9, 122.2, 36.1, 13.9, 9.6. **MS(EI) m/z**: 260.1 $[M+H]^+$, 282.1 $[M+Na]^+$. The spectral data are in accordance with the literature.[4]

2.3 General procedure 3 to synthesize *(E)*-1-(4-(diazenyl-3,5-dimethyl-1*H*-pyrazol-1-yl)ethan-1-one

A solution of acetyl chloride (0.5 mol L^{-1}, 2.5 equiv) in DCM was added to the ice-cooled solution of (*E*)-3,5-dimethyl-4-((4-nitrophenyl)diazenyl)-1*H*-pyrazole derivative (1.0 equiv), $NaHCO_3$ (2.5 equiv) in DCM (0.045 mol L^{-1}), and the reaction mixture was stirred for 16 hours under N_2 atmosphere. Then, 5 mL of water was added, and the resulting solution was extracted (3 × 50 mL) with brine and (3 × 50 mL) DCM. The organic layer was separated, dried using Na_2SO_4, and concentrated under reduced pressure. The crude was purified via FCC (silica gel) to yield the desired product.

(*E*)-1-(4-((4-Nitrophenyl)diazenyl)-3,5-dimethyl-1*H*-pyrazol-1-yl)ethan-1-one

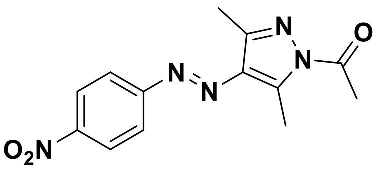

Synthetized according General procedure **3**. 0.020 g of (*E*)-3,5-dimethyl-4-((4-nitrophenyl)diazenyl)-1*H*-pyrazole was used. Mobile phase: 20 % ethyl acetate in pentane. Yellow solid (0.020 g, 85%). **Mp.**: 155.3−157.4 °C **^{1}H NMR** (400 MHz, CD_2Cl_2) δ 8.39 – 8.26 (m, 2H), 8.00 – 7.86 (m, 2H), 2.94 (s, 3H), 2.69 (s, 3H), 2.49 (s, 3H). **^{13}C NMR** (101 MHz, CD_2Cl_2) δ 172.1, 156.9, 148.7, 147.3, 145.0, 138.4, 125.1, 123.1, 23.6, 15.5, 12.5. **HRMS(ESI)** m/z: $[M]^+$ calcd. for $C_{13}H_{14}N_5O_3Na^+$ 310.0911; found 310.0911. **IR** (ATR): $\tilde{\nu}$ (cm^{-1}) 2355, 2335, 1980, 1730, 1574, 1517, 1335, 1289, 872, 772, 593.

(*E*)-1-(4-((4-Iodophenyl)diazenyl)-3,5-dimethyl-1*H*-pyrazol-1-yl)ethan-1-one

Synthetized according General procedure **3**. 0.020 g of (*E*)-3,5-dimethyl-4-((4-iodophenyl)diazenyl)-1*H*-pyrazole was used. Mobile phase: 15 % ethyl acetate in pentane. Yellow solid (0.014 g, 61%). **Mp.**: 146.3–148.2 °C. **^{1}H NMR** (400 MHz, CD_2Cl_2) δ 7.95 – 7.77 (m, 2H), 7.58 (dq, *J* = 9.2, 3.0 Hz, 2H), 2.90 (s, 3H), 2.68 (s, 3H), 2.47 (s, 3H). **^{13}C NMR** (101 MHz, CD_2Cl_2) δ 172.1, 153.0, 145.7, 145.2, 138.7, 138.0, 124.2, 97.0, 23.6, 15.4, 12.5. **HRMS(ESI)** m/z: $[M]^+$ calcd. for $C_{13}H_{13}IN_4ONa^+$ 391.0026; found 391.0026. **IR** (ATR): $\tilde{\nu}$ (cm^{-1}) 2914, 2846, 1738, 1577, 1370, 1334, 1031, 1000, 960, 889, 776, 589.

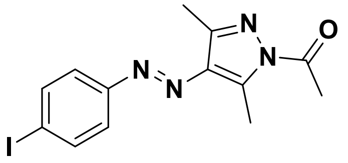

(*E*)-4-((1-Acetyl-3,5-dimethyl-1*H*-pyrazol-4-yl)diazenyl)benzonitrile

Synthetized according General procedure **3**. 0.020 g of (*E*)-4-((3,5-dimethyl-1*H*-pyrazol-4-yl)diazenyl)benzonitrilewas used. Mobile phase: 10 % ethyl acetate in pentane. Yellow solid (0.016 g, 67%). **Mp.**: 177.7–180.1 °C. **^{1}H NMR** (400 MHz, CD_2Cl_2) δ 7.93 – 7.87 (m, 2H), 7.82 – 7.74 (m, 2H), 2.93 (s, 3H), 2.68 (s, 3H), 2.48 (s, 3H). **^{13}C NMR** (101 MHz, CD_2Cl_2) δ 172.1, 155.6, 146.9, 145.1, 138.3, 133.6, 123.0, 118.9, 113.7, 23.6, 15.4, 12.5. **HRMS (ESI)** m/z: $[M]^+$ calcd. for $C_{14}H_{13}N_5ONa^+$ 290.1012; found 290.1012. **IR** (ATR): $\tilde{\nu}$ (cm^{-1}) 2227, 1739, 1369, 1340,1286, 959,851, 695, 689, 593.

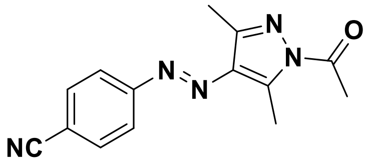

(*E*)-1-(4-((4-Methoxyphenyl)diazenyl)-3,5-dimethyl-1*H*-pyrazol-1-yl)ethan-1-one

Synthetized according General procedure **3**. 0.020 g of (*E*)-3,5-dimethyl-4-((4-methoxyphenyl)diazenyl)-1*H*-pyrazole was used. Mobile phase: 15 % ethyl acetate in pentane. Yellow solid (0.020 g, 84%). **Mp.**: 145.3–146.0 °C. **^{1}H NMR** (400 MHz, CD_2Cl_2) δ 7.87 – 7.76 (m, 2H), 7.06 – 6.94 (m, 2H), 3.87 (s, 3H), 2.89 (s, 3H), 2.67 (s, 3H), 2.48 (s, 3H). **^{13}C NMR** (101 MHz, CD_2Cl_2) δ 172.1, 162.1, 148.0, 145.4, 144.0, 137.8, 124.2, 114.5, 56.0, 23.6, 15.3, 12.4. **HR-MS (ESI)** m/z: $[M]^+$ calcd. for $C_{14}H_{17}N_4O_2^+$ 273.1346; found 273.1346. **IR** (ATR): $\tilde{\nu}$ (cm^{-1}) 2960, 2918, 2831, 1736, 1593, 1343, 1244, 1028, 838, 587, 533.

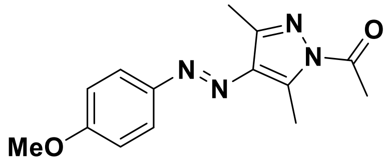

(*E*)-1-(4-((4-Chlorophenyl)diazenyl)-3,5-dimethyl-1*H*-pyrazol-1-yl)ethan-1-one

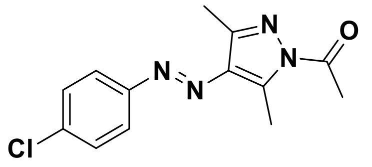

Synthesized according to General procedure **3**. 0.010 g of (*E*)-3,5-dimethyl-4-((4-chlorophenyl)diazenyl)-1*H*-pyrazole was used. Mobile phase: 10 % ethyl acetate in pentane. Yellow solid (0.0067 g, 57%). **Mp**.: 76.8–78.7 °C. **^{1}H NMR** (400 MHz, CD_2Cl_2) δ 7.91 – 7.77 (m, 2H), 7.63 – 7.46 (m, 2H), 2.95 (s, 3H), 2.72 (s, 3H), 2.52 (s, 3H). **^{13}C NMR** (101 MHz, CD_2Cl_2) δ 172.1, 152.1, 145.4 (d, J = 3.6 Hz), 137.9, 136.4, 129.6, 123.8, 23.6, 15.4, 12.5. **HR-MS (ESI)**: m/z: $[M]^+$ calcd. for $C_{13}H_{13}ClN_4ONa^+$ 299.0670; found 299.0670. **IR** (ATR): $\tilde{\nu}$ (cm^{-1}) 1667, 1506, 1406, 1389, 1085, 1081, 830, 773, 583, 522. The spectral data are in accordance with the literature.[11]

((*E*)-1-(4-((4-Bromophenyl)diazenyl)-3,5-dimethyl-1*H*-pyrazol-1-yl)ethan-1-one

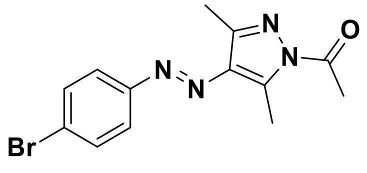

Synthetised according to General procedure **3**. 0.010 g of (*E*)-3,5-dimethyl-4-((4-bromophenyl)diazenyl)-1*H*-pyrazole was used. Mobile phase: 10 % ethyl acetate in pentane. Yellow solid (0.015 g, 65%). **Mp**.: 70.8–73.4 °C. **^{1}H NMR** (300 MHz, CD_3CN) δ 7.80 – 7.73 (m, 2H), 7.72 – 7.66 (m, 2H), 2.88 (s, 3H), 2.63 (s, 3H), 2.46 (s, 3H). **^{13}C NMR** (101 MHz, CD_2Cl_2) δ 172.2, 152.5, 145.6, 145.2, 132.6, 124.8, 124.1, 23.6, 15.4, 12.5. **HR-MS (ESI)**: m/z: $[M]^+$ calcd. for $C_{13}H_{13}BrN_4NaO^+$ 343.0165; found 343.0171. **IR** (ATR): $\tilde{\nu}$ (cm^{-1}) 2920, 2359, 2340, 1734.66, 1373, 1164, 1062, 1059, 827, 776.

(*E*)-1-(4-((4-Hydroxyphenyl)diazenyl)-3,5-dimethyl-1*H*-pyrazol-1-yl)ethan-1-one

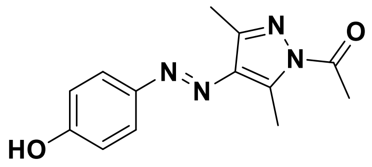

Synthetised according to General procedure **3**. 0.021 g of (*E*)-3,5-dimethyl-4-((4-hydroxyphenyl)diazenyl)-1*H*-pyrazole was used. Mobile phase: 10 % ethyl acetate in pentane. Yellow solid (0.019 g, 92%). **Mp**.: 185.1–187.0 °C. **^{1}H NMR** (400 MHz, DMSO-d_6): δ (ppm) 10.17 (s, 1H), 7.70 (d, J = 8.7 Hz, 2H), 6.90 (d, J = 8.7 Hz, 2H), 2.81 (s, 3H), 2.62 (s, 3H), 2.41 (s, 3H). **^{13}C NMR** (125 MHz, DMSO-d_6): δ (ppm) 171.4, 160.4, 145.9, 144.0, 142.6, 136.7, 124.0, 115.9, 23.2, 14.9, 12.0. **HR-MS (ESI)**: calc. for: $C_{13}H_{14}N_4O_2H$: 259.1190, found.: 259.1183. **IR** (ATR): $\tilde{\nu}$ (cm^{-1}) 3210, 2918, 1701, 1588, 1380, 1332, 1199, 1139, 841, 809.

***(E)*-1-(3,5-Dimethyl-4-(*p*-tolyldiazenyl)-1*H*-pyrazol-1-yl)ethan-1-one**

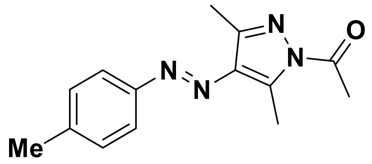

Synthetised according to General procedure **3**. 0.020 g of (*E*)-3,5-dimethyl-4-((4-methylphenyl)diazenyl)-1*H*-pyrazole was used. Mobile phase: 20 % ethyl acetate in pentane. Yellow solid (0.021 g, 84%). **Mp.**: 119.4 – 120.2 °C. **^{1}H NMR** (400 MHz, $CDCl_3$): δ (ppm) 7.75 – 7.71 (m, 2H), 7.31 – 7.26 (m, 2H), 2.93 (s, 3H), 2.71 (s, 3H), 2.50 (s, 3H), 2.42 (s, 3H). **^{13}C NMR** (125 MHz, $CDCl_3$): δ (ppm) 172.0, 151.5, 145.5, 144.4, 141.1, 137.8, 129.8, 122.3, 23.6, 21.6, 15.3, 12.5. **HR-MS (ESI)**: calc. for: $C_{14}H_{16}N_4OH$: 257.1397, found.: 257.1389. **IR** (ATR): $\tilde{\nu}$ (cm^{-1}) 2924, 1733, 1582, 1369, 1335, 1283, 962, 821, 739, 590. The spectral data are in accordance with the literature.[11]

***(E)*-1-(3,5-Dimethyl-4-(phenyldiazenyl)-1*H*-pyrazol-1-yl)ethan-1-one**

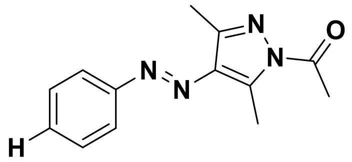

Synthetized according to general procedure **3**. 0.02 g of (*E*)-3,5-dimethyl-4-(phenyldiazenyl)-1*H*-pyrazole was used. Mobile phase: 20 % ethyl acetate in pentane. Yellow solid (16 mg, 66%). **Mp.**: 103.2 – 103.9 °C. **^{1}H NMR** (400 MHz, $CDCl_3$): δ (ppm) 7.85 – 7.80 (m, 2H), 7.52 – 7.46 (m, 2H), 7.46 – 7.40 (m, 1H), 2.94 (s, 3H), 2.71 (s, 3H), 2.51 (s, 3H). **^{13}C NMR** (125 MHz, $CDCl_3$): δ (ppm) 172.0, 153.3, 145.4, 144.9, 137.8, 130.6, 129.2, 122.3, 23.6, 15.3, 12.5. **HR-MS (ESI)**: calc. for: $C_{13}H_{14}N_4OH$: 243.1240, found.: 243.1240. **IR** (ATR): $\tilde{\nu}$ (cm^{-1}) 2924, 1733, 1571, 1394, 1372, 1346, 1289, 769, 694, 673.

(*E*)-1-(3,5-dimethyl-4-((4-(trifluoromethyl)phenyl)-diazenyl)-1*H*-pyrazol-1-yl)ethan-1-one

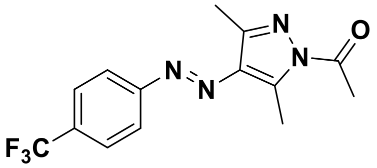

Synthesized according to general procedure **3**. 0.021 g of (*E*)-3,5-dimethyl-4-((4-(trifluoromethyl)phenyl)diazenyl)-1*H*-pyrazole was used. Mobile phase: 20 % ethyl acetate in pentane. Orange solid (19.7 mg, 95%). **Mp.**: 112.8 – 113.4 °C. **^{1}H NMR** (400 MHz, $CDCl_3$): δ (ppm) 7.90 (d, *J* = 8.2 Hz, 2H), 7.74 (d, *J* = 8.3 Hz, 2H), 2.95 (s, 3H), 2.72 (s, 3H), 2.51 (s, 3H). **^{13}C NMR** (125 MHz, $CDCl_3$): δ (ppm) 172.0, 155.2, 146.1, 145.1, 137.9, 131.8 (q, *J* = 32.4 Hz), 126.4 (q, *J* = 3.8 Hz), 125.5, 122.5, 23.6, 12.4, 12.5. **^{19}F NMR** (377 MHz, $CDCl_3$): δ (ppm) -62.5. **HR-MS (ESI)**: calc. for: $C_{14}H_{13}F_3N_4OH$: 311.1114, found.: 311.1115. **IR** (ATR): $\tilde{\nu}$ (cm^{-1}) 1747, 1372, 1337, 1321, 1278, 1167, 1116, 1113, 1065, 596.

***(E)*-1-(3,5-Dimethyl-4-((4-(fluoro)phenyl)diazenyl)-1*H*-pyrazol-1-yl)ethan-1-one**

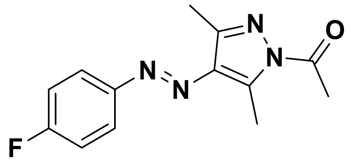

Synthesized according to general procedure **3**. 0.020 g of (*E*)-4-((4-fluorophenyl)diazenyl)-3,5-dimethyl-1*H*-pyrazole was used. Mobile phase: 20 % ethyl acetate in pentane. Orange solid (18.0 mg, 75%). %). **Mp**.: 114.7 – 115.3 °C. **^{1}H NMR** (400 MHz, $CDCl_3$): δ (ppm) 7.83 (ddt, *J* = 7.1, 5.3, 2.5 Hz, 2H), 7.20 – 7.06 (m, 2H), 2.92 (s, 3H), 2.71 (s, 3H), 2.49 (s, 3H). **^{13}C NMR** (125 MHz, $CDCl_3$): δ (ppm) 172.0, 165.4, 162.9, 149.9, 145.3, 137.6, 124.2, 116.2, 116.2, 116.0, 23.5, 15.3, 12.4. **^{19}F NMR** (377 MHz, $CDCl_3$): δ (ppm) -110.2 (tt, *J* = 8.2, 5.3 Hz). HR-MS (ESI): calc. for: $C_{13}H_{13}FN_4OH$: 261.1146, found.: 261.1149. **IR** (ATR): $\tilde{\nu}$ (cm^{-1}) 1733, 1380, 1366, 1346, 1286, 1227, 846, 664, 590, 514.

2.4 pH-Dependent stability of NAc-PAP-H

A stock solution of **NAc-PAP-H** (1 mM) in MeOH was prepared in dark at room temperature at pH 2 and 12 (HCl as acid and $NaOCH_3$ as a base). Both samples were stirred for 2 h and we observed full conversion to **NH-PAP-H** (detected on LC–MS; 95–5% water in CH_3CN, 0.1% formic acid) in both cases as shown in Figure S1.

We reperformed the experiment in MeCN with DBU (10^{-2} M), and we observed after 2 h full conversion to various products and **NH-PAP-H**.

Already on the standard LC–MS trace, one can already observe a small appearance of the decomposition (NMR showed no impurities), since the mobile phase contained 0.1% of formic acid.

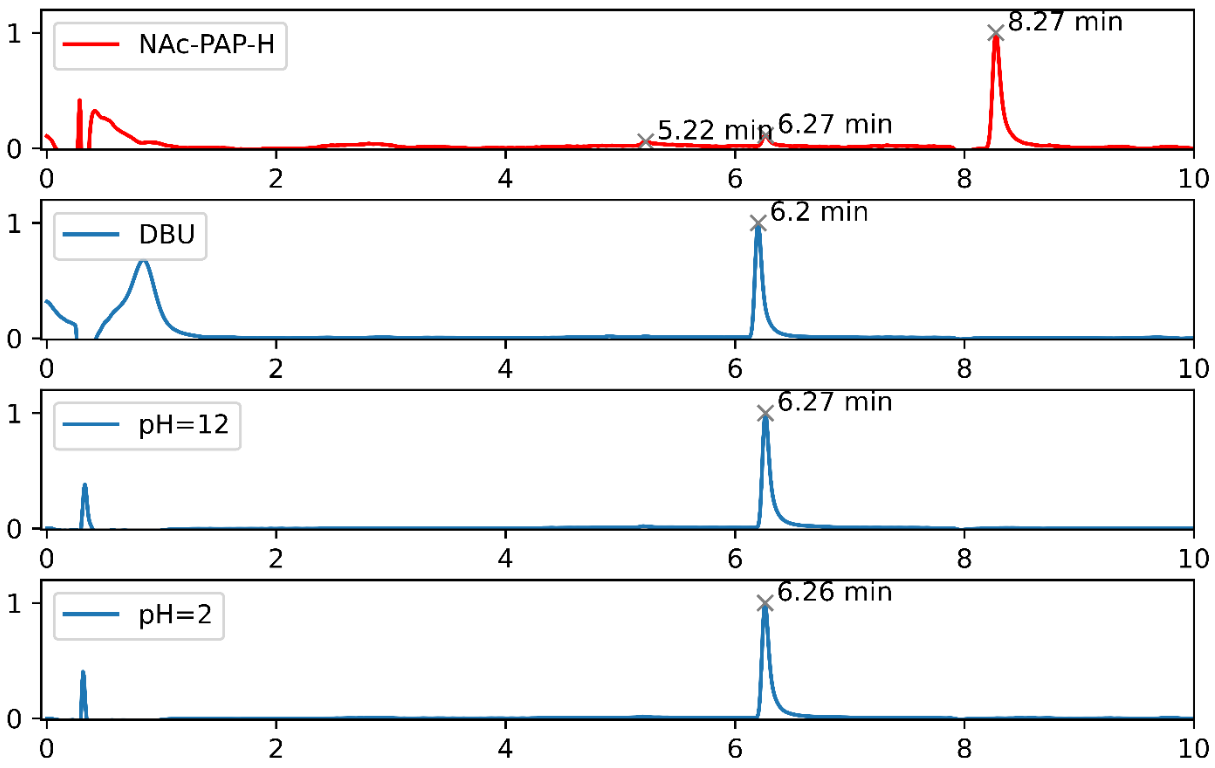

Figure S1: Chromatograph at 365 nm (maximum set to 1) of the pH stability experiments after 2 hours with the comparison of the starting material (**NAc PAP-H**).

3. Photochemical and photophysical studies

Upon 365 nm irradiation, the solution undergoes a color change from pale yellow to dark yellow, corresponding to an increased absorption of the n–π* transition associated with the *Z*-isomer, which can be observed by naked eye. The solution changes from pale to dark yellow.

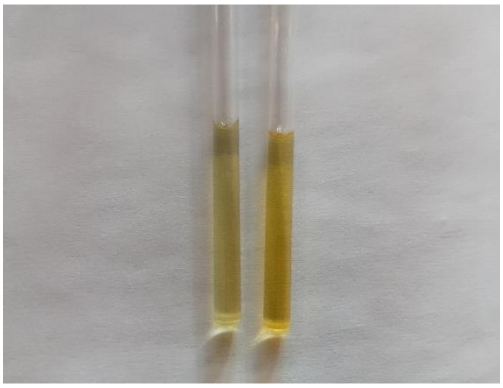

Figure S2: 0.5mM **NAc-PAP-H** on the right kept in the dark and on the left after 365 nm irradiation.

3.1 Determination of photostationary state distribution by ^{1}H NMR

The NMR samples of **NAc-PAPs** were irradiated with 365 or 445 nm LED for 10 min, and then immediately ^{1}H NMR spectra were recorded using a Bruker Advance III HD 300 at 25 °C. The PSS was determined by the integration of the *E* isomer and *Z* isomer signals.

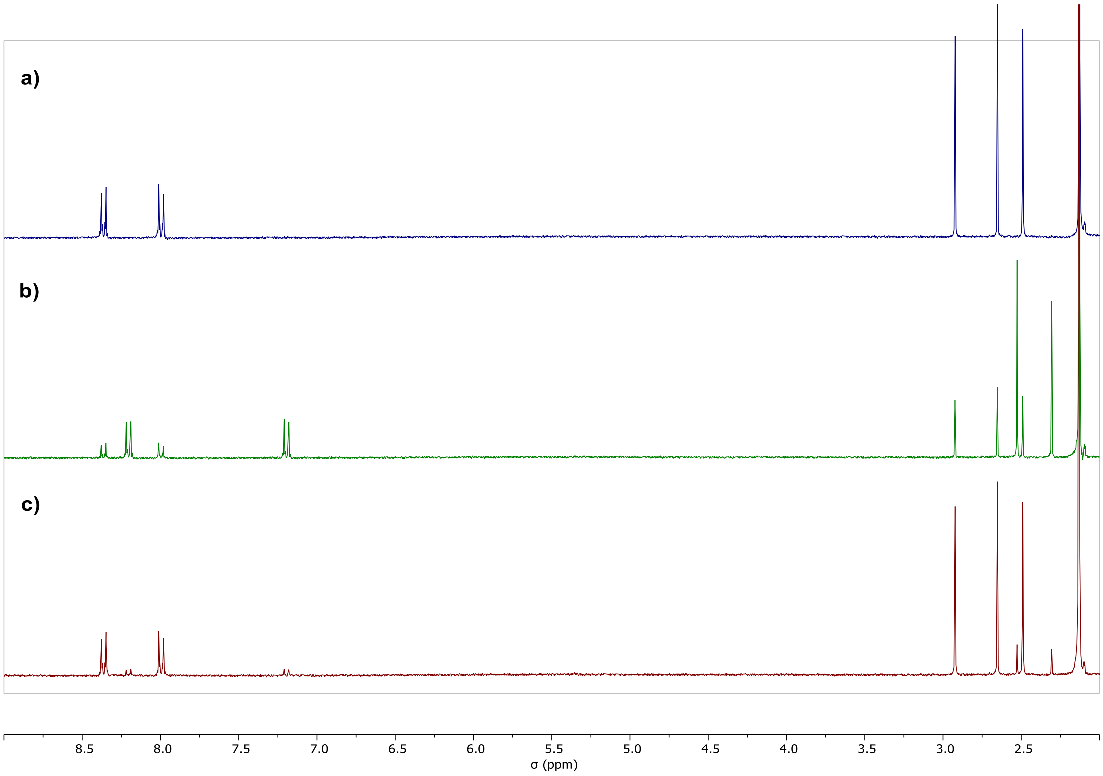

Figure S3: ^{1}H NMR spectra of **NAc-PAP-NO$_2$**- a) in dark b) irradiated by 365 nm c) irradiated with 445 nm in CD_3CN.

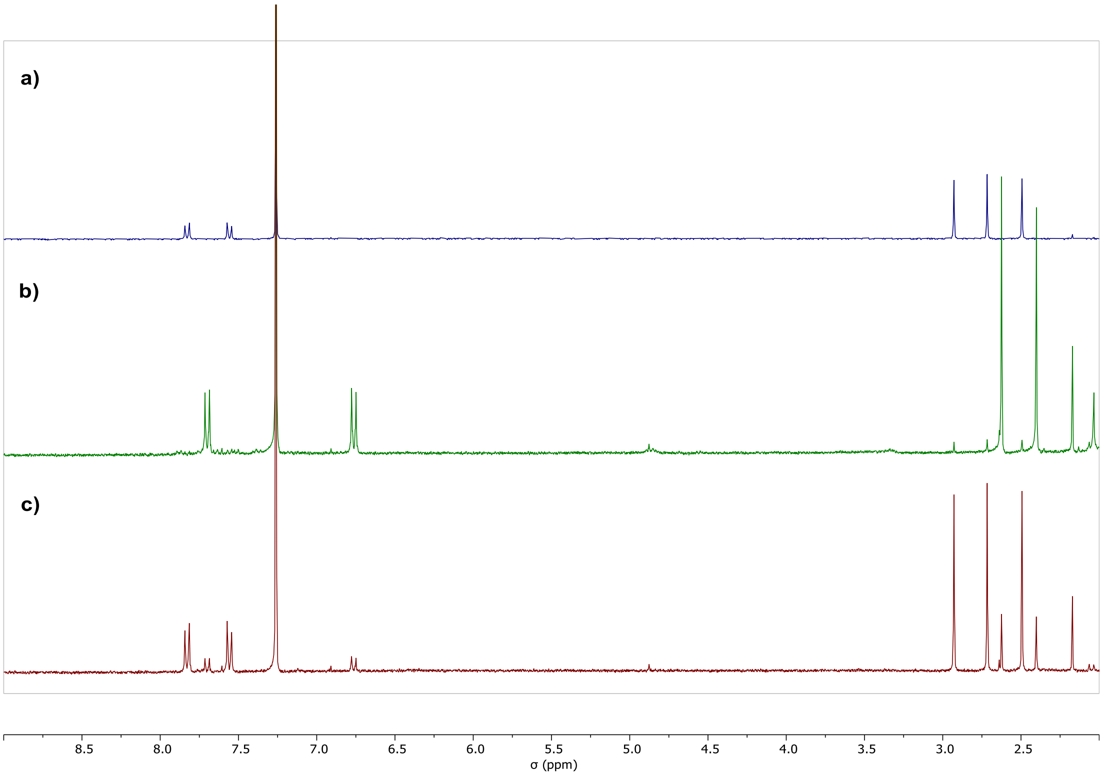

Figure S4: ^1H NMR spectra of **NAc-PAP-I**. a) in dark b) irradiated by 365 nm c) irradiated with 445 nm in $CDCl_3$.

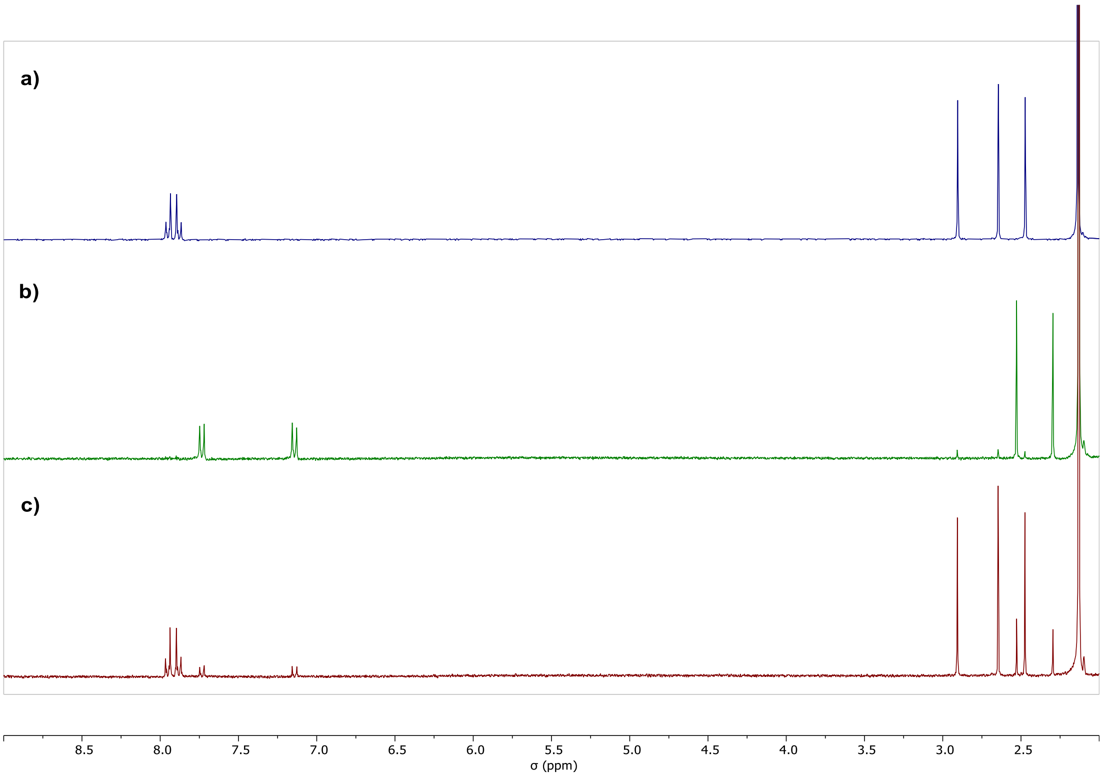

Figure S5: ^1H NMR spectra of **NAc-PAP-CN**. a) in dark b) irradiated by 365 nm c) irradiated with 445 nm in CD_3CN.

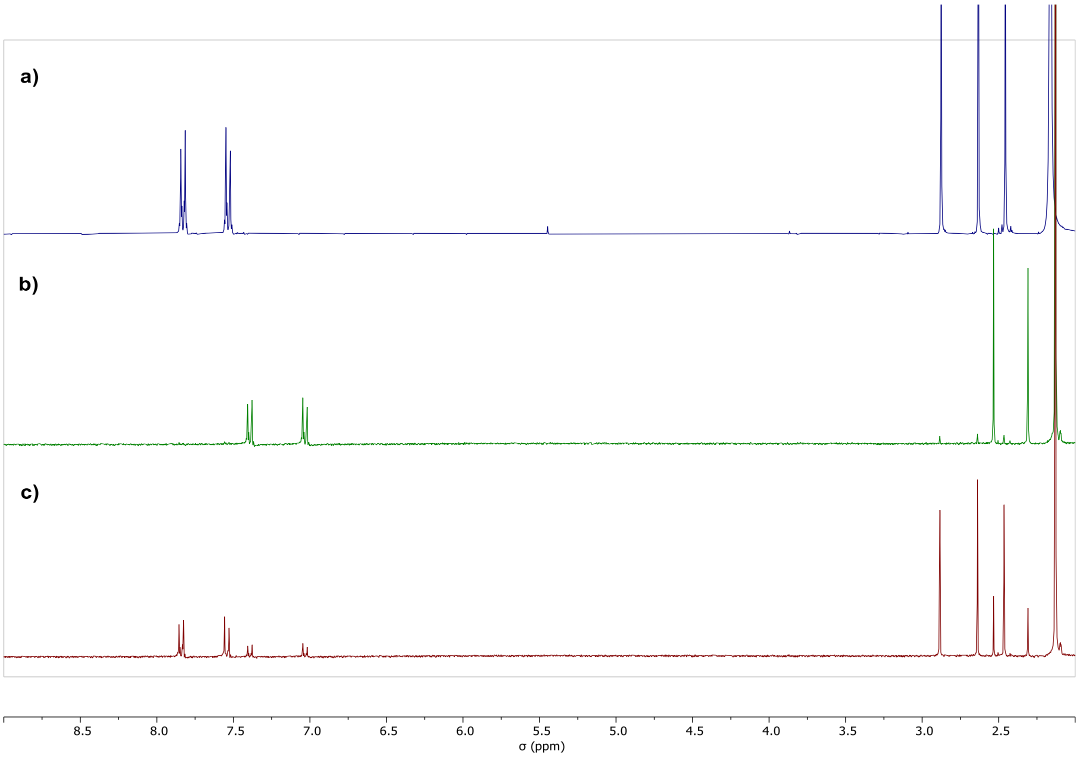

Figure S6: ^{1}H NMR spectra of **NAc-PAP-Cl**. a) in dark b) irradiated by 365 nm c) irradiated with 445 nm in CD_3CN.

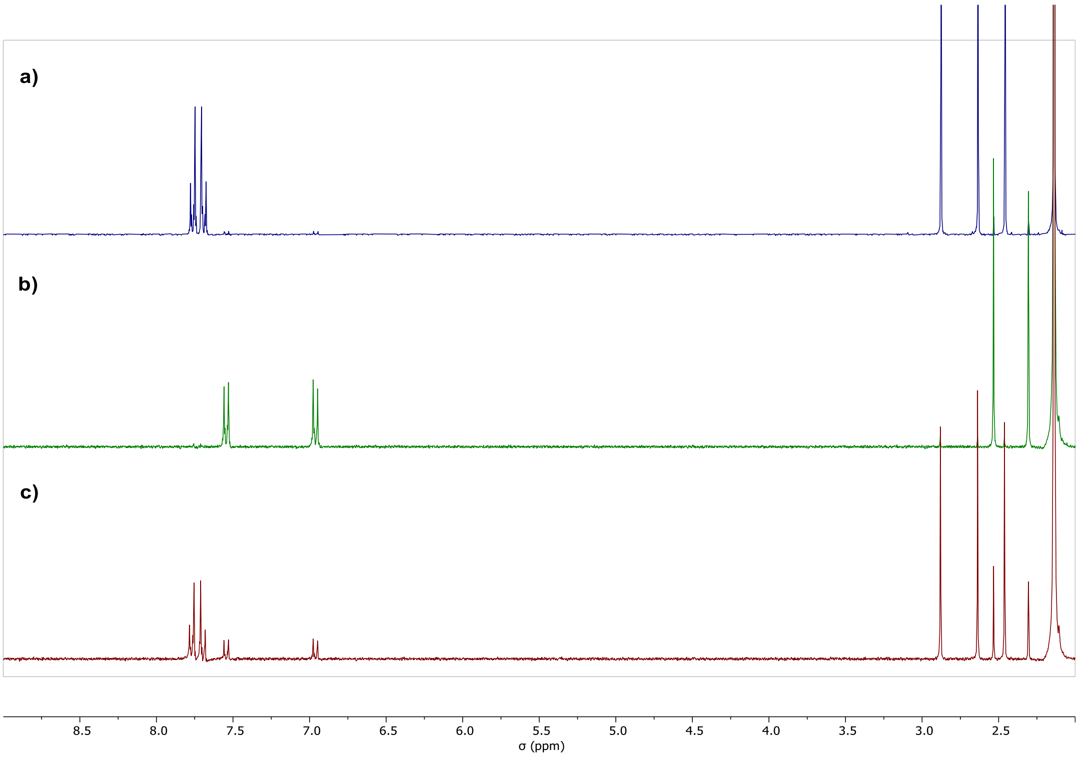

Figure S7: ^{1}H NMR spectra of **NAc-PAP-Br**. a) in dark b) irradiated by 365 nm c) irradiated with 445 nm in CD_3CN.

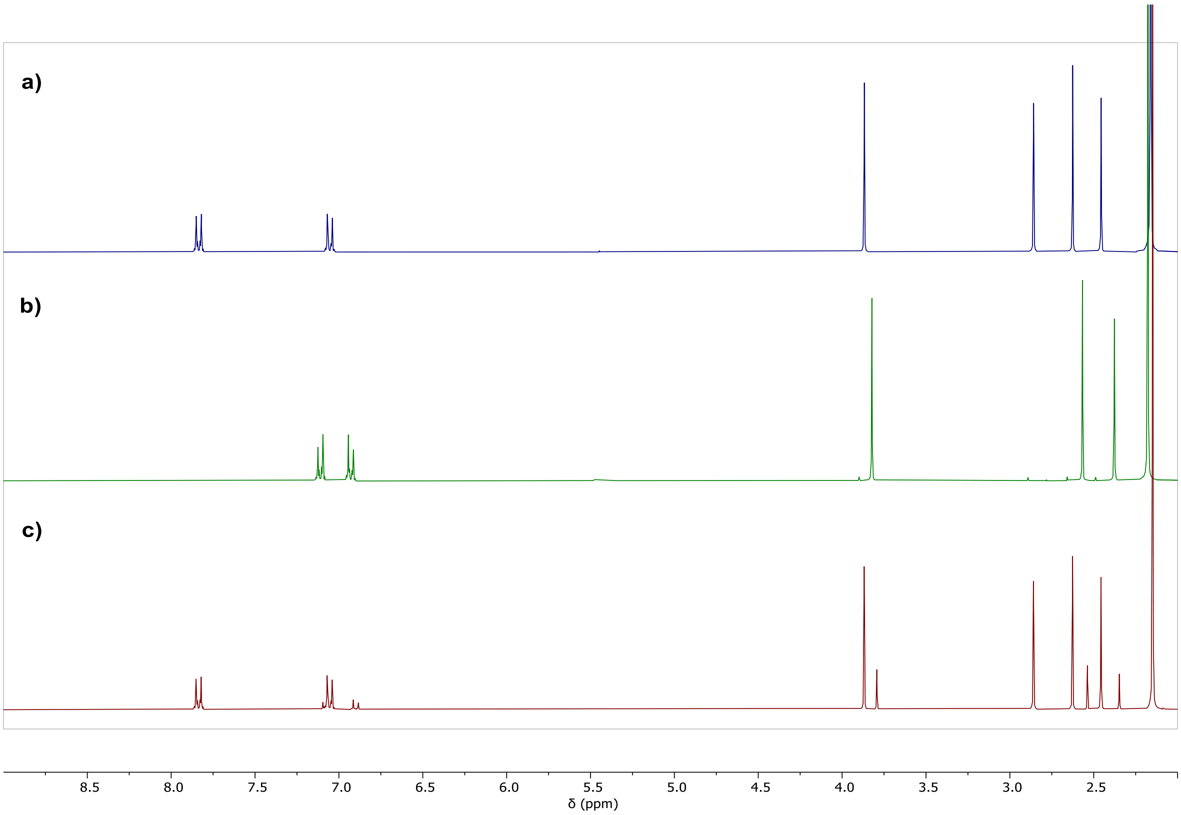

Figure S8: ^{1}H NMR spectra of **NAc-PAP-OMe**. a) in dark b) irradiated by 365 nm c) irradiated with 445 nm in CD_3CN.

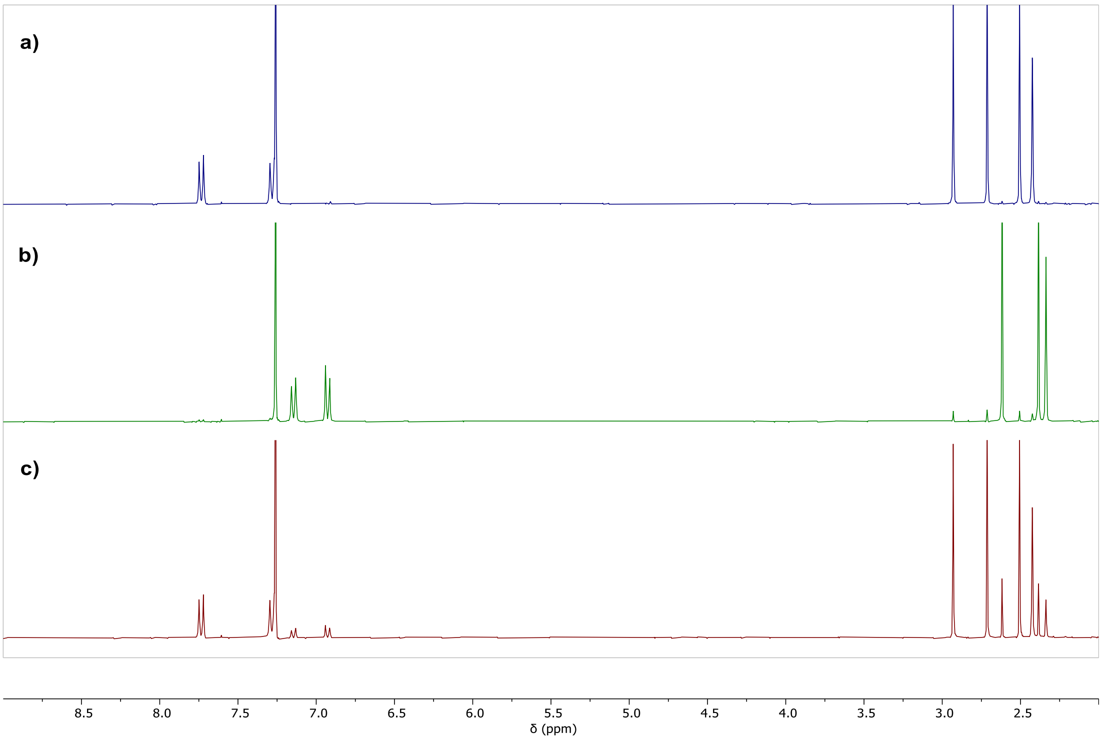

Figure S9: ^{1}H NMR spectra of **NAc-PAP-Me**. a) in dark b) irradiated by 365 nm c) irradiated with 445 nm in $CDCl_3$.

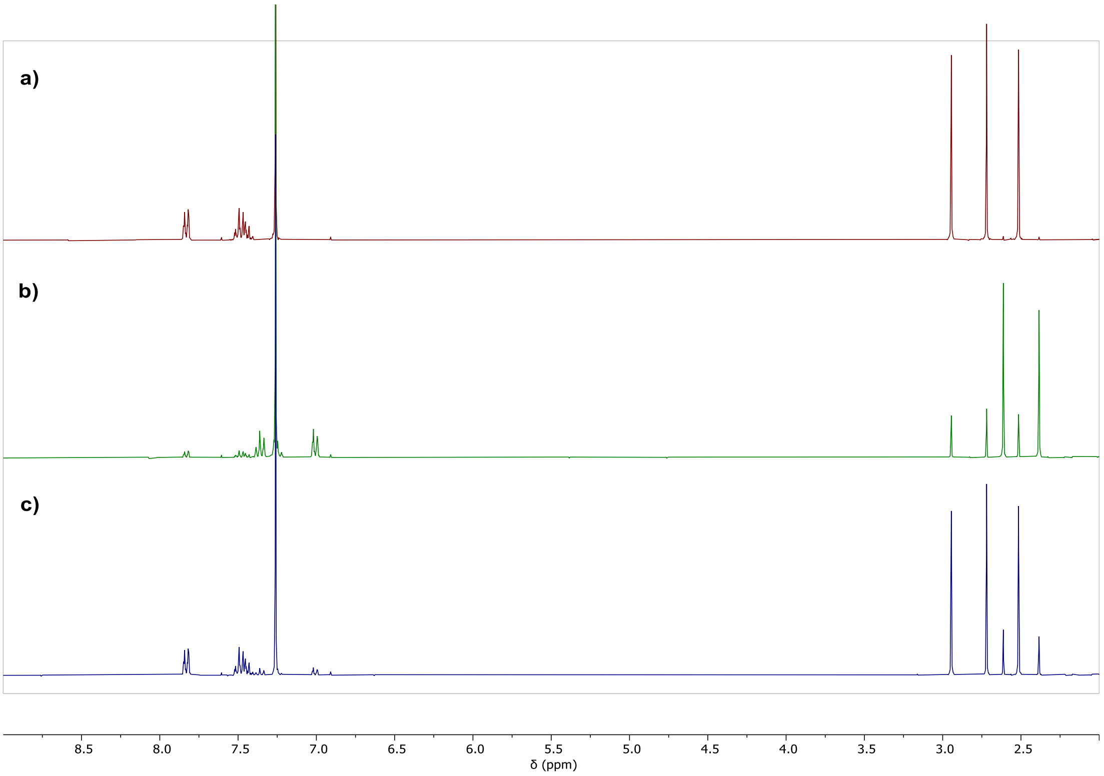

Figure S10: ^{1}H NMR spectra of **NAc-PAP-H**. a) in dark b) irradiated by 365 nm c) irradiated with 445 nm in $CDCl_3$.

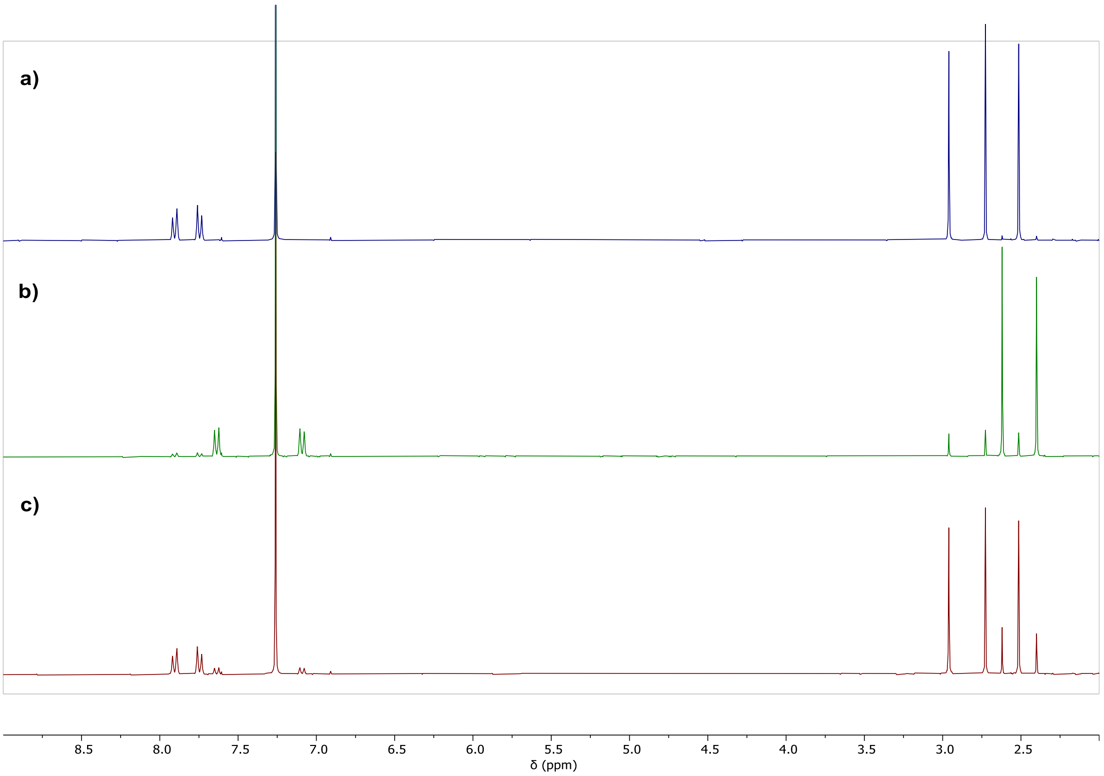

Figure S11: ^{1}H NMR spectra of **NAc-PAP-CF$_3$**. a) in dark b) irradiated by 365 nm c) irradiated with 445 nm in $CDCl_3$.

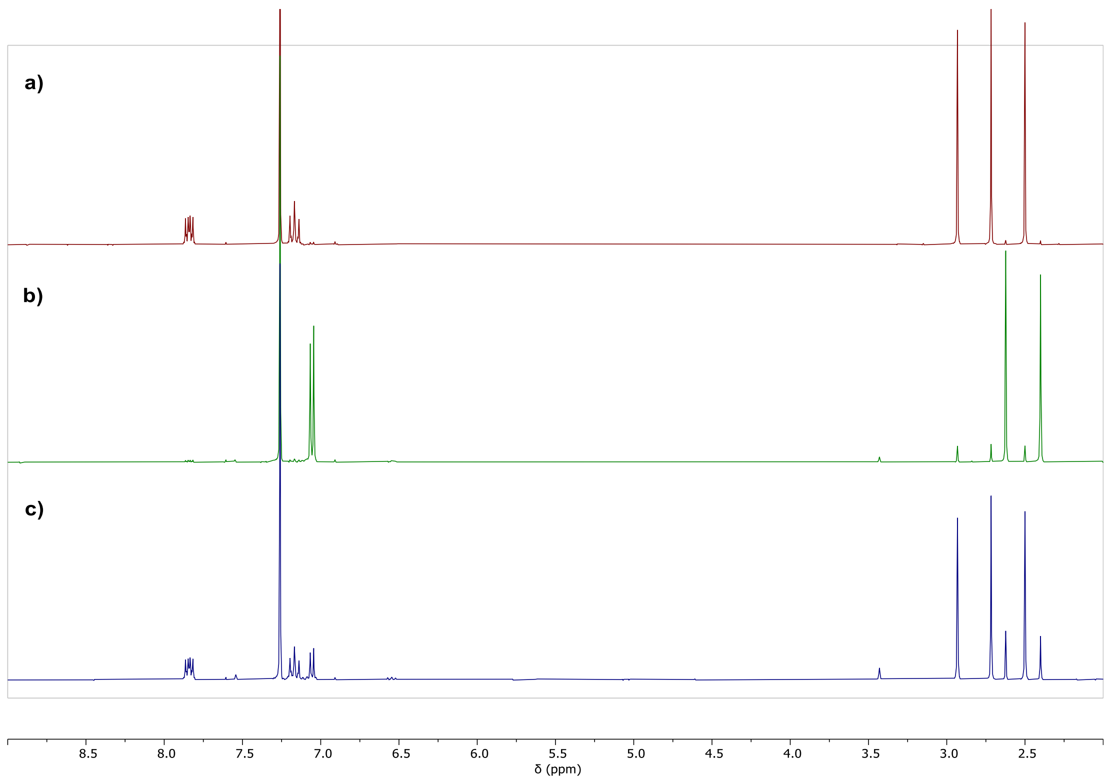

Figure S12: ^{1}H NMR spectra of **NAc-PAP-F**. a) in dark b) irradiated by 365 nm c) irradiated with 445 nm in $CDCl_3$.

3.2 Cyclic irradiation to study fatigue resistance

Photoswitching stability of **NAc-PAP** derivatives was investigated in CH_3CN. For the forward *E*→*Z*-isomerization step, 365 nm light was used until the PSS was reached, whereas for the *Z*→*E*-isomerization, 445 nm light was used until reaching PSS. Both the irradiation steps were repeated 10 to 20 times.

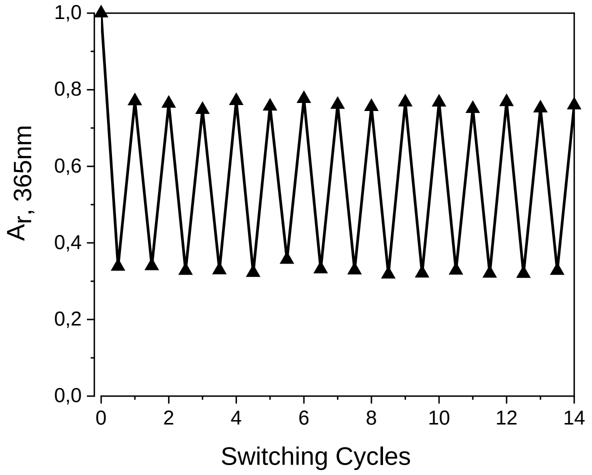

Figure S13: Photoswitching cycles of **NAc-PAP-NO$_2$** in CH_3CN at 25 °C.

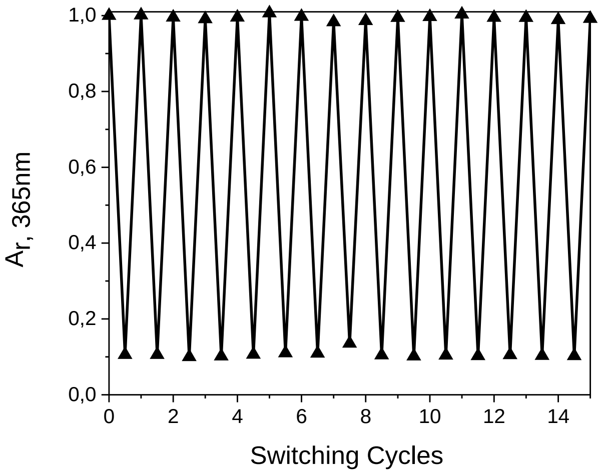

Figure S14: Photoswitching cycles of **NAc-PAP-I** in CH_3CN at 25 °C.

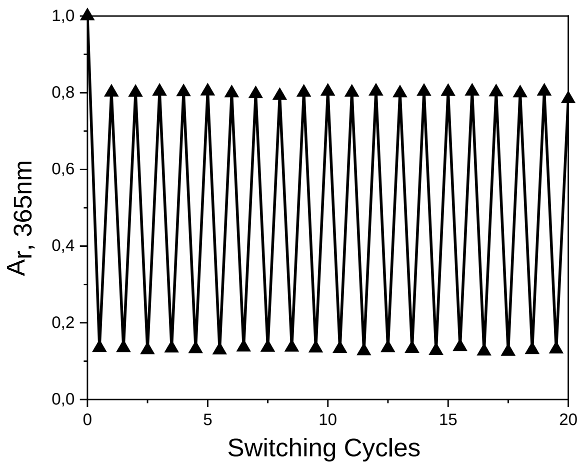

Figure S15: Photoswitching cycles of **NAc-PAP-CN** in CH_3CN at 25 °C.

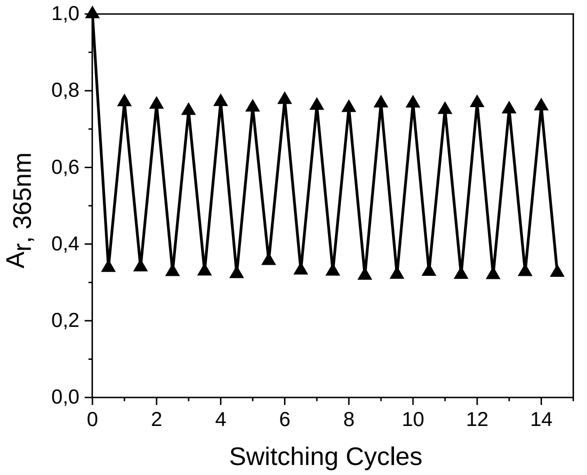

Figure S16: Photoswitching cycles of **NAc-PAP-OMe** in CH_3CN at 25 °C.

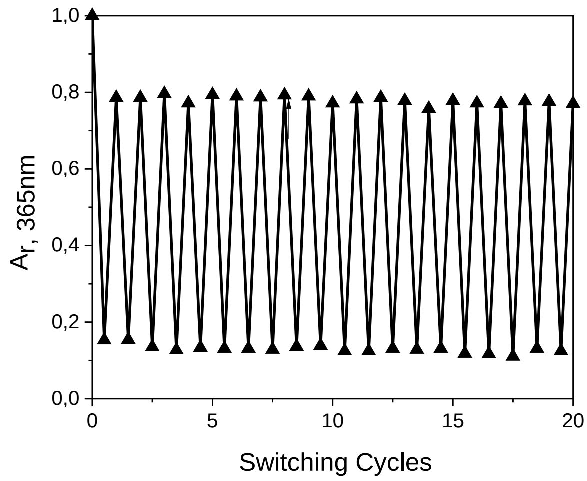

Figure S17: Photoswitching cycles of **NAc-PAP-Cl** in CH_3CN at 25 °C.

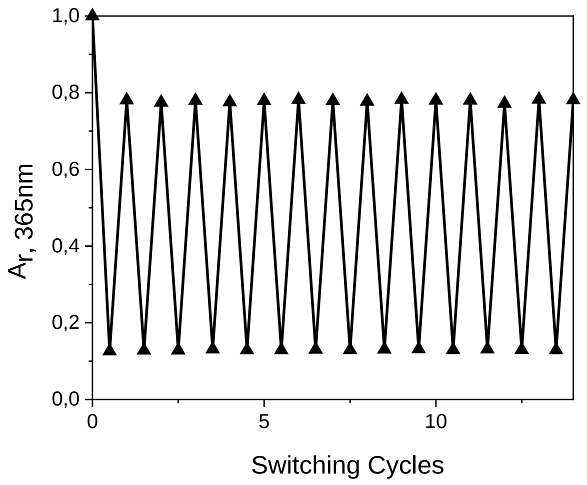

Figure S18: Photoswitching cycles of **NAc-PAP-Br** in CH_3CN at 25 °C.

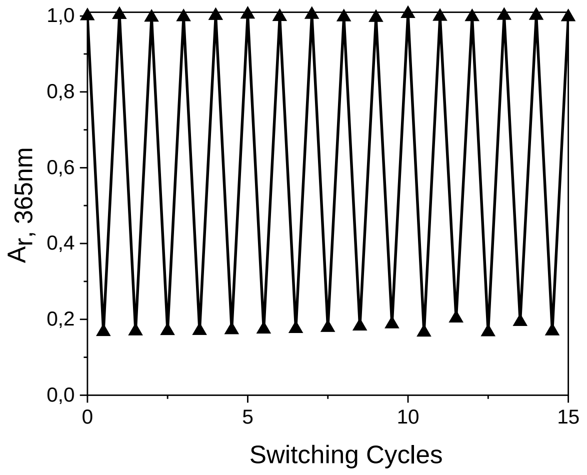

Figure S19: Photoswitching cycles of **NAc-PAP-CF$_3$** in CH_3CN at 25 °C.

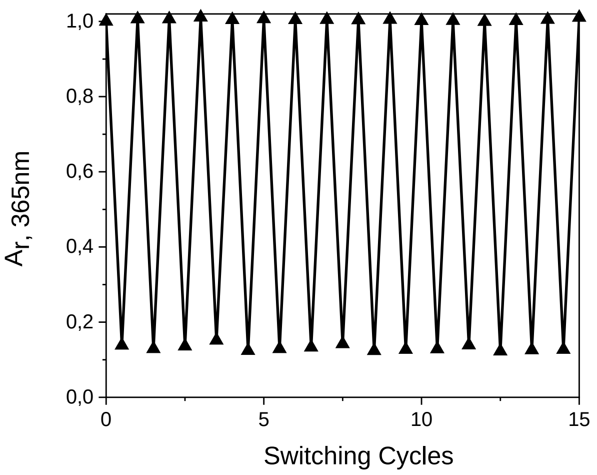

Figure S20: Photoswitching cycles of **NAc-PAP-F** in CH_3CN at 25 °C.

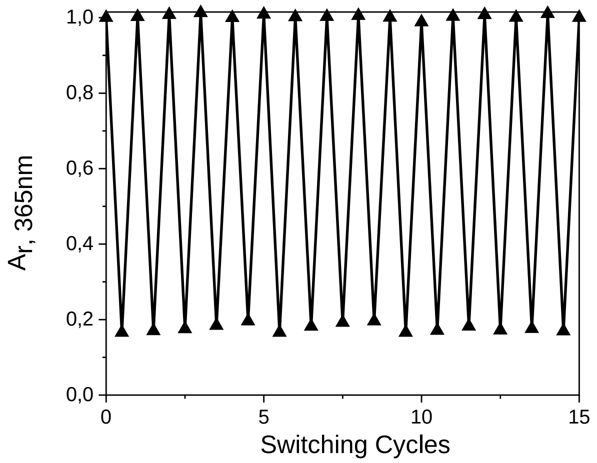

Figure S21: Photoswitching cycles of **NAc-PAP-H** in CH_3CN at 25 °C.

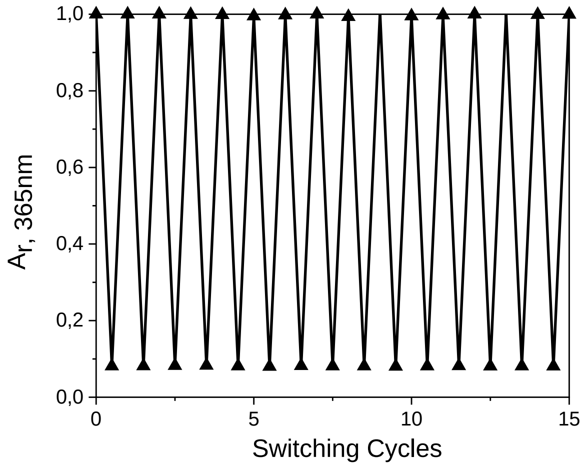

Figure S22: Photoswitching cycles of **NAc-PAP-Me** in CH_3CN at 25 °C.

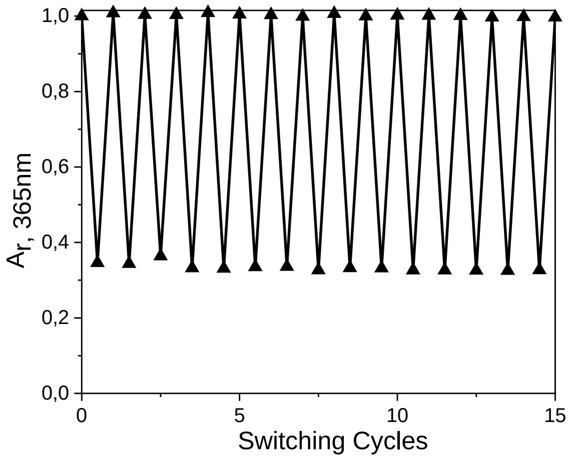

Figure S23: Photoswitching cycles of **NAc-PAP-OH** in CH_3CN at 25 °C.

3.3 Determination of molar extinction coefficients

Molar extinction coefficients (ε) were determined by fitting the slope of absorbance dependency to the concentration taken from at least two separate dilutions at 12.5 µM, 25 µM, 50 µM or 100 µM in CH_3CN. The values were fitted linearly and the y-intercept was set to zero.

3.3.1 Absorbance vs. concentrations of NAc-PAP derivatives

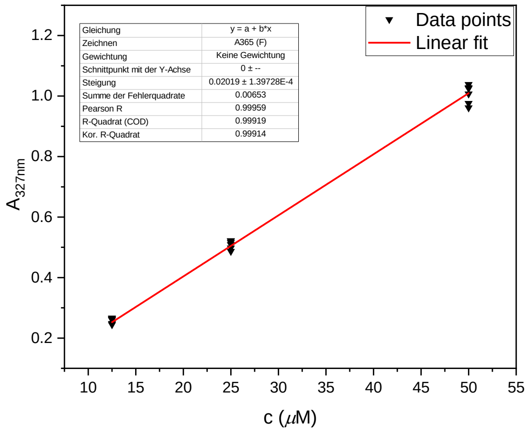

Figure S24: Absorbance at 327 nm dependency on concentration and the slopes of **NAc-PAP-F** in CH_3CN at 12.5, 25 and 50 µM.

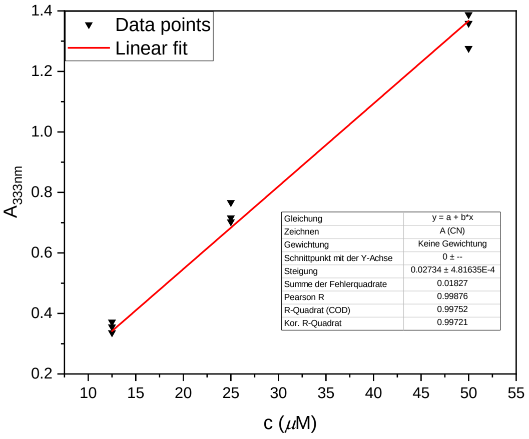

Figure S25: Absorbance at 333 nm dependency on concentration and the slopes of **NAc-PAP-CN** in CH_3CN at 12.5, 25 and 50 µM.

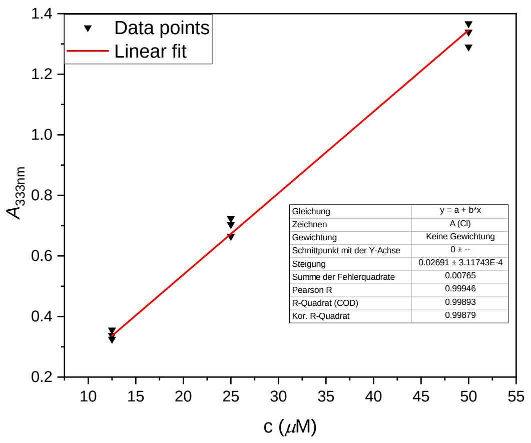

Figure S26: Absorbance at 333 nm dependency on concentration and the slopes of **NAc-PAP-Cl** in CH_3CN at 12.5, 25 and 50 μM.

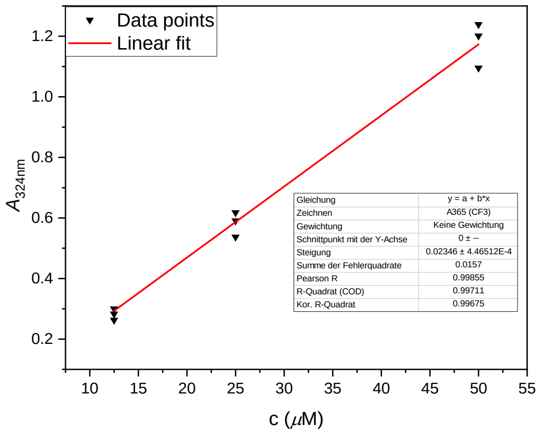

Figure S27: Absorbance at 324 nm dependency on concentration and the slopes of **NAc-PAP-CF$_3$** in CH_3CN at 12.5, 25 and 50 μM.

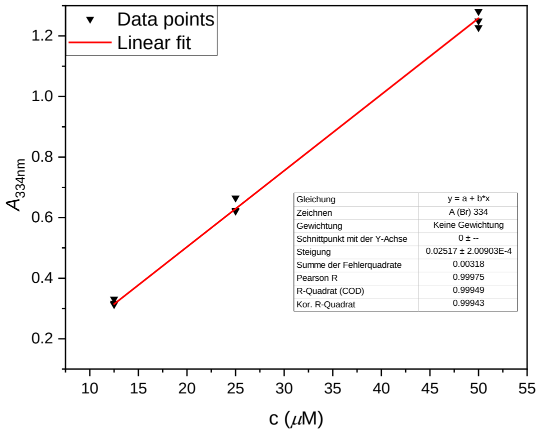

Figure S28: Absorbance at 344 nm dependency on concentration and the slopes of **NAc-PAP-Br** in CH_3CN at 12.5, 25 and 50 μM.

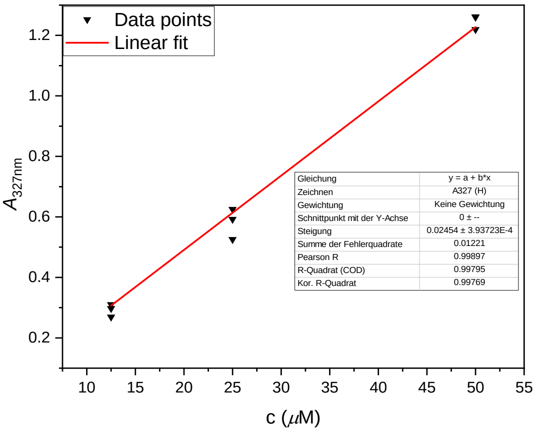

Figure S29: Absorbance at 327 nm dependency on concentration and the slopes of **NAc-PAP-H** in CH_3CN at 12.5, 25 and 50 µM.

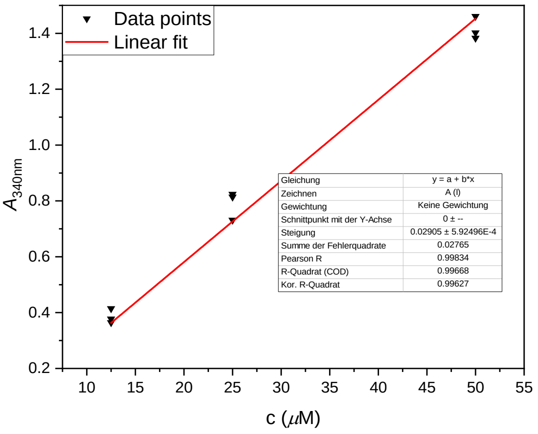

Figure S30: Absorbance at 340 nm dependency on concentration and the slopes of **NAc-PAP-I** in CH_3CN at 12.5, 25 and 50 µM.

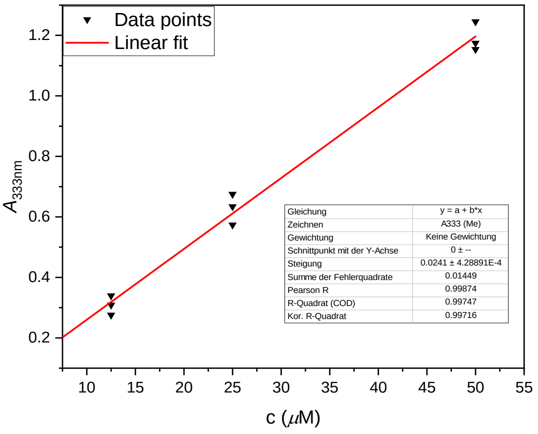

Figure S31: Absorbance at 333 nm dependency on concentration and the slopes of **NAc-PAP-Me** in CH_3CN at 12.5, 25 and 50 µM.

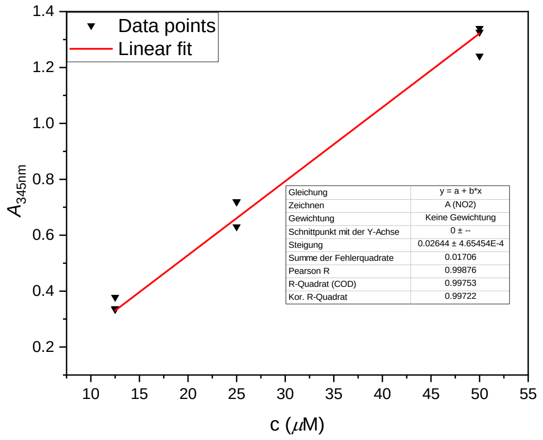

Figure S32: Absorbance at 345 nm dependency on concentration and the slopes of **NAc-PAP-NO₂** in CH_3CN at 12.5, 25 and 50 µM.

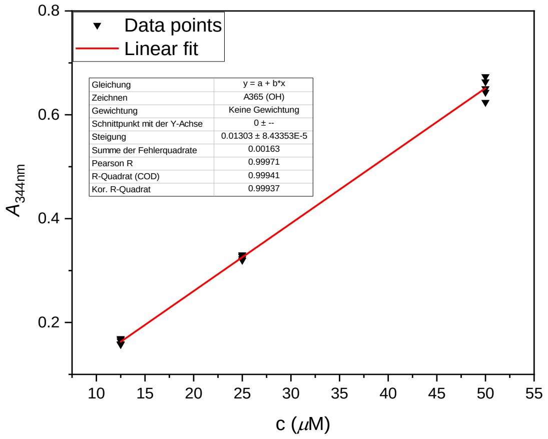

Figure S33: Absorbance at 344 nm dependency on concentration and the slopes of **NAc-PAP-OH** in CH_3CN at 12.5, 25 and 50 µM.

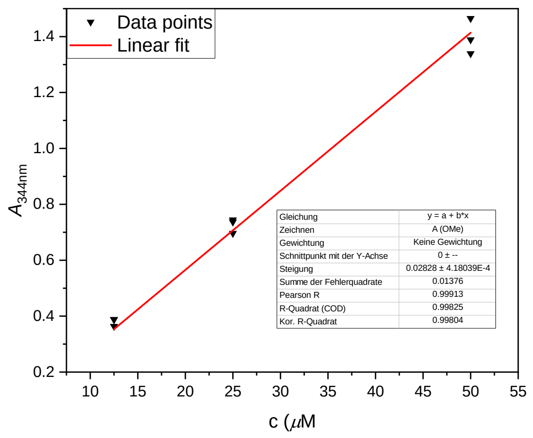

Figure S34: Absorbance at 344 nm dependency on concentration and the slopes of **NAc-PAP-OMe** in CH_3CN at 12.5, 25 and 50 µM.

3.3.2 Absorbance vs. concentrations of NMe-PAP derivatives

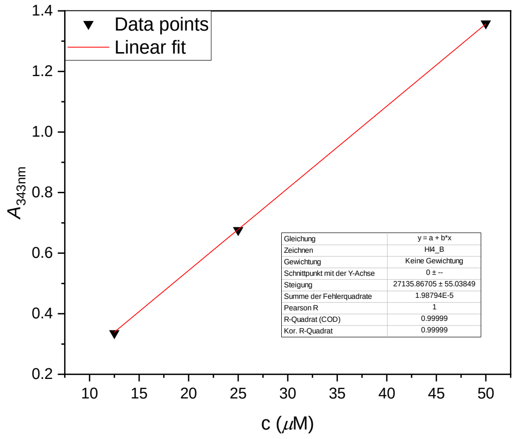

Figure S35: Absorbance at 343 nm dependency on concentration and the slopes of **NMe-PAP-Br** in CH_3CN at 12.5, 25 and 50 µM.

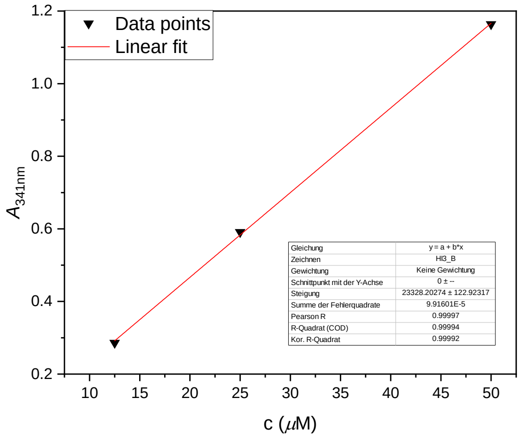

Figure S36: Absorbance at 341 nm dependency on concentration and the slopes of **NMe-PAP-CF_3** in CH_3CN at 12.5, 25 and 50 µM.

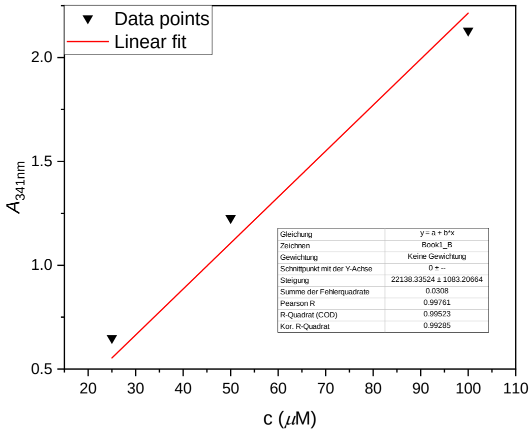

Figure S37: Absorbance at 341 nm dependency on concentration and the slopes of **NMe-PAP-Cl** in CH_3CN at 25, 50 and 100 µM.

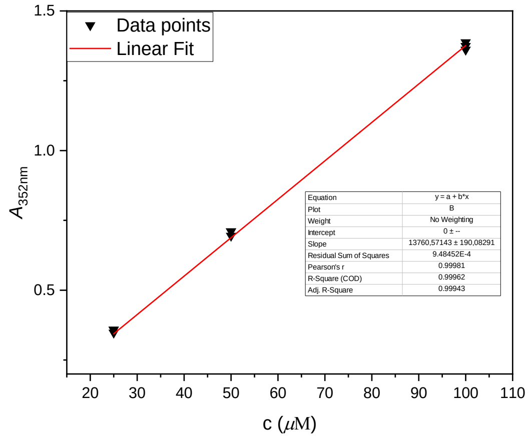

Figure S38: Absorbance at 352 nm dependency on concentration and the slopes of **NMe-PAP-CN** in CH_3CN at 25, 50 and 100 µM.

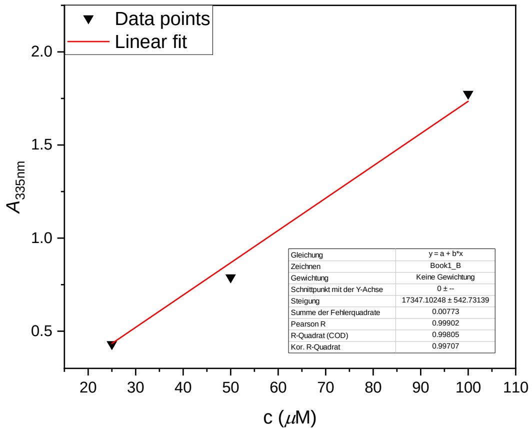

Figure S39: Absorbance at 335 nm dependency on concentration and the slopes of **NMe-PAP-F** in CH_3CN at 25, 50 and 100 µM.

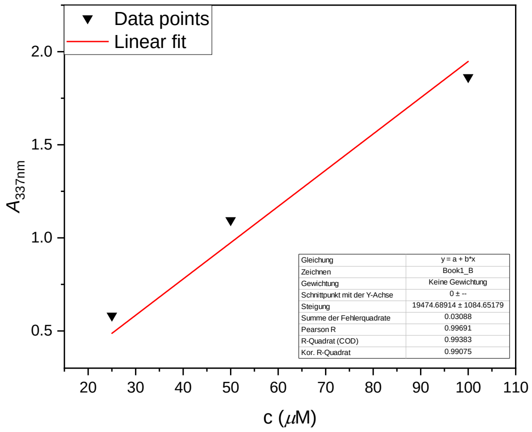

Figure S40: Absorbance at 337 nm dependency on concentration and the slopes of **NMe-PAP-H** in CH_3CN at 25, 50 and 100 µM.

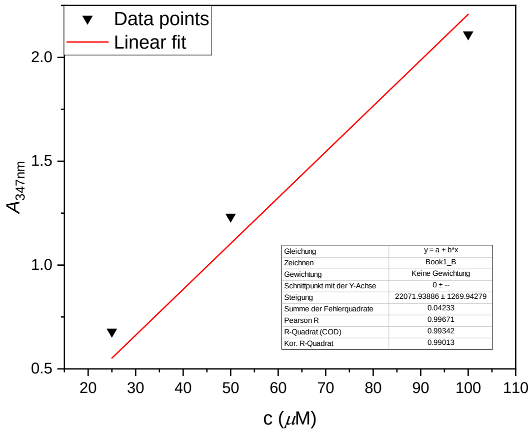

Figure S41: Absorbance at 347 nm dependency on concentration and the slopes of **NMe-PAP-I** in CH_3CN at 25, 50 and 100 µM.

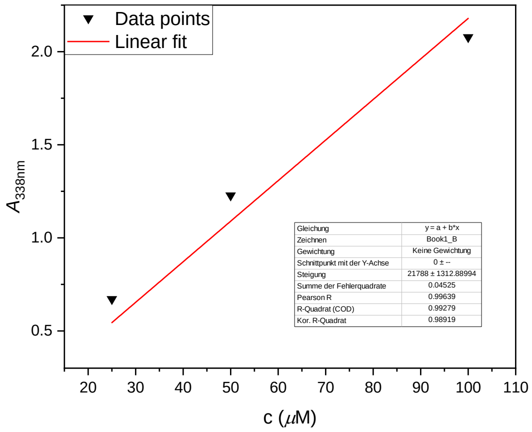

Figure S42: Absorbance at 338 nm dependency on concentration and the slopes of **NMe-PAP-Me** in CH_3CN at 12.5, 25 and 50 µM.

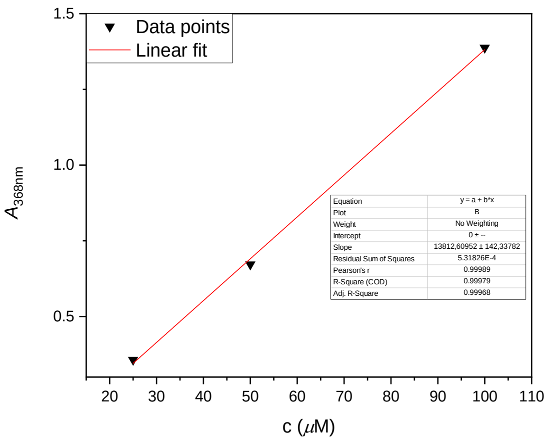

Figure S43: Absorbance at 368 nm dependency on concentration and the slopes of **NMe-PAP-NO$_2$** in CH_3CN at 25, 50 and 100 µM.

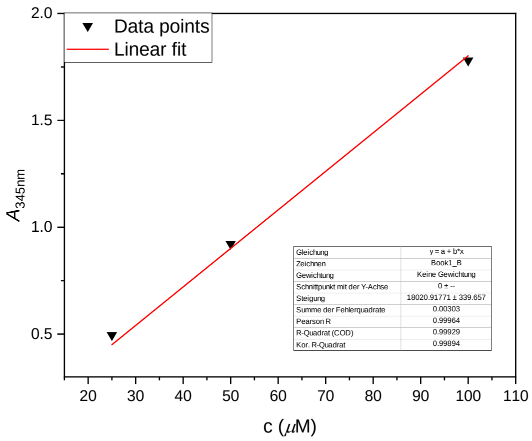

Figure S44: Absorbance at 345 nm dependency on concentration and the slopes of **NMe-PAP-OH** in CH_3CN at 12.5, 25 and 50 µM.

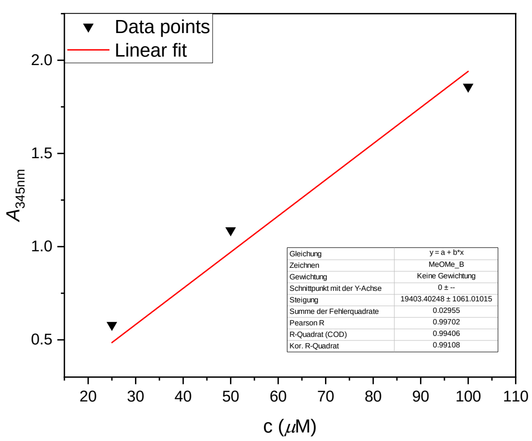

Figure S45: Absorbance at 345 nm dependency on concentration and the slopes of **NMe-PAP-OMe** in CH_3CN at 25, 50 and 100 µM.

3.3.3 Absorbance vs. concentrations of NH-PAP derivatives

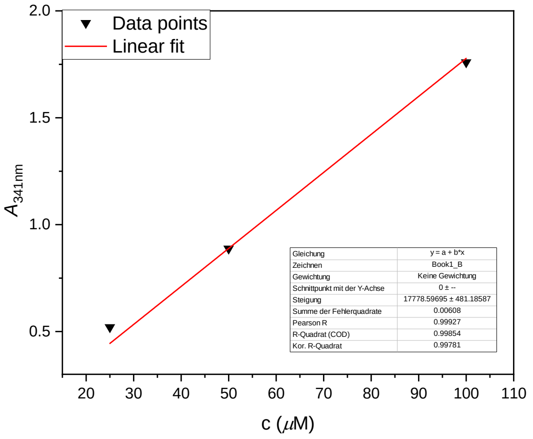

Figure S46: Absorbance at 341 nm dependency on concentration and the slopes of **NH-PAP-Br** in CH_3CN at 25, 50 and 100 μM.

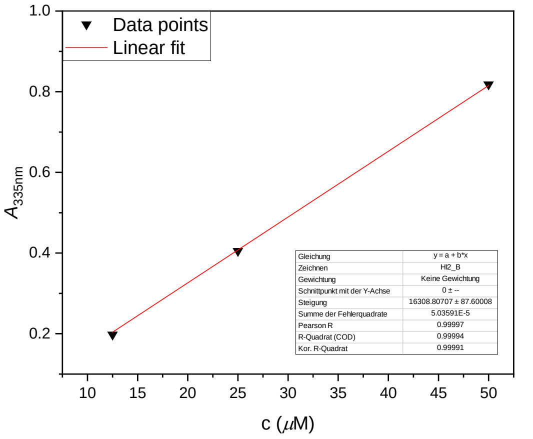

Figure S47: Absorbance at 335 nm dependency on concentration and the slopes of **NH-PAP-CF_3** in CH_3CN at 12.5, 25 and 50 μM.

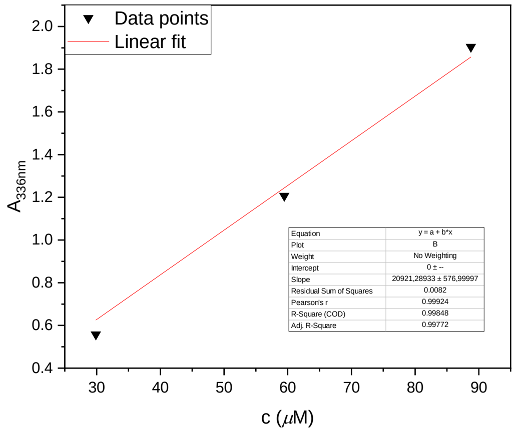

Figure S48: Absorbance at 336 nm dependency on concentration and the slopes of **NH-PAP-Cl** in CH_3CN at 30, 60 and 90 µM.

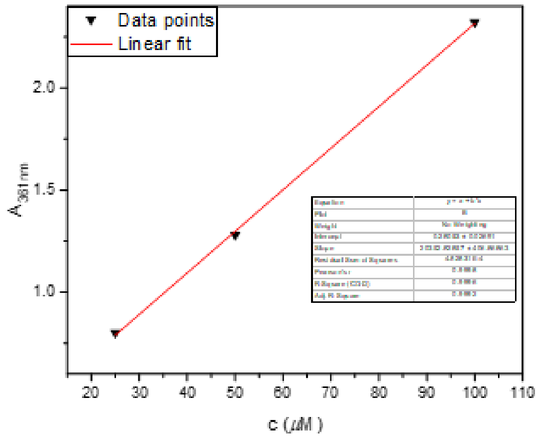

Figure S49: Absorbance at 361 nm dependency on concentration and the slopes of **NH-PAP-CN** in CH_3CN at 25, 50 and 100 µM.

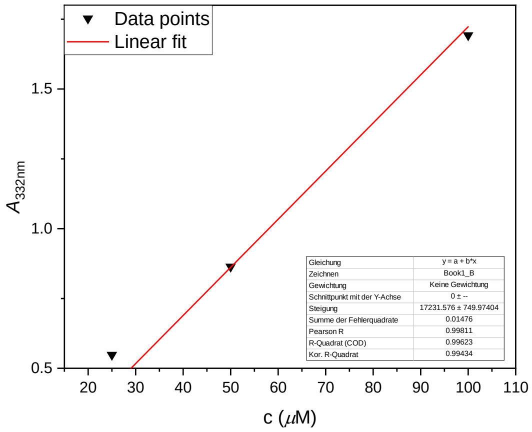

Figure S50: Absorbance at 332 nm dependency on concentration and the slopes of **NH-PAP-F** in CH_3CN at 25, 50 and 100 µM.

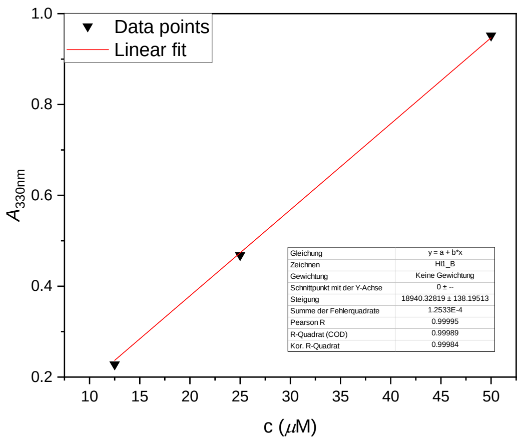

Figure S51: Absorbance at 330 nm dependency on concentration and the slopes of **NH-PAP-H** in CH_3CN at 12.5, 25 and 50 µM.

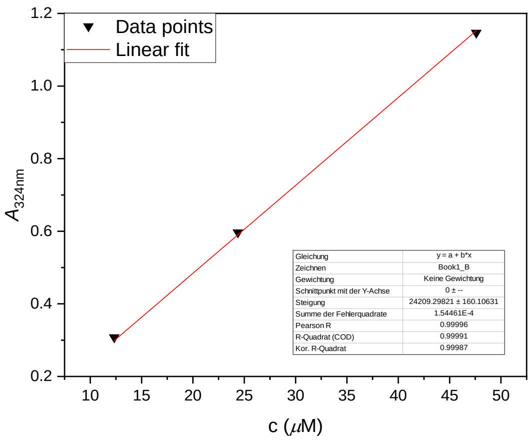

Figure S52: Absorbance at 324 nm dependency on concentration and the slopes of **NH-PAP-I** in CH_3CN at 12.5, 25 and 50 µM.

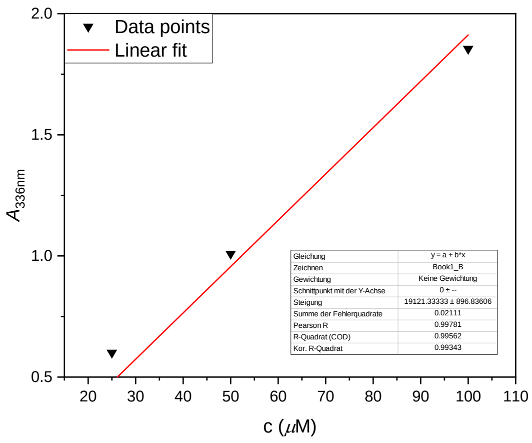

Figure S53: Absorbance at 336 nm dependency on concentration and the slopes of **NH-PAP-Me** in CH_3CN at 25, 50 and 100 µM.

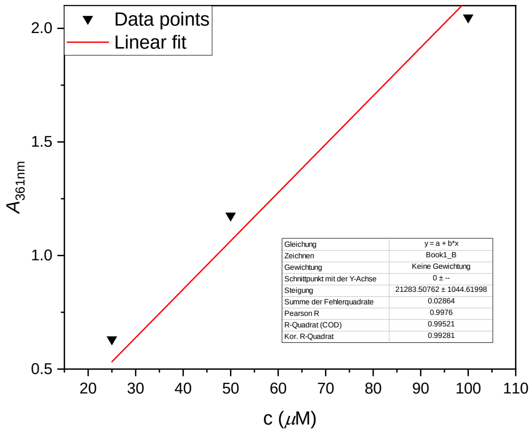

Figure S54: Absorbance at 361 nm dependency on concentration and the slopes of **NH-PAP-NO$_2$** in CH_3CN at 25, 50 and 100 µM.

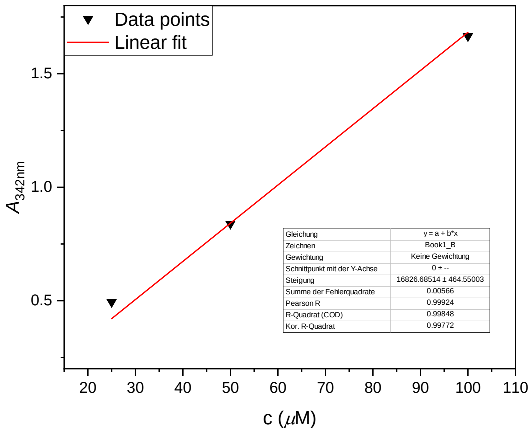

Figure S55: Absorbance at 342 nm dependency on concentration and the slopes of **NH-PAP-OH** in CH_3CN at 25, 50 and 100 µM.

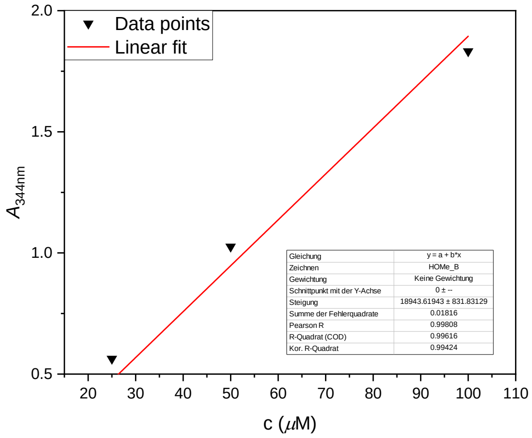

Figure S56: Absorbance at 344 nm dependency on concentration and the slopes of **NH-PAP-OMe** in CH_3CN at 25, 50 and 100 µM.

3.4 Chemical actinometry

A modification of a standard protocol was applied for the determination of the photon flux.[12] An aqueous H_2SO_4 solution (50 mM) containing freshly recrystallized $K_3[Fe(C_2O_4)_3]$ (41 mM, 2 mL, 1 cm quartz cuvette) was irradiated at 20 °C for a given period in the dark with a 365 nm then 445 nm LED. The solution was then diluted with 1.0 mL of an aqueous H_2SO_4 solution (0.5 M) containing phenanthroline (1 g/L) and NaOAc (122.5 g/L) and left to react for 10 min. The absorption at λ = 510 nm was measured and compared to an identically prepared non-irradiated sample. The concentration of $[Fe(phenanthroline)_3]^{2+}$ complex was calculated using its molar absorptivity (ε = 11 100 M^{-1} cm^{-1}) and considering the dilution. The quantity of Fe^{2+} ions expressed in mol was plotted versus time (expressed in seconds) and the slope, obtained by linear fitting the data points to the equation y = ax +b, equals the rate of formation of the Fe^{2+} ion at the given wavelength. This rate can be converted into the photon flux (I) by dividing it by the quantum yield of $[Fe(phenanthroline)_3]^{2+}$ complex (Φ^{365nm} = 1.29, Φ^{445nm} = 1.06) at 365 or 445 nm and by the probability of photon absorption at 365 nm of the Fe^{3+} complex (approximated to 1 as we were working in the total absorption regime). The obtained photon flux values for 365 nm and 445 nm are listed in Table S1.

Table S1: Determined photon flux values for 365 nm and 445 nm.

λ / nm	I / 10^{-5} mE s^{-1}.
365	2.38
445	6.43

3.5 Determination of quantum yields

The quantum yield of the photochemical isomerization of **PAP** compounds is determined using the initial slope method. Photoisomerization was measured under 365 (at concentration 12.5 µM) or 445 nm (at concentration 50 µM) irradiation at 25% or 10% intensity, the natural logarithm of the absorbance was plotted as a function of time. By applying equation 1,[12] the quantum yield of the light-induced isomerization of **PAP** compounds can be calculated.

$$\Phi = \frac{-k[X]_{t_0}V}{I\,(1-10^{A(t_0,\lambda)})} \tag{1}$$

Where Φ is the quantum yield; $-k$ is the reaction rate; $[X]_{t_0}$ is the concentration of the **PAP** compounds in the dark state (e.g. >>99% trans-isomer), V is the volume, I is the photon flux and $A(t_0, \lambda)$ is the absorption value before irradiation at 365 nm or 445 nm respectively. By applying first-order kinetics, $-k$ can be derived from the slope of the linear fit to the plot of the natural logarithm of absorbance as a function of time.

3.5.1 Irradiation of NAc-PAP derivatives and evaluation of the kinetic traces

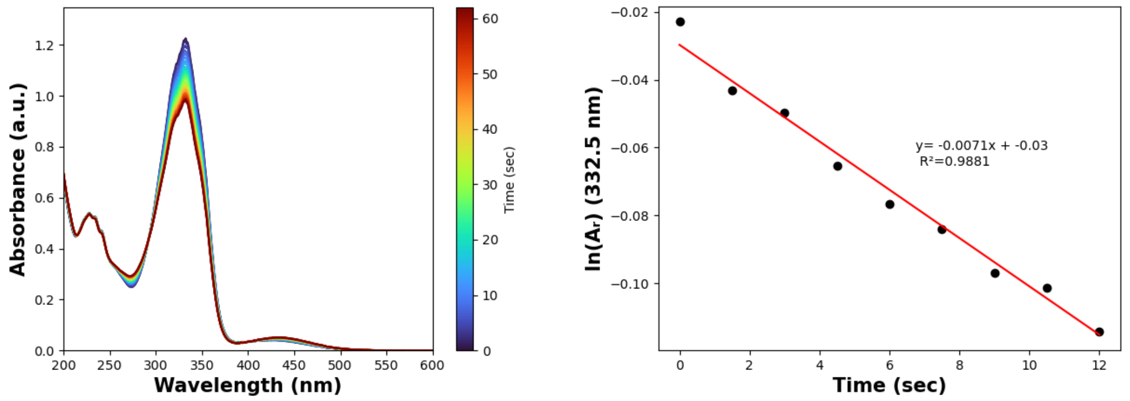

Figure S57: Left: Time-resolved UV–vis absorption spectra of **NAc-PAP-Me** (50 µM in CH_3CN) upon 445 nm irradiation. Right: Linear fit of the logarithmic kinetic trace of the change of absorbance at the absorption maximum of **NAc-PAP-Me**.

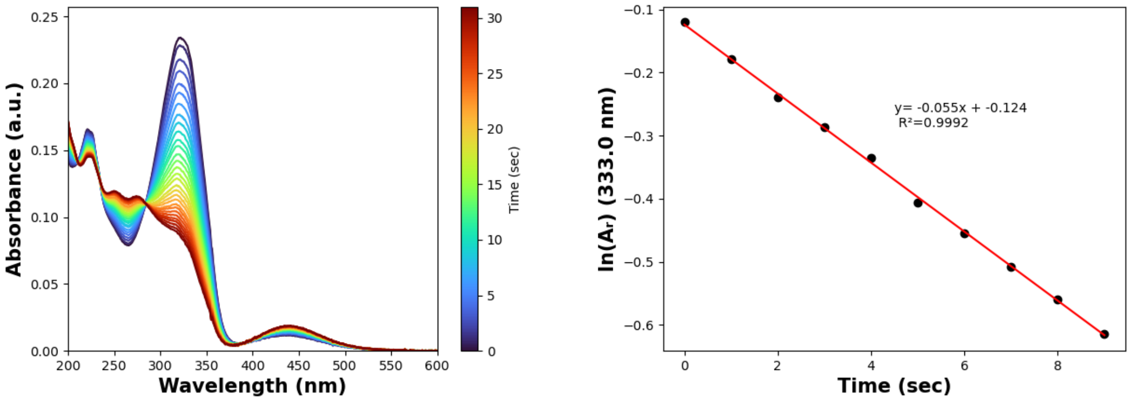

Figure S58: Left: Time-resolved UV–vis absorption spectra of **NAc-PAP-Me** (12.5 µM in CH_3CN) upon 365 nm irradiation. Right: Linear fit of the logarithmic kinetic trace of the change of absorbance at the absorption maximum of **NAc-PAP-Me**.

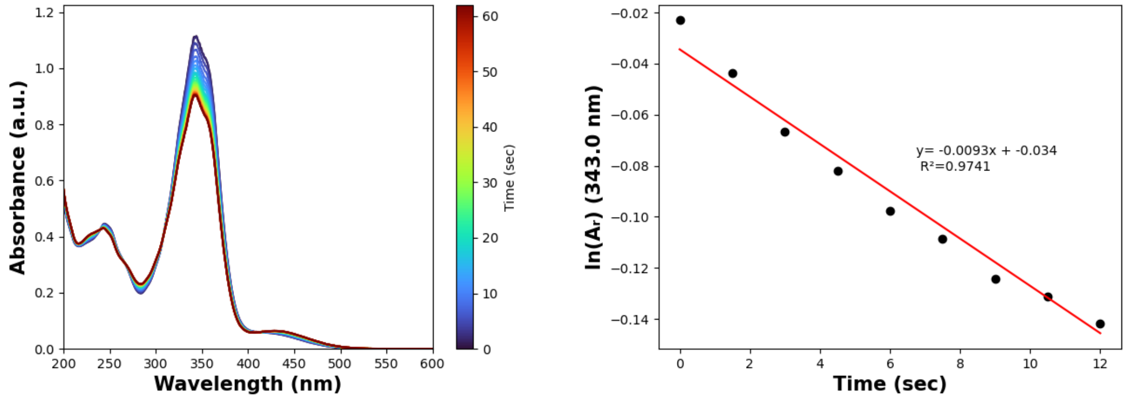

Figure S59: Left: Time-resolved UV–vis absorption spectra of **NAc-PAP-OMe** (37.5 µM in CH_3CN) upon 445 nm irradiation. Right: Linear fit of the logarithmic kinetic trace of the change of absorbance at the absorption maximum of **NAc-PAP-OMe**.

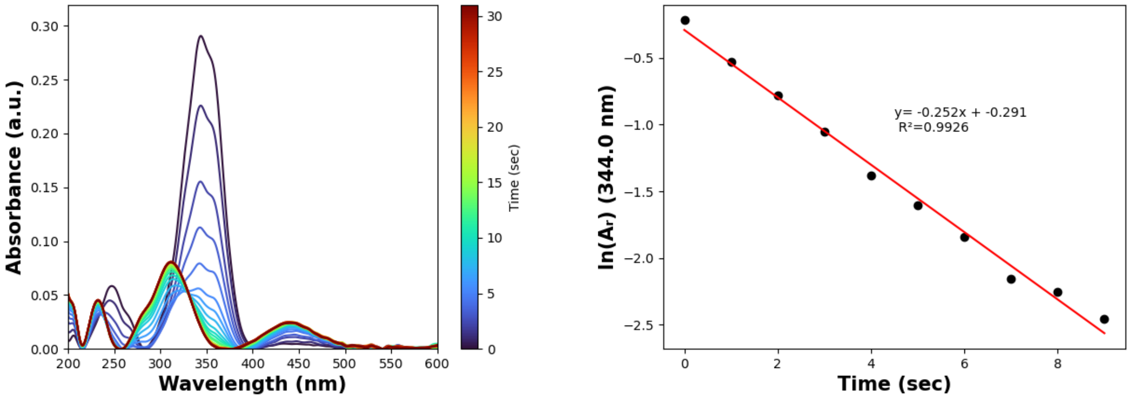

Figure S60: Time-resolved UV–vis absorption spectra of **NAc-PAP-OMe** (12.5 µM in CH_3CN) upon 365 nm irradiation. Right: Linear fit of the logarithmic kinetic trace of the change of absorbance at the absorption maximum of **NAc-PAP-OMe**.

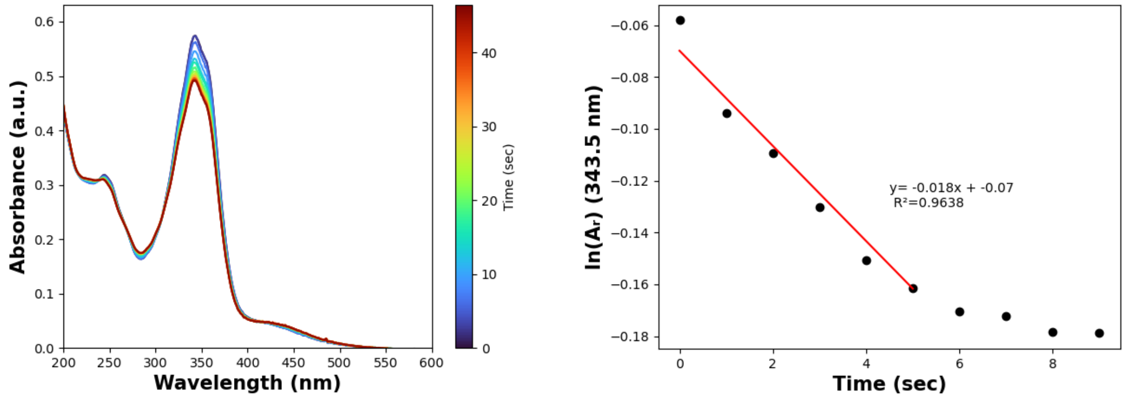

Figure S61: Left: Time-resolved UV–vis absorption spectra of **NAc-PAP-OH** (50 µM in CH_3CN) upon 445 nm irradiation. Right: Linear fit of the logarithmic kinetic trace of the change of absorbance at the absorption maximum of **NAc-PAP-OH**.

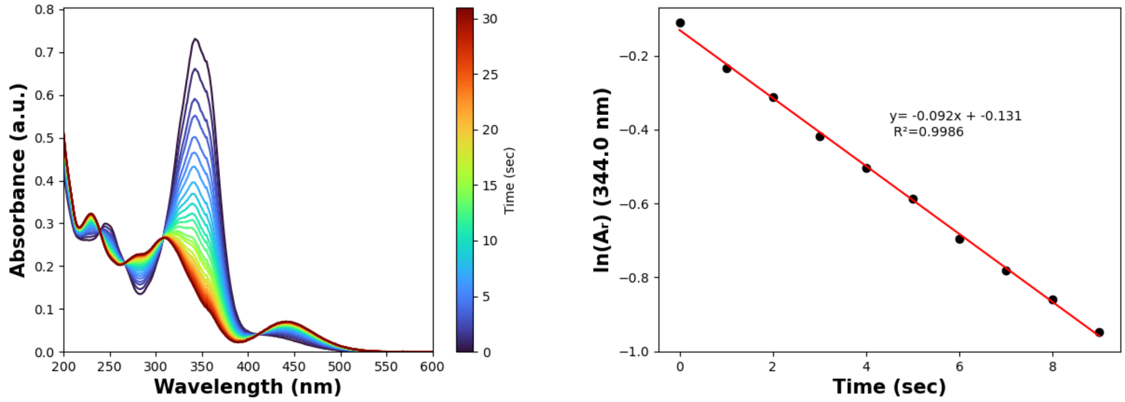

Figure S62: Left: Time-resolved UV–vis absorption spectra of **NAc-PAP-OH** (50 µM in CH_3CN) upon 365 nm irradiation. Right: Linear fit of the logarithmic kinetic trace of the change of absorbance at the absorption maximum of **NAc-PAP-OH**.

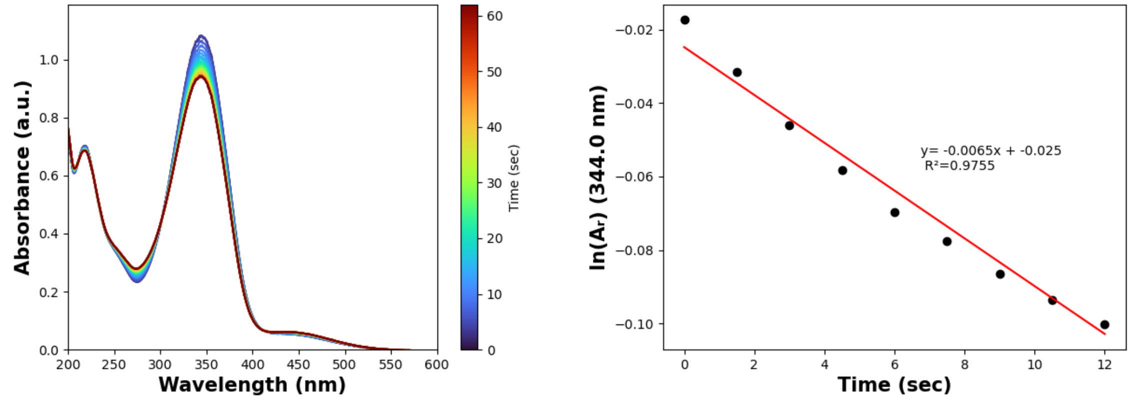

Figure S63: Left: Time-resolved UV–vis absorption spectra of **NAc-PAP-NO$_2$** (37.5 µM in CH_3CN) upon 445 nm irradiation. Right: Linear fit of the logarithmic kinetic trace of the change of absorbance at the absorption maximum of **NAc-PAP-NO$_2$**.

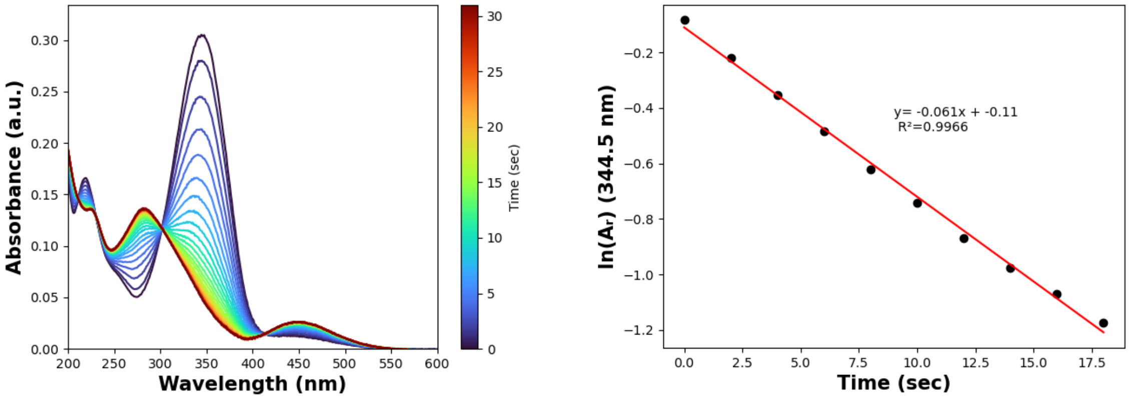

Figure S64: Left: Time-resolved UV–vis absorption spectra of **NAc-PAP-NO$_2$** (12.5 µM in CH_3CN) upon 365 nm irradiation. Right: Linear fit of the logarithmic kinetic trace of the change of absorbance at the absorption maximum of **NAc-PAP-NO$_2$**.

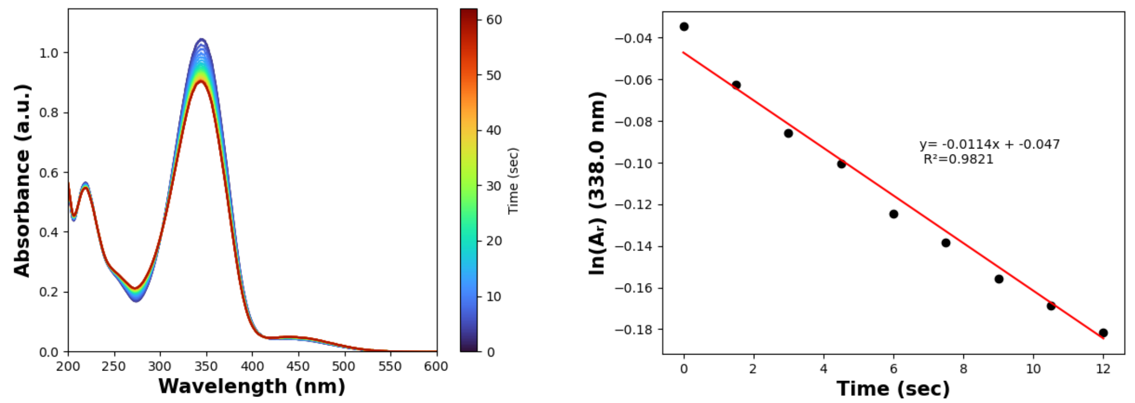

Figure S65: Left: Time-resolved UV–vis absorption spectra of **NAc-PAP-I** (37.5 µM in CH_3CN) upon 445 nm irradiation. Right: Linear fit of the logarithmic kinetic trace of the change of absorbance at the absorption maximum of **NAc-PAP-I**.

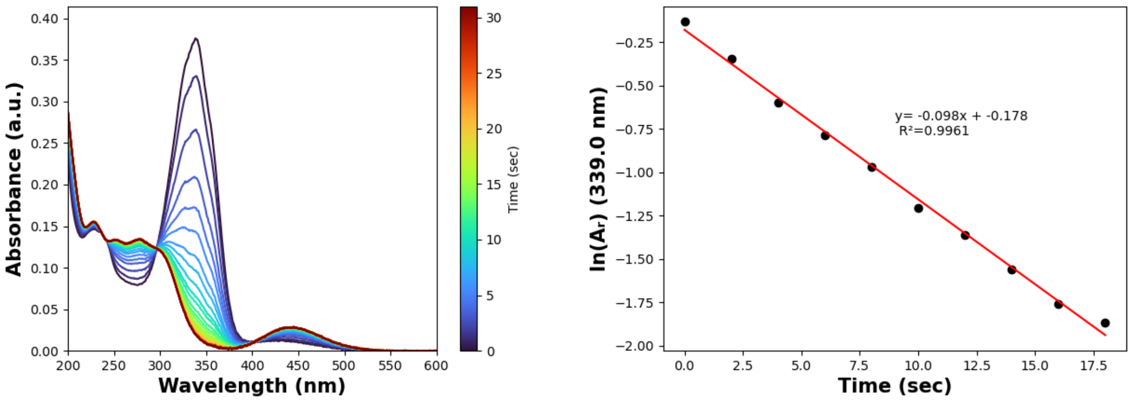

Figure S66: Left: Time-resolved UV–vis absorption spectra of **NAc-PAP-I** (50 µM in CH_3CN) upon 365 nm irradiation. Right: Linear fit of the logarithmic kinetic trace of the change of absorbance at the absorption maximum of **NAc-PAP-I**.

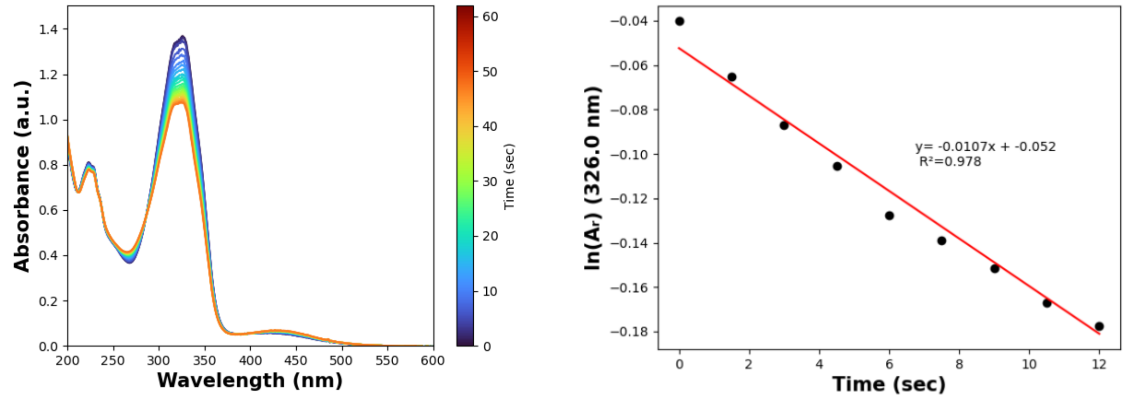

Figure S67: Left: Time-resolved UV–vis absorption spectra of **NAc-PAP-H** (50 µM in CH_3CN) upon 445 nm irradiation. Right: Linear fit of the logarithmic kinetic trace of the change of absorbance at the absorption maximum of **NAc-PAP-H**.

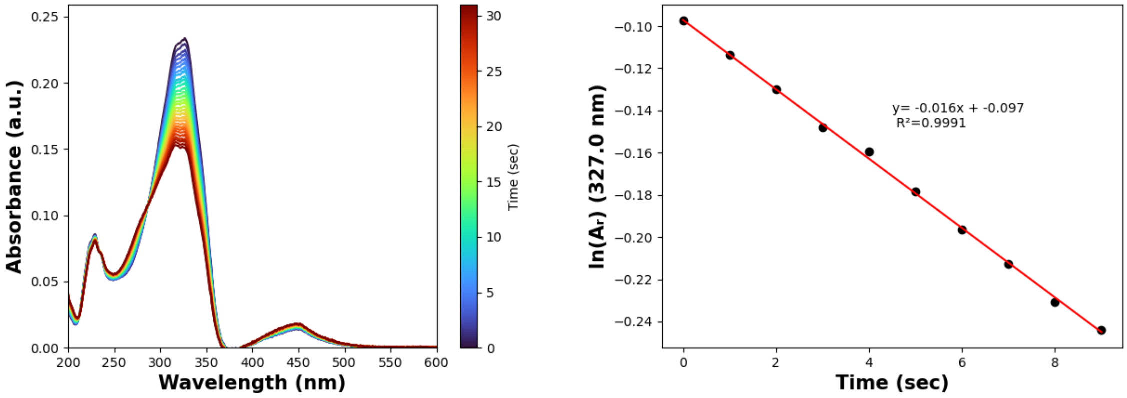

Figure S68: Left: Time-resolved UV–vis absorption spectra of **NAc-PAP-H** (12.5 µM in CH_3CN) upon 365 nm irradiation. Right: Linear fit of the logarithmic kinetic trace of the change of absorbance at the absorption maximum of **NAc-PAP-H**.

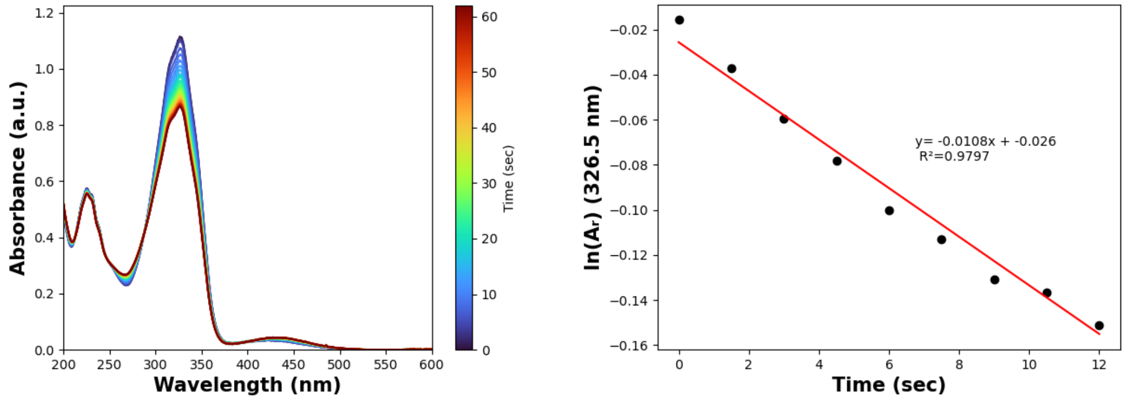

Figure S69: Left: Time-resolved UV–vis absorption spectra of **NAc-PAP-F** (50 µM in CH_3CN) upon 445 nm irradiation. Right: Linear fit of the logarithmic kinetic trace of the change of absorbance at the absorption maximum of **NAc-PAP-F**.

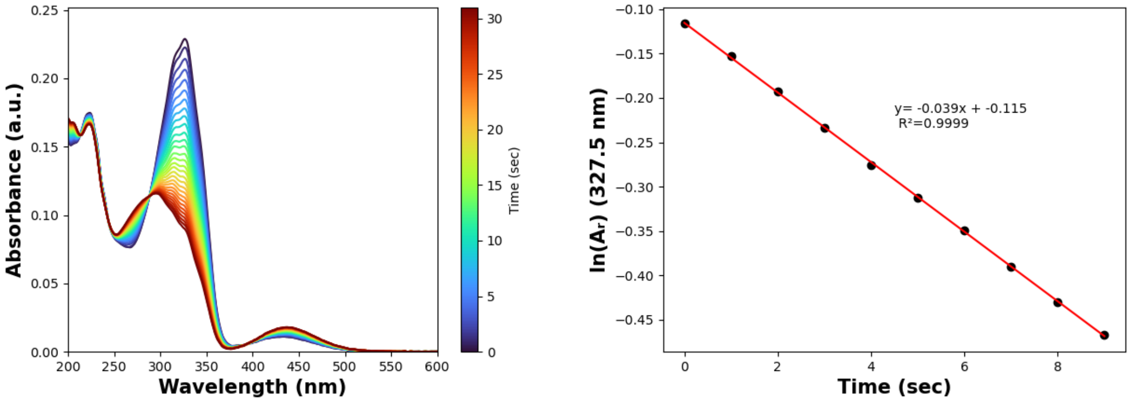

Figure S70: Left: Time-resolved UV–vis absorption spectra of **NAc-PAP-F** (12.5 µM in CH_3CN) upon 365 nm irradiation. Right: Linear fit of the logarithmic kinetic trace of the change of absorbance at the absorption maximum of **NAc-PAP-F**.

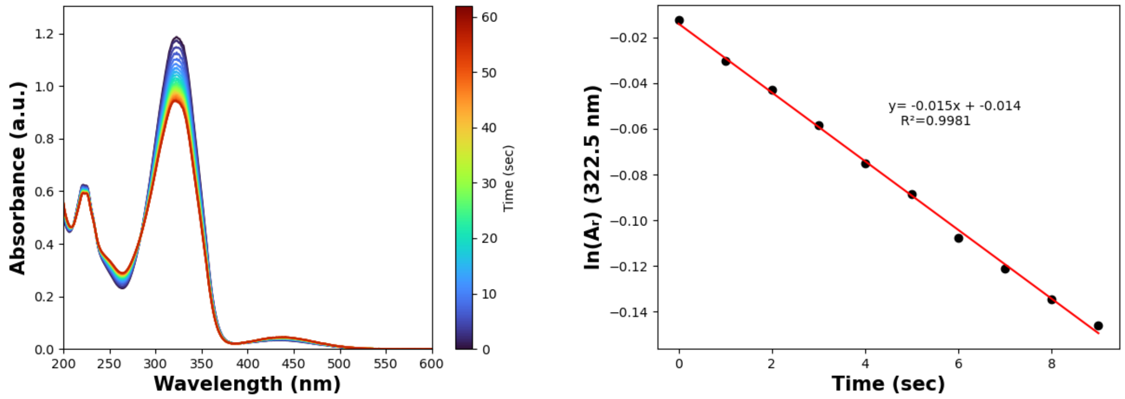

Figure S71: Left: Time-resolved UV–vis absorption spectra of **NAc-PAP-CF$_3$** (50 µM in CH$_3$CN) upon 445 nm irradiation. Right: Linear fit of the logarithmic kinetic trace of the change of absorbance at the absorption maximum of **NAc-PAP-CF$_3$**.

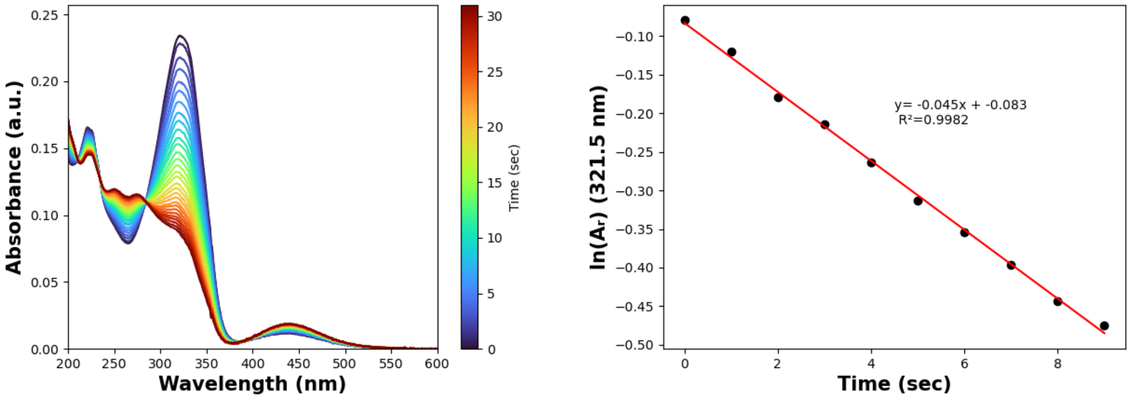

Figure S72: Left: Time-resolved UV–vis absorption spectra of **NAc-PAP-CF$_3$** (50 µM in CH$_3$CN) upon 365 nm irradiation. Right: Linear fit of the logarithmic kinetic trace of the change of absorbance at the absorption maximum of **NAc-PAP-CF$_3$**.

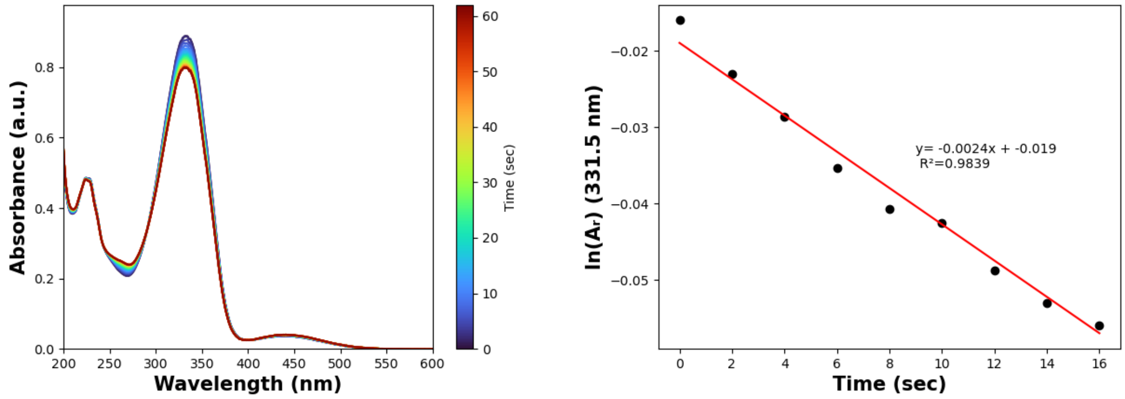

Figure S73: Left: Time-resolved UV–vis absorption spectra of **NAc-PAP-CN** (50 µM in CH$_3$CN) upon 445 nm irradiation. Right: Linear fit of the logarithmic kinetic trace of the change of absorbance at the absorption maximum of **NAc-PAP-CN**.

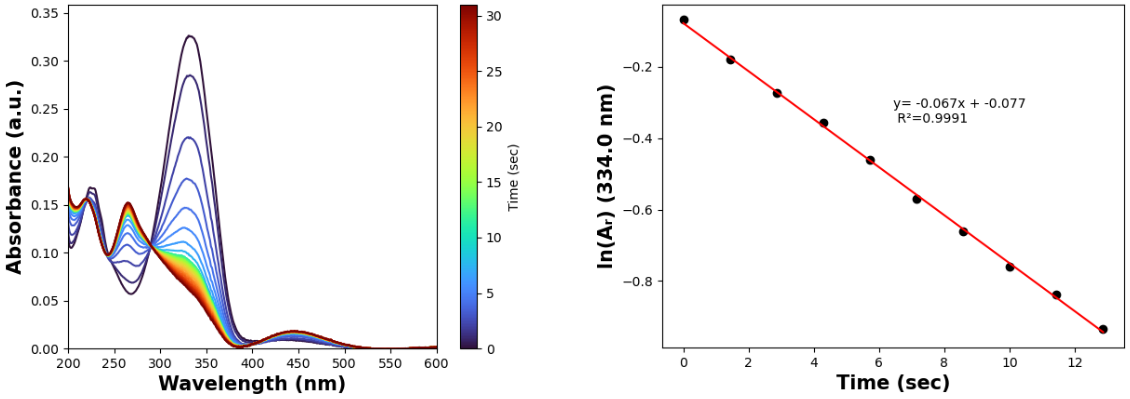

Figure S74: Left: Time-resolved UV–vis absorption spectra of **NAc-PAP-CN** (12.5 µM in CH_3CN) upon 365 nm irradiation. Right: Linear fit of the logarithmic kinetic trace of the change of absorbance at the absorption maximum of **NAc-PAP-CN**.

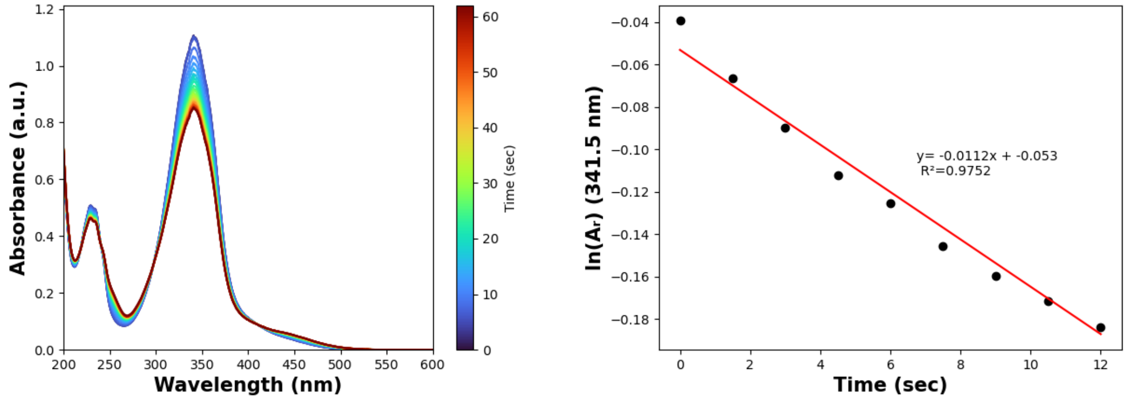

Figure S75: Left: Time-resolved UV–vis absorption spectra of **NAc-PAP-Cl** (50 µM in CH_3CN) upon 445 nm irradiation. Right: Linear fit of the logarithmic kinetic trace of the change of absorbance at the absorption maximum of **NAc-PAP-Cl**.

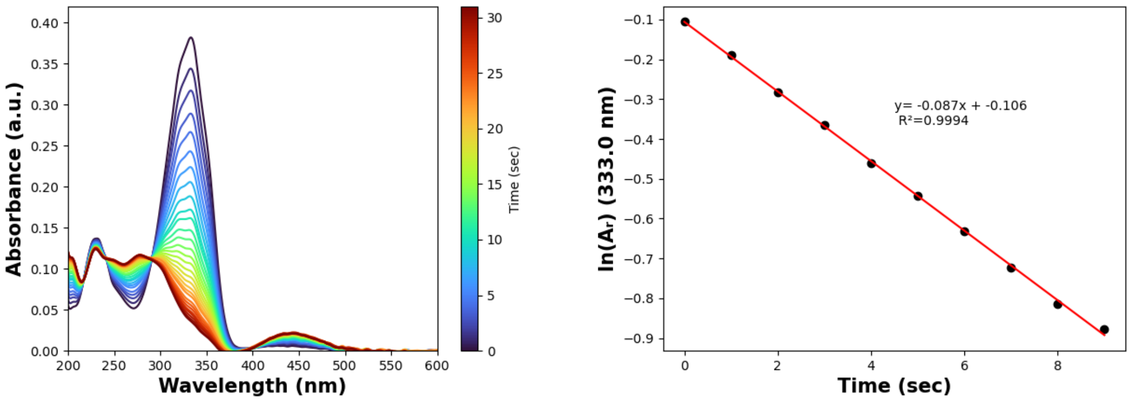

Figure S76: Left: Time-resolved UV–vis absorption spectra of **NAc-PAP-Cl** (12.5 µM in CH_3CN) upon 365 nm irradiation. Right: Linear fit of the logarithmic kinetic trace of the change of absorbance at the absorption maximum of **NAc-PAP-Cl**.

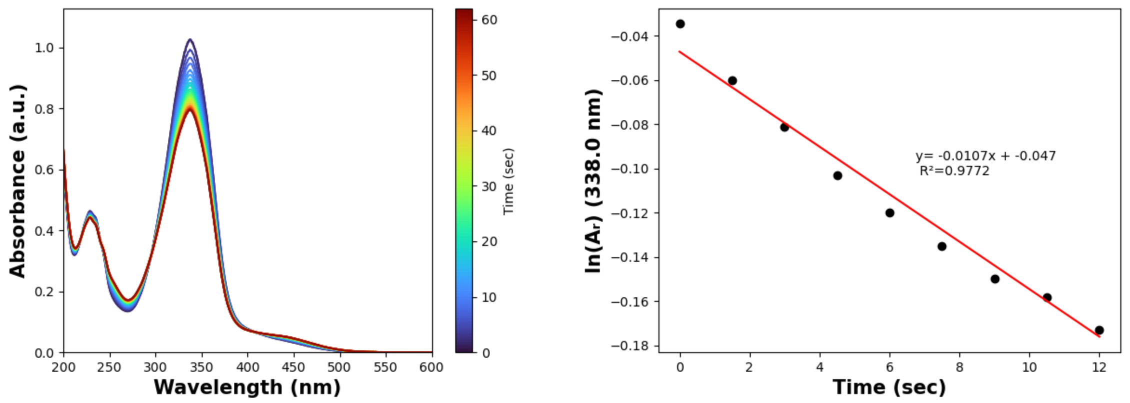

Figure S77: Left: Time-resolved UV–vis absorption spectra of **NAc-PAP-Br** (50 µM in CH_3CN) upon 445 nm irradiation. Right: Linear fit of the logarithmic kinetic trace of the change of absorbance at the absorption maximum of **NAc-PAP-Br**.

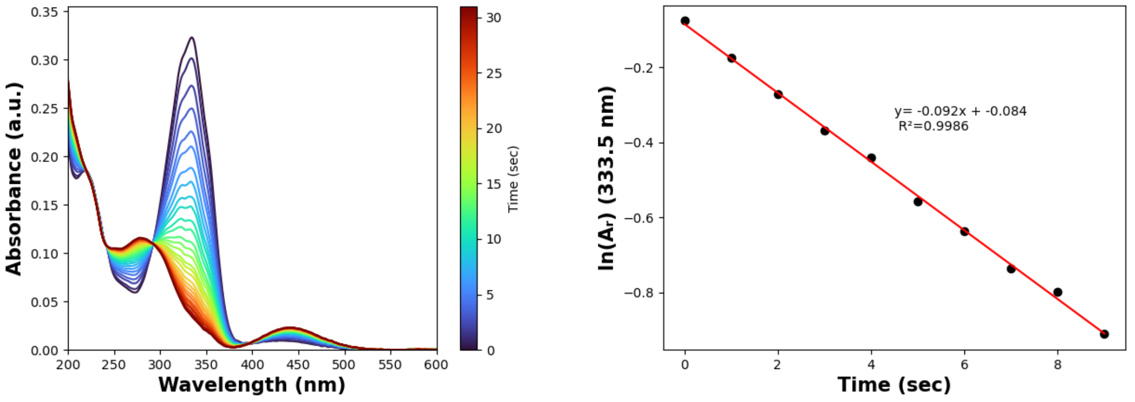

Figure S78: Left: Time-resolved UV–vis absorption spectra of **NAc-PAP-Br** (12.5 µM in CH_3CN) upon 365 nm irradiation. Right: Linear fit of the logarithmic kinetic trace of the change of absorbance at the absorption maximum of **NAc-PAP-Br**.

3.5.2 Irradiation of NH-PAP derivatives and evaluation of the kinetic traces

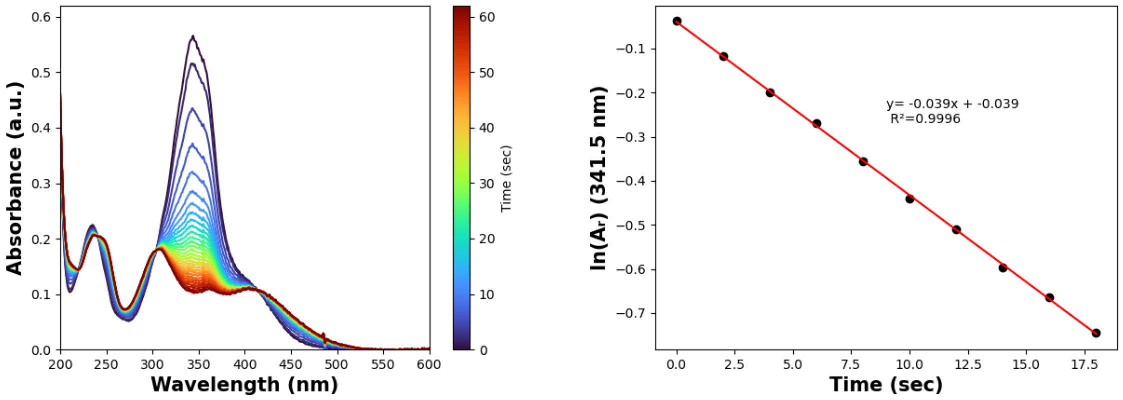

Figure S79: Left: Time-resolved UV–vis absorption spectra of **NH-PAP-OMe** (50 µM in CH_3CN) upon 365 nm irradiation. Right: Linear fit of the logarithmic kinetic trace of the change of absorbance at the absorption maximum of **NH-PAP-OMe**.

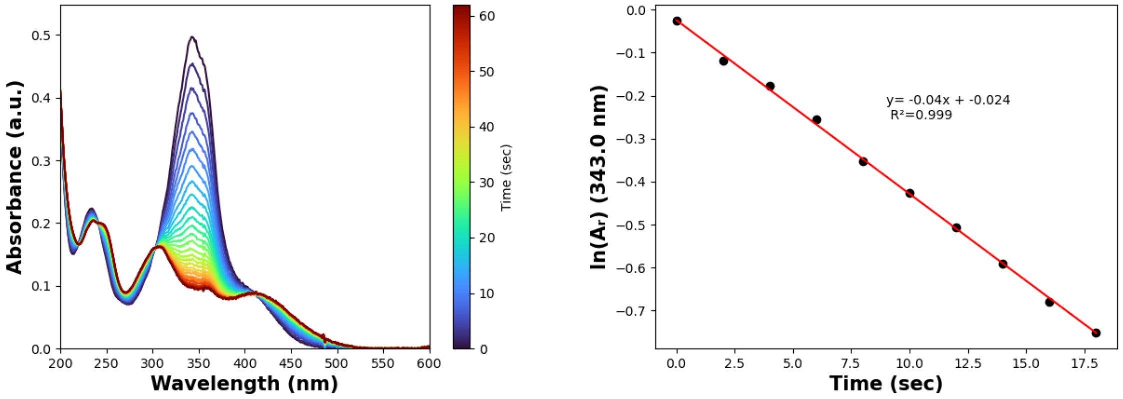

Figure S80: Left: Time-resolved UV–vis absorption spectra of **NH-PAP-OH** (50 µM in CH_3CN) upon 365 nm irradiation. Right: Linear fit of the logarithmic kinetic trace of the change of absorbance at the absorption maximum of **NH-PAP-OH**.

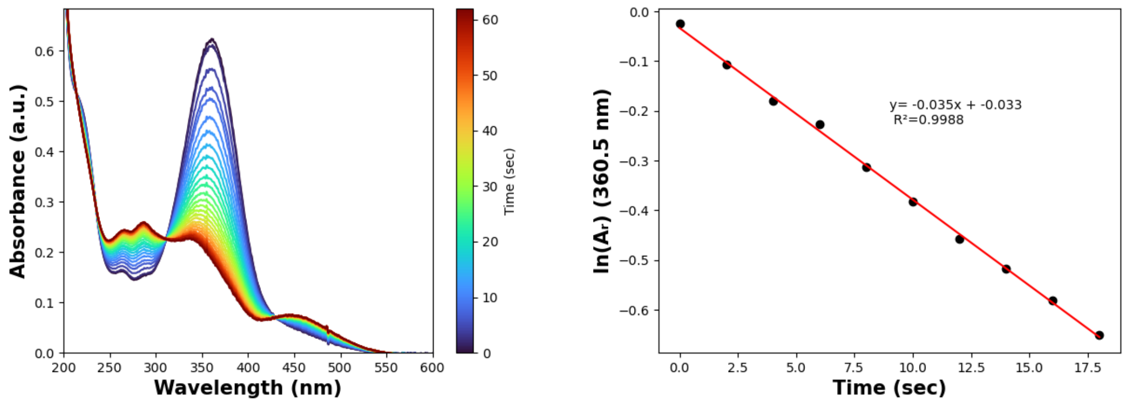

Figure S81: Left: Time-resolved UV–vis absorption spectra of **NH-PAP-NO$_2$** (25 µM in CH_3CN) upon 365 nm irradiation. Right: Linear fit of the logarithmic kinetic trace of the change of absorbance at the absorption maximum of **NH-PAP-NO$_2$**.

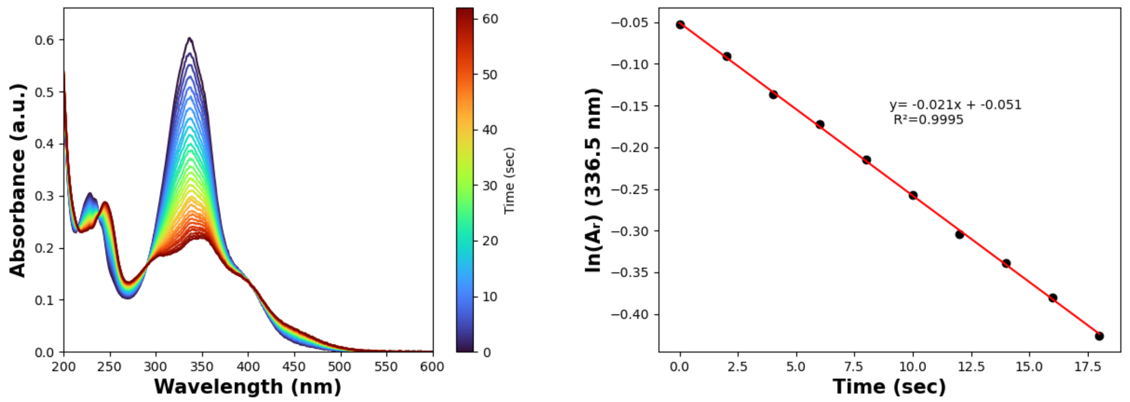

Figure S82: Left: Time-resolved UV–vis absorption spectra of **NH-PAP-Me** (25 µM in CH_3CN) upon 365 nm irradiation. Right: Linear fit of the logarithmic kinetic trace of the change of absorbance at the absorption maximum of **NH-PAP-Me**.

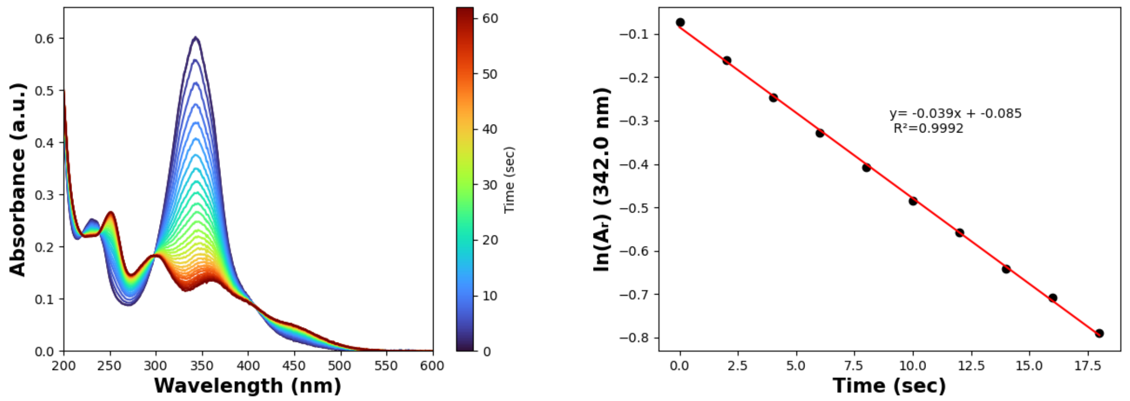

Figure S83: Left: Time-resolved UV–vis absorption spectra of **NH-PAP-I** (25 µM in CH_3CN) upon 365 nm irradiation. Right: Linear fit of the logarithmic kinetic trace of the change of absorbance at the absorption maximum of **NH-PAP-I**.

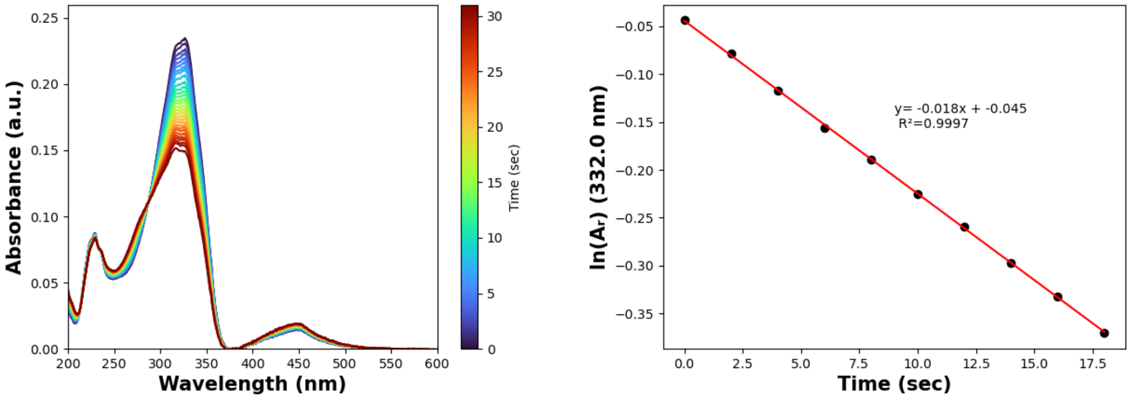

Figure S84: Left: Time-resolved UV–vis absorption spectra of **NH-PAP-H** (25 µM in CH_3CN) upon 365 nm irradiation. Right: Linear fit of the logarithmic kinetic trace of the change of absorbance at the absorption maximum of **NH-PAP-H**.

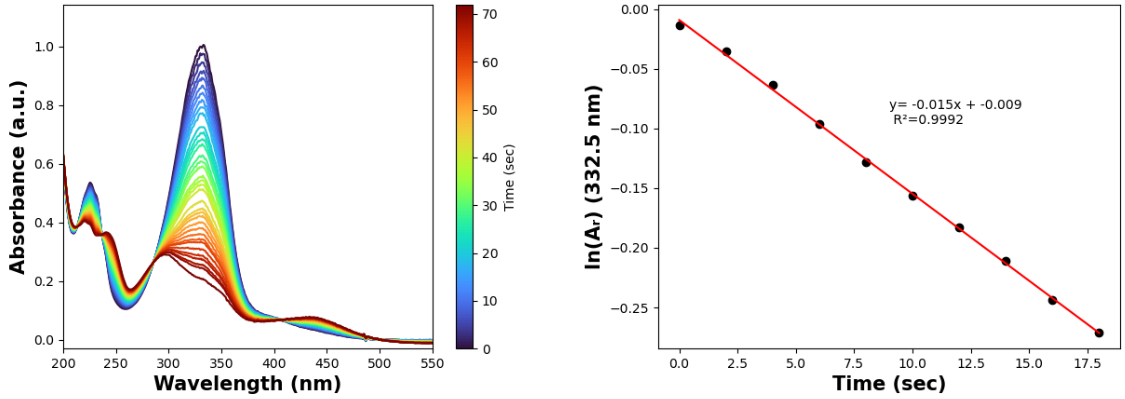

Figure S85: Left: Time-resolved UV–vis absorption spectra of **NH-PAP-F** (50 µM in CH_3CN) upon 365 nm irradiation. Right: Linear fit of the logarithmic kinetic trace of the change of absorbance at the absorption maximum of **NH-PAP-F**.

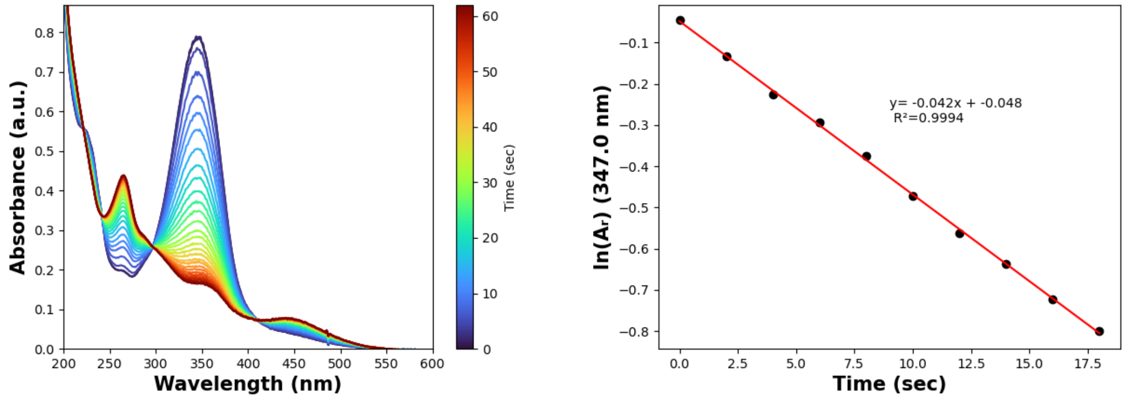

Figure S86: Left: Time-resolved UV–vis absorption spectra of **NH-PAP-CN** (25 µM in CH_3CN) upon 365 nm irradiation. Right: Linear fit of the logarithmic kinetic trace of the change of absorbance at the absorption maximum of **NH-PAP-CN**.

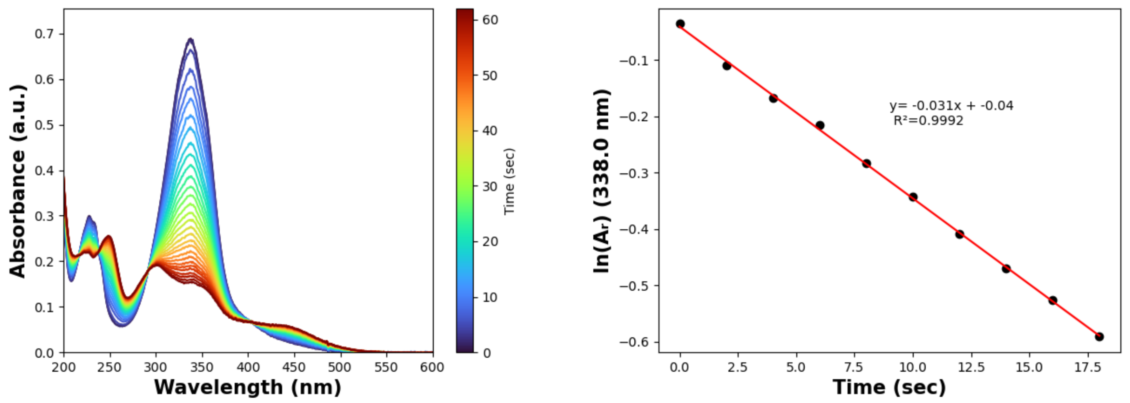

Figure S87: Left: Time-resolved UV–vis absorption spectra of **NH-PAP-Cl** (25 µM in CH_3CN) upon 365 nm irradiation. Right: Linear fit of the logarithmic kinetic trace of the change of absorbance at the absorption maximum of **NH-PAP-Cl**.

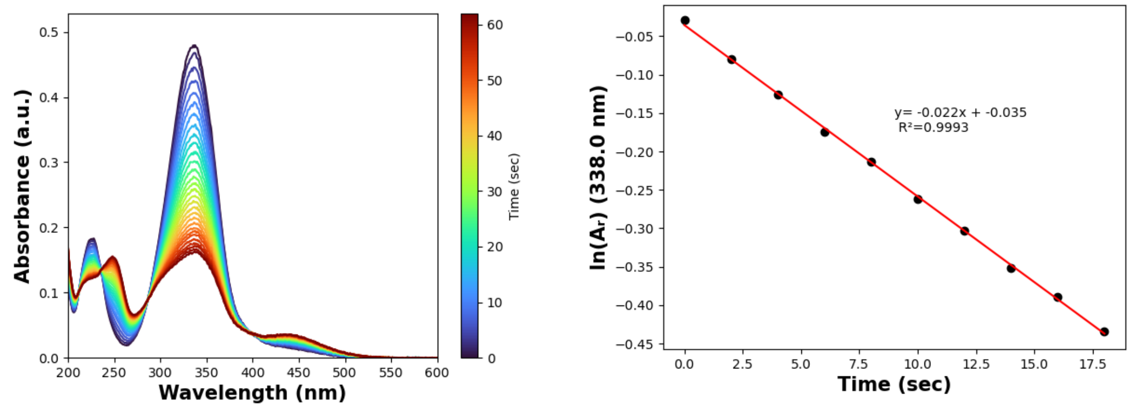

Figure S88: Left: Time-resolved UV–vis absorption spectra of **NH-PAP-CF₃** (25 µM in CH_3CN) upon 365 nm irradiation. Right: Linear fit of the logarithmic kinetic trace of the change of absorbance at the absorption maximum of **NH-PAP-CF₃**.

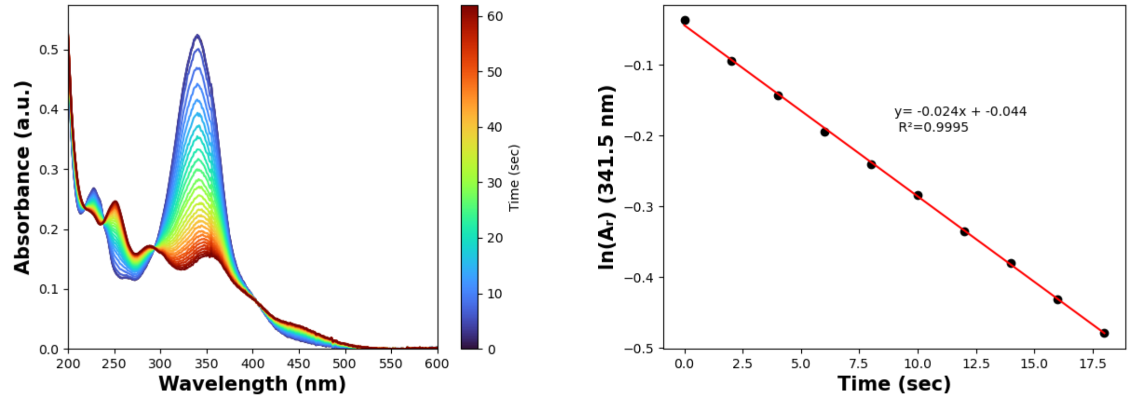

Figure S89: Left: Time-resolved UV–vis absorption spectra of **NH-PAP-Br** (25 µM in CH_3CN) upon 365 nm irradiation. Right: Linear fit of the logarithmic kinetic trace of the change of absorbance at the absorption maximum of **NH-PAP-Br**.

3.5.3 Irradiation of NMe-PAP derivatives and evaluation of the kinetic traces

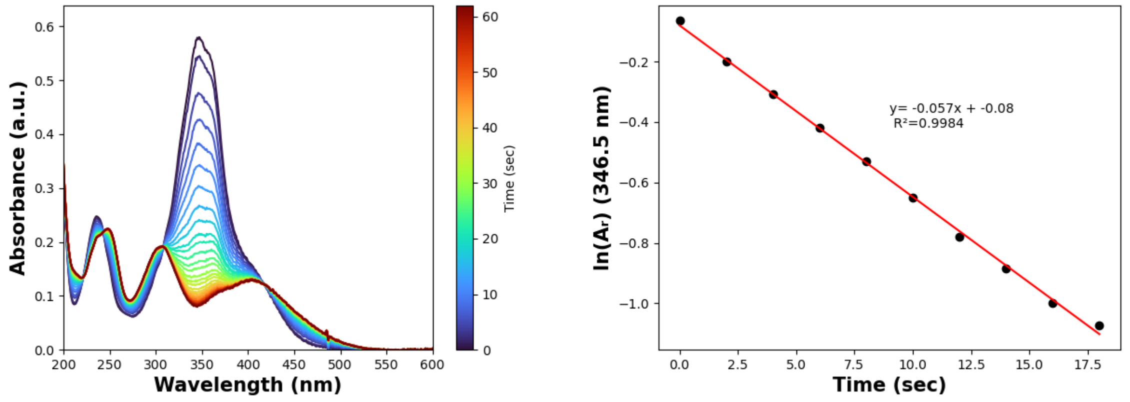

Figure S90: Left: Time-resolved UV–vis absorption spectra of **NMe-PAP-OMe** (25 µM in CH_3CN) upon 365 nm irradiation. Right: Linear fit of the logarithmic kinetic trace of the change of absorbance at the absorption maximum of **NMe-PAP-OMe**.

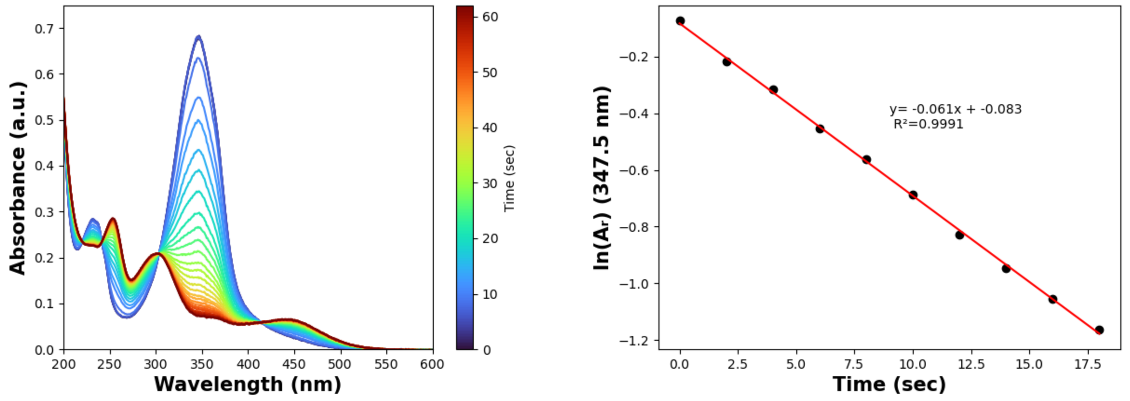

Figure S91: Left: Time-resolved UV–vis absorption spectra of **NMe-PAP-I** (25 µM in CH_3CN) upon 365 nm irradiation. Right: Linear fit of the logarithmic kinetic trace of the change of absorbance at the absorption maximum of **NMe-PAP-I**.

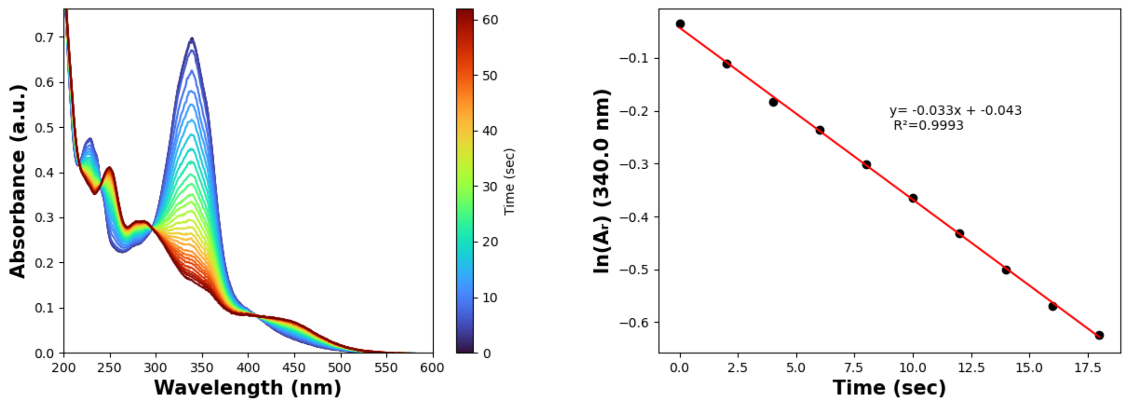

Figure S92: Left: Time-resolved UV–vis absorption spectra of **NMe-PAP-Me** (25 µM in CH_3CN) upon 365 nm irradiation. Right: Linear fit of the logarithmic kinetic trace of the change of absorbance at the absorption maximum of **NMe-PAP-Me**.

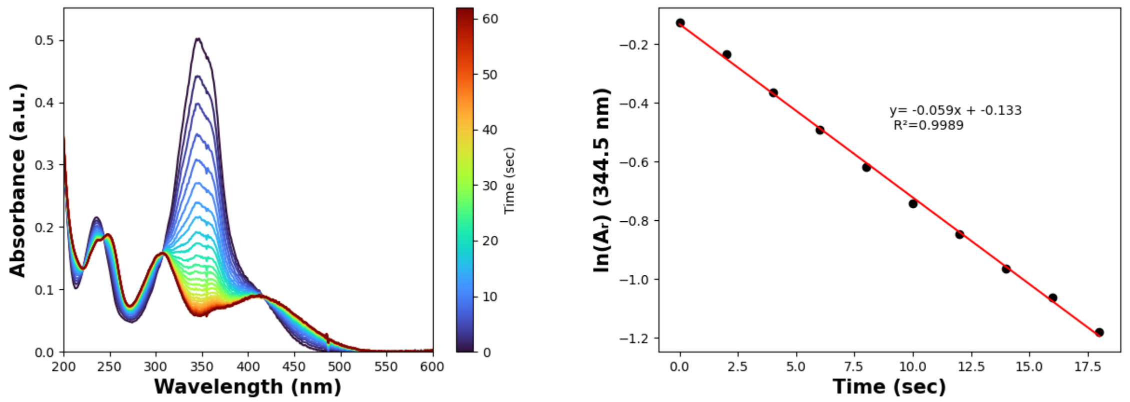

Figure S93: Left: Time-resolved UV–vis absorption spectra of **NMe-PAP-OH** (25 µM in CH_3CN) upon 365 nm irradiation. Right: Linear fit of the logarithmic kinetic trace of the change of absorbance at the absorption maximum of **NMe-PAP-OH**.

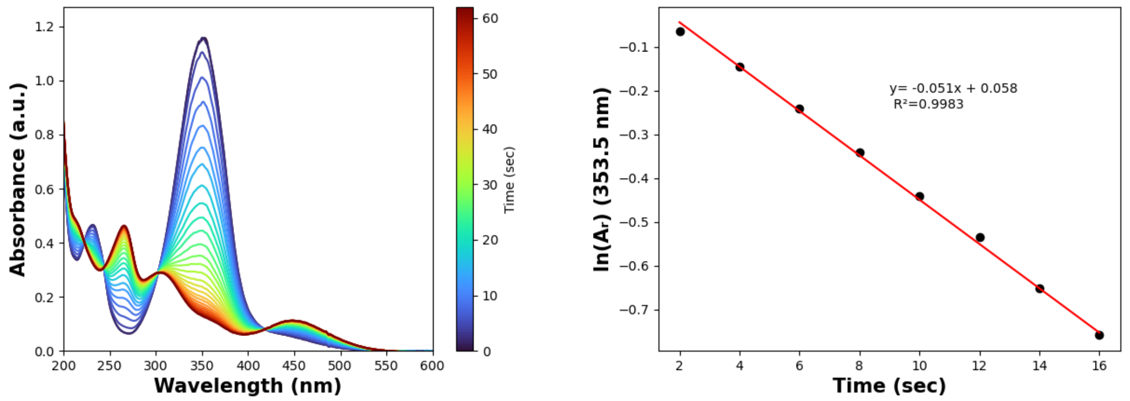

Figure S94: Left: Time-resolved UV–vis absorption spectra of **NMe-PAP-CN** (25 µM in CH_3CN) upon 365 nm irradiation. Right: Linear fit of the logarithmic kinetic trace of the change of absorbance at the absorption maximum of **NMe-PAP-CN**.

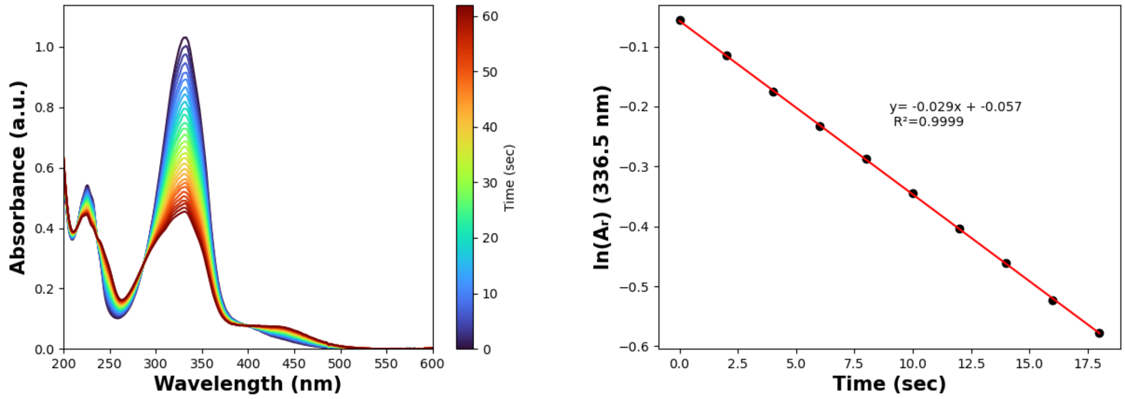

Figure S95: Left: Time-resolved UV–vis absorption spectra of **NMe-PAP-F** (50 µM in CH_3CN) upon 365 nm irradiation. Right: Linear fit of the logarithmic kinetic trace of the change of absorbance at the absorption maximum of **NMe-PAP-F**.

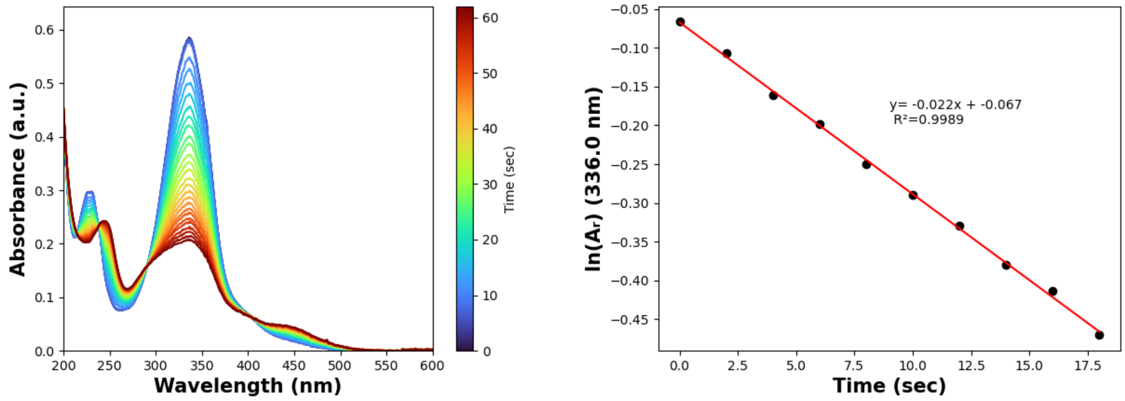

Figure S96: Left: Time-resolved UV–vis absorption spectra of **NMe-PAP-H** (25 µM in CH_3CN) upon 365 nm irradiation. Right: Linear fit of the logarithmic kinetic trace of the change of absorbance at the absorption maximum of **NMe-PAP-H**.

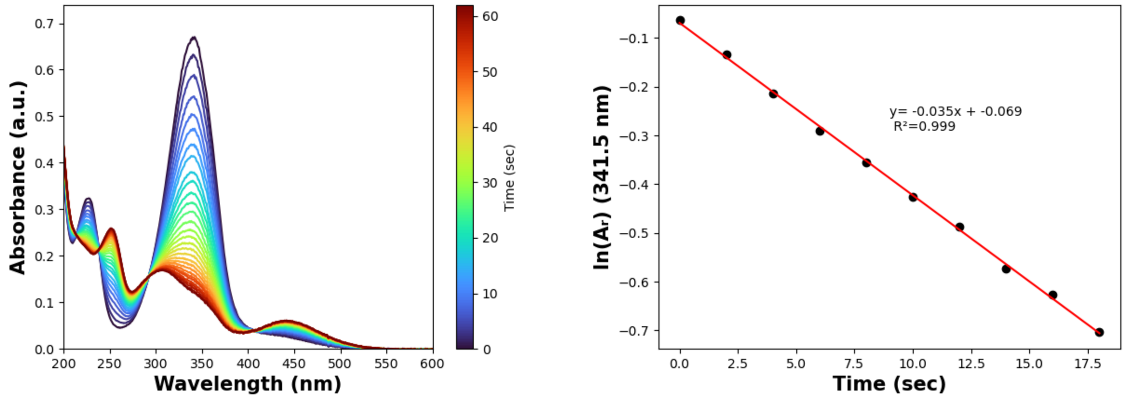

Figure S97: Left: Time-resolved UV–vis absorption spectra of **NMe-PAP-CF$_3$** (25 µM in CH_3CN) upon 365 nm irradiation. Right: Linear fit of the logarithmic kinetic trace of the change of absorbance at the absorption maximum of **NMe-PAP-CF$_3$**.

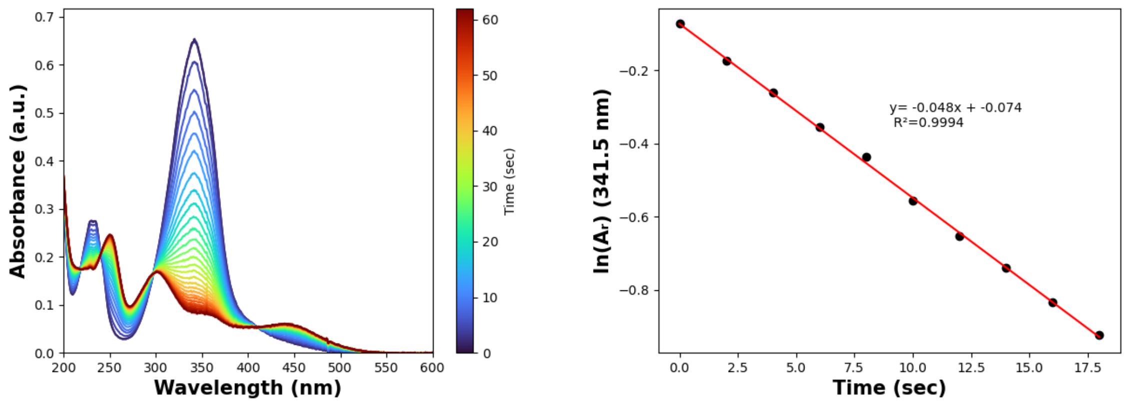

Figure S98: Left: Time-resolved UV–vis absorption spectra of **NMe-PAP-Cl** (25 µM in CH_3CN) upon 365 nm irradiation. Right: Linear fit of the logarithmic kinetic trace of the change of absorbance at the absorption maximum of **NMe-PAP-Cl**.

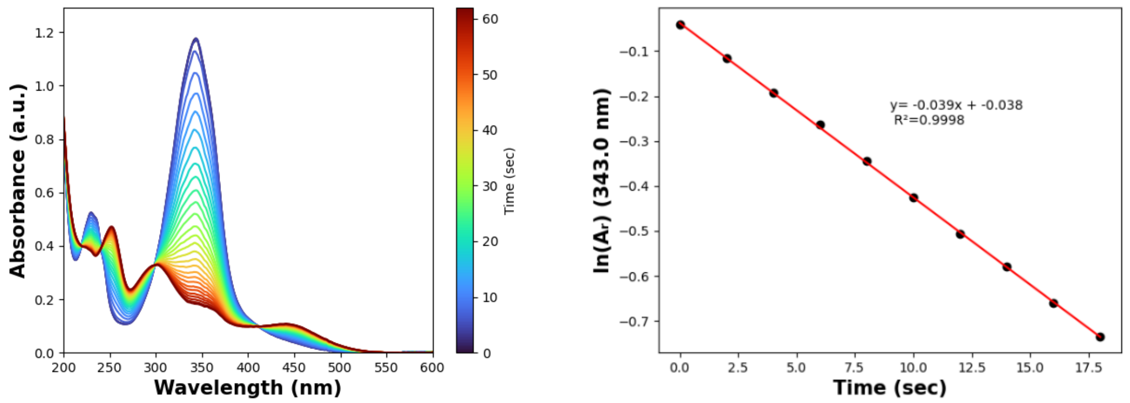

Figure S99: Left: Time-resolved UV–vis absorption spectra of **NMe-PAP-Br** (25 µM in CH_3CN) upon 365 nm irradiation. Right: Linear fit of the logarithmic kinetic trace of the change of absorbance at the absorption maximum of **NMe-PAP-Br**.

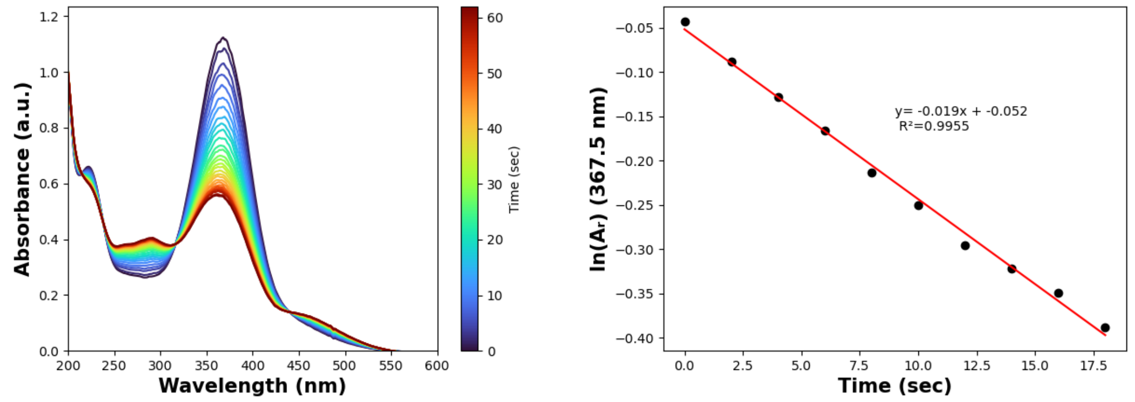

Figure S100: Left: Time-resolved UV–vis absorption spectra of **NMe-PAP-NO$_2$** (50 µM in CH_3CN) upon 365 nm irradiation. Right: Linear fit of the logarithmic kinetic trace of the change of absorbance at the absorption maximum of **NMe-PAP-NO$_2$**.

3.6 Determination of thermal half-lives

Determination of the thermal half-lives $\tau_{1/2}$ of the metastable *Z* isomers were recorded as follows. A freshly prepared solution of the **NAc-PAPs** of interest in CH_3CN was irradiated at 365 nm until reaching the PSS_{365nm} and immediately moved to a Jasco V-670 spectrometer for thermal back *Z*–*E* isomerization in the dark at 30 °C. Then, a first-order rate constant $-k$ for the thermal back *Z*→*E* isomerization reaction was obtained using equation 2.

$$\ln\left(\frac{A_{\mathrm{t}}}{A_0}\right) = \ln\left(\frac{A_\infty - A_0}{A_\infty - A_{\mathrm{t}}}\right) = -kt \qquad (2)$$

Where A_∞ is the absorbance at λ_{max} before irradiation (e.g. >>99 % trans-Isomer); A_0 is the absorbance at λ_{max} at PSS_{365nm} and A_{t} is the absorbance at λ_{max} at a certain time.

The linearized data is shown in black, the fit is shown in red.

From $-k$ the half-live $\tau_{1/2}$ can be calculated as shown in equation 3.

$$\tau_{1/2} = \frac{\ln(2)}{k} \qquad (3)$$

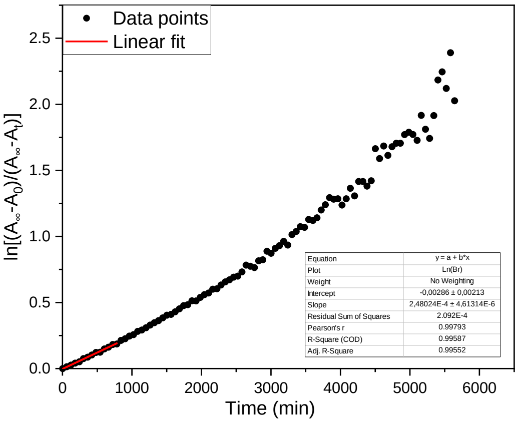

Figure S101: Thermal *Z*-*E* isomerization of **NAc-PAP-Br** in CH_3CN at 30 °C.

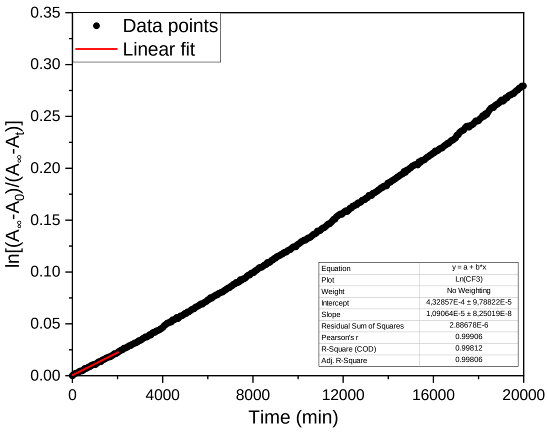

Figure S102: Thermal *Z*-*E* isomerization of **NAc-PAP-CF₃** in CH_3CN at 30 °C.

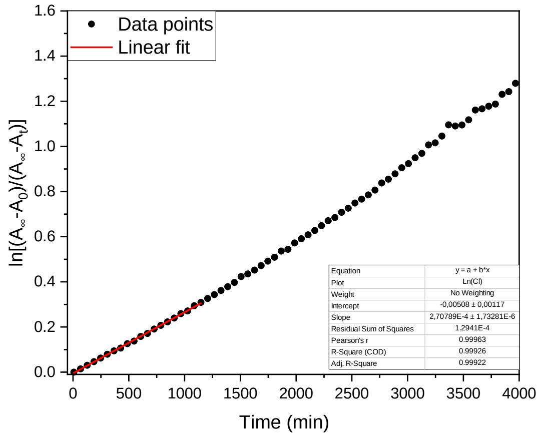

Figure S103: Thermal *Z*-*E* isomerization of **NAc-PAP-Cl** in CH_3CN at 30 °C.

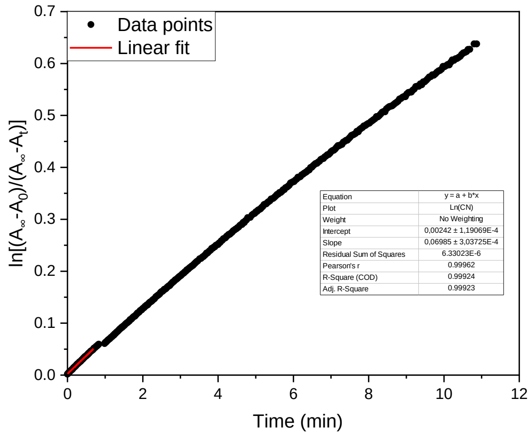

Figure S104: Thermal *Z*-*E* isomerization of **NAc-PAP-CN** in CH_3CN at 30 °C.

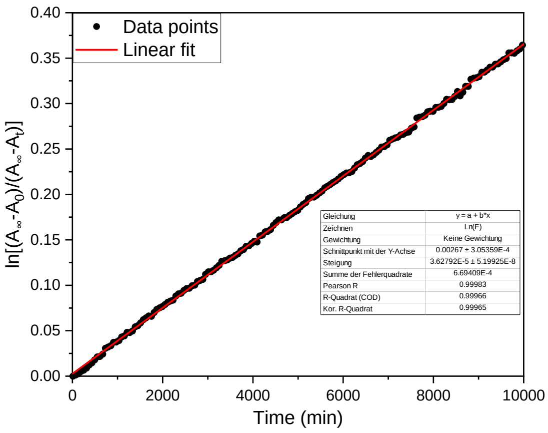

Figure S105: Thermal *Z*-*E* isomerization of **NAc-PAP-F** in CH_3CN at 30 °C.

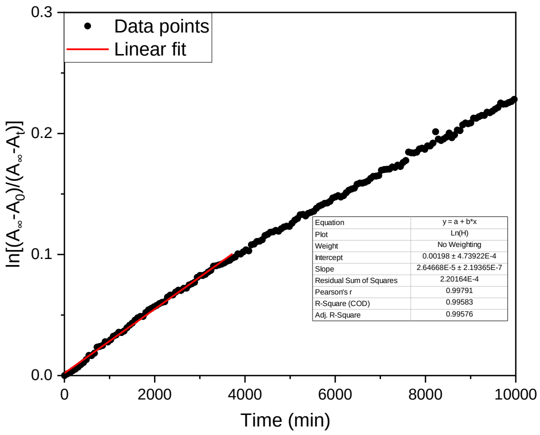

Figure S106: Thermal *Z*-*E* isomerization of **NAc-PAP-H** in CH_3CN at 30 °C.

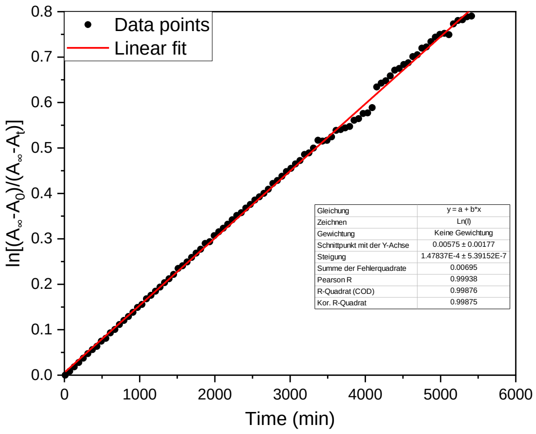

Figure S107: Thermal *Z*-*E* isomerization of **NAc-PAP-I** in CH_3CN at 30 °C.

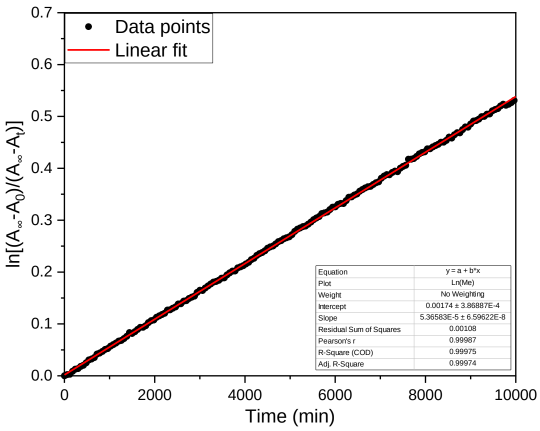

Figure S108: Thermal *Z*-*E* isomerization of **NAc-PAP-Me** in CH_3CN at 30 °C.

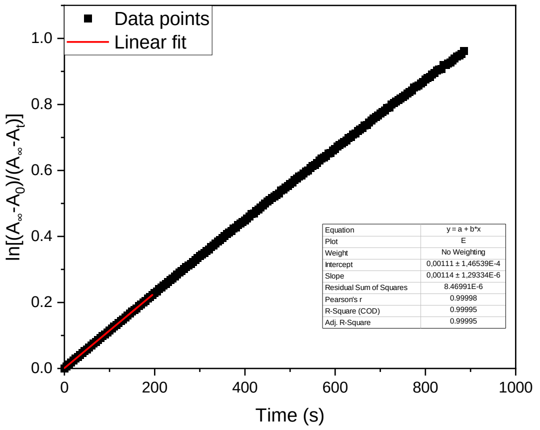

Figure S109: Thermal *Z*-*E* isomerization of **NAc-PAP-NO₂** in CH_3CN at 30 °C.

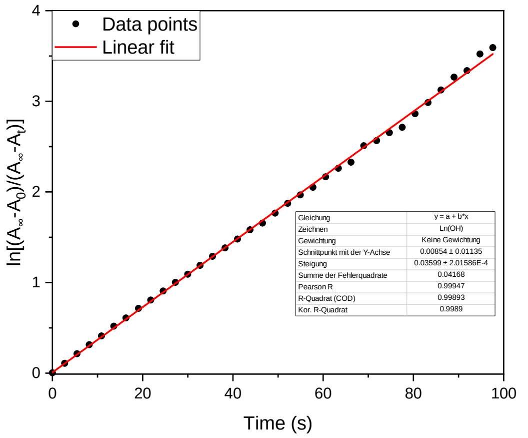

Figure S110: Thermal *Z*-*E* isomerization of **NAc-PAP-OH** in CH_3CN at 30 °C.

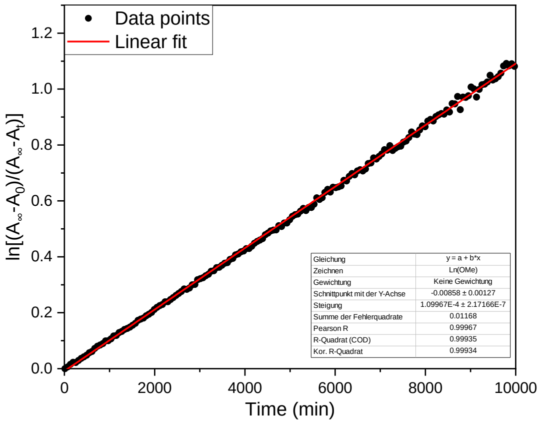

Figure S111: Thermal *Z-E* isomerization of **NAc-PAP-OMe** in CH_3CN at 30 °C.

3.7 Hammett correlation of thermal half-lives of NAc-PAP derivatives

The correlation of thermal relaxation with the R-substituent parameters resulted in the Hammett plot, depicted in Figure S112. We found the best correlation using the Hammett substitution constant σ resulting in two linear fits (OH was treated as exception due to the possible presence of a tautomerism mechanism).

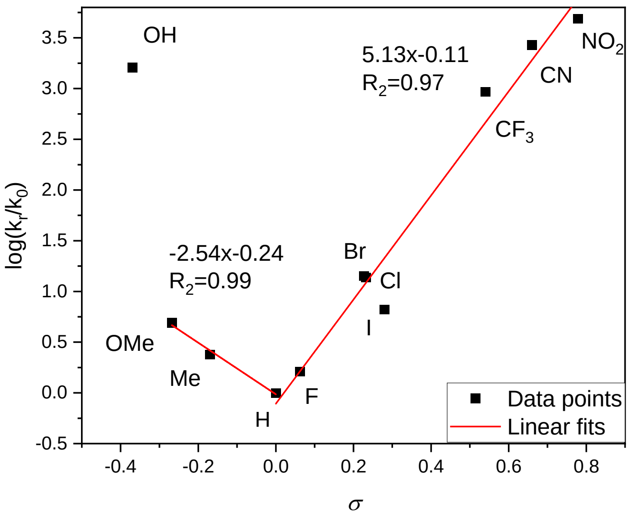

Figure S112: Hammett plot of thermal relaxation for **NAc-PAP** derivatives.

To get a deeper insight into the nature of those, two representative compounds were chosen (**NAc-PAP-CN** and **NAc-PAP-OMe**) and their *Z*→*E* thermal relaxation was measured at different temperatures in toluene. Three measurements were conducted at every temperature and were then fitted to the linearized form of the Eyring equation:

$$ln\frac{k}{T} = \frac{-\Delta H^{\ddagger}}{RT} + ln\frac{k_B}{h} + \frac{\Delta S^{\ddagger}}{R} \quad (4)$$

where ΔH‡ is activation enthalpy, ΔS‡ is activation entropy, *R* is the universal gas constant, *T* is the temperature, *k* the kinetic constant, *h* is the Planck constant, and k_B is the Boltzmann constant. By numerically fitting the data using the package *lift* as implemented in Python3, it is possible to obtain the standard errors on the slope and intercept and directly the standard errors on ΔH‡ ($\sigma\Delta H$) and ΔS‡ $\sigma\Delta S$ by multiplying these values with *R*. Using the covariance matrix obtained from the fit, it is possible to numerically obtain the correlation between ΔH‡ and ΔS‡ ($\rho\Delta H\Delta S$) which resulted to be close to 1 for both CN- (0.9979) and MeO- (0.9995) derivatives. This correlation can be used to obtain the error on the Gibbs free energy of activation, ΔG‡, by exploiting the formula

$$\sigma_{\Delta G} = \sqrt{\sigma_{\Delta H}^2 + T^2 + \sigma_{\Delta S}^2 - 2T\rho_{\Delta H\Delta S}\sigma_{\Delta H}\sigma_{\Delta S}} \quad (5)$$

associated with the canonical form of the Eyring equation

$$\Delta G^{\ddagger} = \Delta H^{\ddagger} - T\Delta S^{\ddagger} \quad (6)$$

Table S2 provides an overview of the measured rates and the resulting thermodynamic parameters and Table S3 of the results of the Eyring analysis of the data set.

Table S2: Thermal relaxation rates (recorded in min^{-1}) of **NAc-PAP-CN** and **NAc-PAP-OMe** in toluene at different temperatures.

Temperatures / °C	**CN**			**OMe**		
20	0.0008	0.0008	0.0008	0.0001	0.0001	0.0001
35	0.0057	0.0054	0.0056	0.0008	0.0007	0.0008
50	0.0251	0.0240	0.0266	0.0040	0.0037	0.0040
65	0.1248	0.1312	0.1280	0.0188	0.0190	0.0179
80	0.5870	0.5390	0.5330	0.0677	0.0705	0.0715

Table S3: Eyring analysis of the measurements in Table S2.

	**NAc-PAP-CN**	**NAc-PAP-OMe**
**$\Delta G^{\ddagger}$** / kJ/mol	99.1 ± 0.07[a]	104.3 ± 0.1[a]
**$\Delta H^{\ddagger}$** / kJ/mol	90.0 ± 0.7	93.0 ± 1.0
**$\Delta S^{\ddagger}$** / J/(mol K)	-30.0 ± 2.0	-39.0 ± 4.0

3.7.1 Thermal relaxation of NAc-PAP-CN at different temperatures

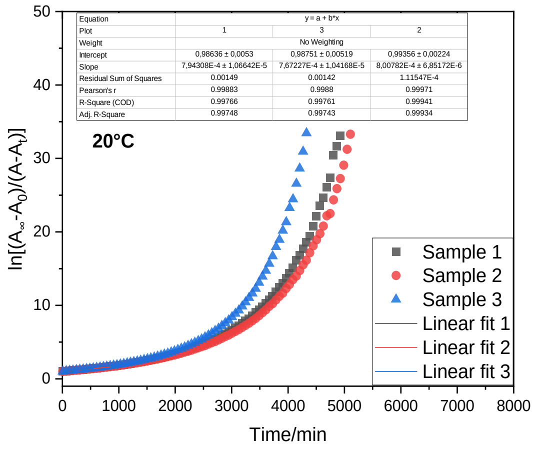

Figure S113: Thermal relaxation absorbance trace of the **NAc-PAP-CN** at 20 °C in toluene.

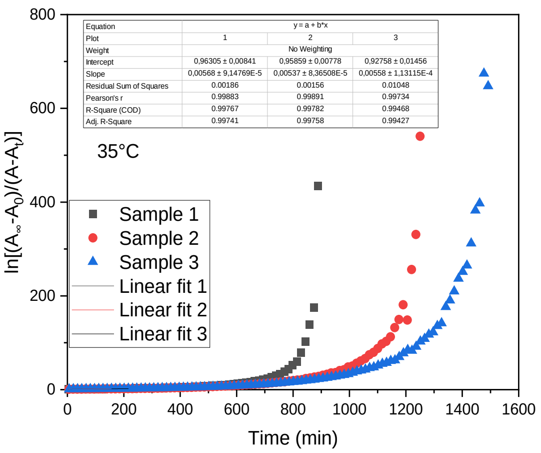

Equation	y = a + b*x		
Plot	1	2	3
Weight	No Weighting		
Intercept	0,96305 ± 0,00841	0,95859 ± 0,00778	0,92758 ± 0,01456
Slope	0,00568 ± 9,14769E-5	0,00537 ± 8,36508E-5	0,00558 ± 1,13115E-4
Residual Sum of Squares	0.00186	0.00156	0.01048
Pearson's r	0.99883	0.99891	0.99734
R-Square (COD)	0.99767	0.99782	0.99468
Adj. R-Square	0.99741	0.99758	0.99427

Figure S114: Thermal relaxation absorbance trace of the **NAc-PAP-CN** at 35 °C in toluene.

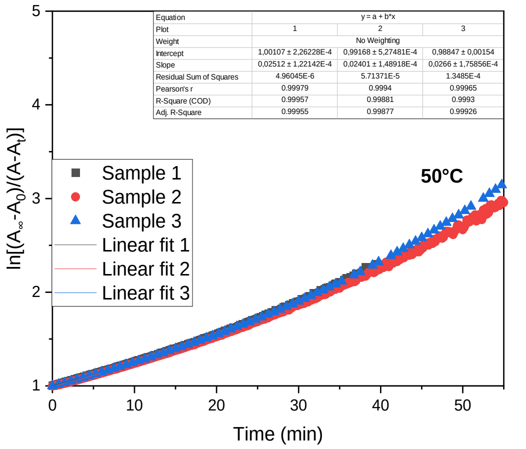

Equation	y = a + b*x		
Plot	1	2	3
Weight	No Weighting		
Intercept	1,00107 ± 2,26228E-4	0,99168 ± 5,27481E-4	0,98847 ± 0,00154
Slope	0,02512 ± 1,22142E-4	0,02401 ± 1,48918E-4	0,0266 ± 1,75856E-4
Residual Sum of Squares	4.96045E-6	5.71371E-5	1.3485E-4
Pearson's r	0.99979	0.9994	0.99965
R-Square (COD)	0.99957	0.99881	0.9993
Adj. R-Square	0.99955	0.99877	0.99926

Figure S115: Thermal relaxation absorbance trace of the **NAc-PAP-CN** at 50 °C in toluene.

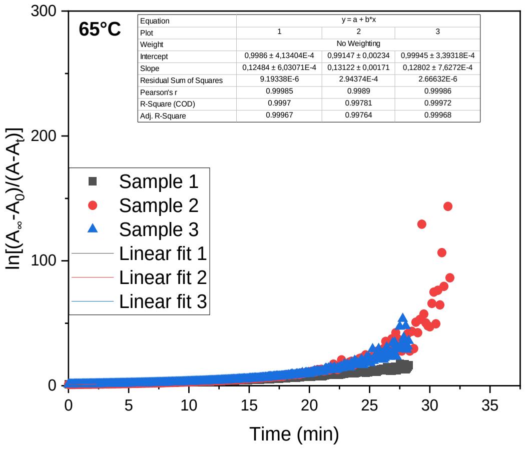

Figure S116: Thermal relaxation absorbance trace of the **NAc-PAP-CN** at 65 °C in toluene.

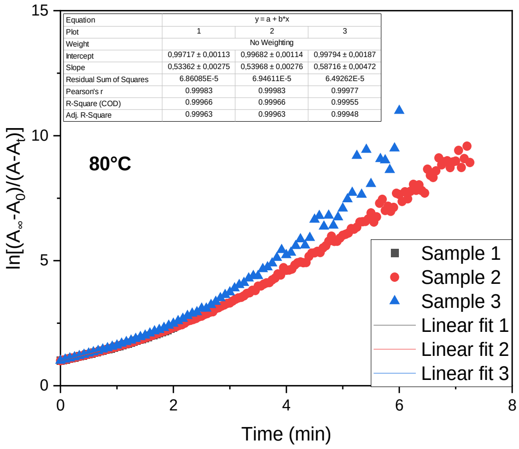

Figure S117: Thermal relaxation absorbance trace of the **NAc-PAP-CN** at 80 °C in toluene.

3.7.2 Thermal relaxation of NAc-PAP-OMe at different temperatures

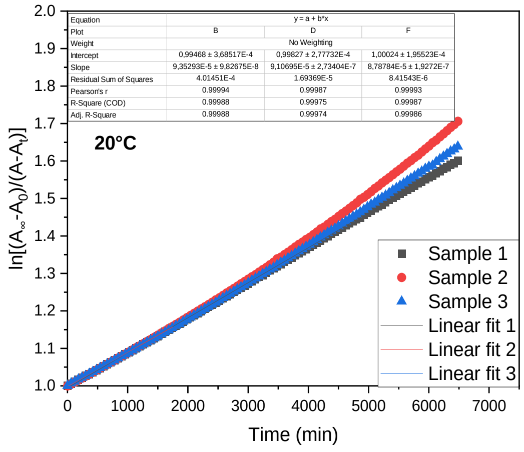

Figure S118: Thermal relaxation absorbance trace of the **NAc-PAP-OMe** at 20 °C in toluene.

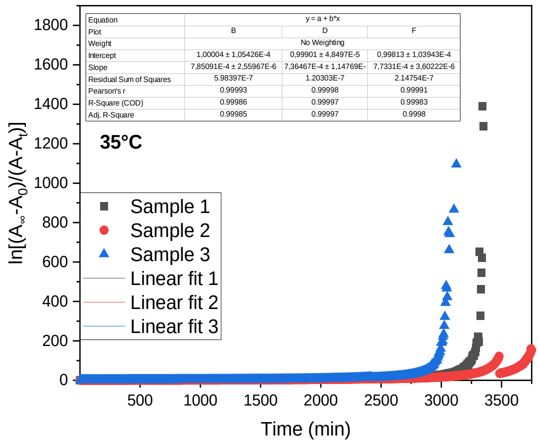

Figure S119: Thermal relaxation absorbance trace of the **NAc-PAP-OMe** at 35 °C in toluene.

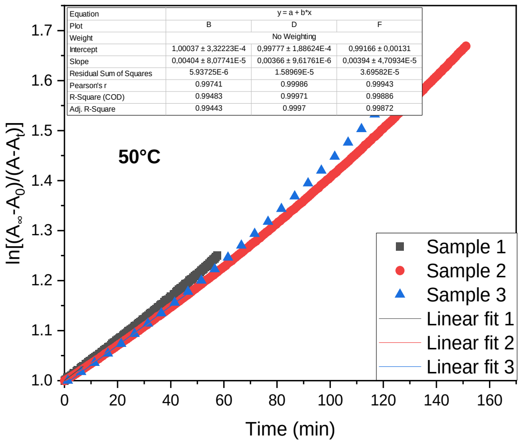

Equation	y = a + b*x		
Plot	B	D	F
Weight	No Weighting		
Intercept	1,00037 ± 3,32223E-4	0,99777 ± 1,88624E-4	0,99166 ± 0,00131
Slope	0,00404 ± 8,07741E-5	0,00366 ± 9,61761E-6	0,00394 ± 4,70934E-5
Residual Sum of Squares	5.93725E-6	1.58969E-5	3.69582E-5
Pearson's r	0.99741	0.99986	0.99943
R-Square (COD)	0.99483	0.99971	0.99886
Adj. R-Square	0.99443	0.9997	0.99872

Figure S120: Thermal relaxation absorbance trace of the **NAc-PAP-OMe** at 50 °C in toluene.

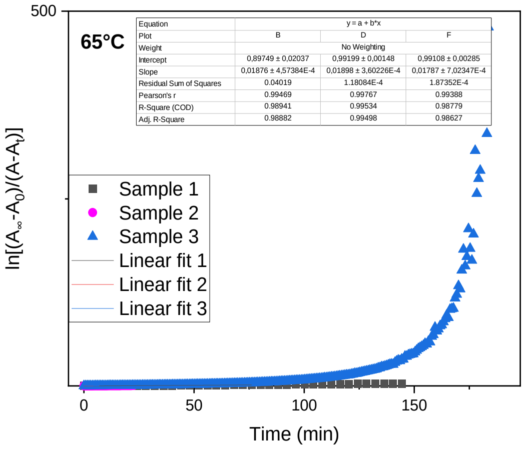

Equation	y = a + b*x		
Plot	B	D	F
Weight	No Weighting		
Intercept	0,89749 ± 0,02037	0,99199 ± 0,00148	0,99108 ± 0,00285
Slope	0,01876 ± 4,57384E-4	0,01898 ± 3,60226E-4	0,01787 ± 7,02347E-4
Residual Sum of Squares	0.04019	1.18084E-4	1.87352E-4
Pearson's r	0.99469	0.99767	0.99388
R-Square (COD)	0.98941	0.99534	0.98779
Adj. R-Square	0.98882	0.99498	0.98627

Figure S121: Thermal relaxation absorbance trace of the **NAc-PAP-OMe** at 65 °C in toluene.

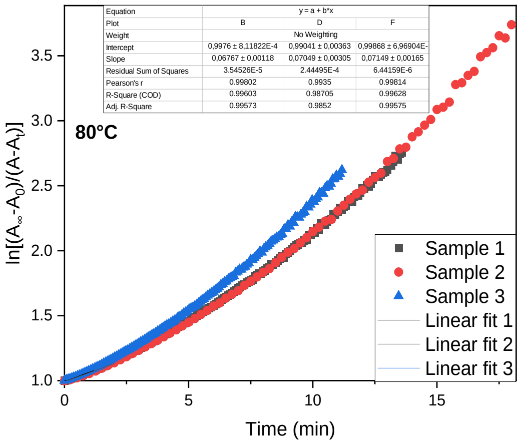

Figure S122: Thermal relaxation absorbance trace of the **NAc-PAP-OMe** at 80 °C in toluene.

4. NMR Spectra

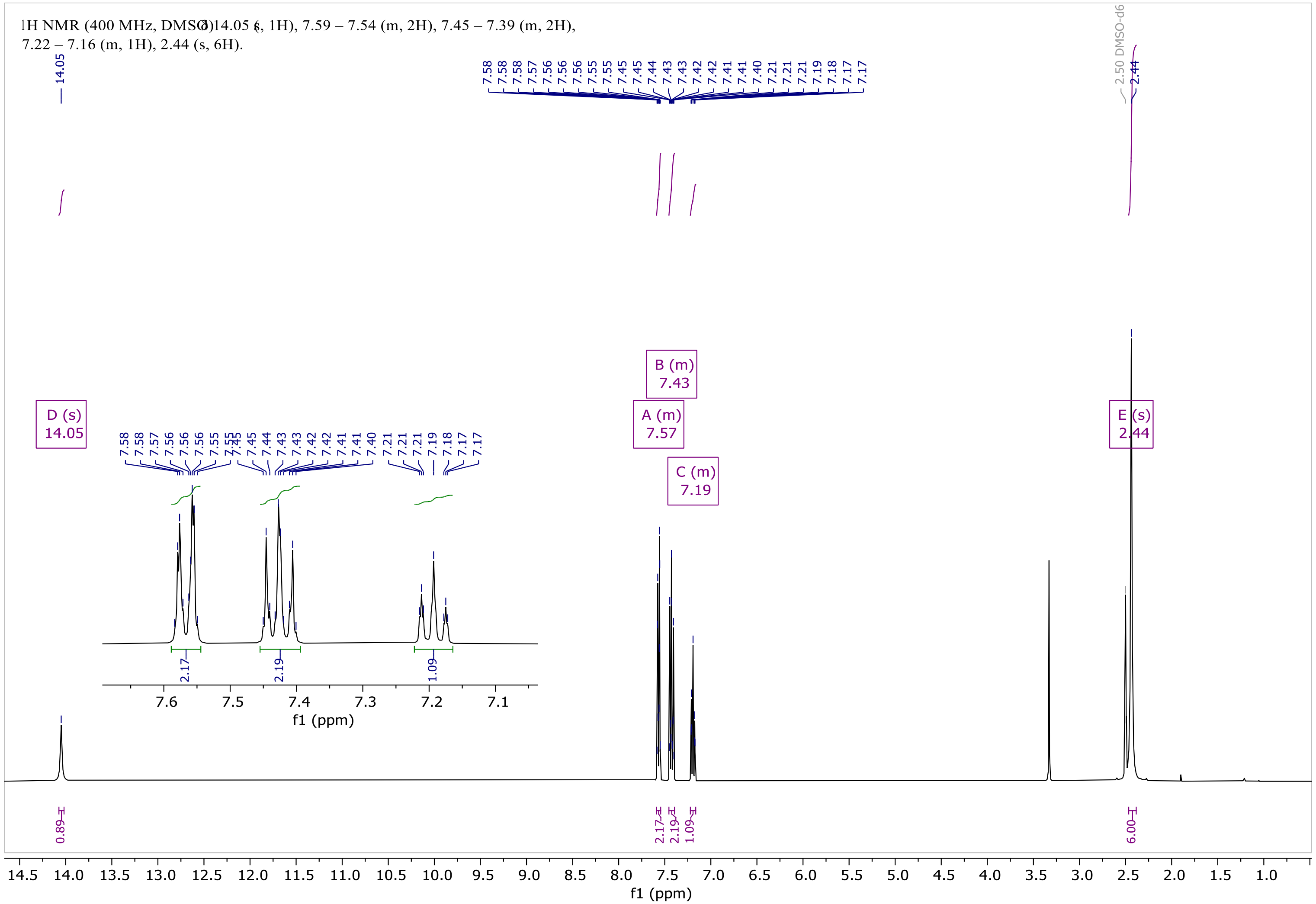

Figure S123: ^{1}H NMR spectrum of 3-(2-phenylhydrazono)pentane-2,4-dione in DMSO-d_6.

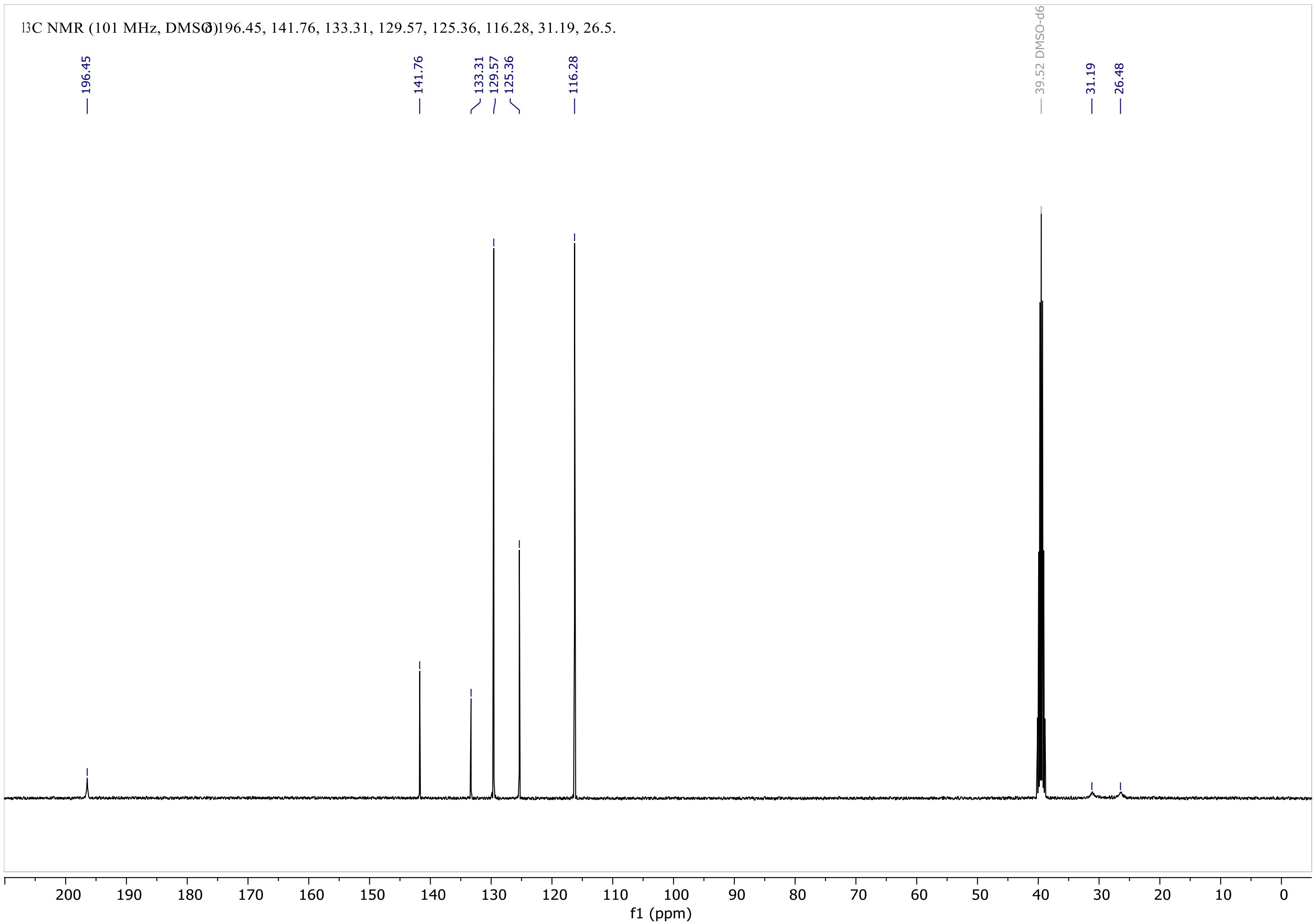

Figure S124: ^{13}C NMR spectrum of 3-(2-phenylhydrazono)pentane-2,4-dione in DMSO-d_6.

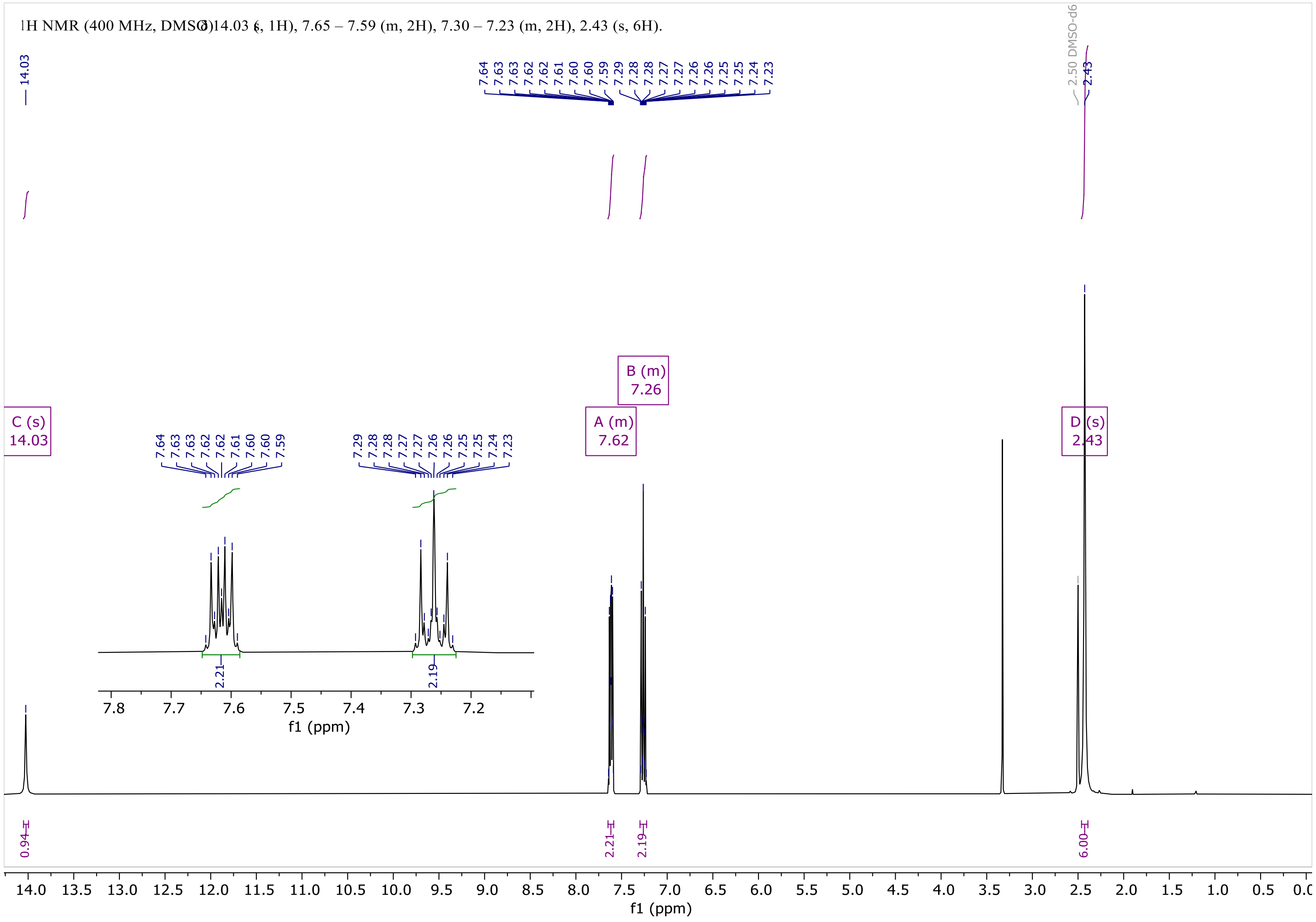

Figure S125: ^{1}H NMR spectrum of 3-(2-(4-fluorophenyl)hydrazono)pentane-2,4-dione in DMSO-d_6.

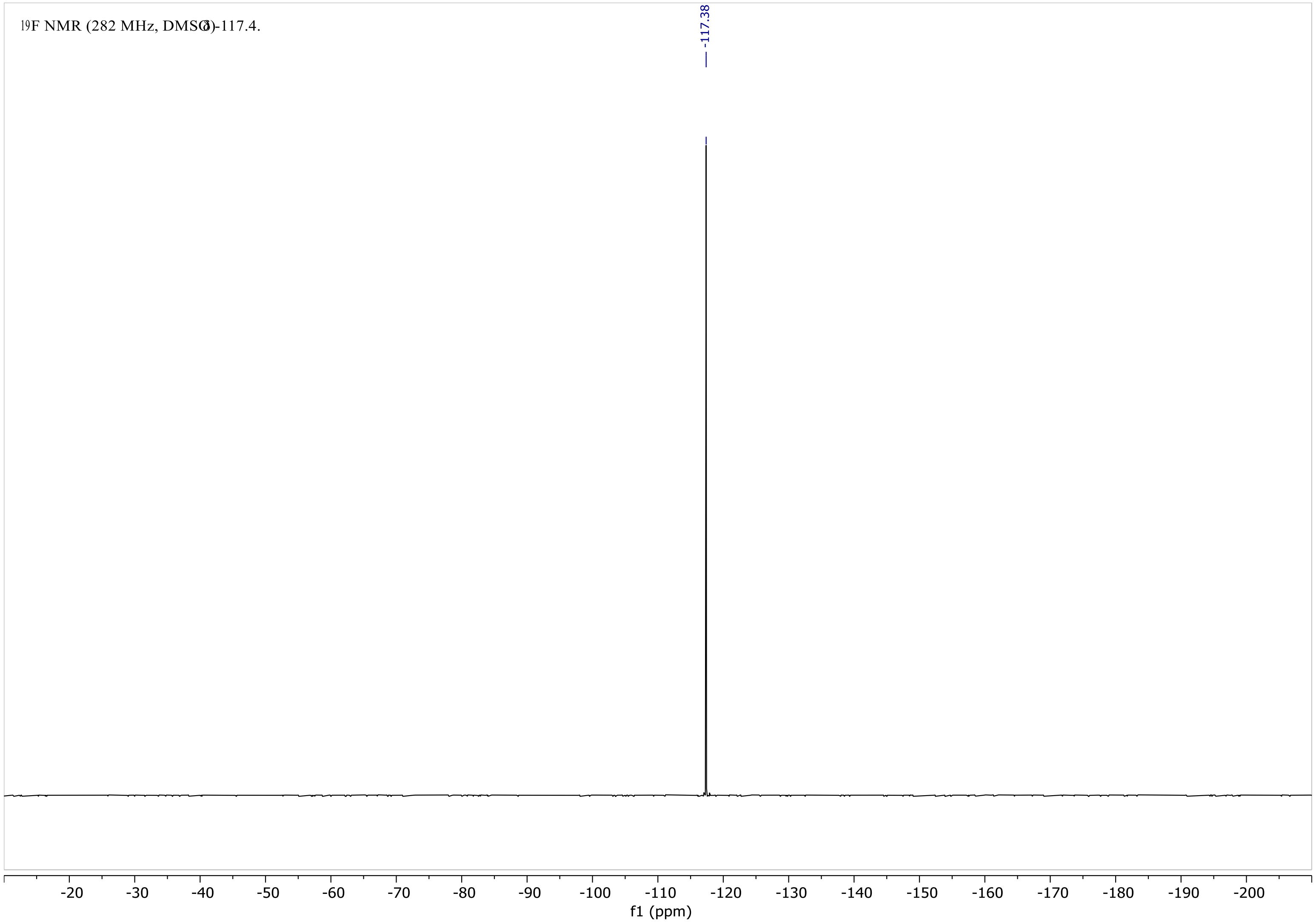

Figure S126: ^{19}F NMR spectrum of 3-(2-(4-fluorophenyl)hydrazono)pentane-2,4-dione in DMSO-d_6.

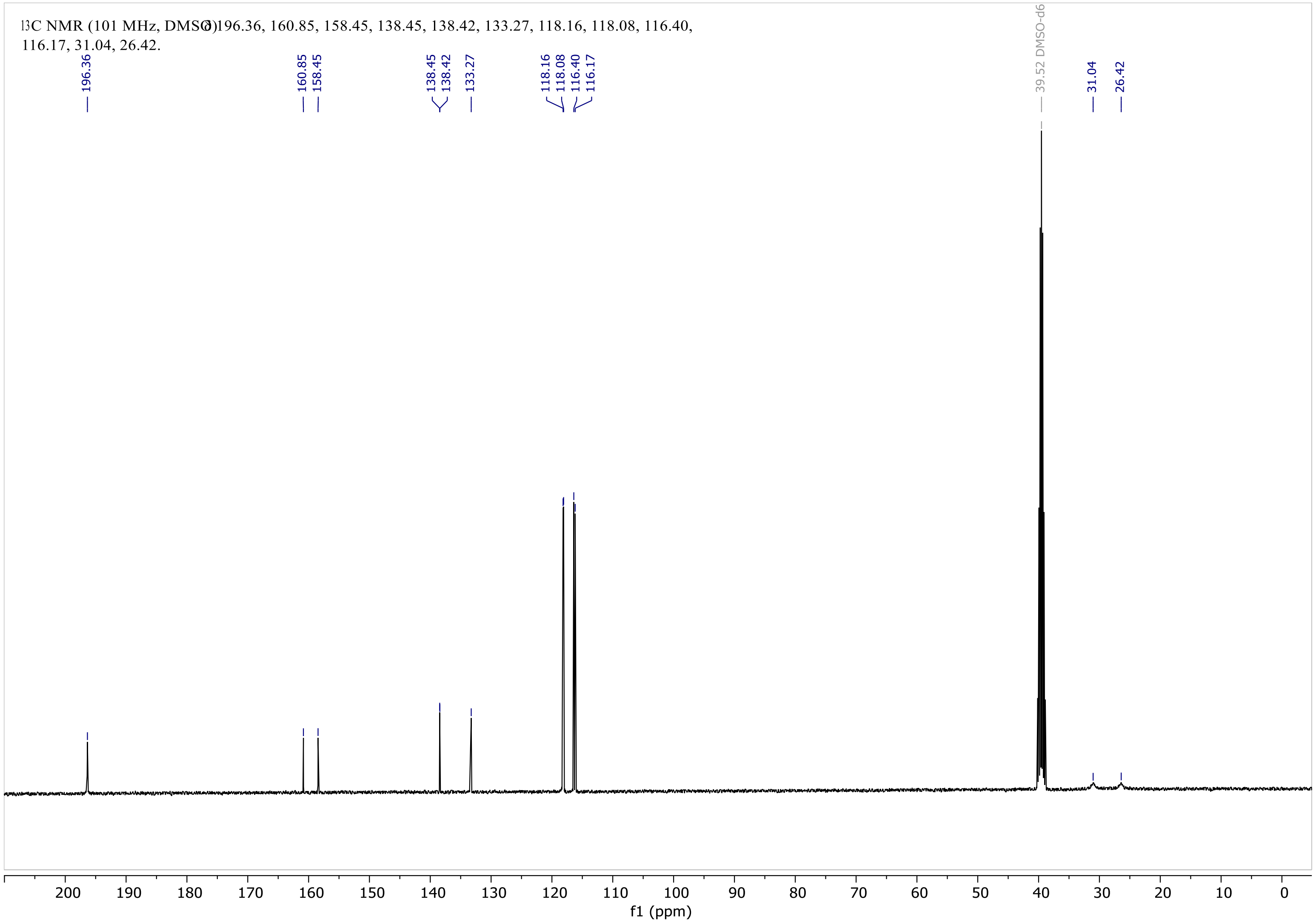

Figure S127: ^{13}C NMR spectrum of 3-(2-(4-fluorophenyl)hydrazono)pentane-2,4-dione in DMSO-d_6.

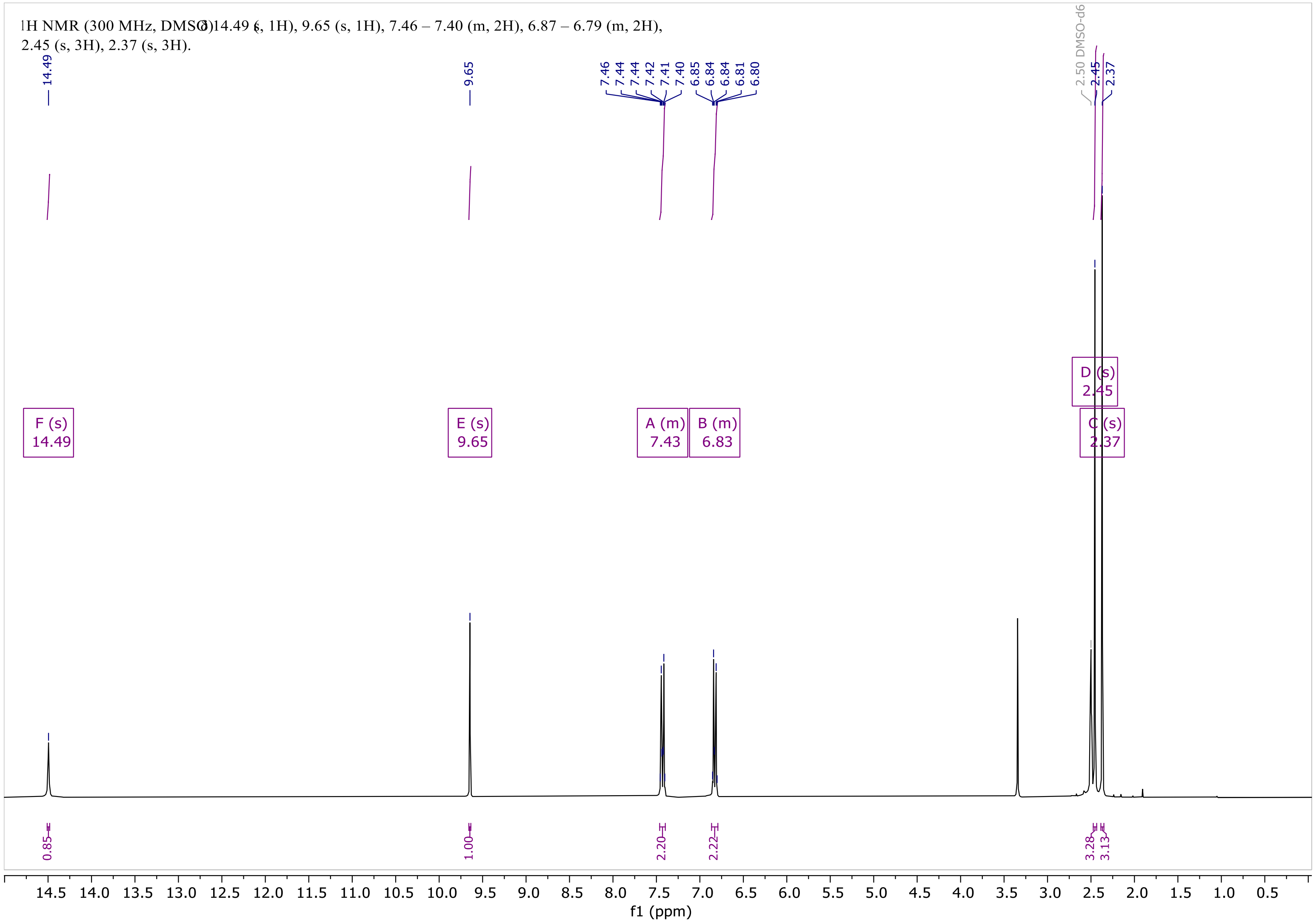

Figure S128: ^{1}H NMR spectrum of 3-(2-(4-hydroxyphenyl)hydrazono)pentane-2,4-dione in DMSO-d_6.

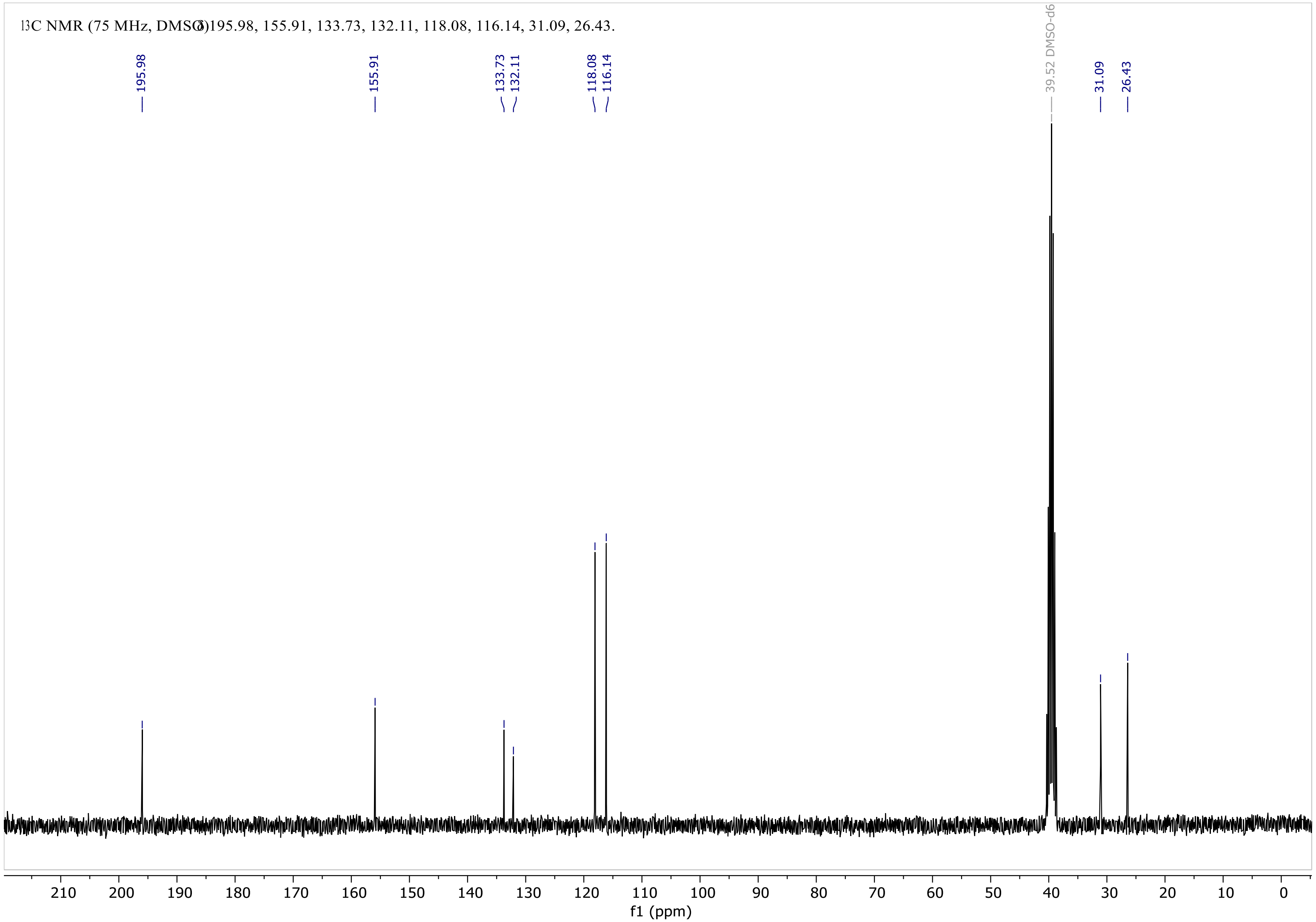

Figure S129: ^{13}C NMR spectrum of 3-(2-(4-hydroxyphenyl)hydrazono)pentane-2,4-dione in DMSO-d_6.

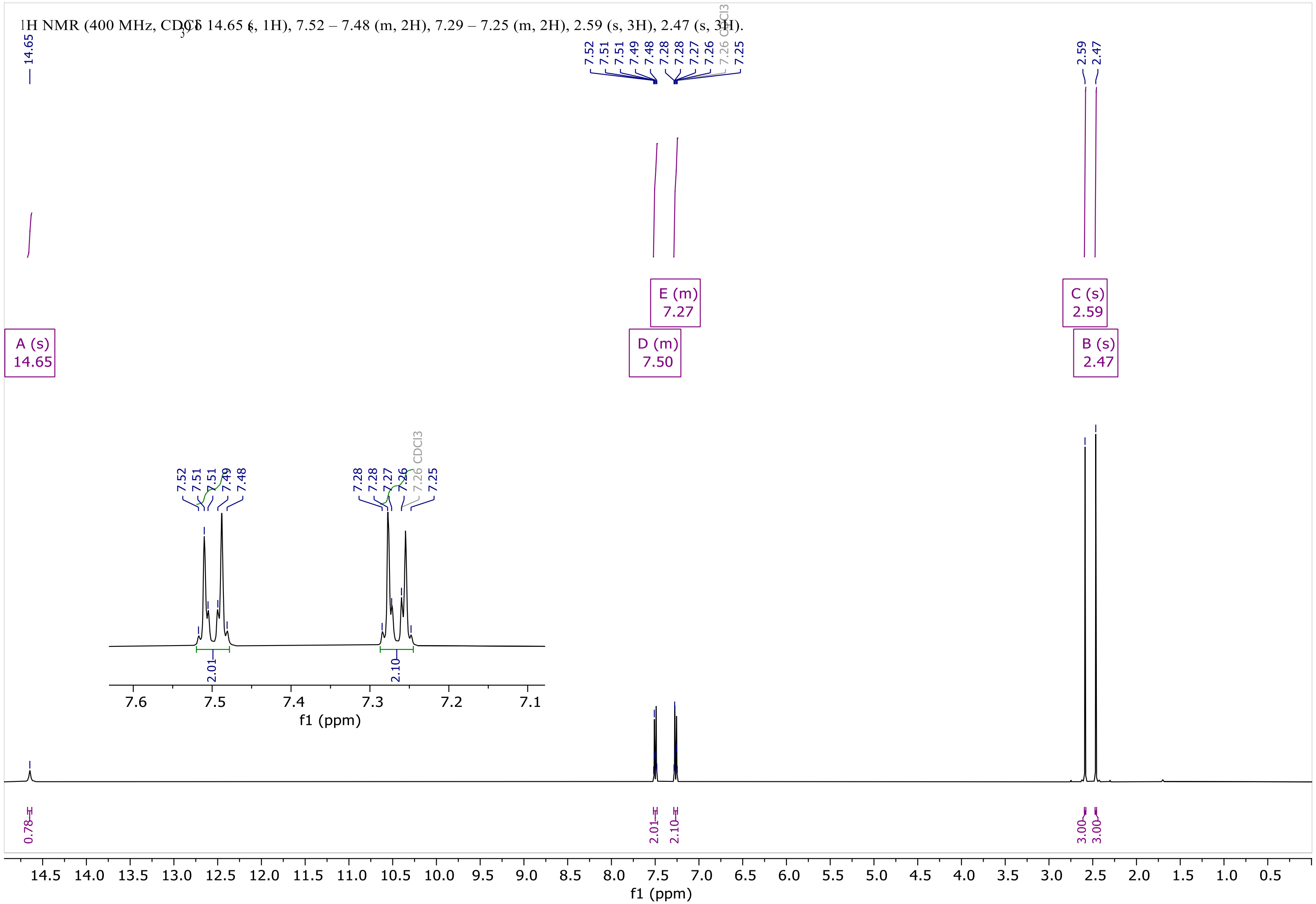

Figure S130: ^{1}H NMR spectrum of 3-(2-(4-bromophenyl)hydrazono)pentane-2,4-dione in $CDCl_3$.

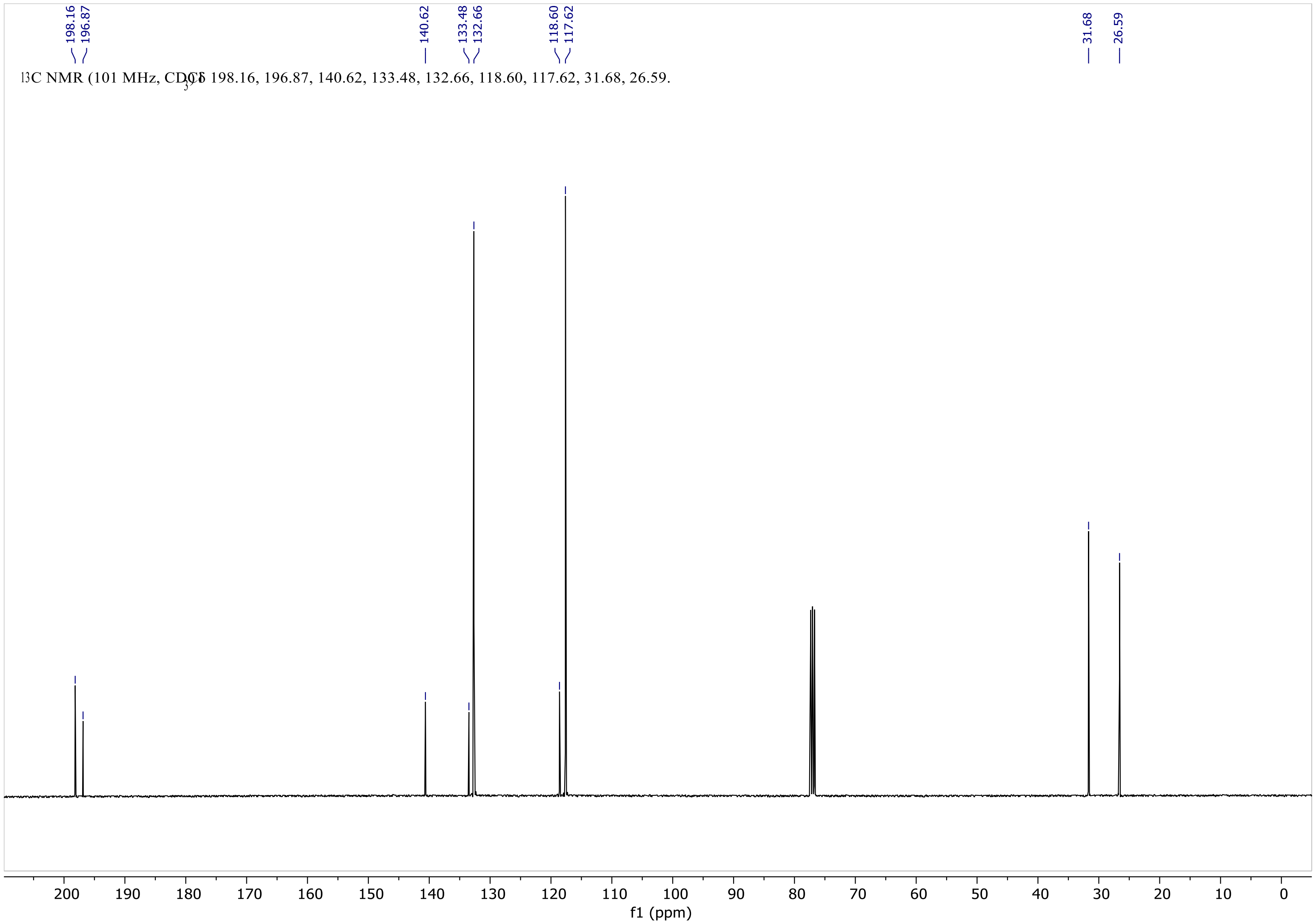

Figure S131: ^{13}C NMR spectrum of 3-(2-(4-bromophenyl)hydrazono)pentane-2,4-dione in $CDCl_3$.

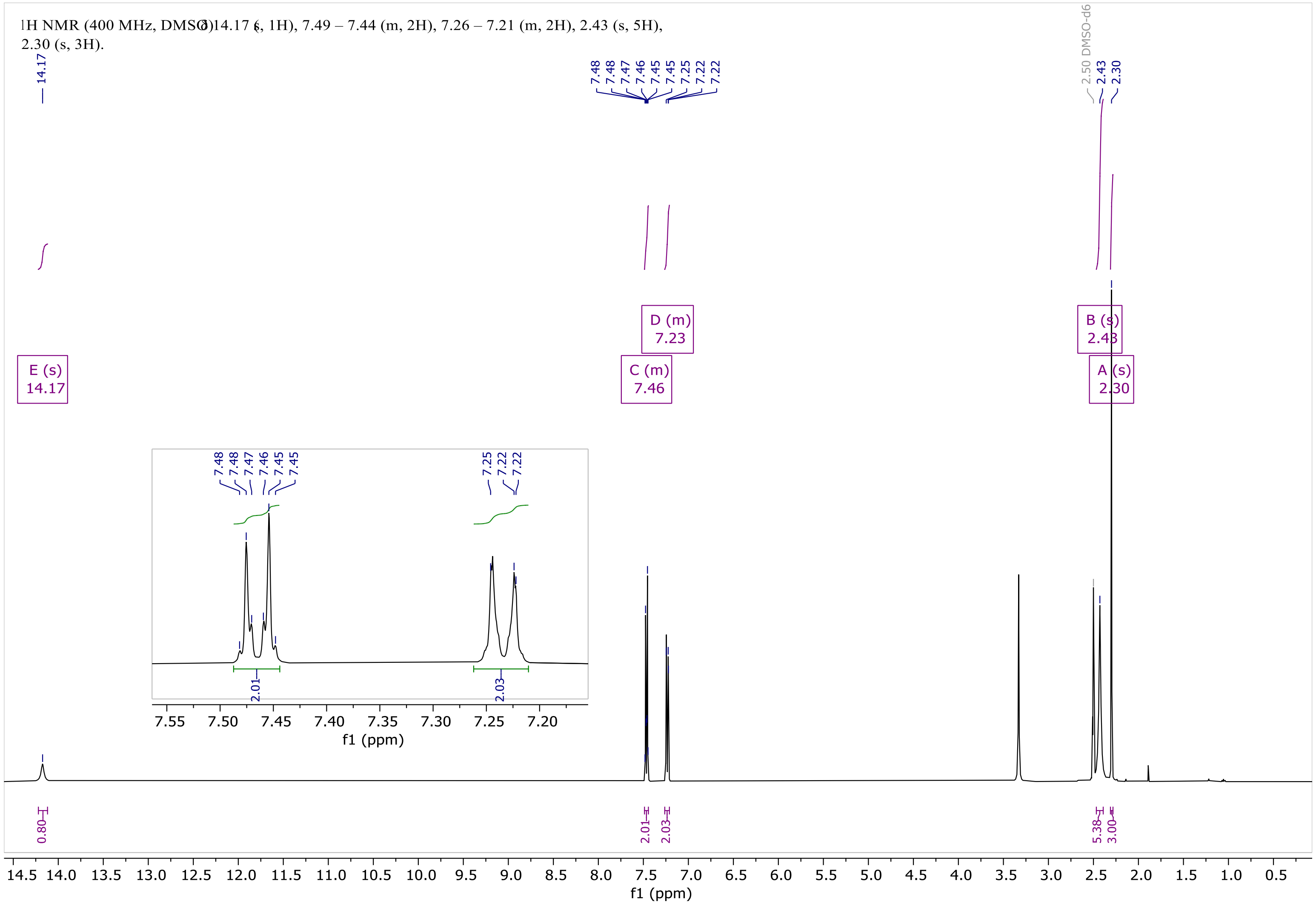

Figure S132: ^{1}H NMR spectrum of 3-(2-(*p*-tolyl)hydrazono)pentane-2,4-dione in DMSO-d_6.

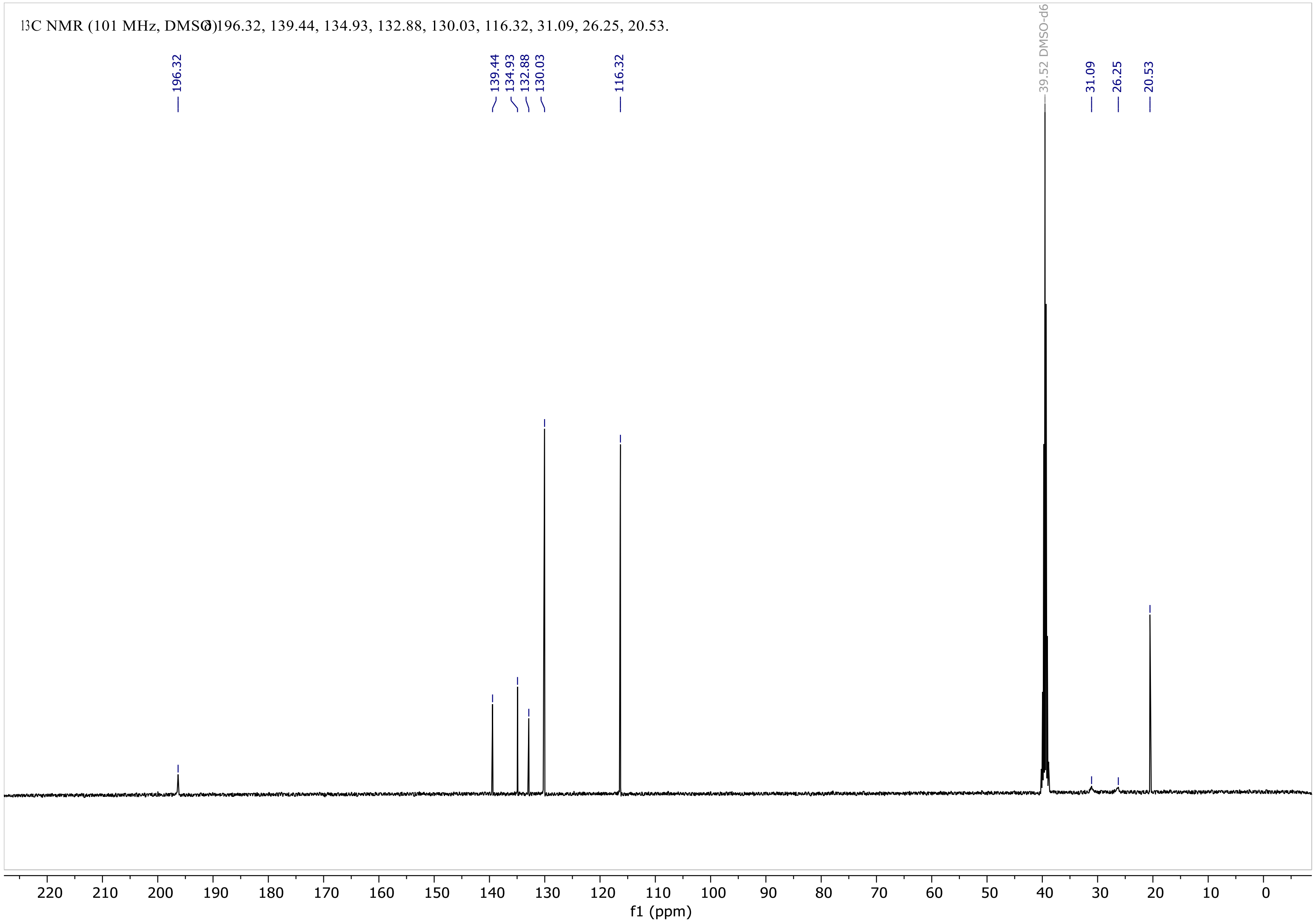

Figure S133: ^{13}C NMR spectrum of 3-(2-(*p*-tolyl)hydrazono)pentane-2,4-dione in DMSO-d_6.

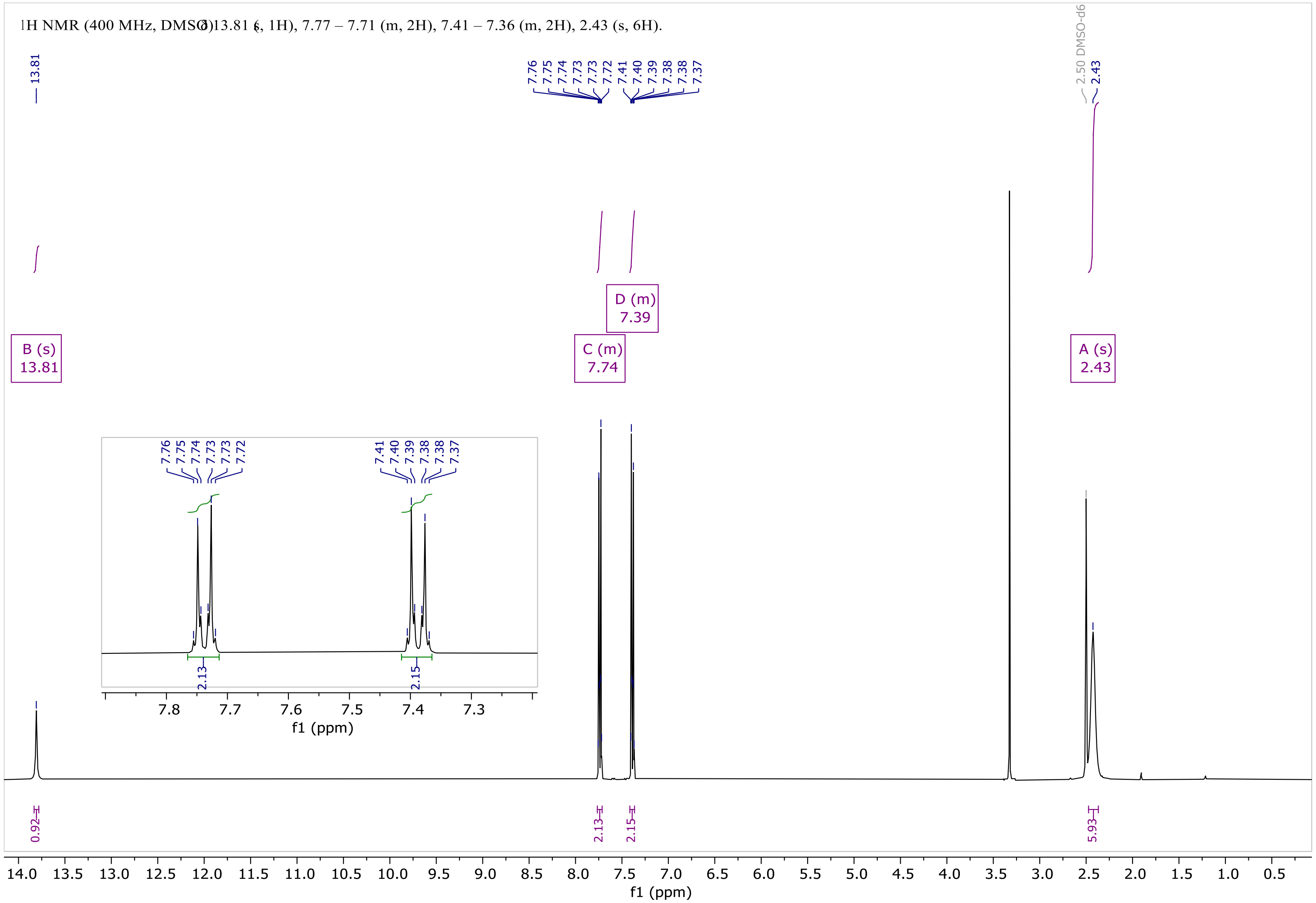

Figure S134: ^{1}H NMR spectrum of 3-(2-(4-iodophenyl)hydrazono)pentane-2,4-dione in DMSO-d_6.

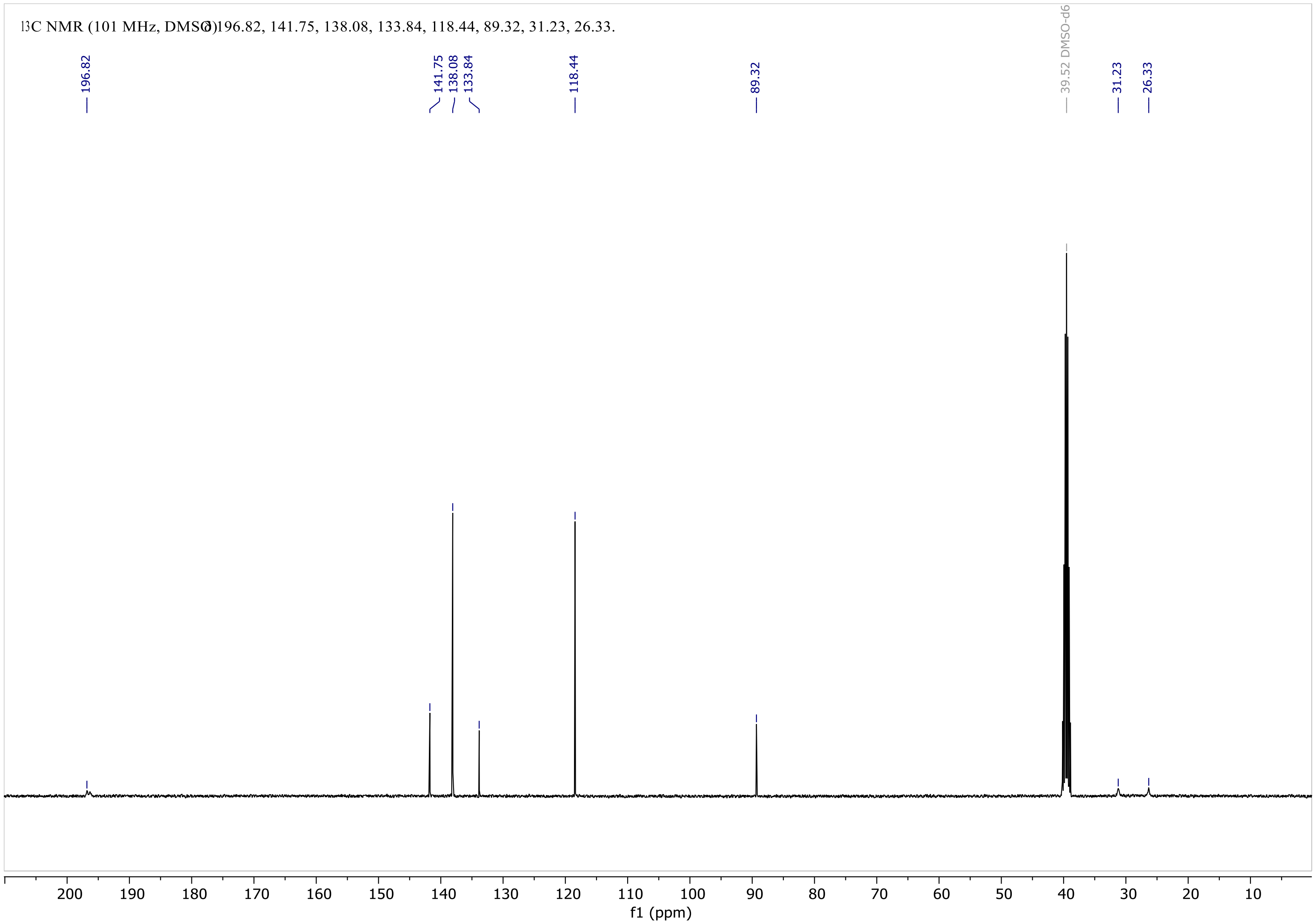

Figure S135: ^{13}C NMR spectrum of 3-(2-(4-iodophenyl)hydrazono)pentane-2,4-dione in DMSO-d_6.

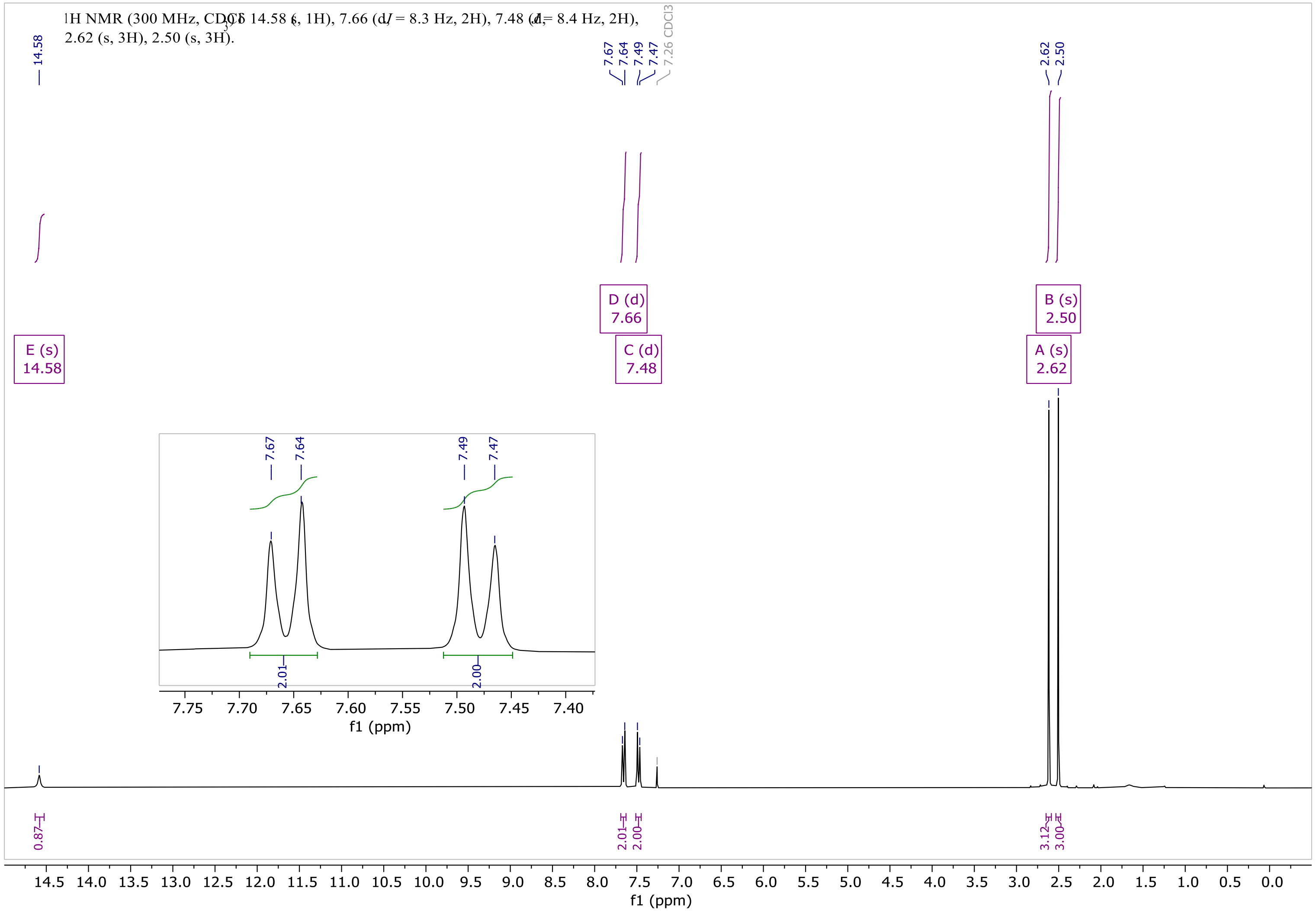

Figure S136: ^{1}H NMR spectrum of 3-(2-(4-(trifluoromethyl)phenyl)hydrazono)pentane-2,4-dione in $CDCl_3$.

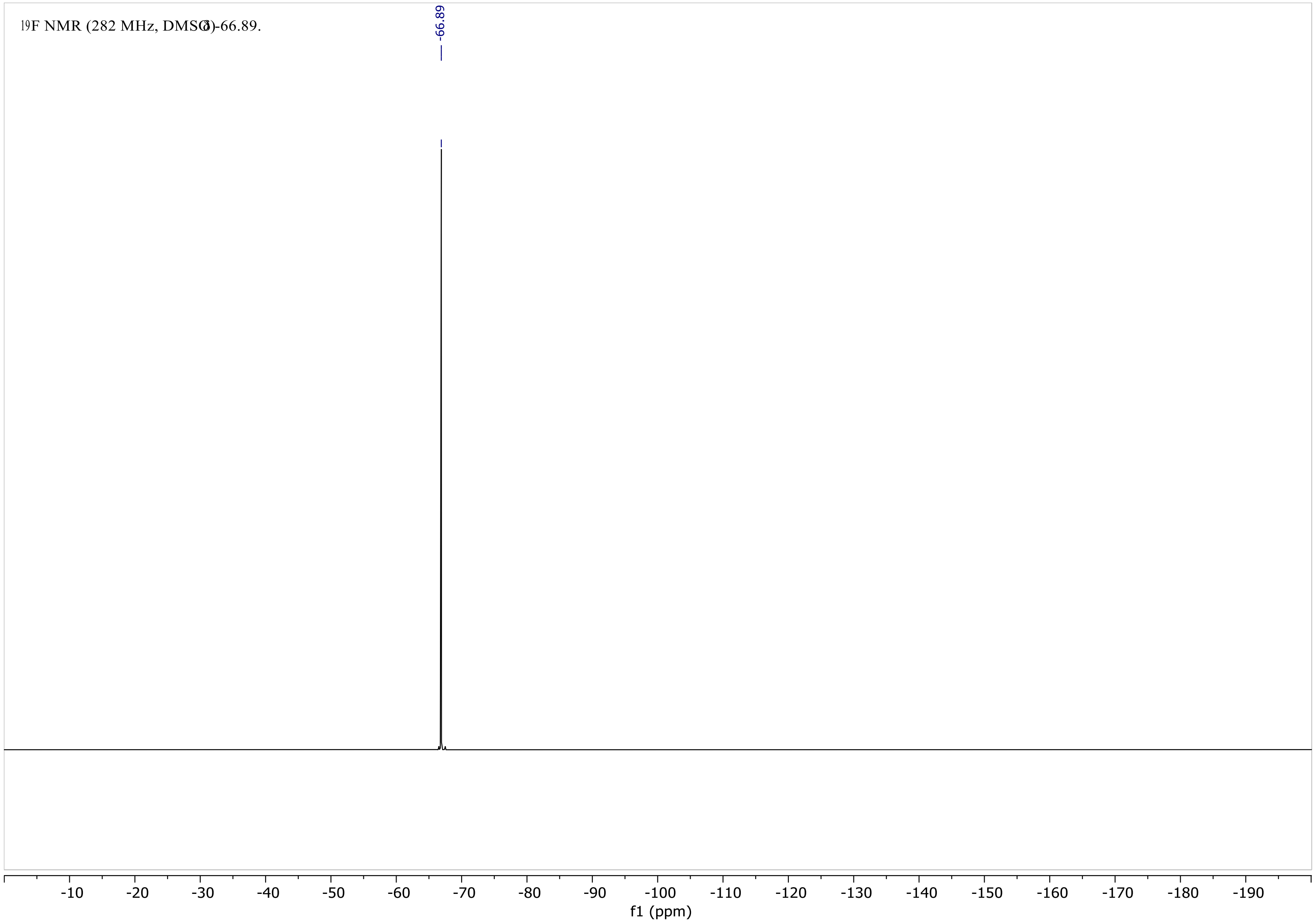

Figure S137: ^{19}F NMR spectrum of 3-(2-(4-(trifluoromethyl)phenyl)hydrazono)pentane-2,4-dione in DMSO-d_6.

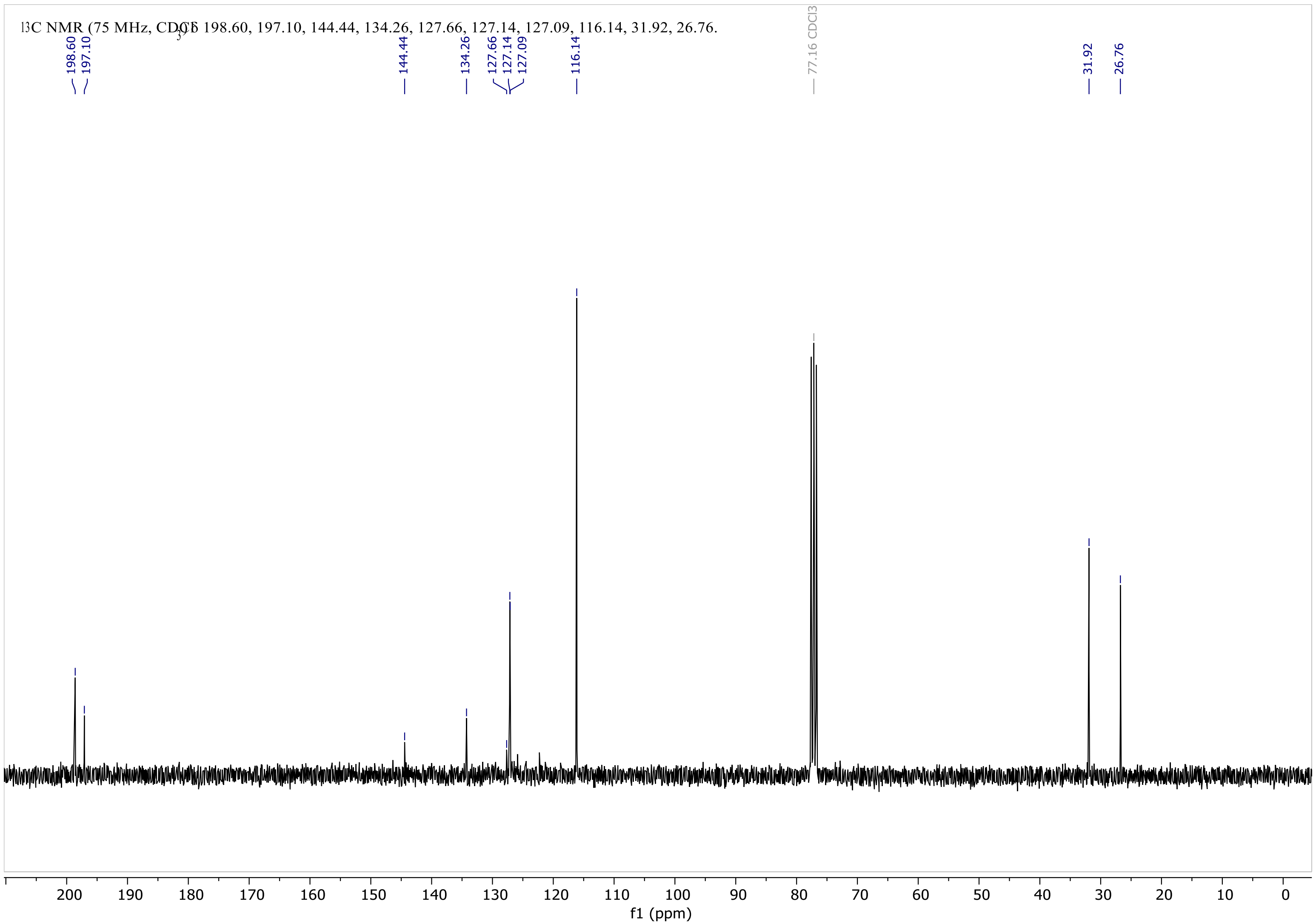

Figure S138: ^{13}C NMR spectrum of 3-(2-(4-(trifluoromethyl)phenyl)hydrazono)pentane-2,4-dione in $CDCl_3$.

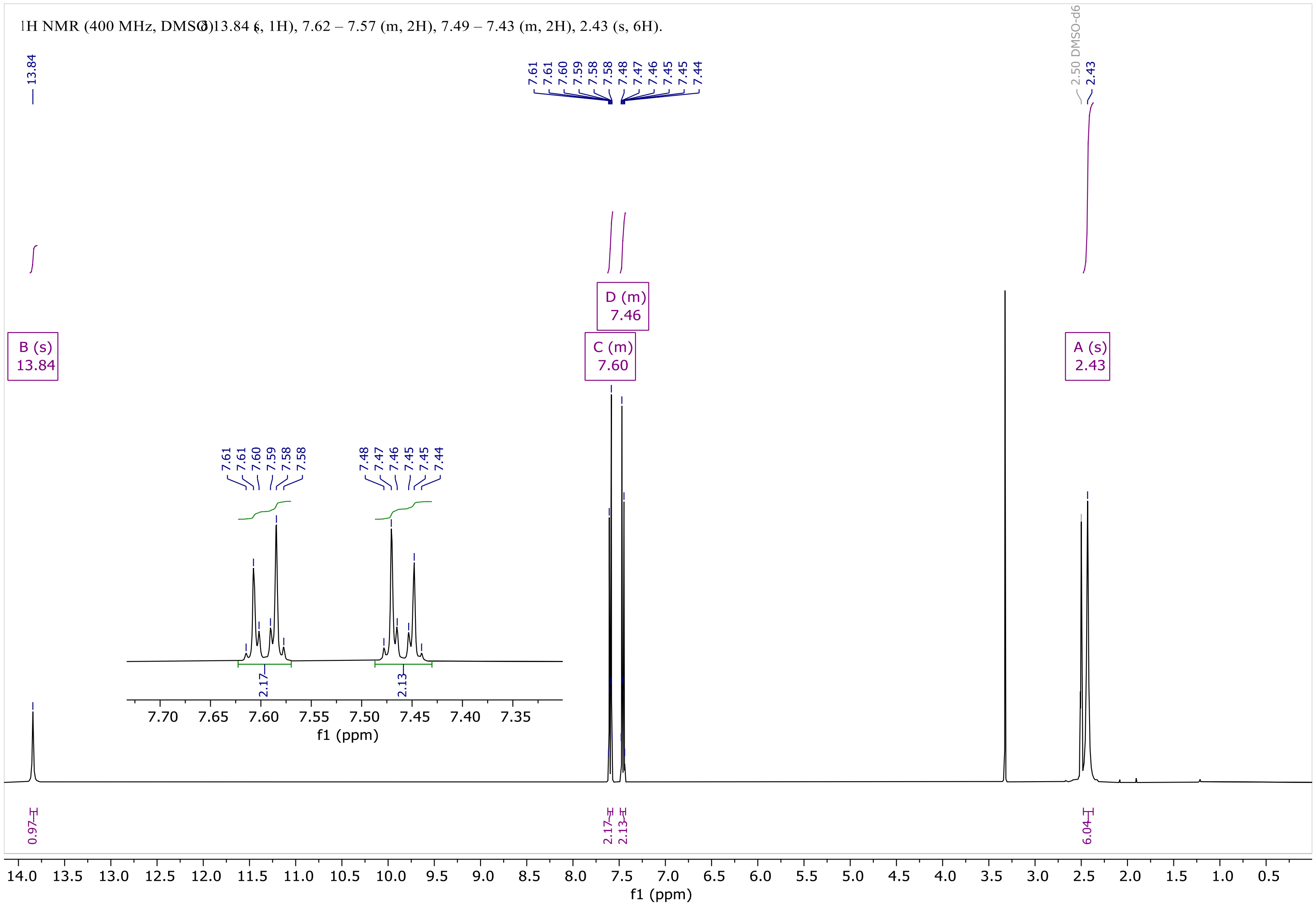

Figure S139: ^{1}H NMR spectrum of 3-(2-(4-chlorophenyl)hydrazono)pentane-2,4-dione in DMSO-d_6.

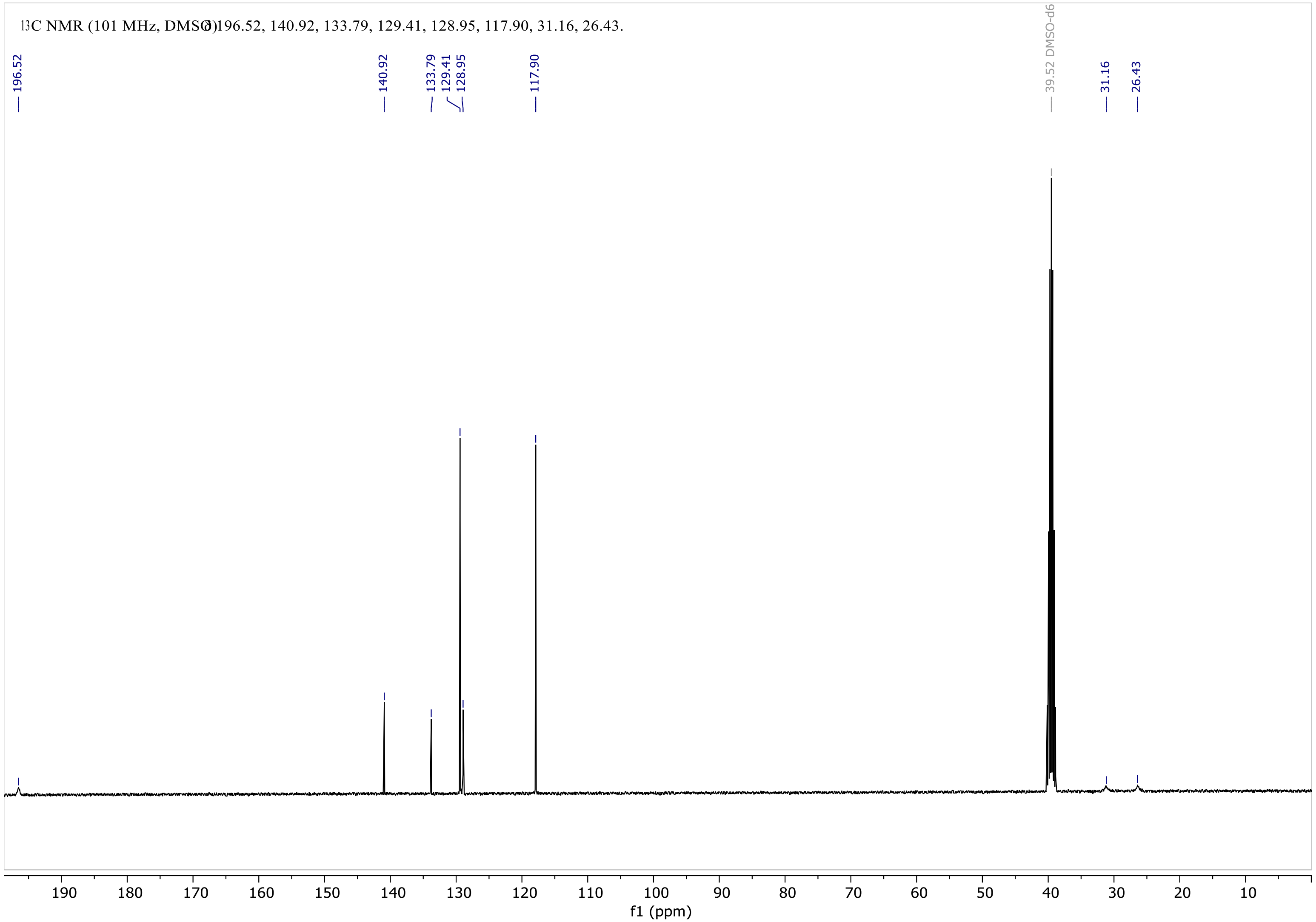

Figure S140: ^{13}C NMR spectrum of 3-(2-(4-chlorophenyl)hydrazono)pentane-2,4-dione in DMSO-d_6.

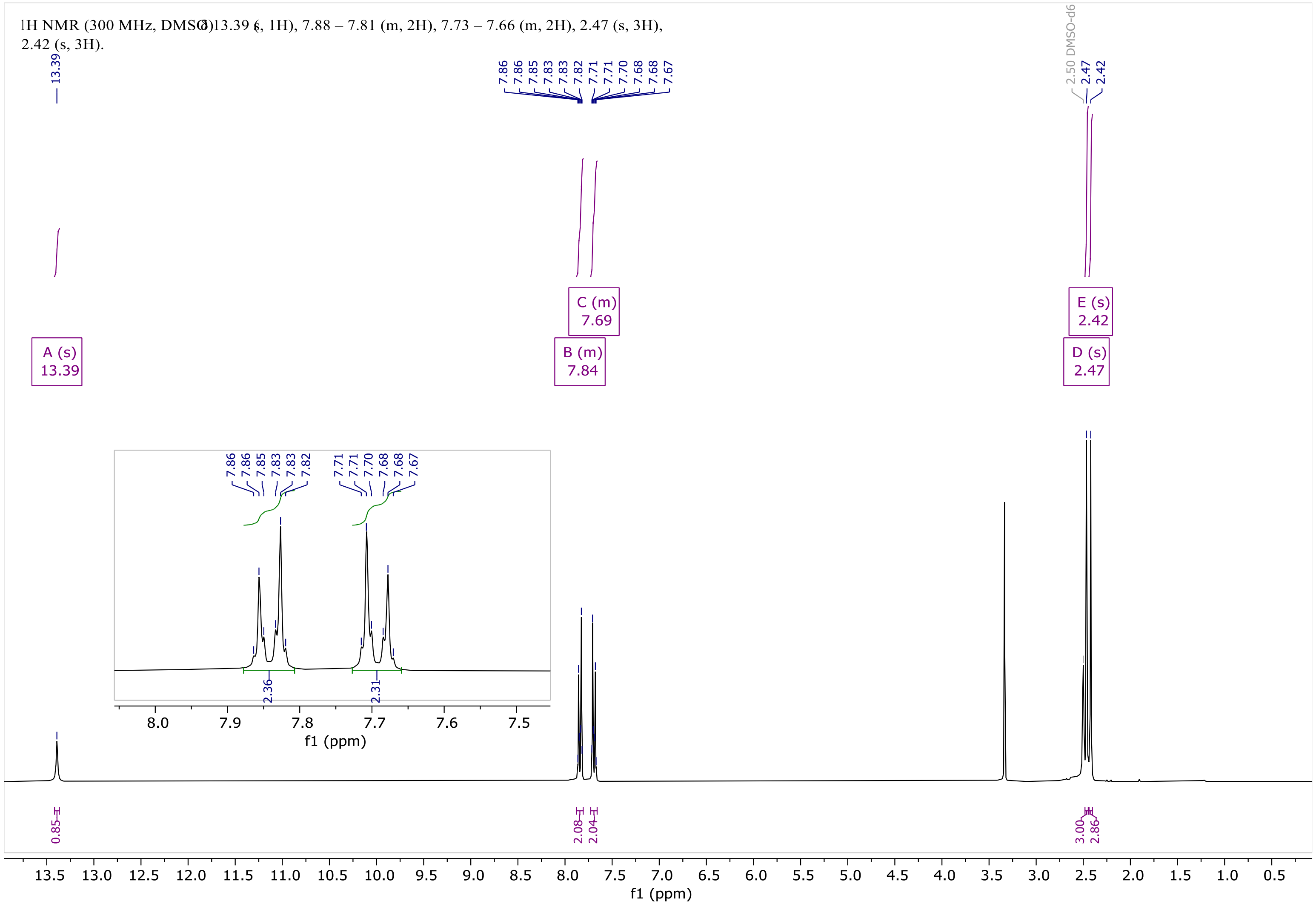

Figure S141: ^{1}H NMR spectrum of 4-(2-(2,4-dioxopentan-3-ylidene)hydrazineyl)benzonitrile in DMSO-d_6.

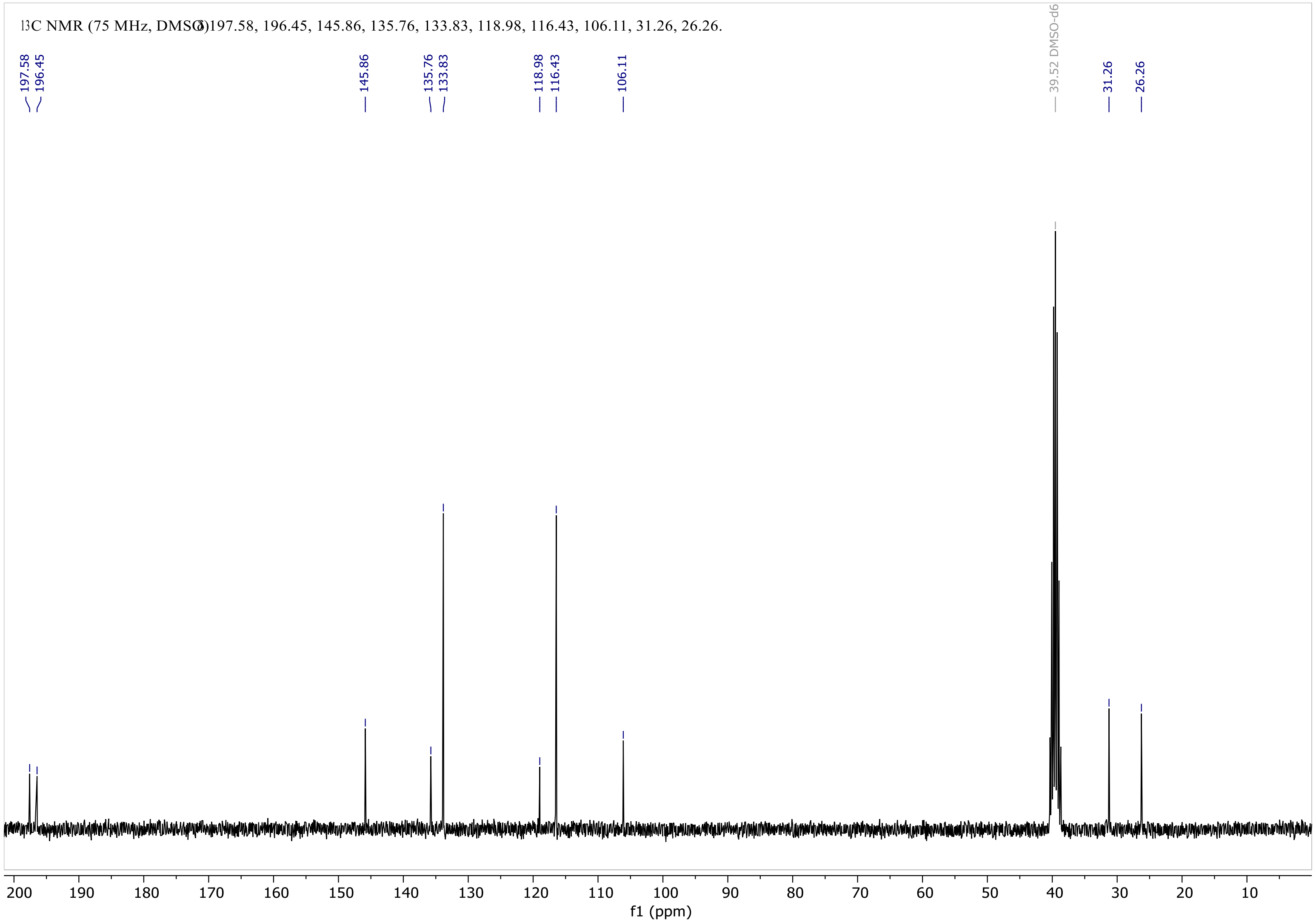

Figure S142: ^{13}C NMR spectrum of 4-(2-(2,4-dioxopentan-3-ylidene)hydrazineyl)benzonitrile in DMSO-d_6.

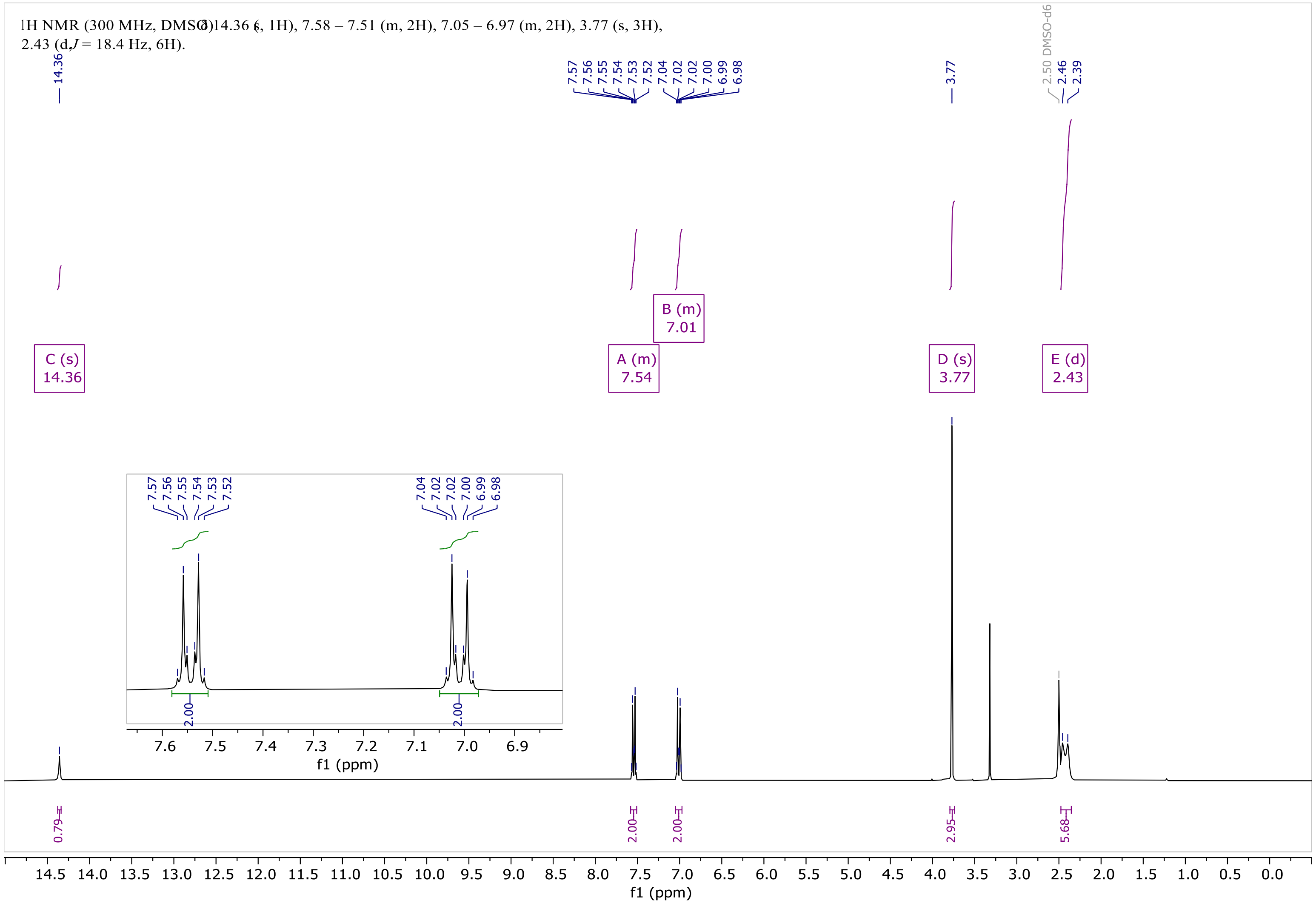

Figure S143: ^{1}H NMR spectrum of 3-(2-(4-methoxyphenyl)hydrazono)pentane-2,4-dione in DMSO-d_6.

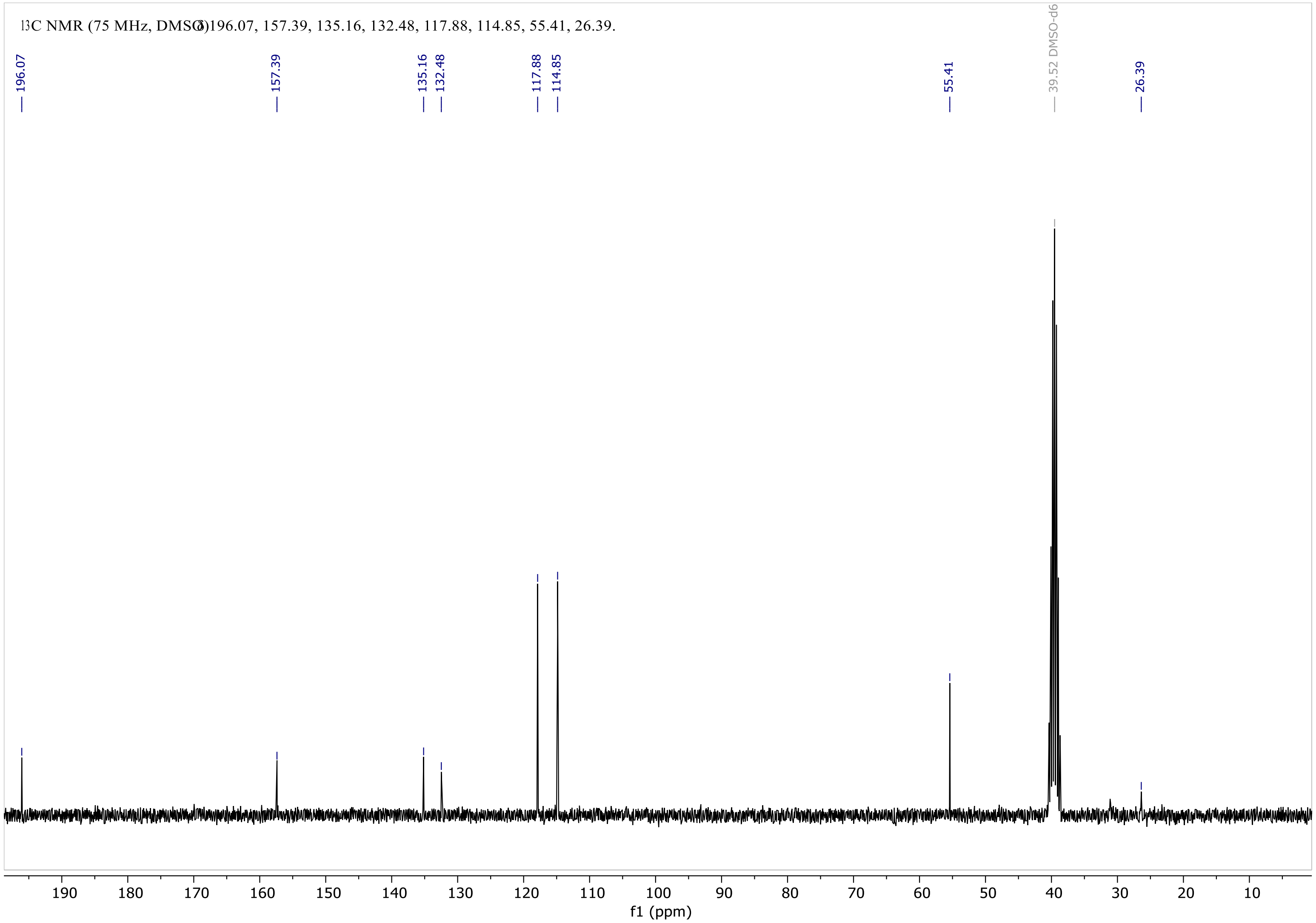

Figure S144: ^{13}C NMR spectrum of 3-(2-(4-methoxyphenyl)hydrazono)pentane-2,4-dione in DMSO-d_6.

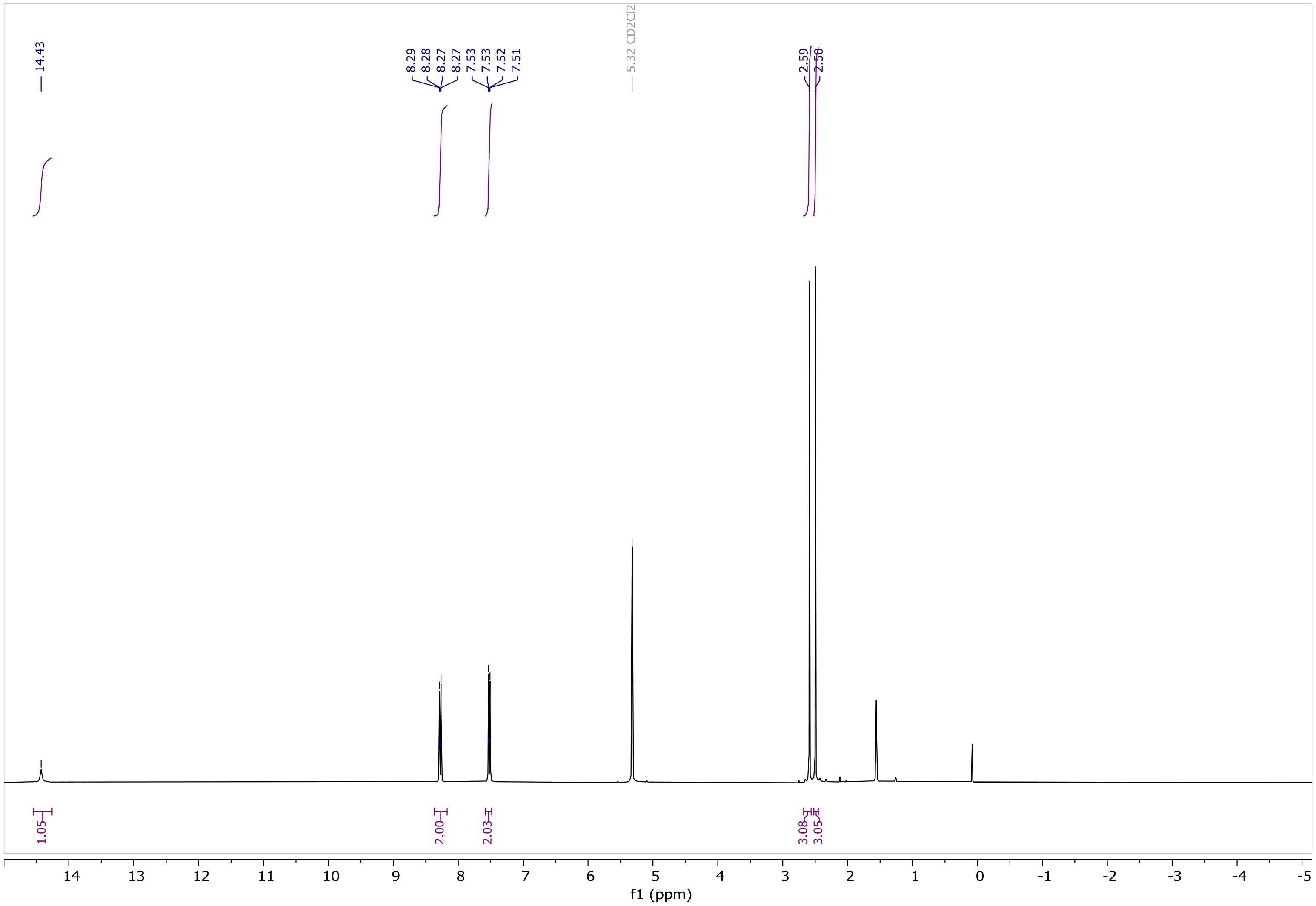

Figure S145: ^{1}H NMR spectrum of 3-(2-(4-nitrophenyl)hydrazono)pentane-2,4-dione in CD_2Cl_2.

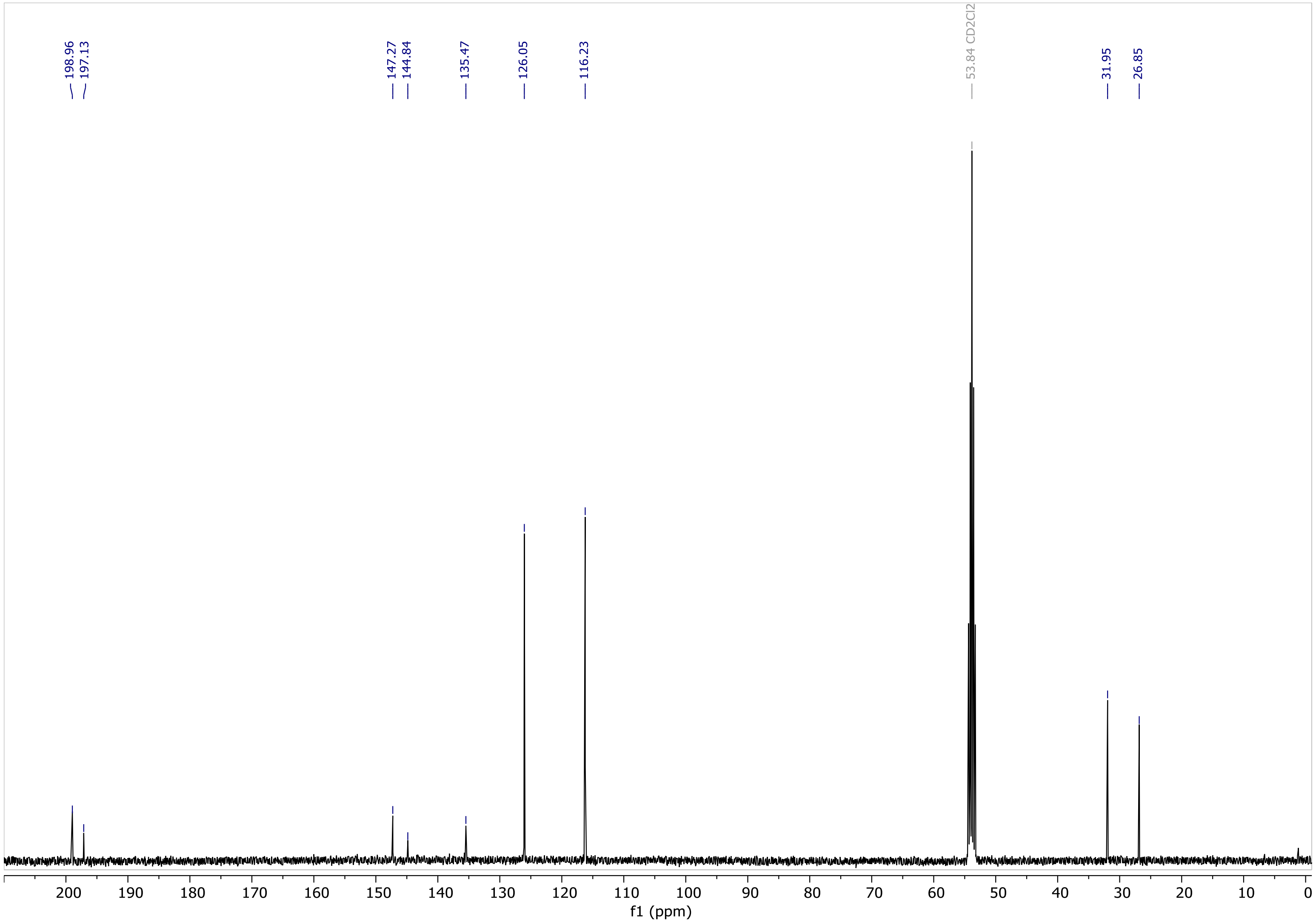

Figure S146: ^{13}C NMR spectrum of 3-(2-(4-nitrophenyl)hydrazono)pentane-2,4-dione in CD_2Cl_2.

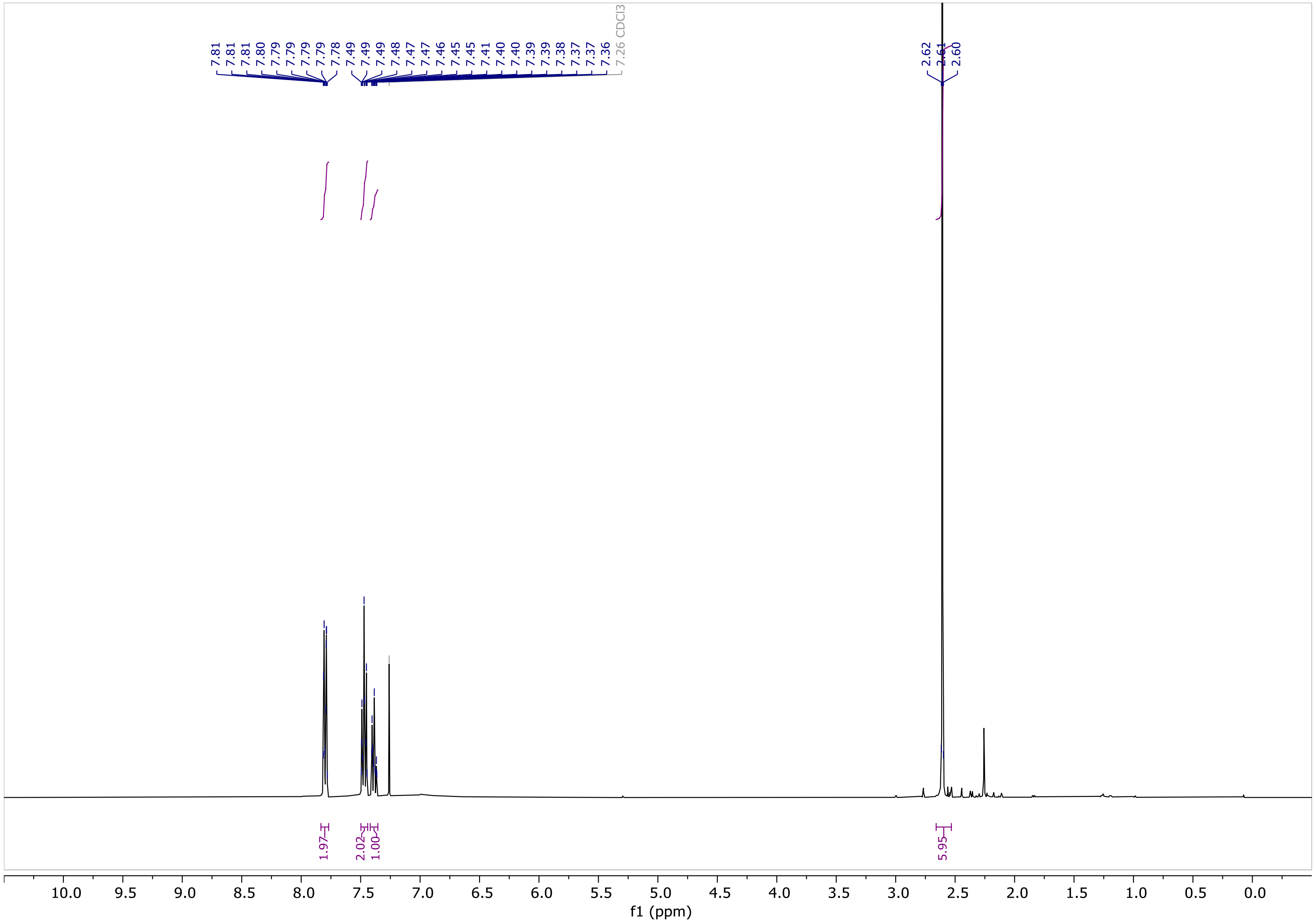

Figure S147: ^{1}H NMR spectrum of (*E*)-3,5-dimethyl-4-(phenyldiazenyl)-1*H*-pyrazole in $CDCl_3$.

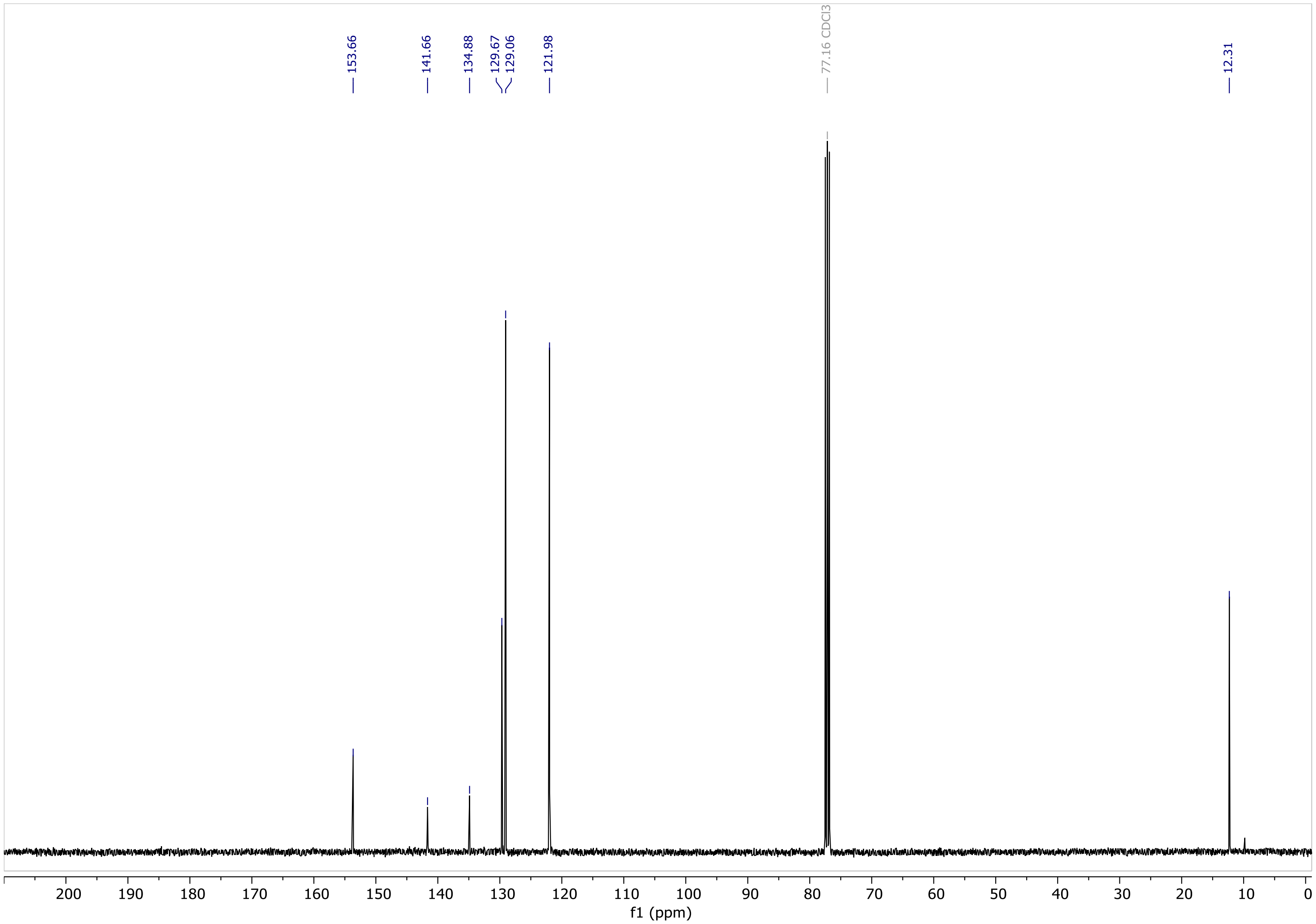

Figure S148: ^{13}C NMR spectrum of (*E*)-3,5-dimethyl-4-(phenyldiazenyl)-1*H*-pyrazole in $CDCl_3$.

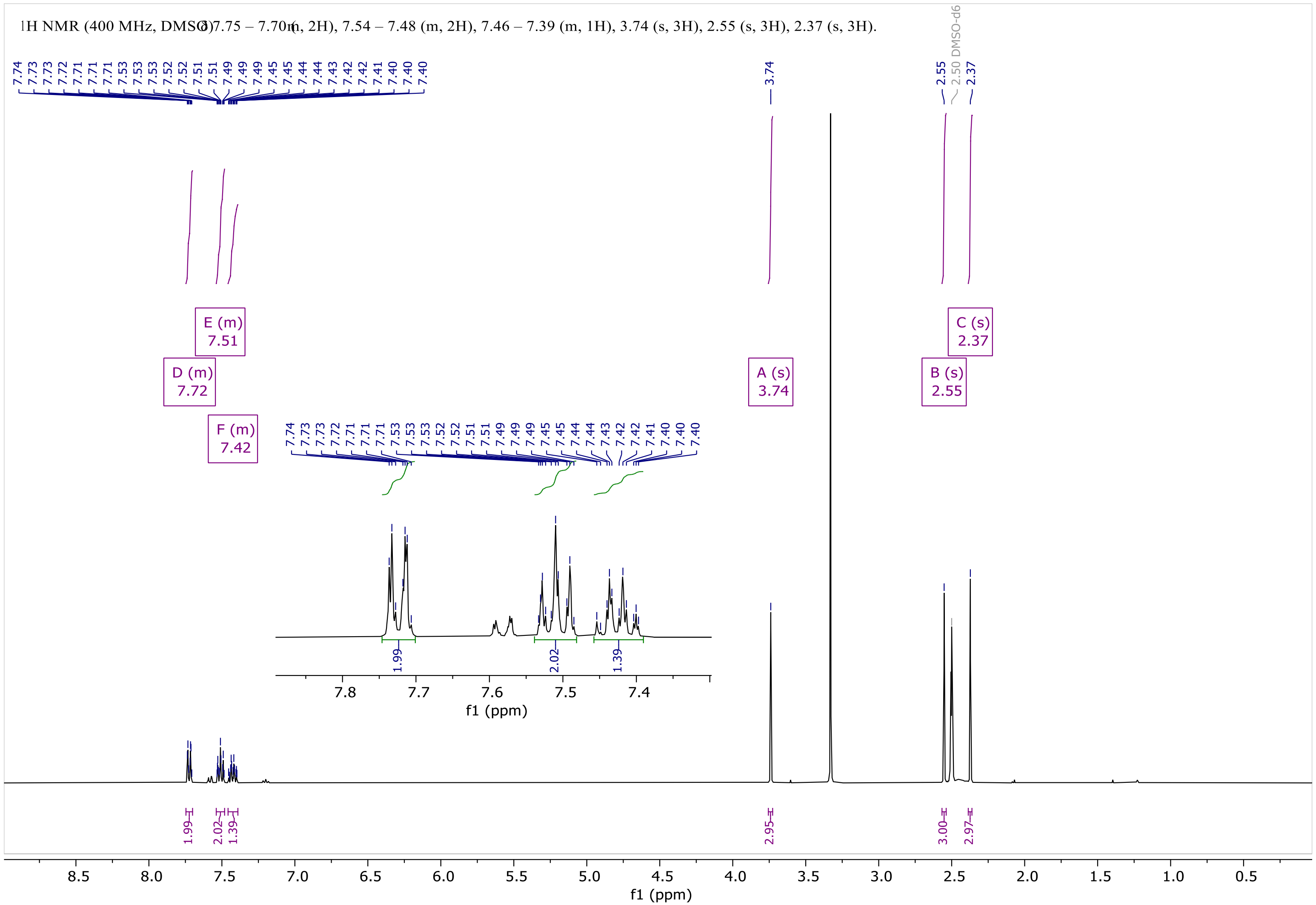

Figure S149: ^{1}H NMR spectrum of (*E*)-1,3,5-trimethyl-4-(phenyldiazenyl)-1*H*-pyrazole in DMSO-d_6.

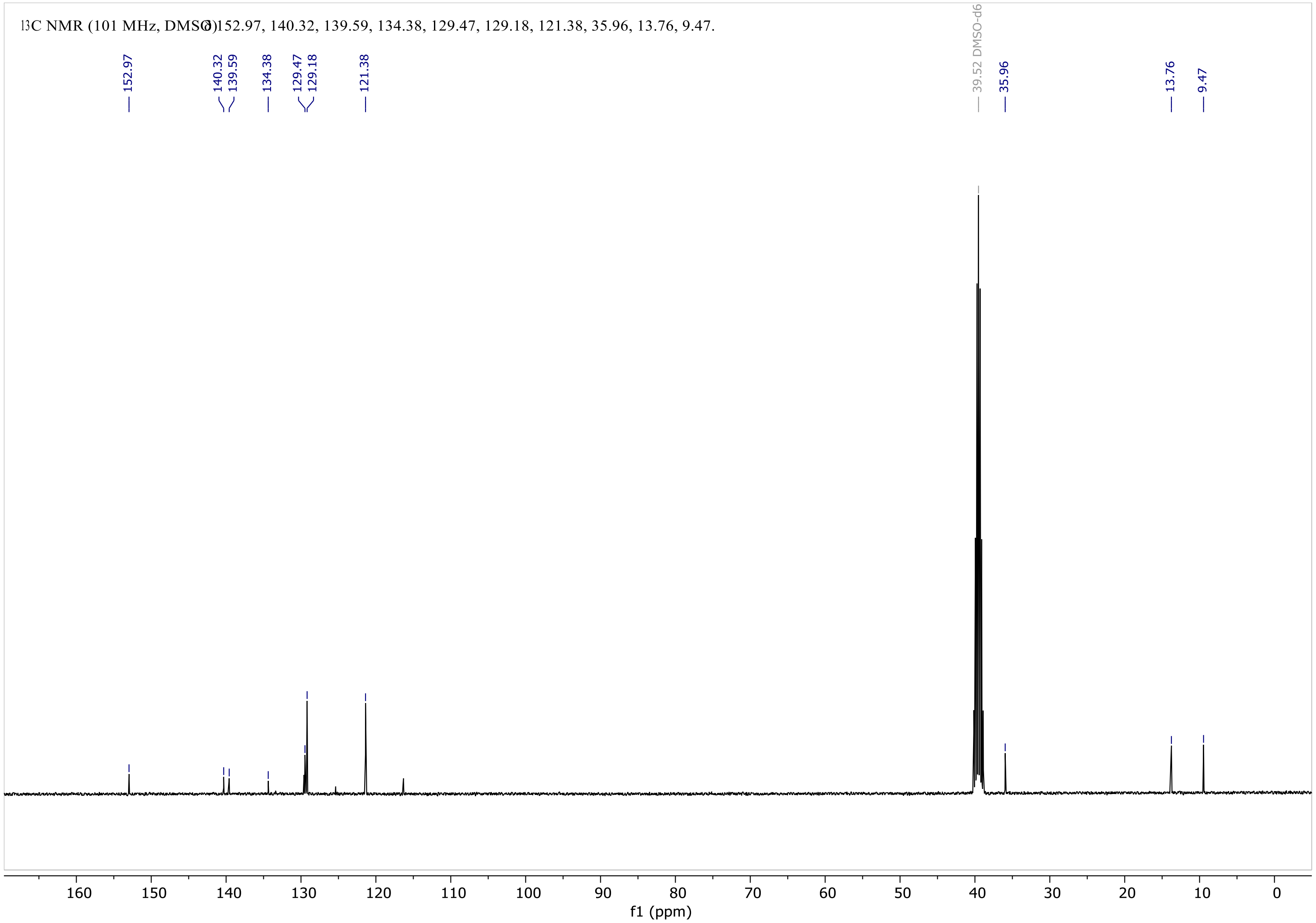

Figure S150: ^{13}C NMR spectrum of (*E*)-1,3,5-trimethyl-4-(phenyldiazenyl)-1*H*-pyrazole in DMSO-d_6.

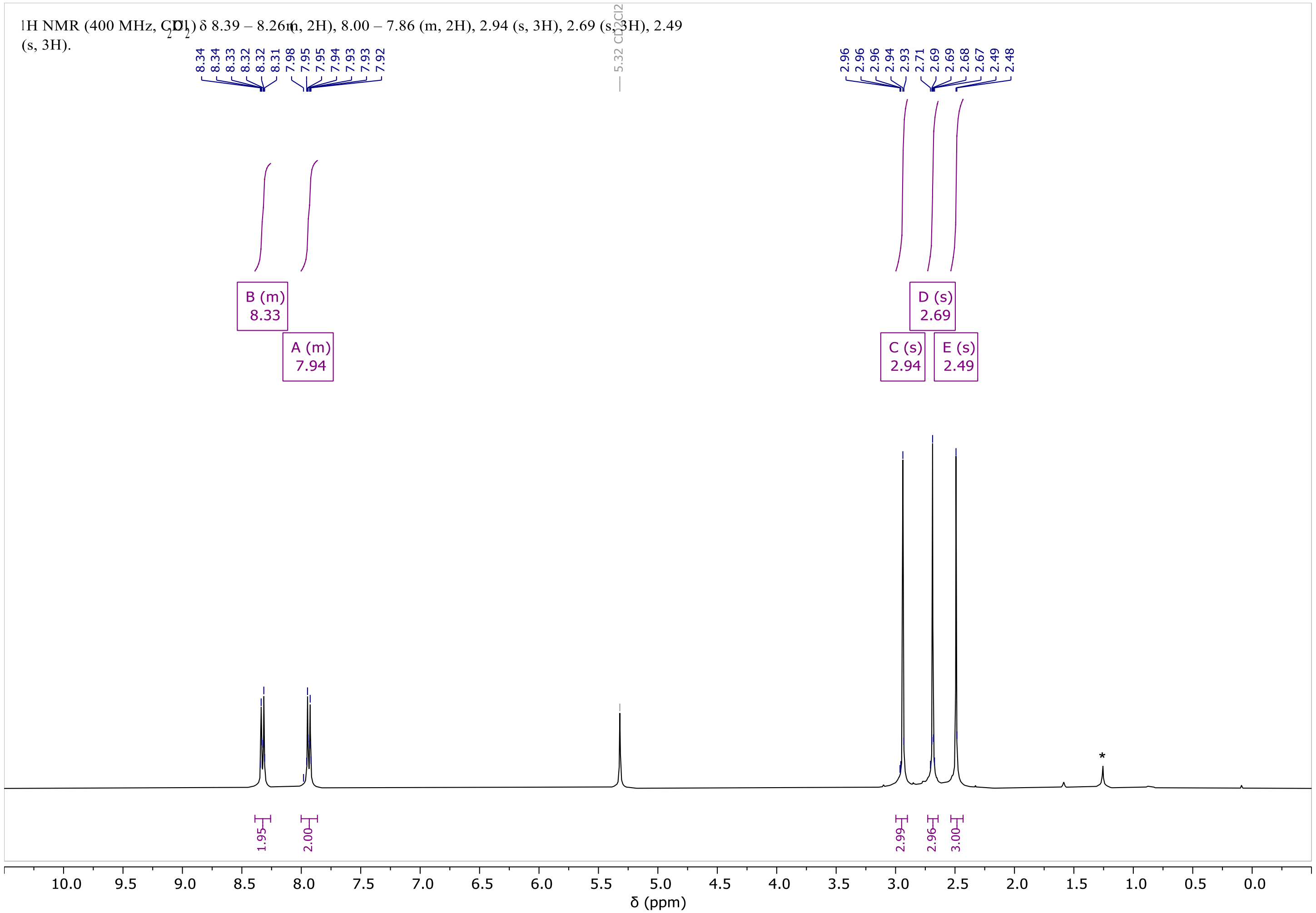

Figure S151: ^{1}H NMR spectrum of (*E*)-4-((4-fluorophenyl)diazenyl)-3,5-dimethyl)-1*H*-pyrazole in CD_2Cl_2.

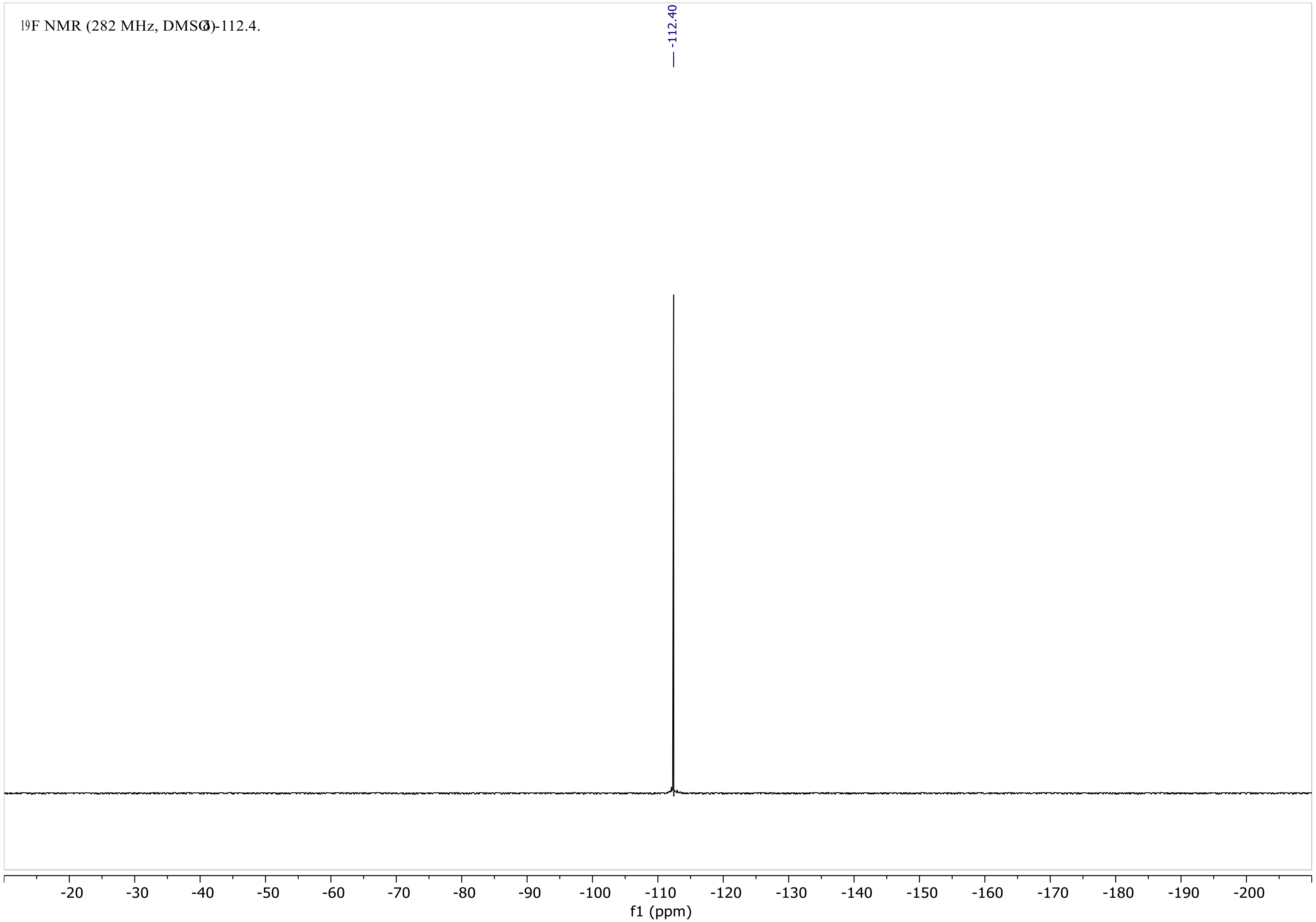

Figure S152: ^{19}F NMR spectrum of (*E*)-4-((4-fluorophenyl)diazenyl)-3,5-dimethyl-1*H*-pyrazole in DMSO-d_6.

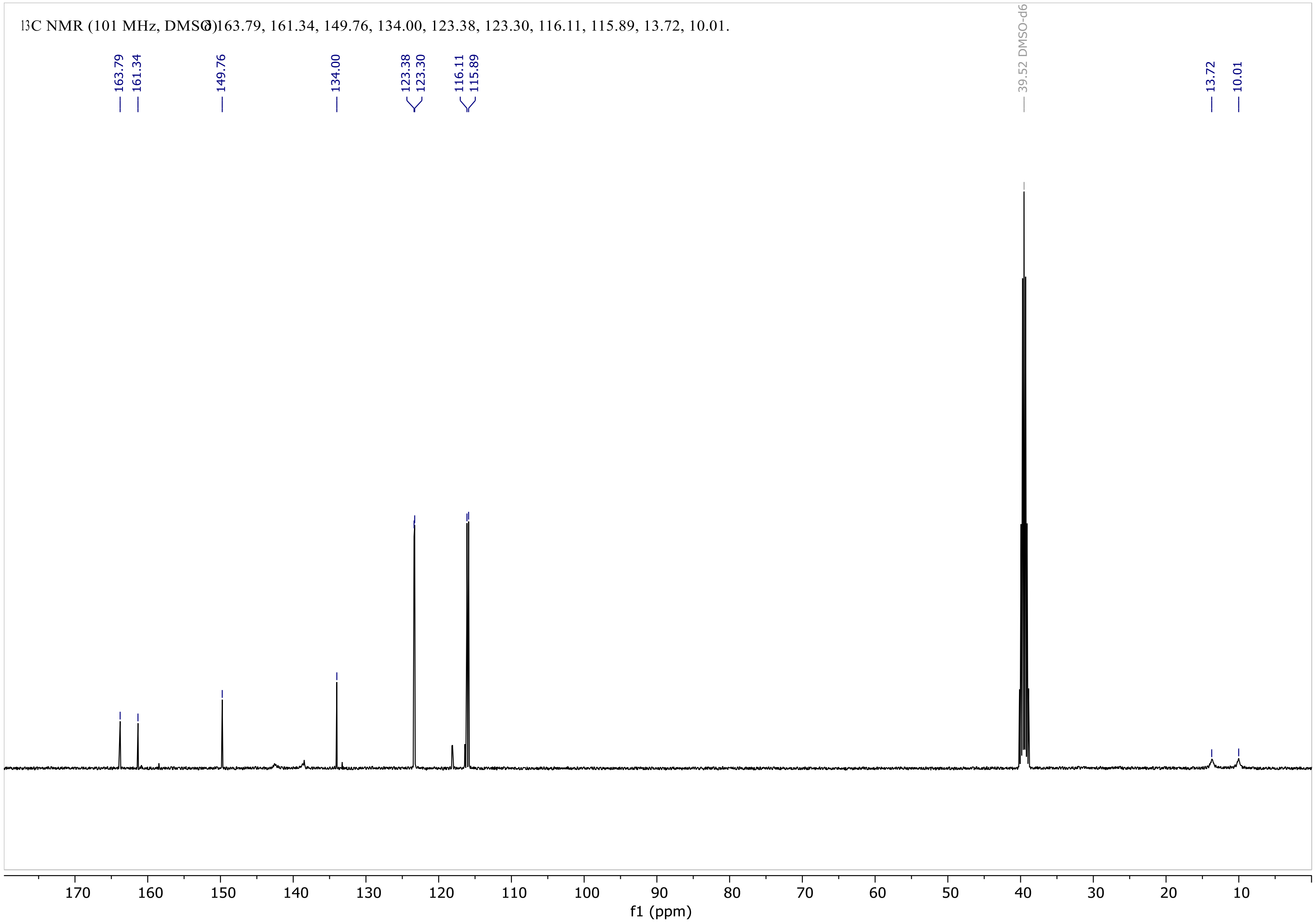

Figure S153: ^{13}C NMR spectrum of (*E*)-4-((4-fluorophenyl)diazenyl)-3,5-dimethyl)-1*H*-pyrazole in DMSO-*d*$_6$.

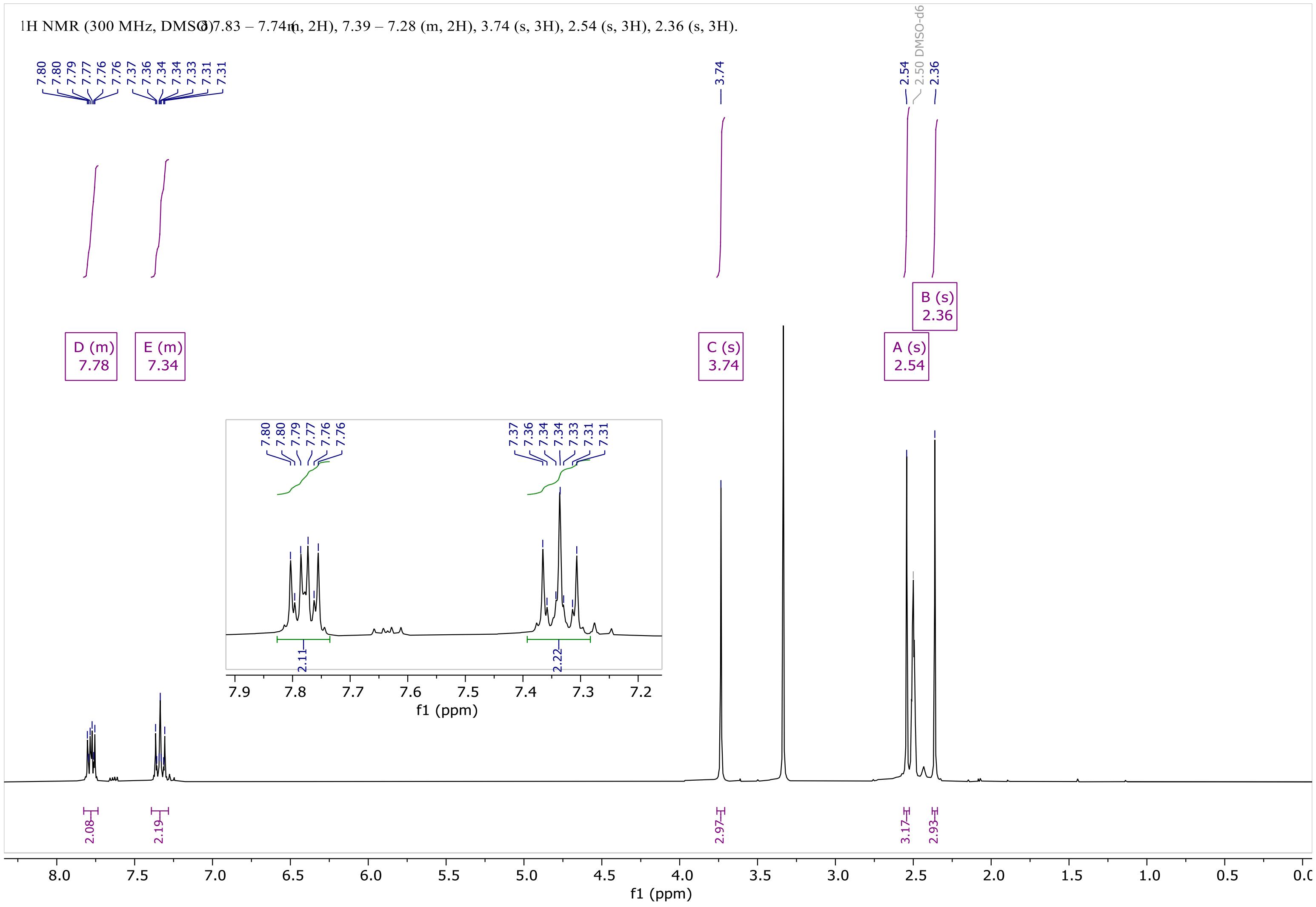

Figure S154: ^{1}H NMR spectrum of (*E*)-4-((4-fluorophenyl)diazenyl)-1,3,5-trimethyl-1*H*-pyrazole in DMSO-d_6.

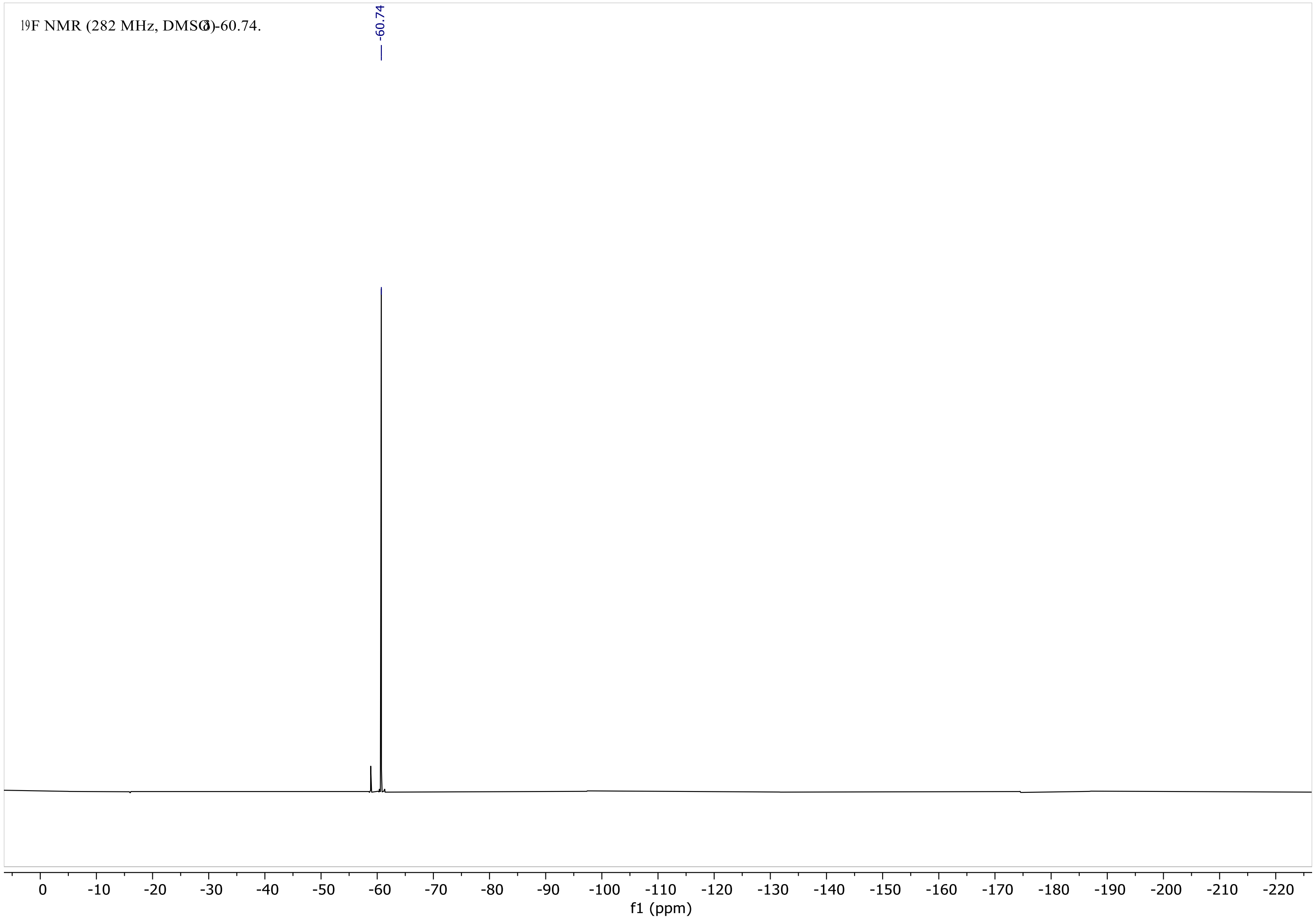

Figure S155: ^{19}F NMR spectrum of (*E*)-4-((4-fluorophenyl)diazenyl)-1,3,5-trimethyl-1*H*-pyrazole in DMSO-d_6.

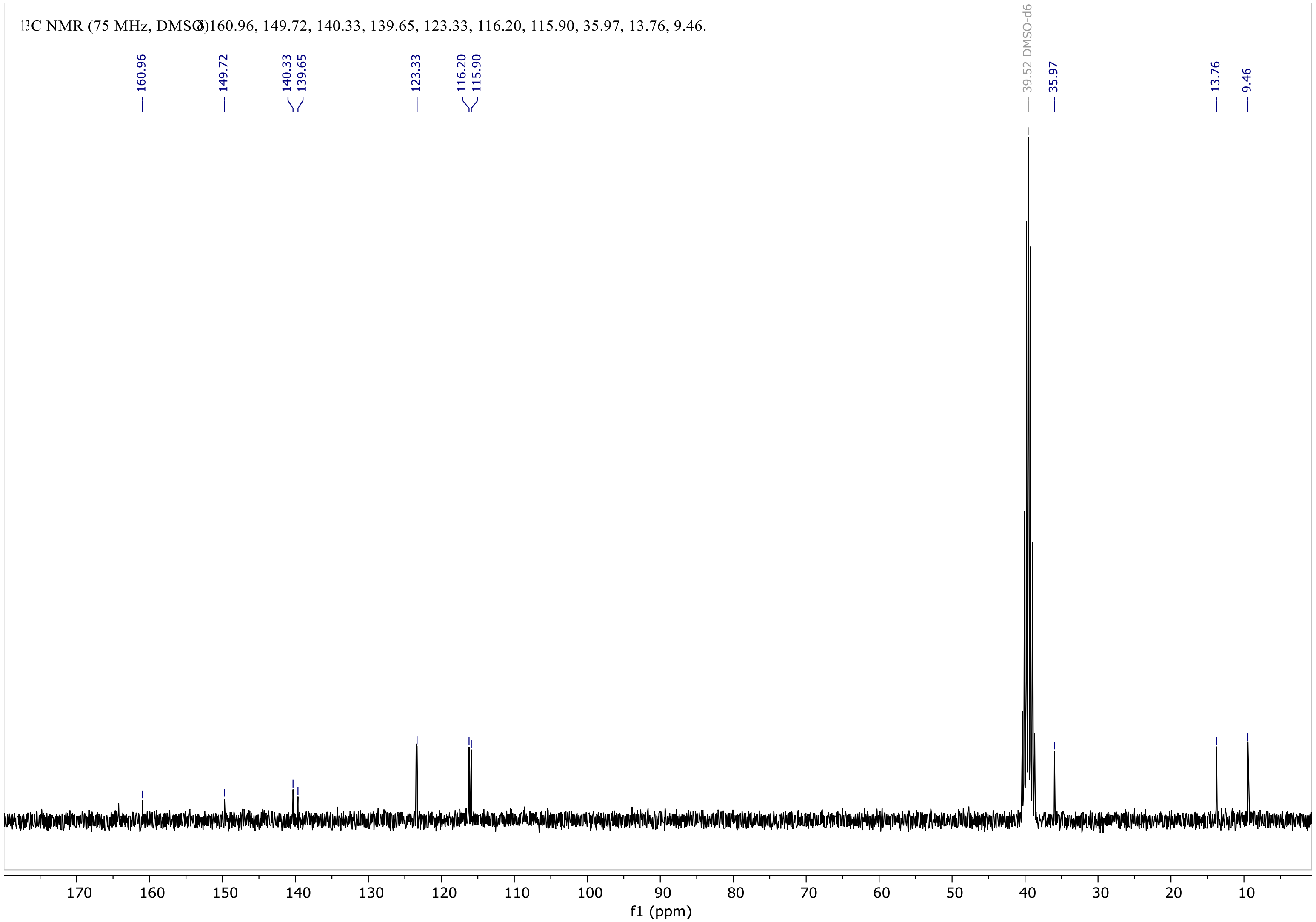

Figure S156: ^{13}C NMR spectrum of (*E*)-4-((4-fluorophenyl)diazenyl)-1,3,5-trimethyl-1*H*-pyrazole in DMSO-d_6.

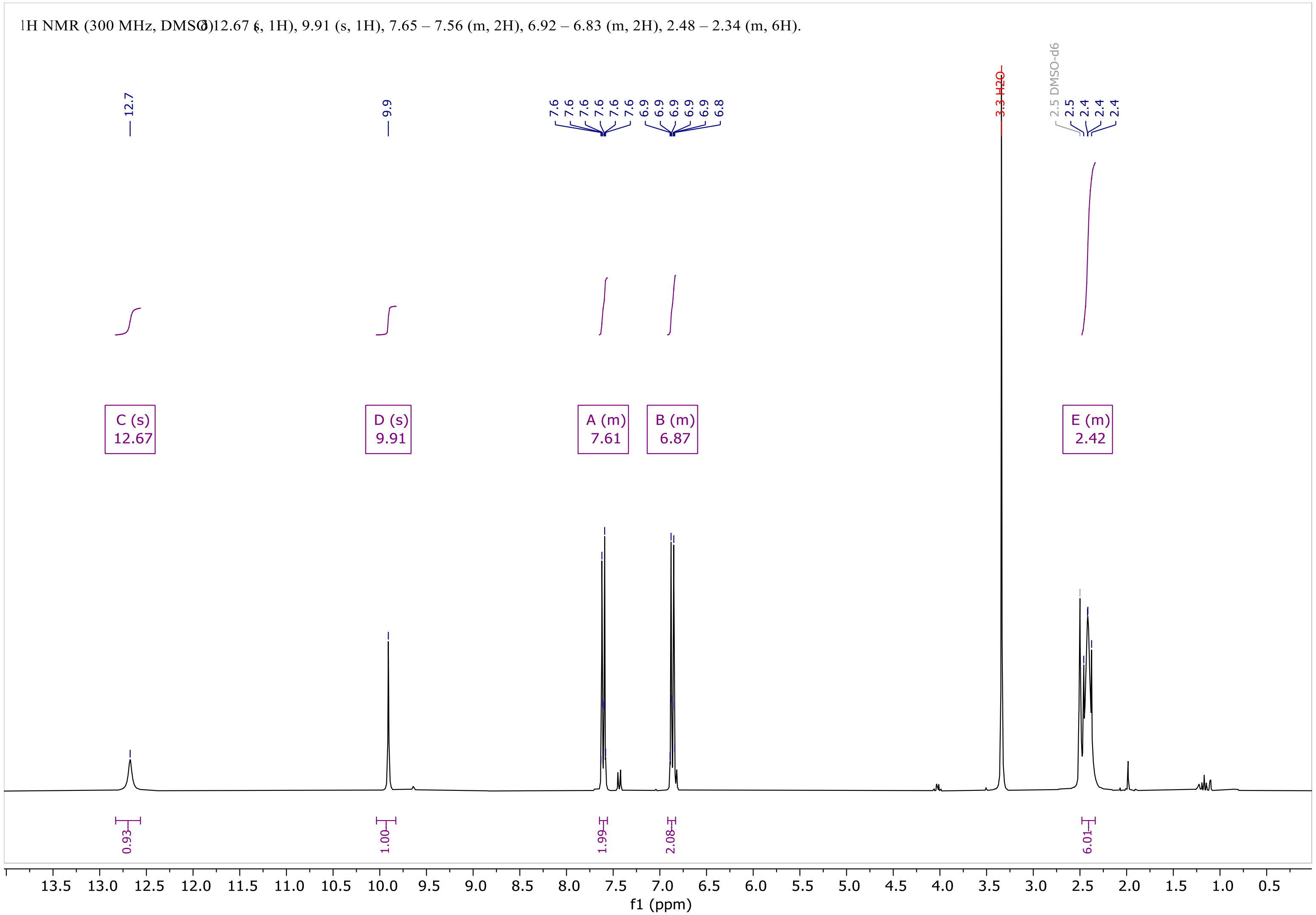

Figure S157: ^{1}H NMR spectrum of (*E*)-4-((3,5-dimethyl-1*H*-pyrazol-4-yl)diazenyl)phenol in DMSO-d_6.

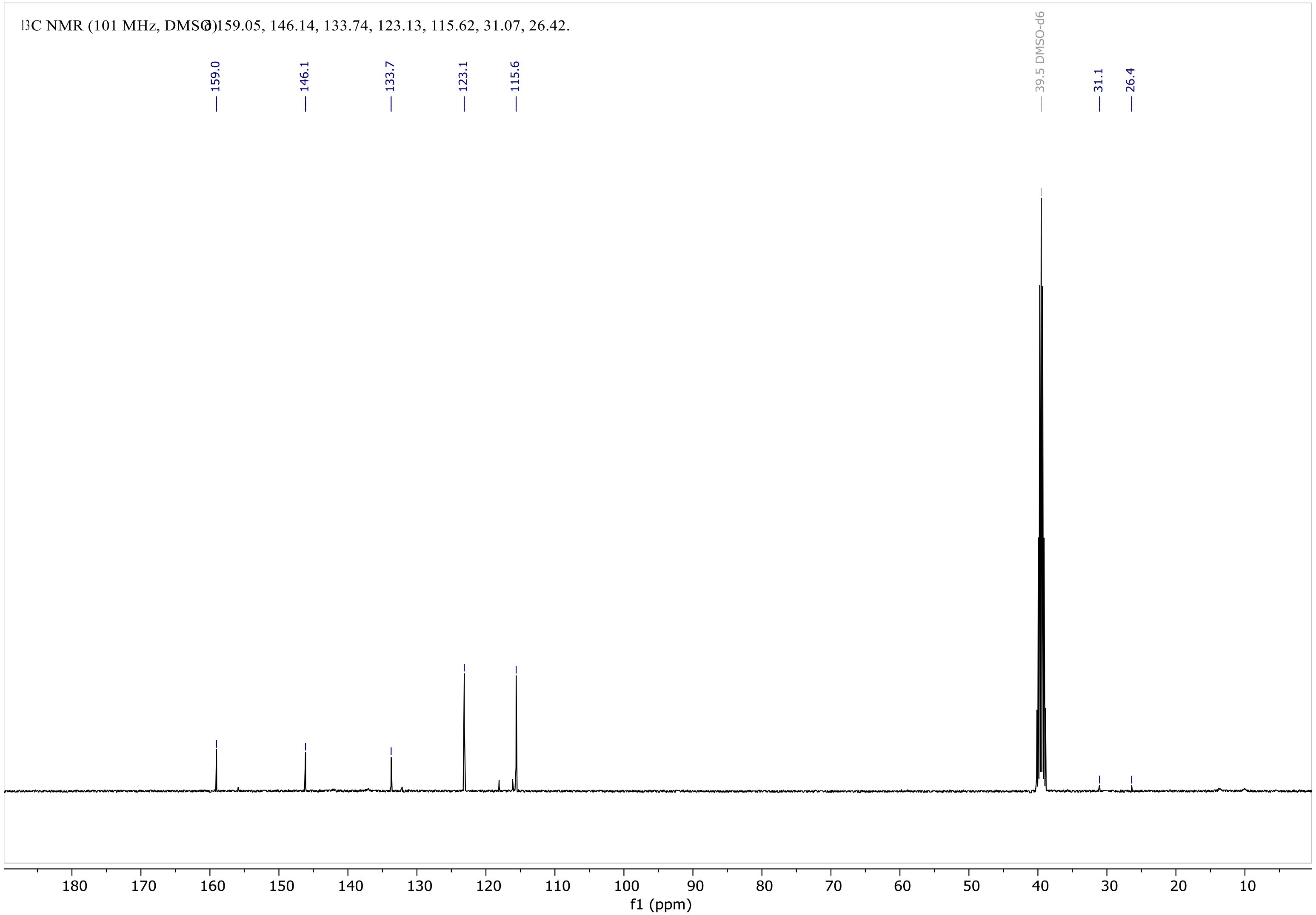

Figure S158: ^{13}C NMR spectrum of (*E*)-4-((3,5-dimethyl-1*H*-pyrazol-4-yl)diazenyl)phenol in DMSO-d_6.

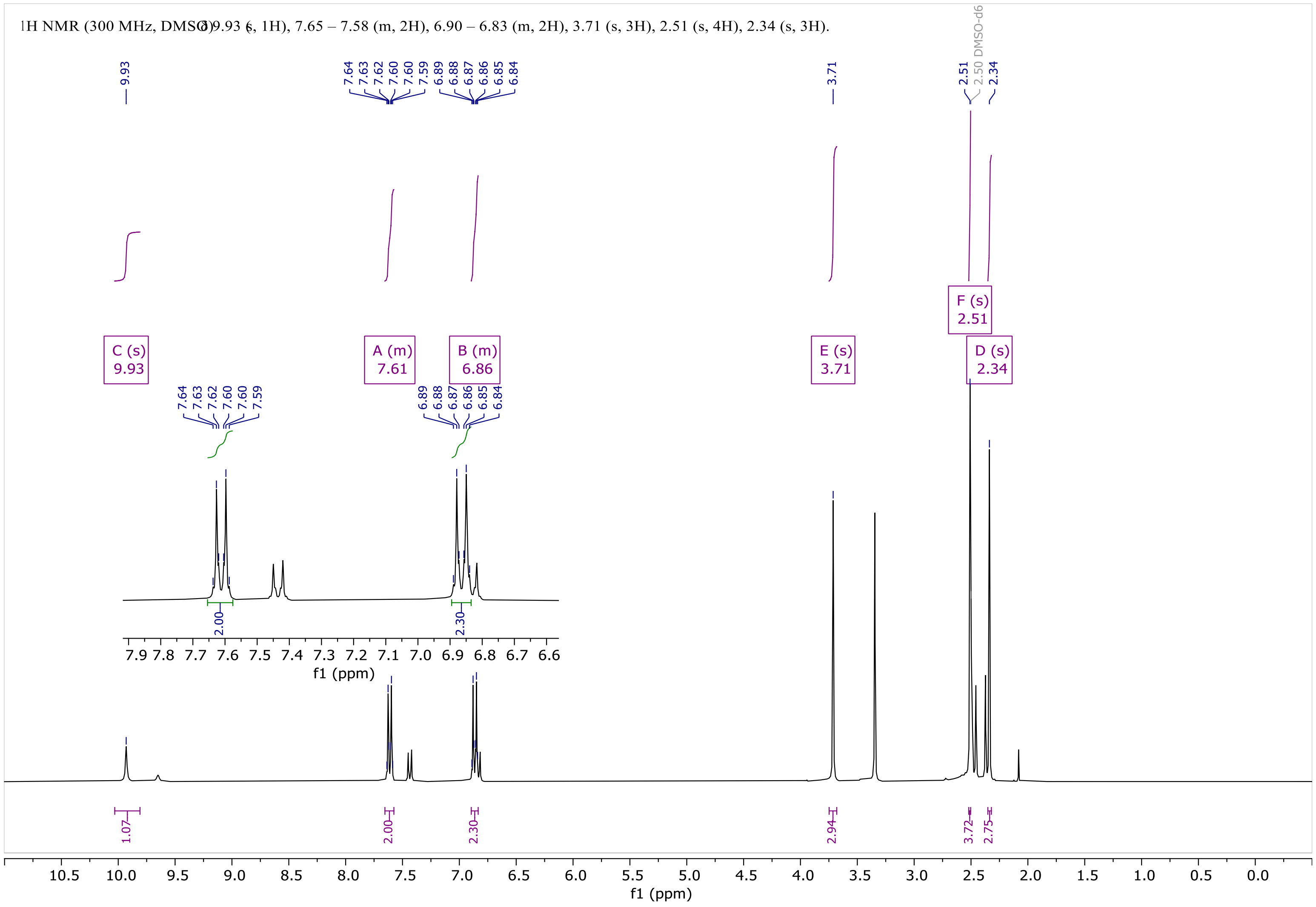

Figure S159: ^{1}H NMR spectrum of (*E*)-4-((1,3,5-trimethyl-1*H*-pyrazol-4-yl)diazenyl)phenol in DMSO-d_6.

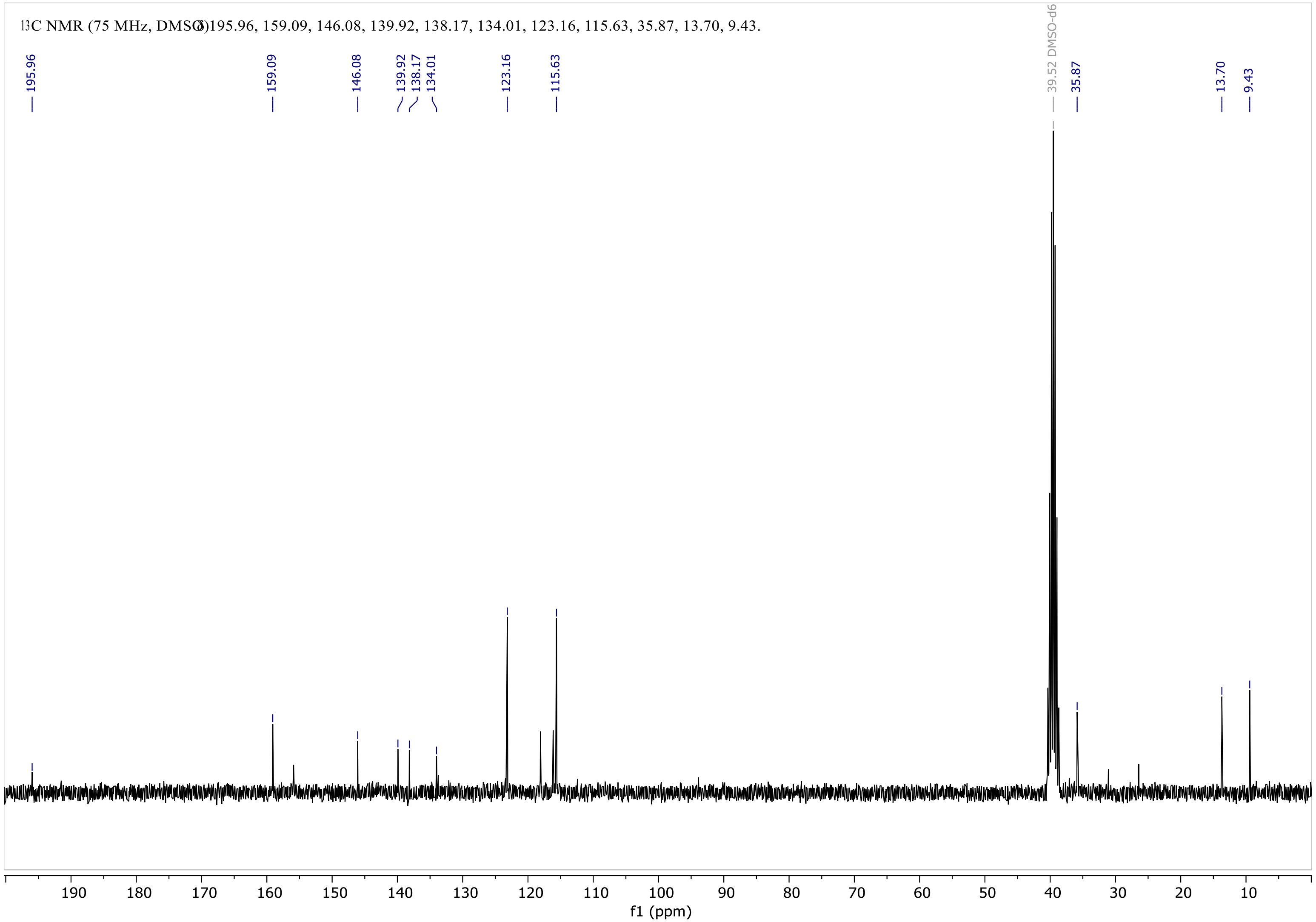

Figure S160: ^{13}C NMR spectrum of (*E*)-4-((1,3,5-trimethyl-1*H*-pyrazol-4-yl)diazenyl)phenol in DMSO-d_6.

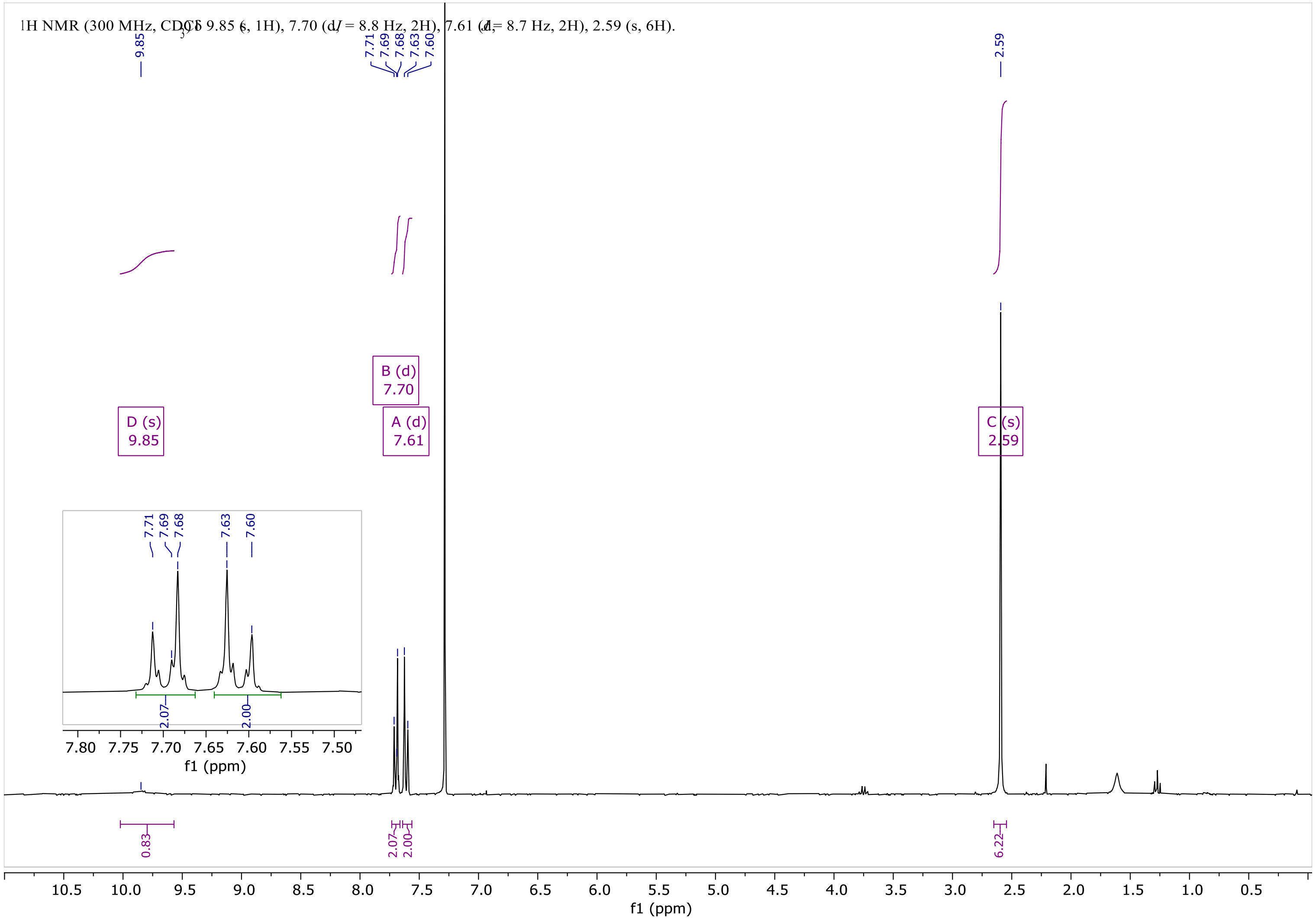

Figure S161: ^{1}H NMR spectrum of (*E*)-4-((4-bromophenyl)diazenyl)-3,5-dimethyl-1*H*-pyrazole in DMSO-d_6.

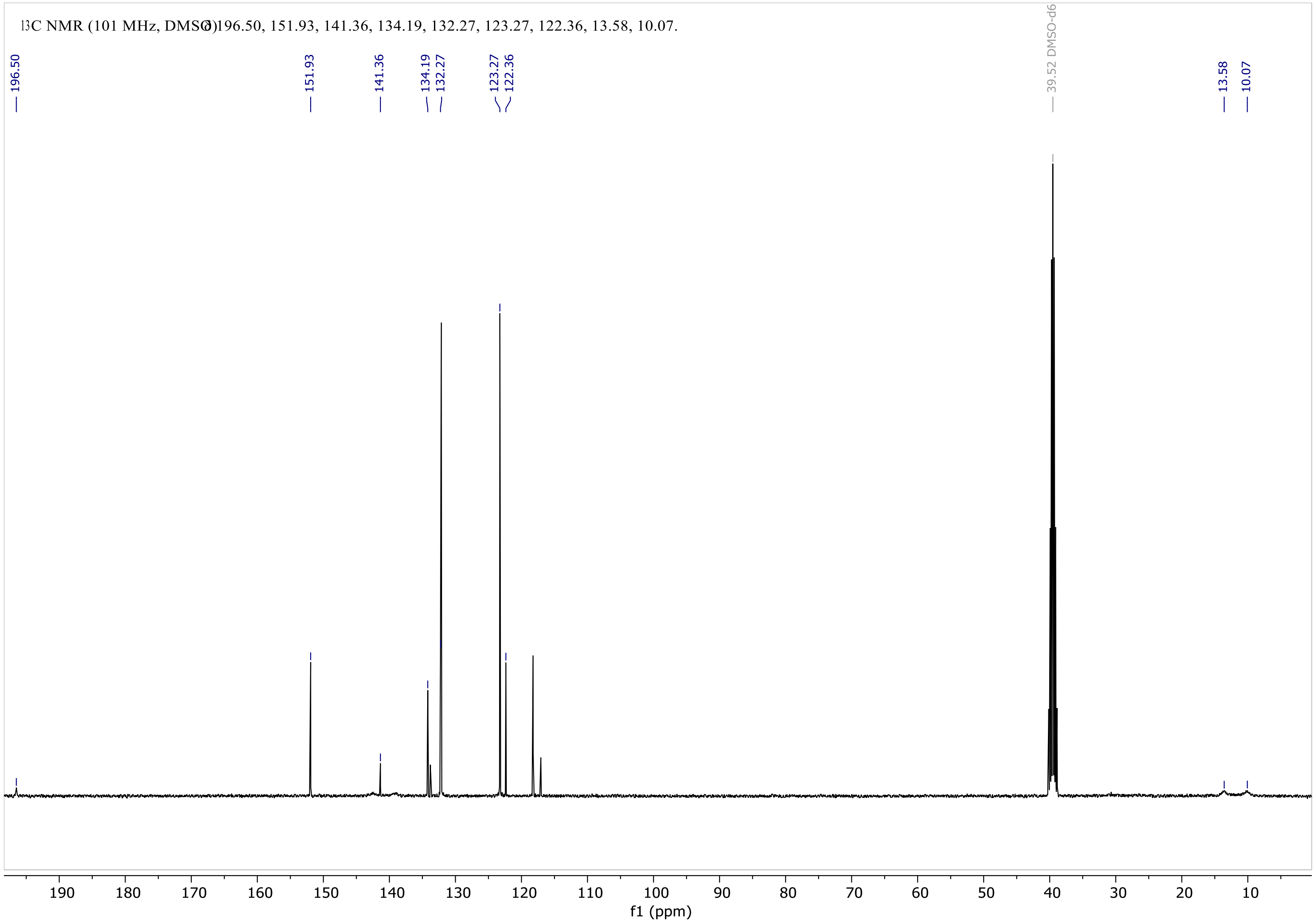

Figure S162: ^{13}C NMR spectrum of (*E*)-4-((4-bromophenyl)diazenyl)-3,5-dimethyl-1*H*-pyrazole in DMSO-d_6.

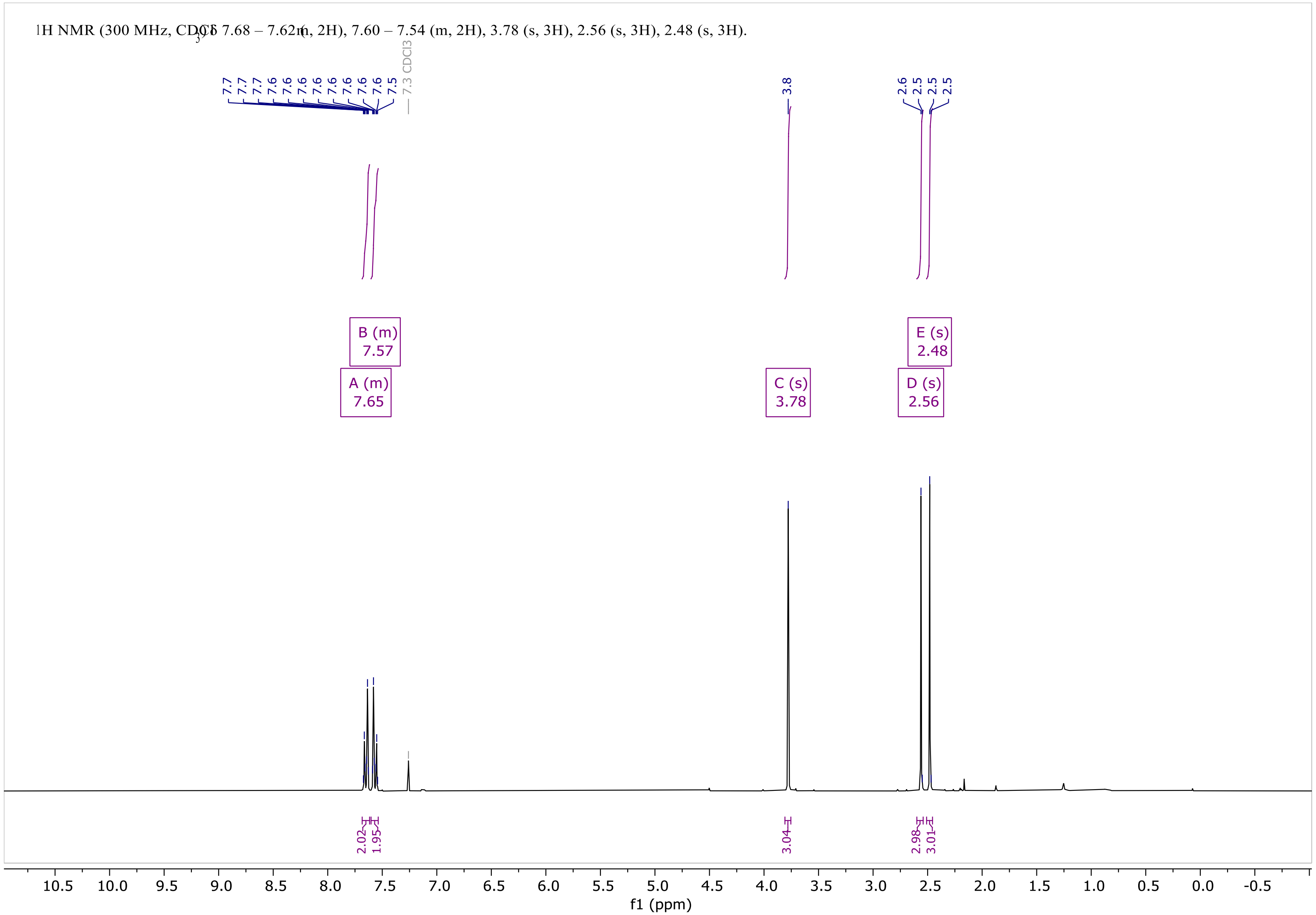

Figure S163: ^{1}H NMR spectrum of (*E*)-4-((4-bromophenyl)diazenyl)-1,3,5-trimethyl-1*H*-pyrazole in DMSO-d_6.

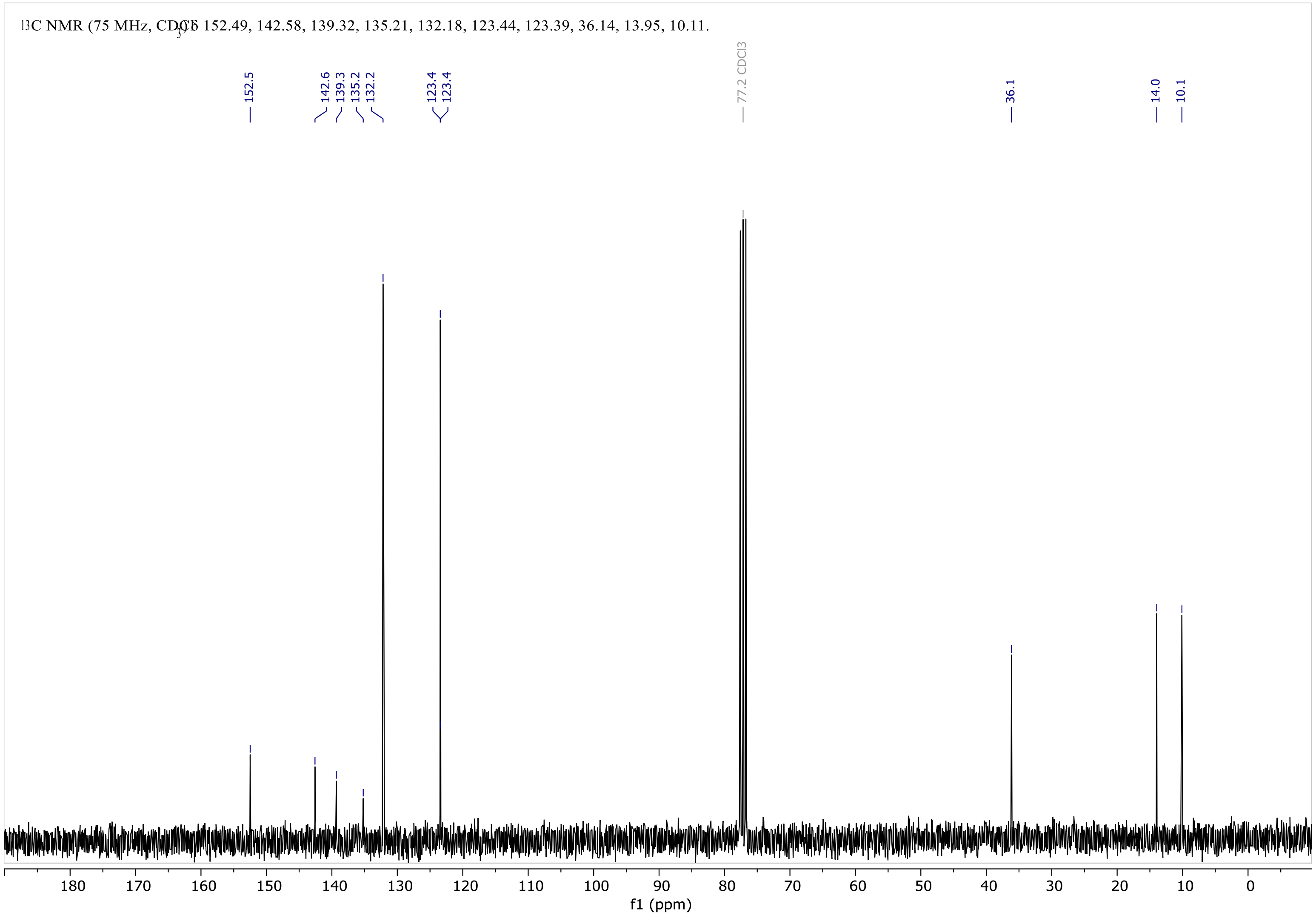

Figure S164: ^{13}C NMR spectrum of (*E*)-4-((4-bromophenyl)diazenyl)-1,3,5-trimethyl-1*H*-pyrazole in DMSO-d_6.

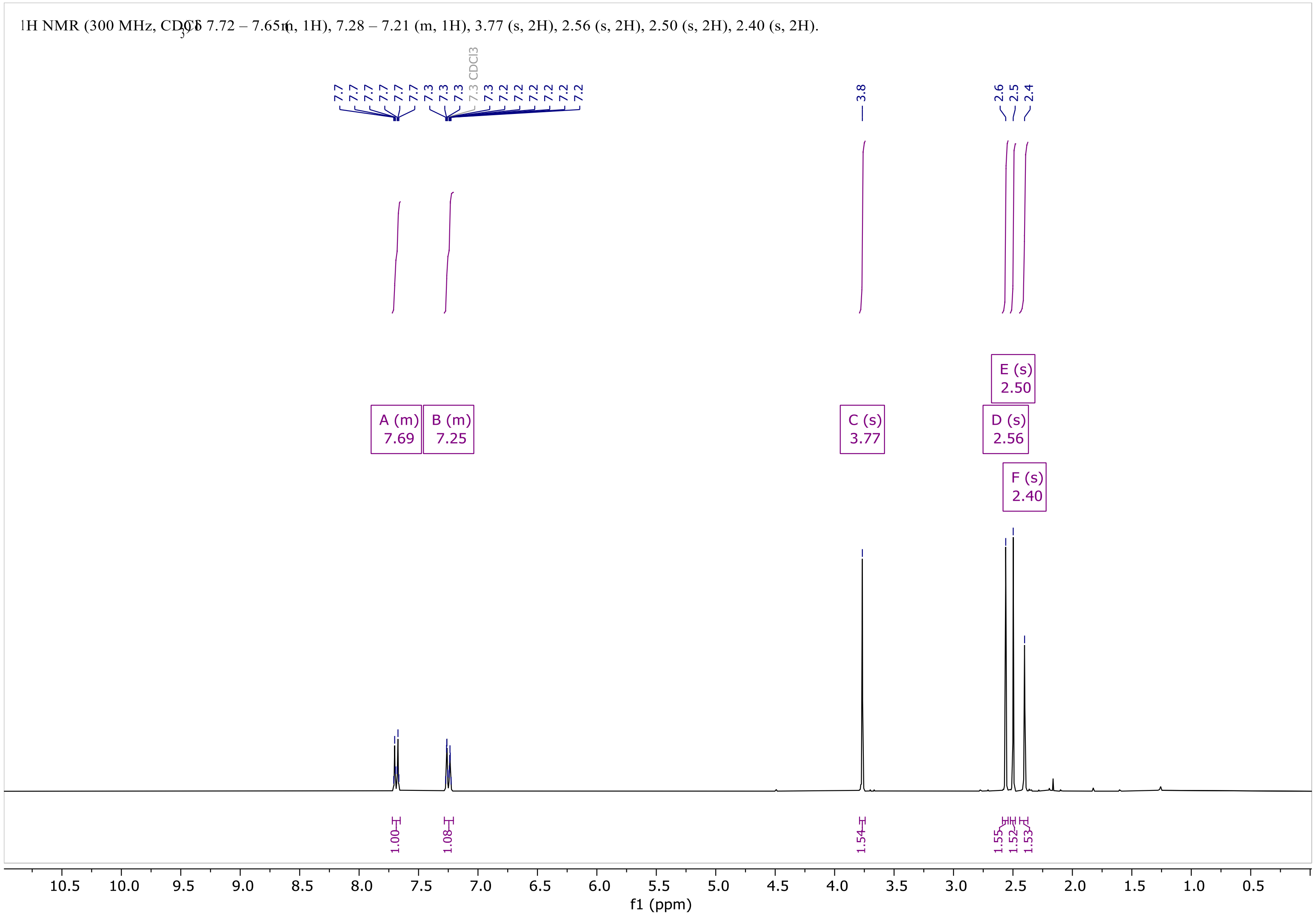

Figure S165: ^{1}H NMR spectrum (*E*)-3,5-dimethyl-4-(*p*-tolyldiazenyl)-1*H*-pyrazole in DMSO-d_6.

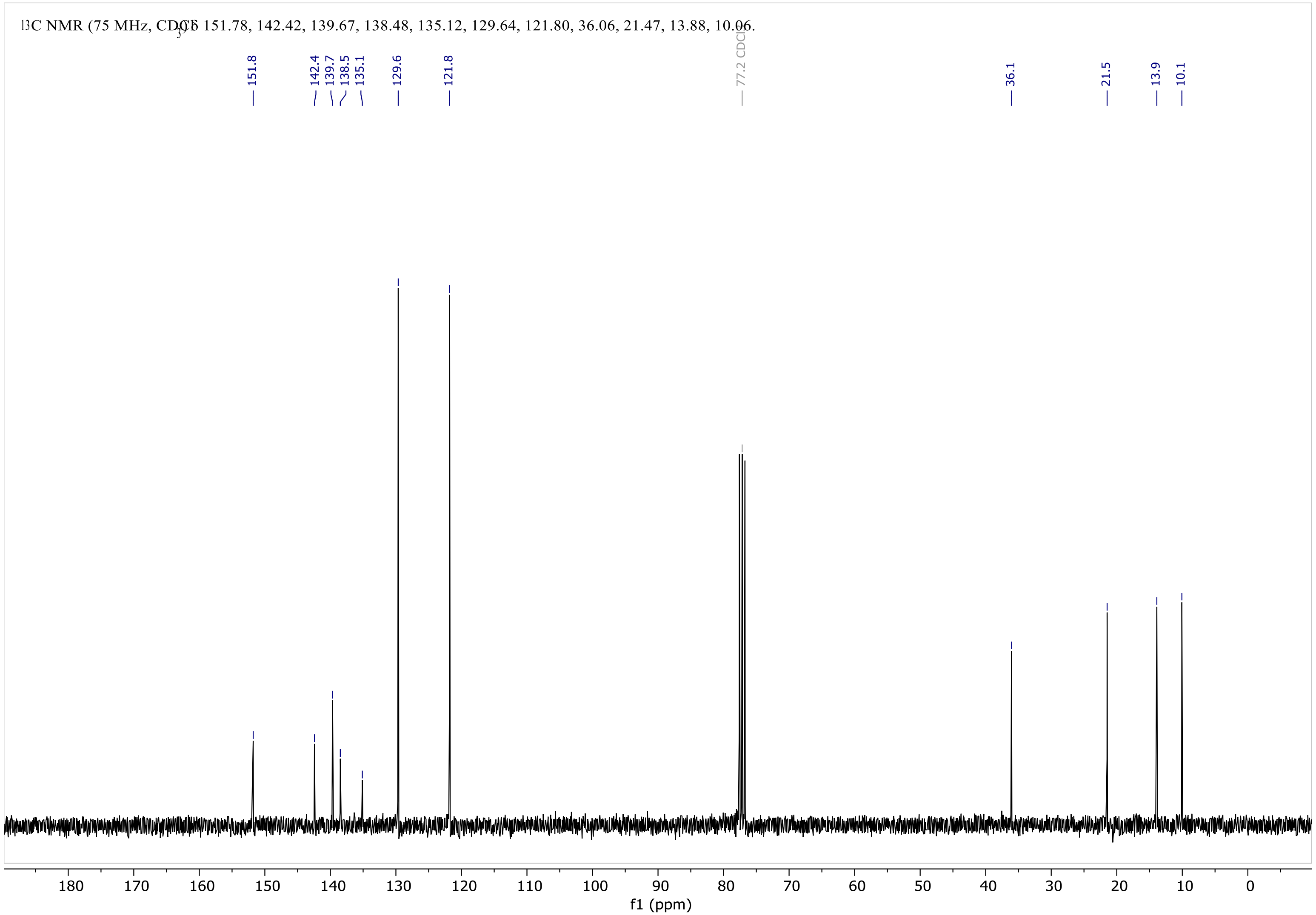

Figure S166: ^{13}C NMR spectrum of (*E*)-3,5-dimethyl-4-(*p*-tolyldiazenyl)-1*H*-pyrazole in DMSO-d_6.

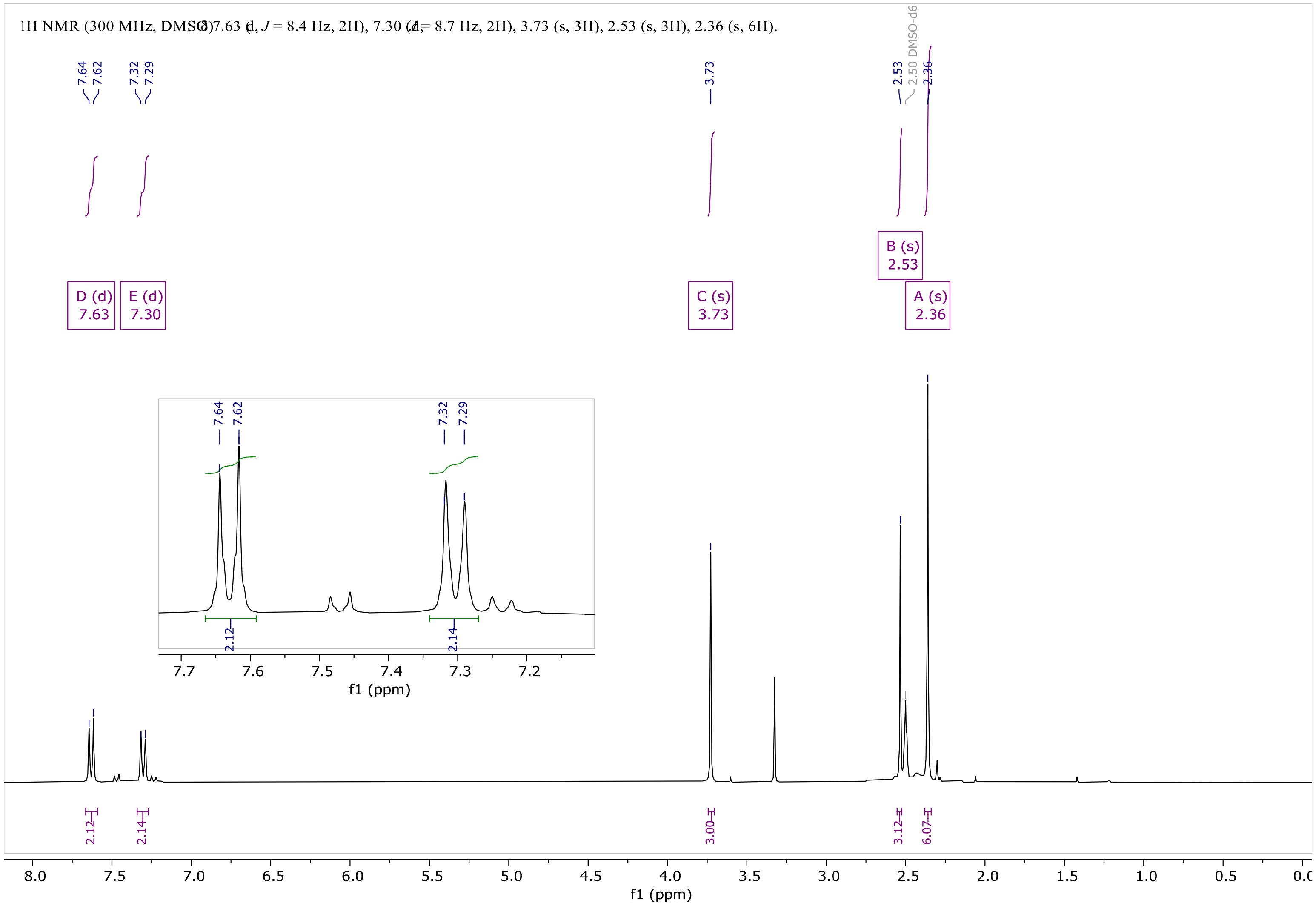

Figure S167: ^{1}H NMR spectrum of (*E*)-1,3,5-trimethyl-4-(*p*-tolyldiazenyl)-1*H*-pyrazole in DMSO-d_6.

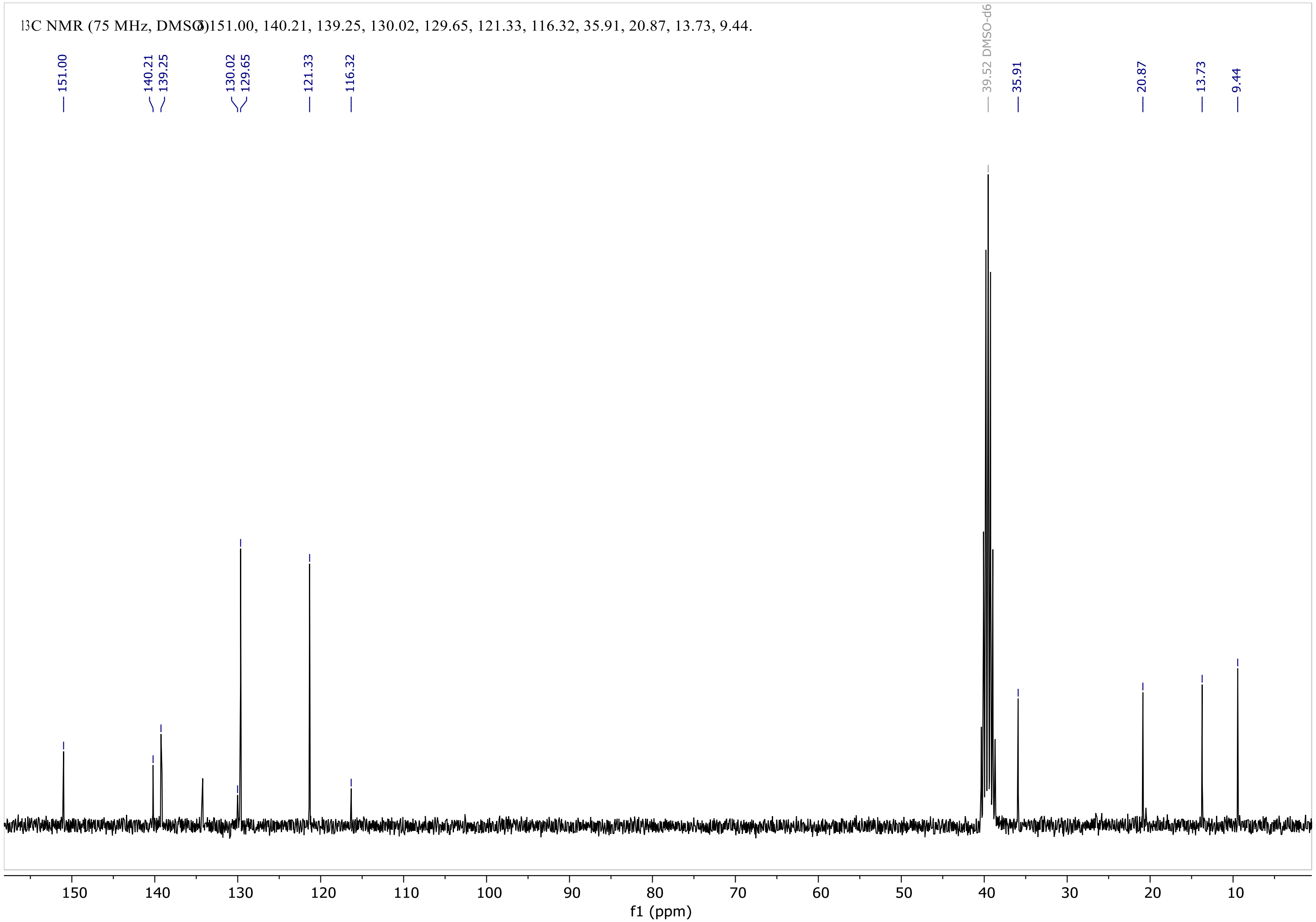

Figure S168: ^{13}C NMR spectrum of (*E*)-1,3,5-trimethyl-4-(*p*-tolyldiazenyl)-1*H*-pyrazole in DMSO-d_6.

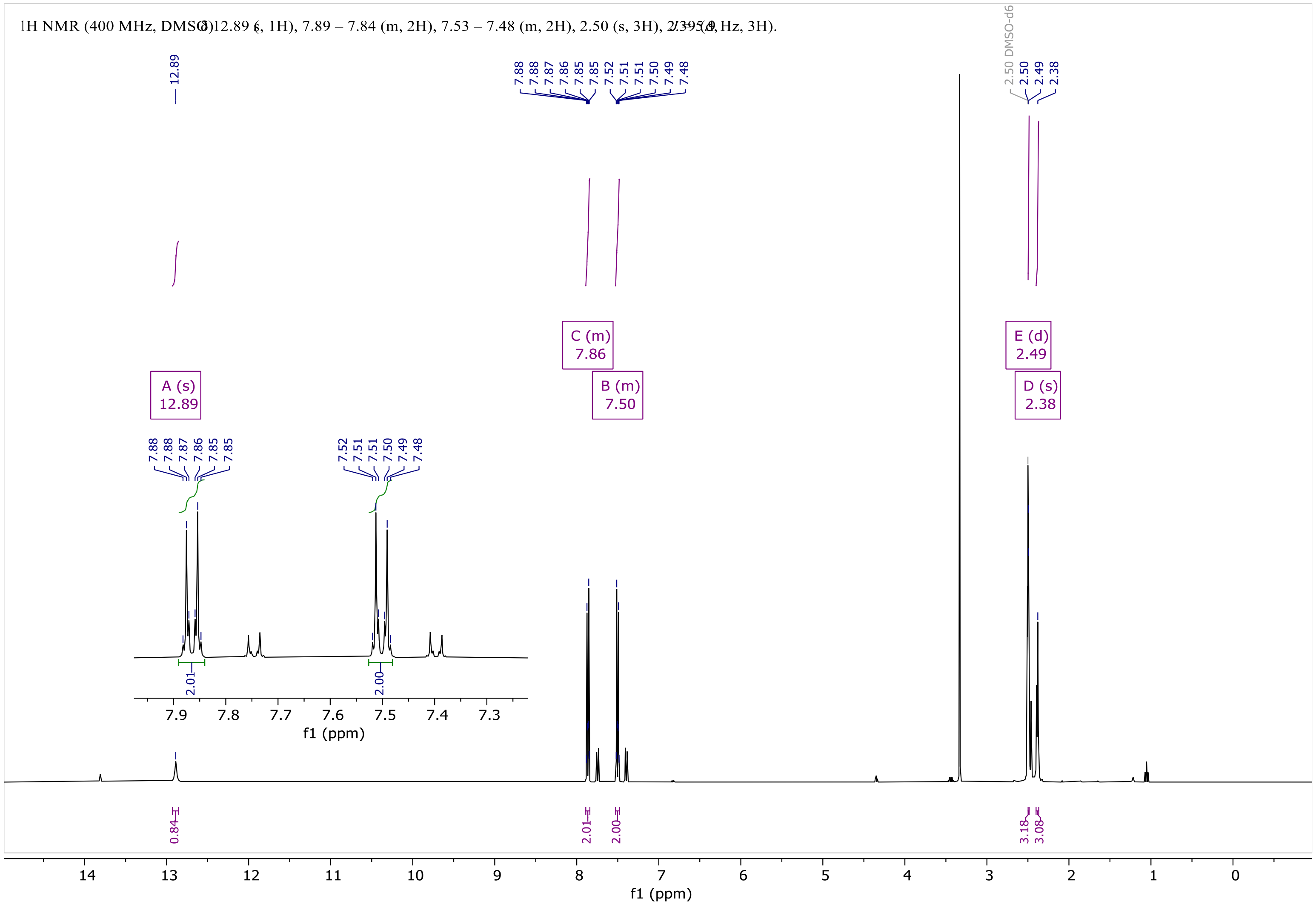

Figure S169: ^{1}H NMR spectrum of (*E*)-4-((4-iodophenyl)diazenyl)-3,5-dimethyl-1*H*-pyrazole in DMSO-d_6.

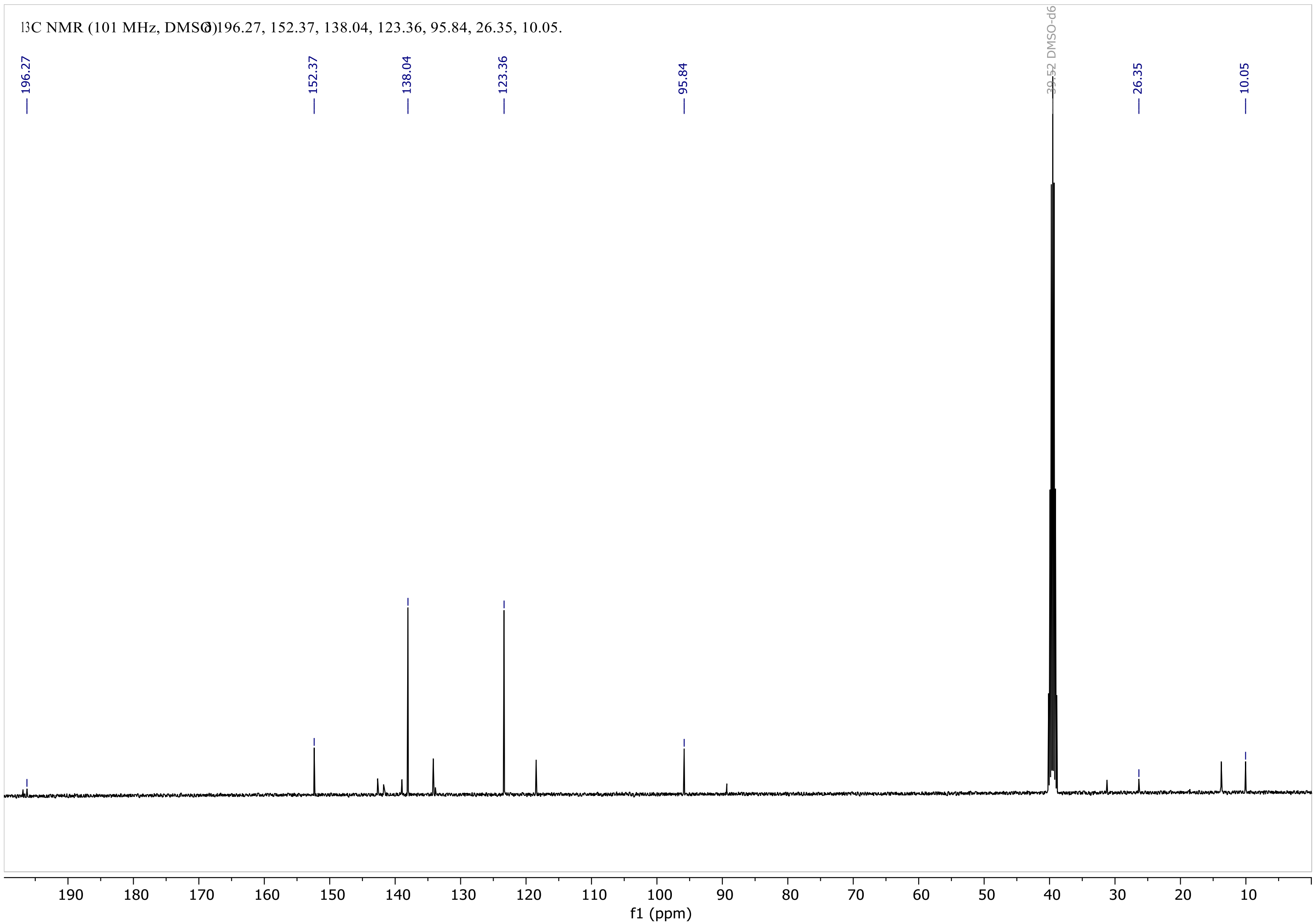

Figure S170: ^{13}C NMR spectrum of (*E*)-4-((4-iodophenyl)diazenyl)-3,5-dimethyl-1*H*-pyrazole in DMSO-d_6.

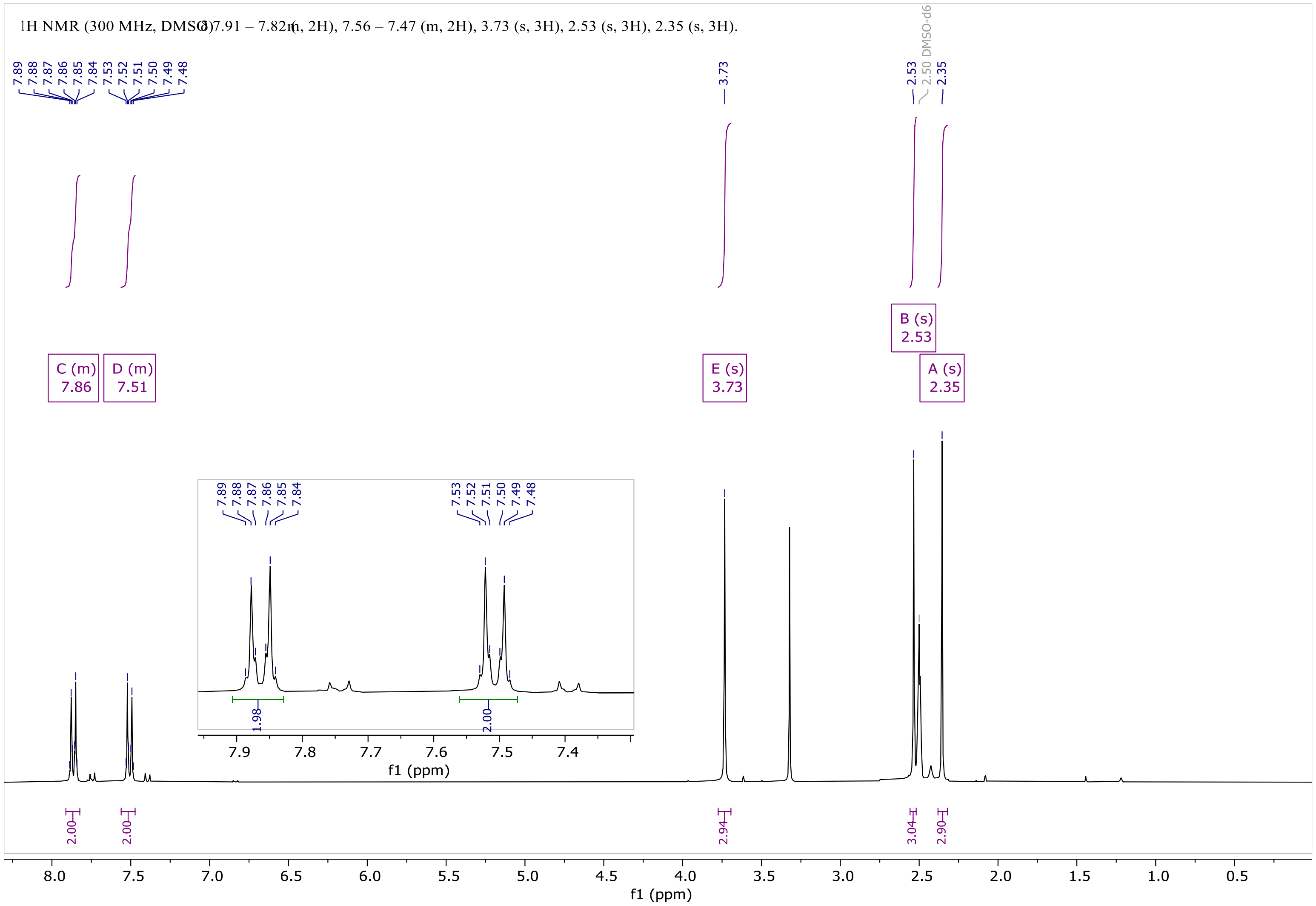

Figure S171: ^{1}H NMR spectrum of (*E*)-4-((4-iodophenyl)diazenyl)-1,3,5-trimethyl-1*H*-pyrazole in DMSO-d_6.

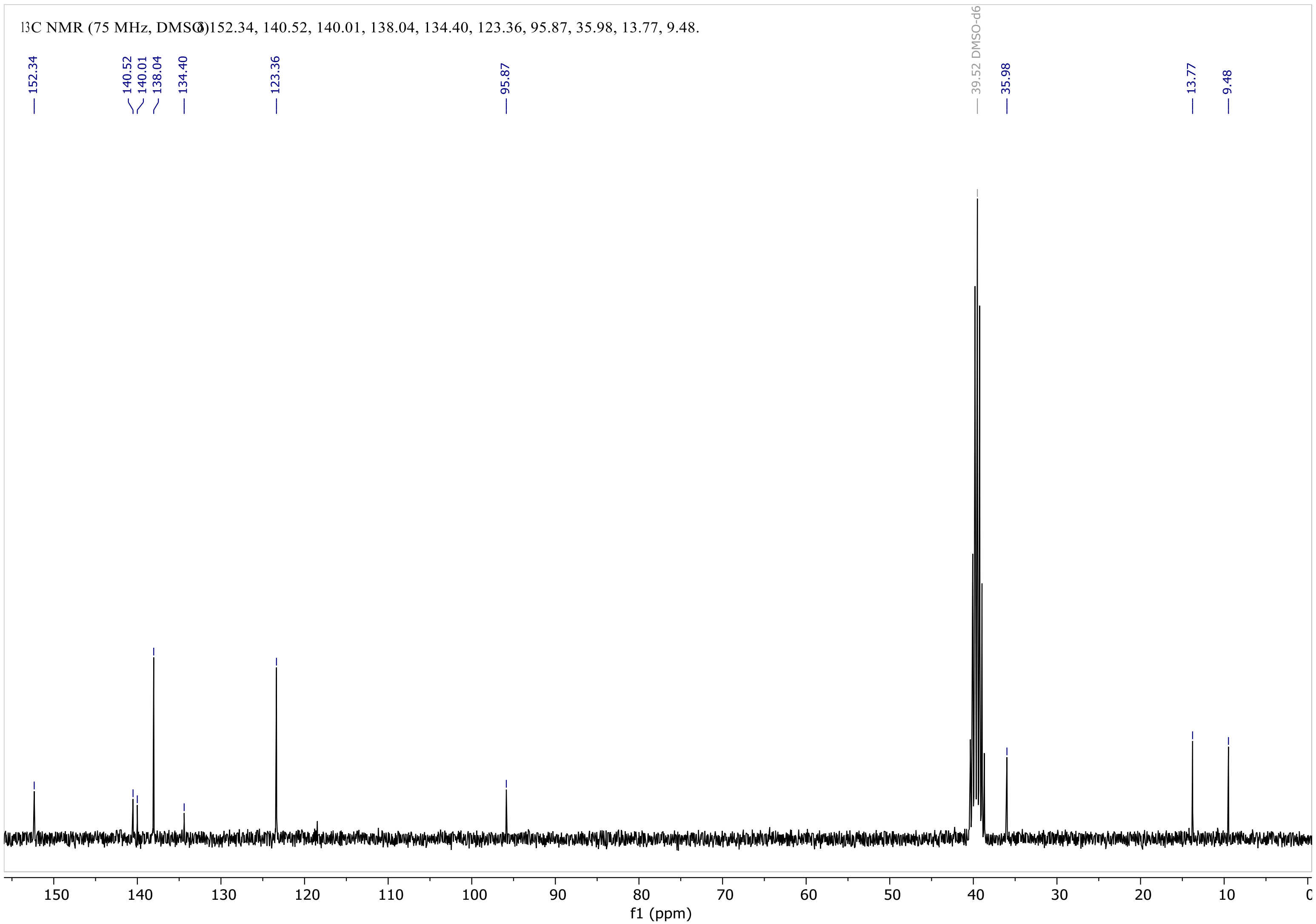

Figure 172: ^{13}C NMR spectrum of (*E*)-4-((4-iodophenyl)diazenyl)-1,3,5-trimethyl-1*H*-pyrazole in DMSO-d_6.

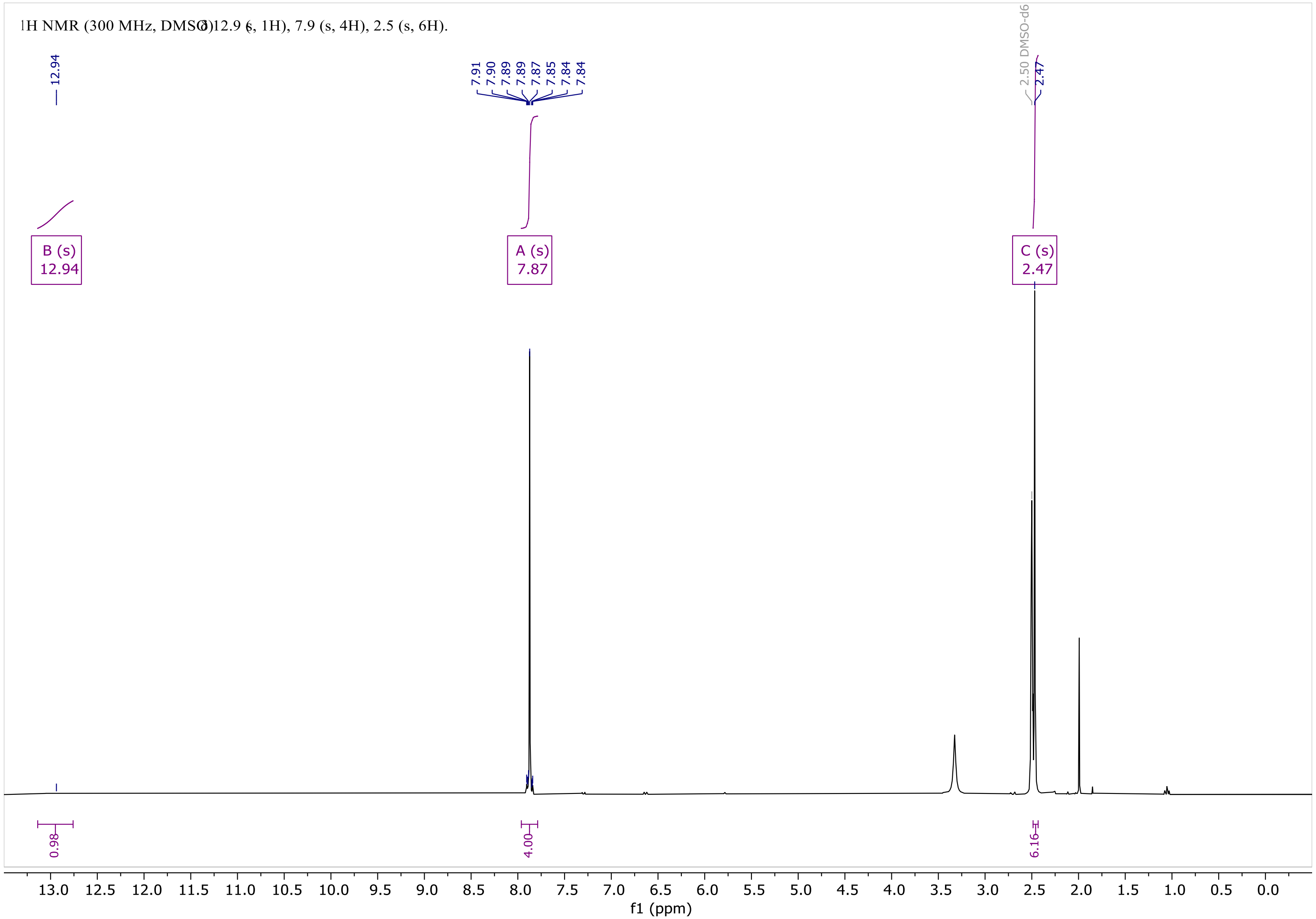

Figure S173: ^{1}H NMR spectrum of (*E*)-3,5-dimethyl-4-((4-(trifluoromethyl)phenyl)diazenyl)-1*H*-pyrazole in DMSO-d_6.

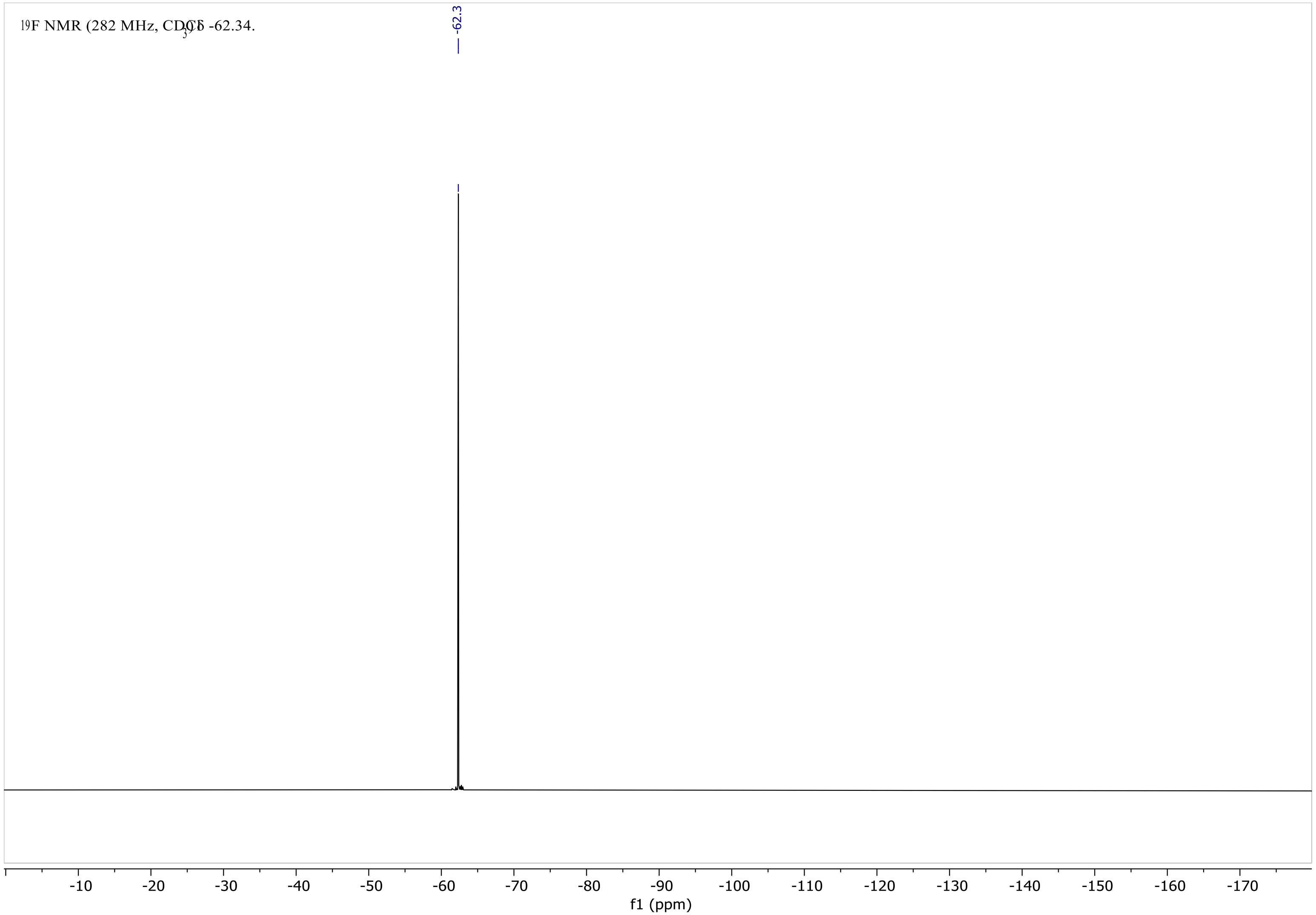

Figure S174: ^{19}F NMR spectrum of (*E*)-3,5-dimethyl-4-((4-(trifluoromethyl)phenyl)diazenyl)-1*H*-pyrazole in DMSO-d_6.

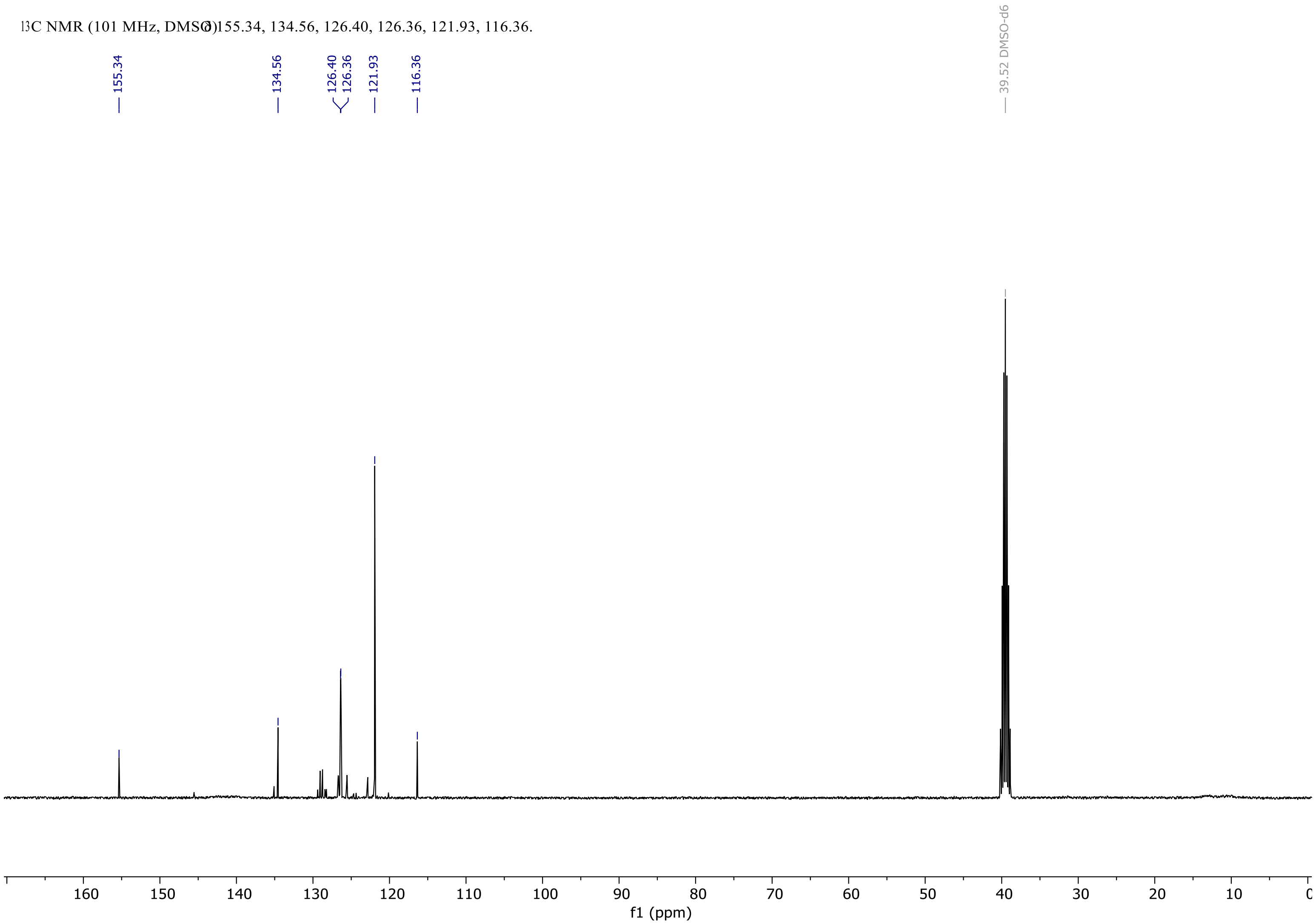

Figure S175: ^{13}C NMR spectrum of (*E*)-3,5-dimethyl-4-((4-(trifluoromethyl)phenyl)diazenyl)-1*H*-pyrazole in DMSO-d_6.

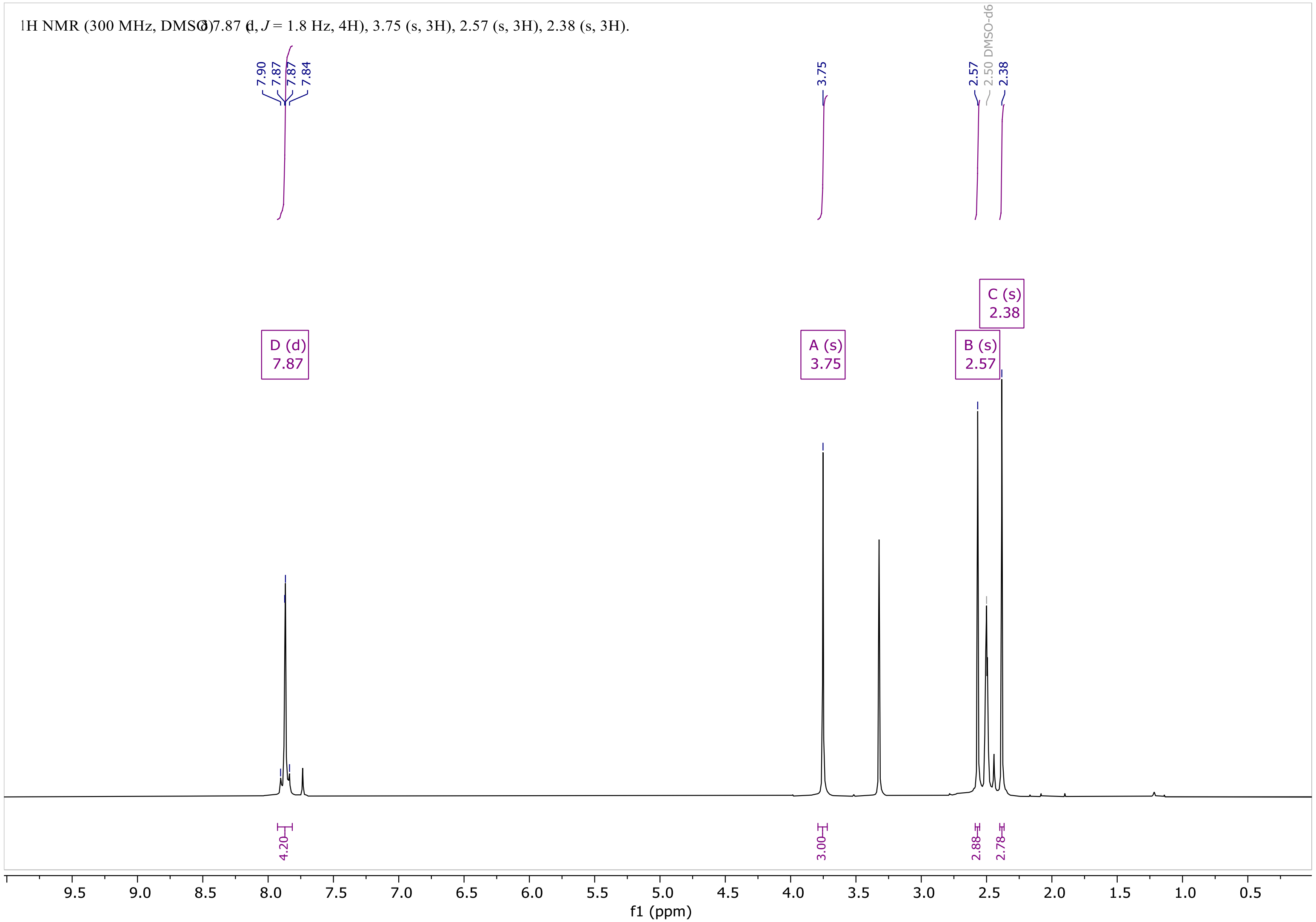

Figure S176: ^{1}H NMR spectrum of (*E*)-1,3,5-trimethyl-4-((4-(trifluoromethyl)phenyl)diazenyl)-1*H*-pyrazole in DMSO-d_6.

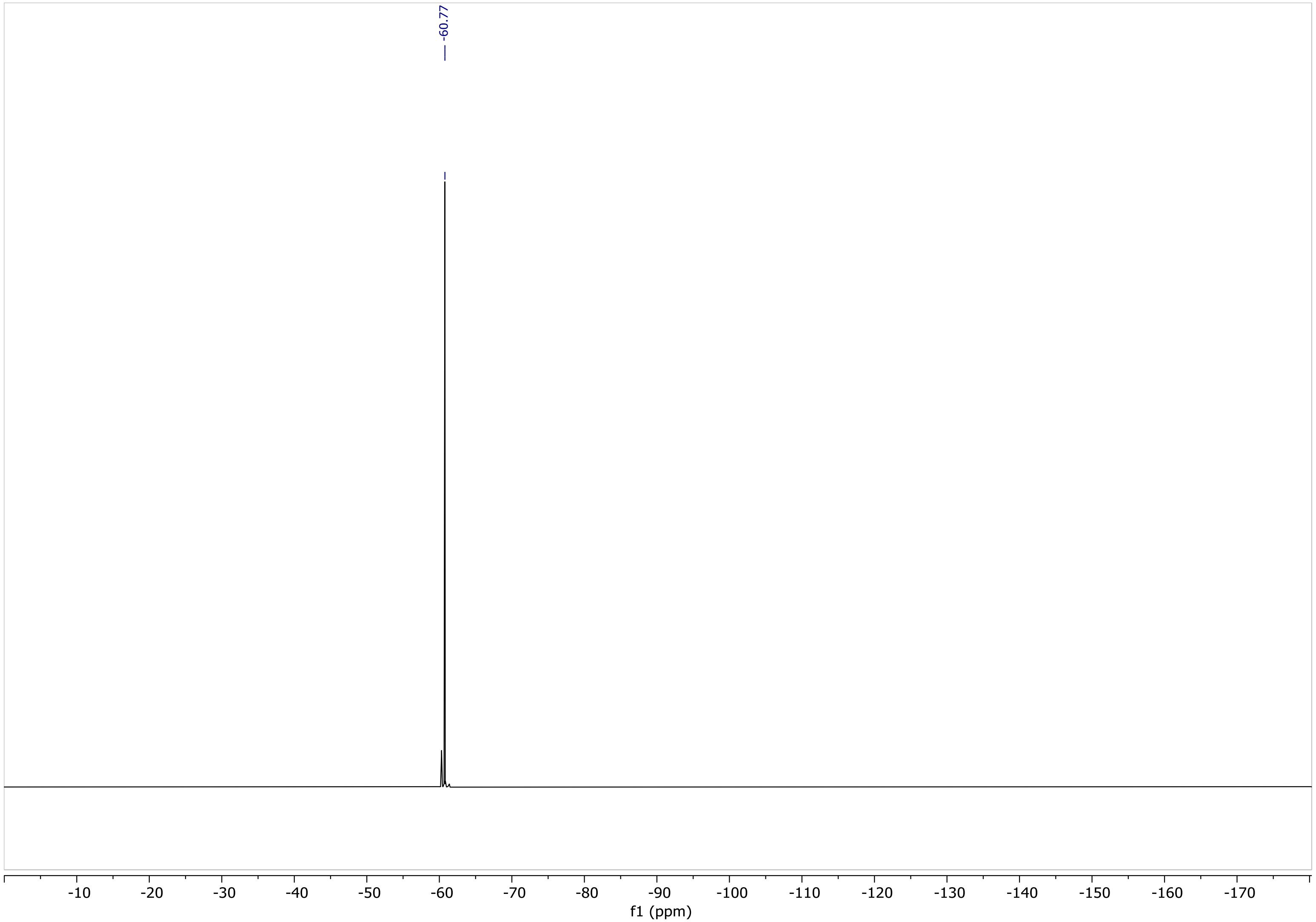

Figure S177: ^{19}F NMR spectrum of (*E*)-1,3,5-trimethyl-4-((4-(trifluoromethyl)phenyl)diazenyl)-1*H*-pyrazole in DMSO-d_6.

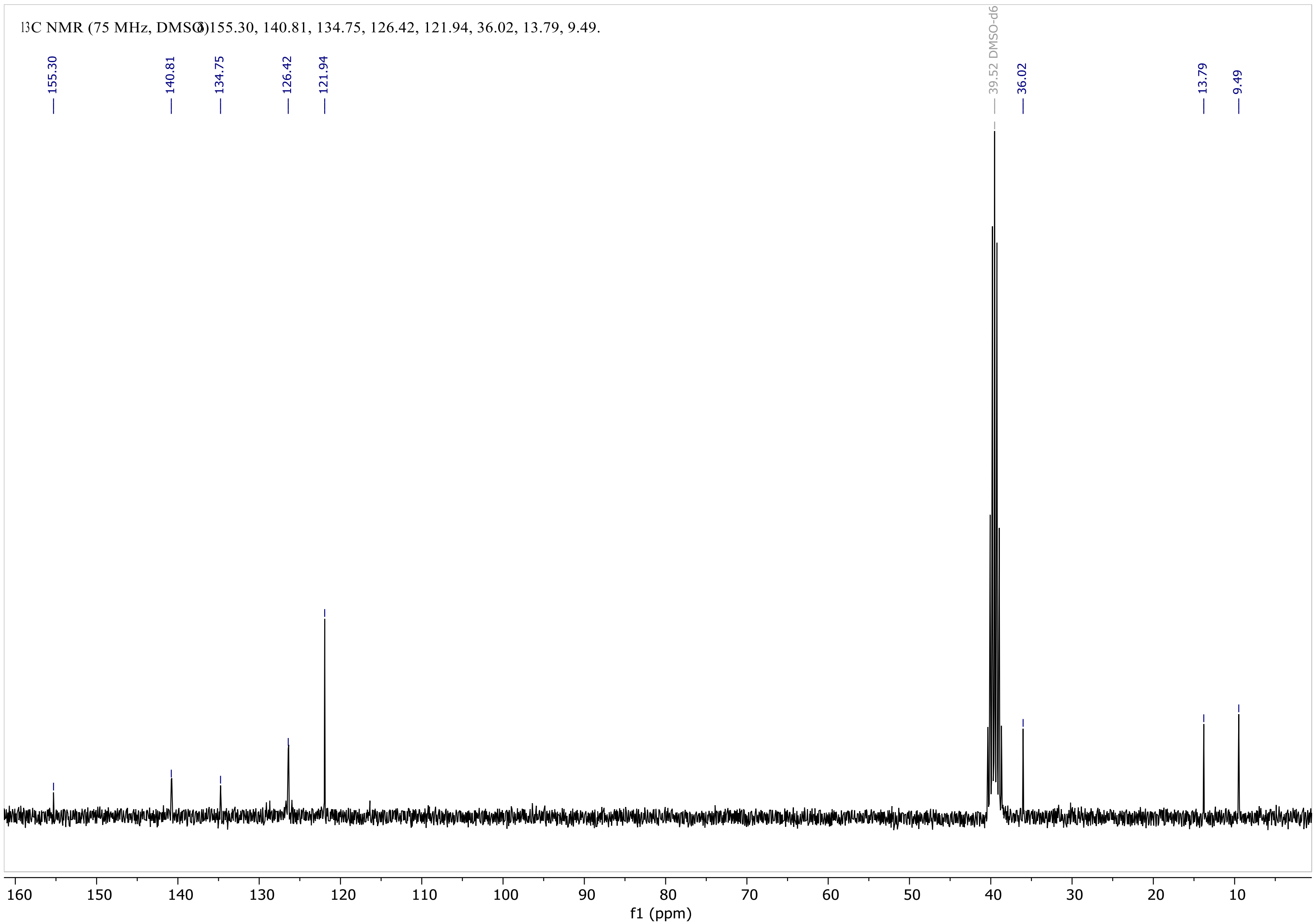

Figure S178: ^{13}C NMR spectrum of (*E*)-1,3,5-trimethyl-4-((4-(trifluoromethyl)phenyl)diazenyl)-1*H*-pyrazole in DMSO-d_6.

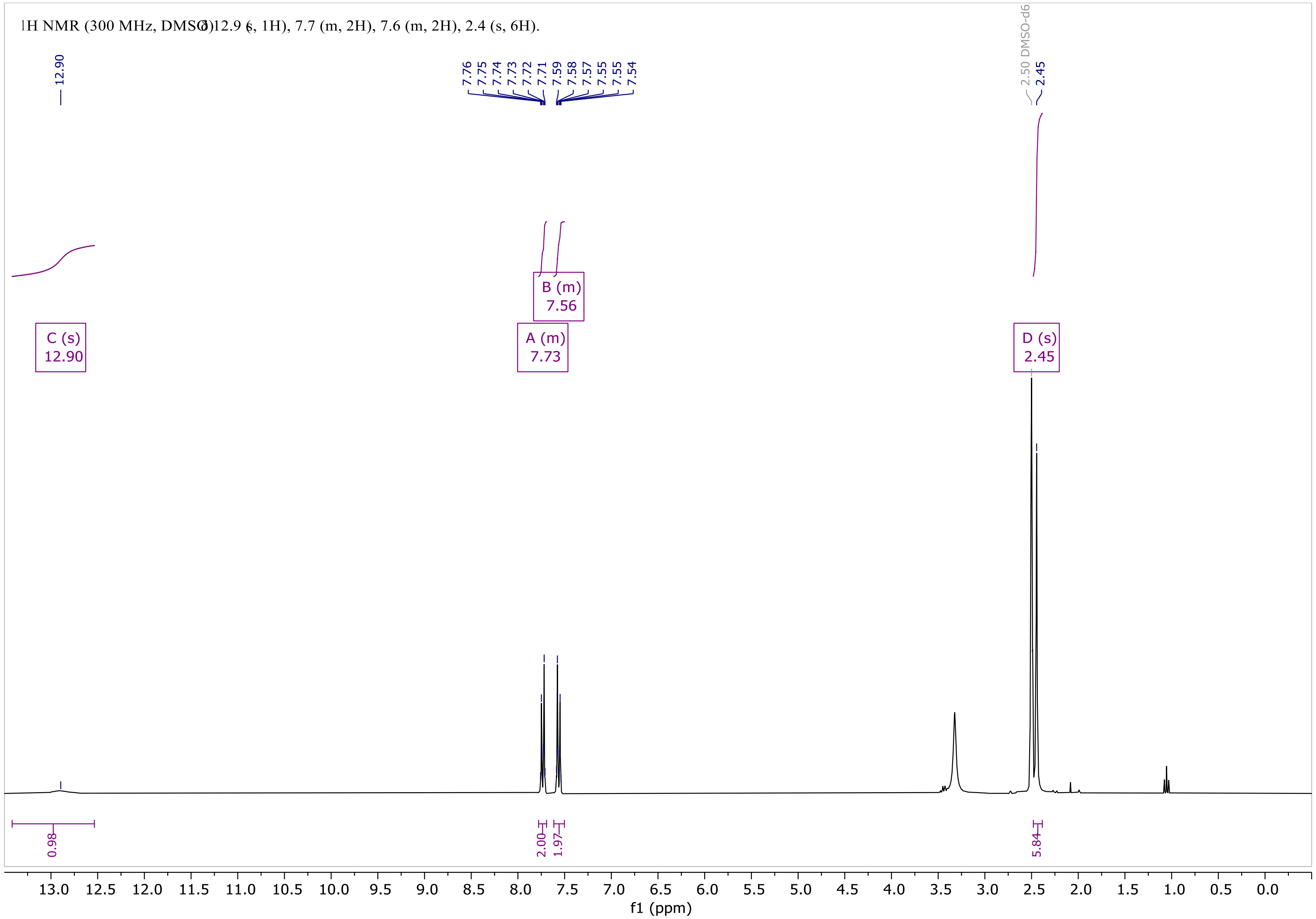

Figure S179: ^{1}H NMR spectrum of (*E*)-4-((4-chlorophenyl)diazenyl)-3,5-dimethyl-1*H*-pyrazole in DMSO-d_6.

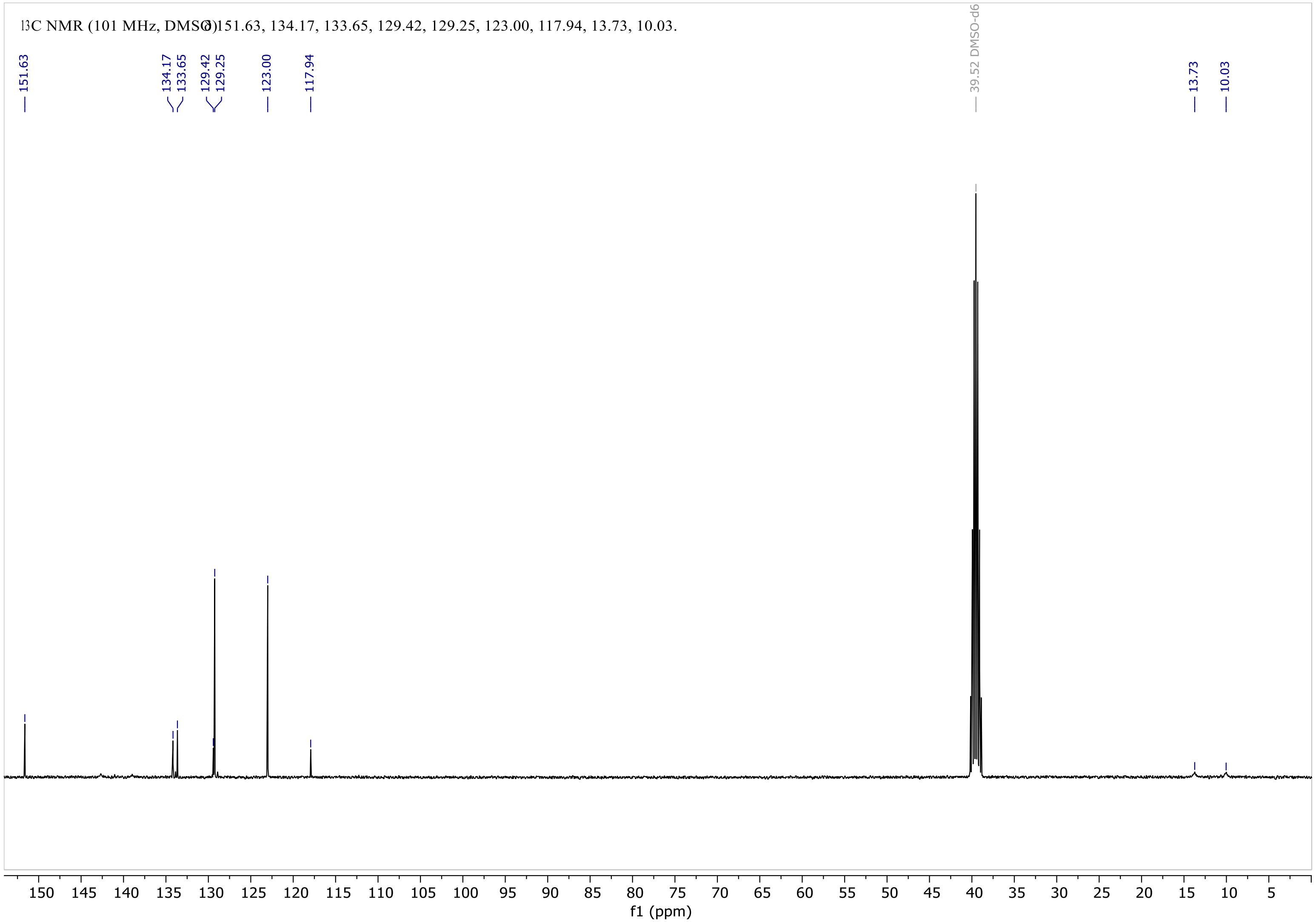

Figure S180: ^{13}C NMR spectrum of (*E*)-4-((4-chlorophenyl)diazenyl)-3,5-dimethyl-1*H*-pyrazole in DMSO-d_6.

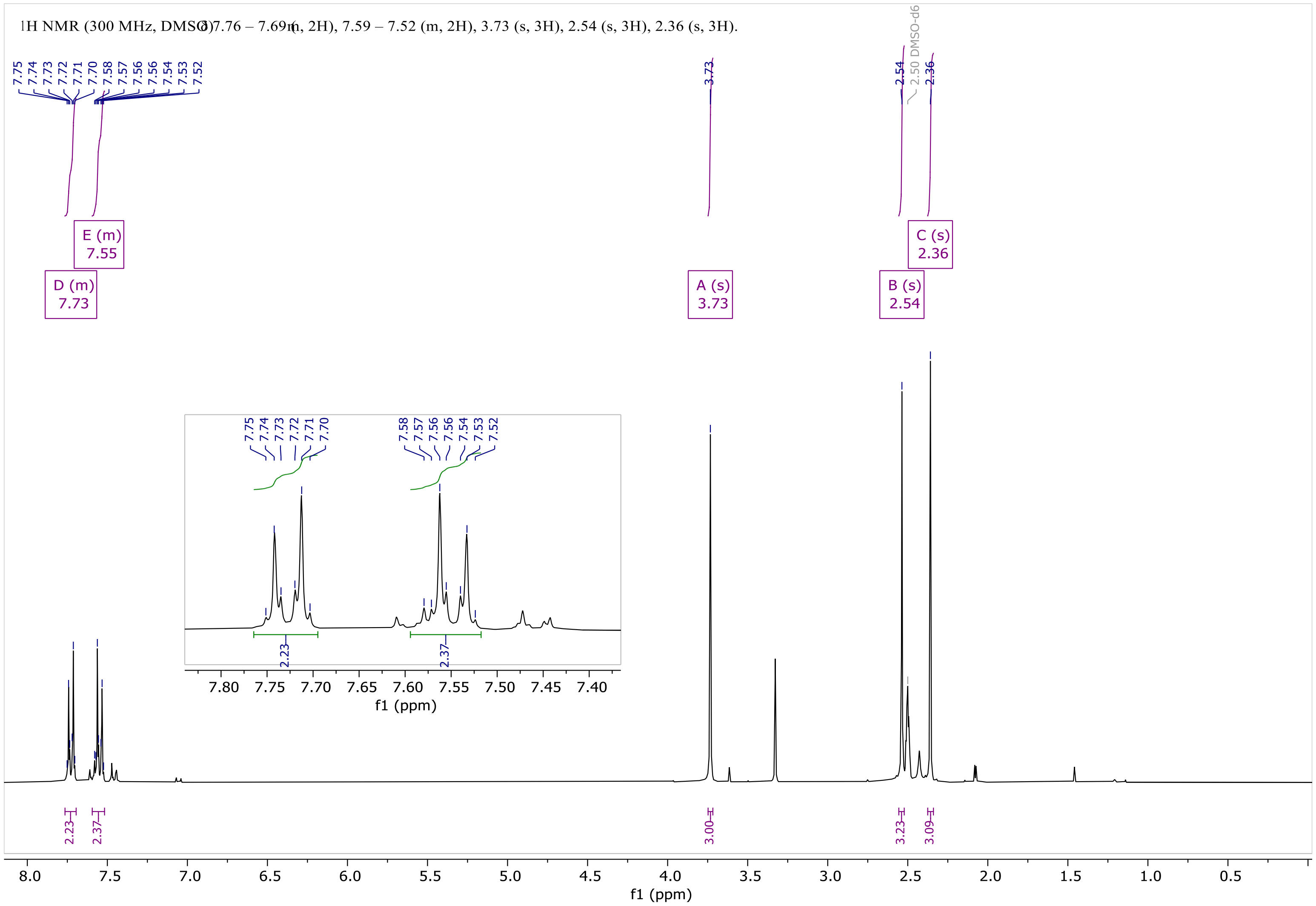

Figure S181: ^{1}H NMR spectrum of (*E*)-4-((4-chlorophenyl)diazenyl)-1,3,5-triimethyl-1*H*-pyrazole in DMSO-d_6.

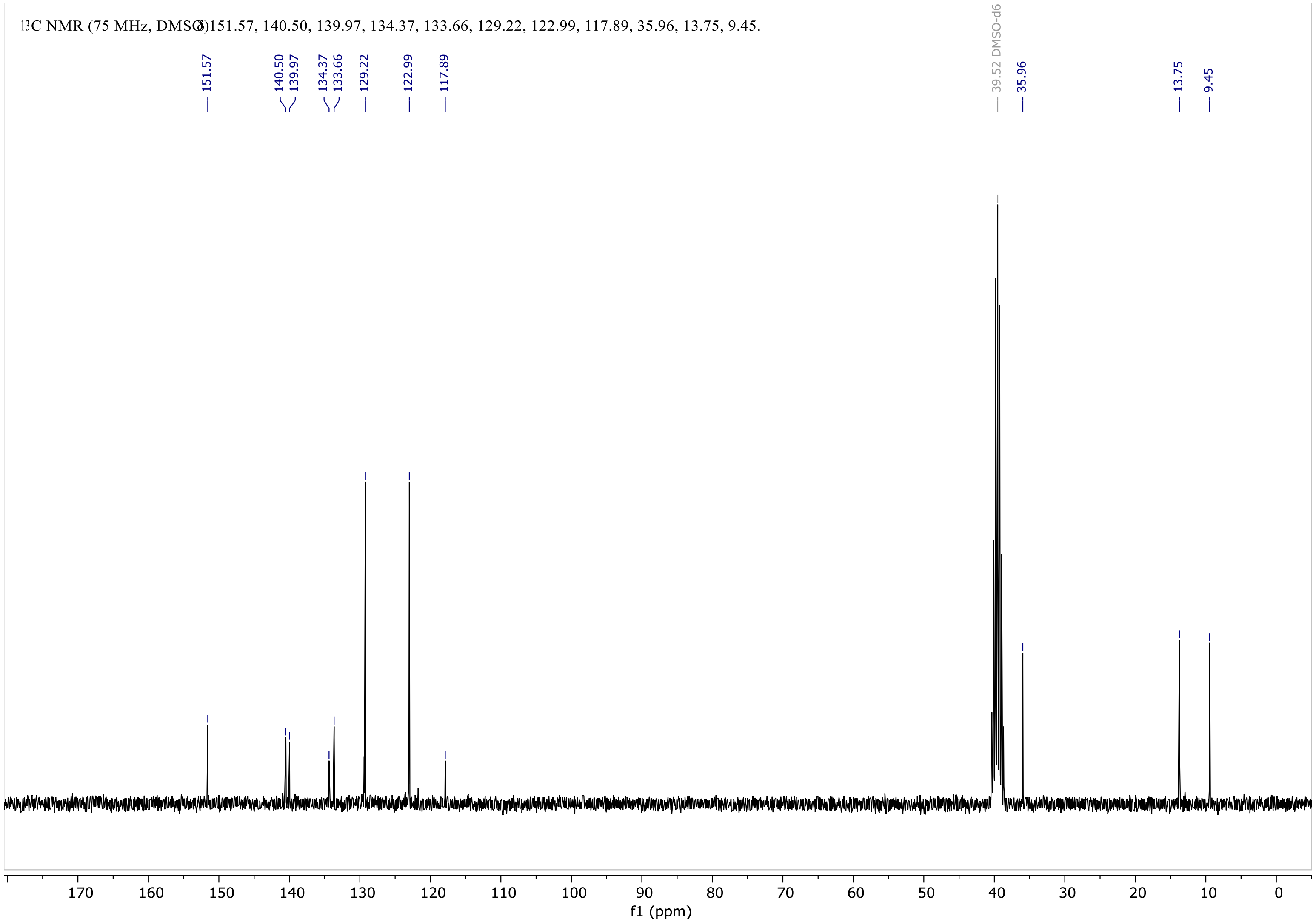

Figure S182: ^{13}C NMR spectrum of (*E*)-4-((4-chlorophenyl)diazenyl)-1,3,5-triimethyl-1*H*-pyrazole in DMSO-d_6.

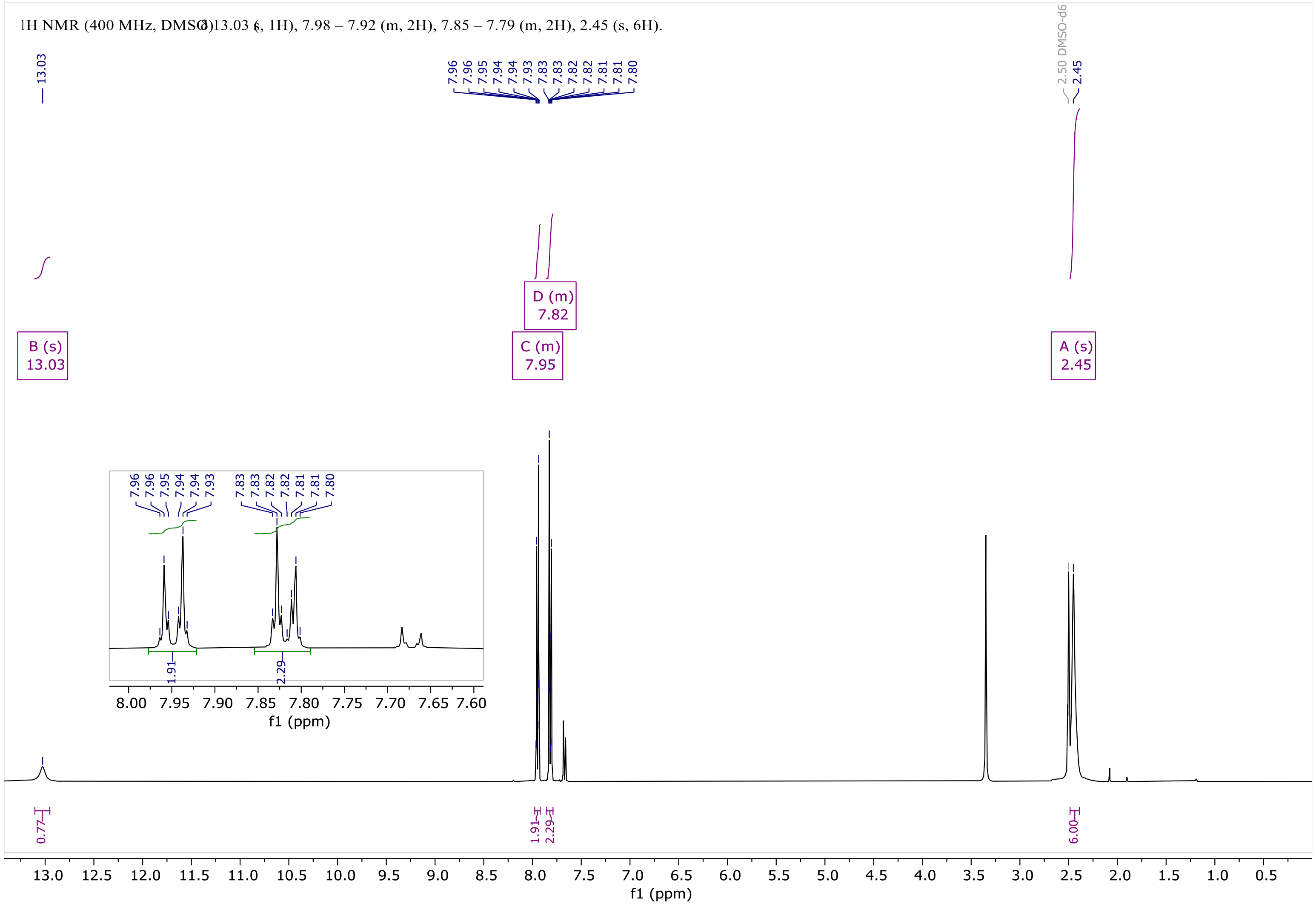

Figure S183: ^{1}H NMR spectrum of (*E*)-4-((3,5-dimethyl-1*H*-pyrazol-4-yl)diazenyl)benzonitrile in DMSO-d_6.

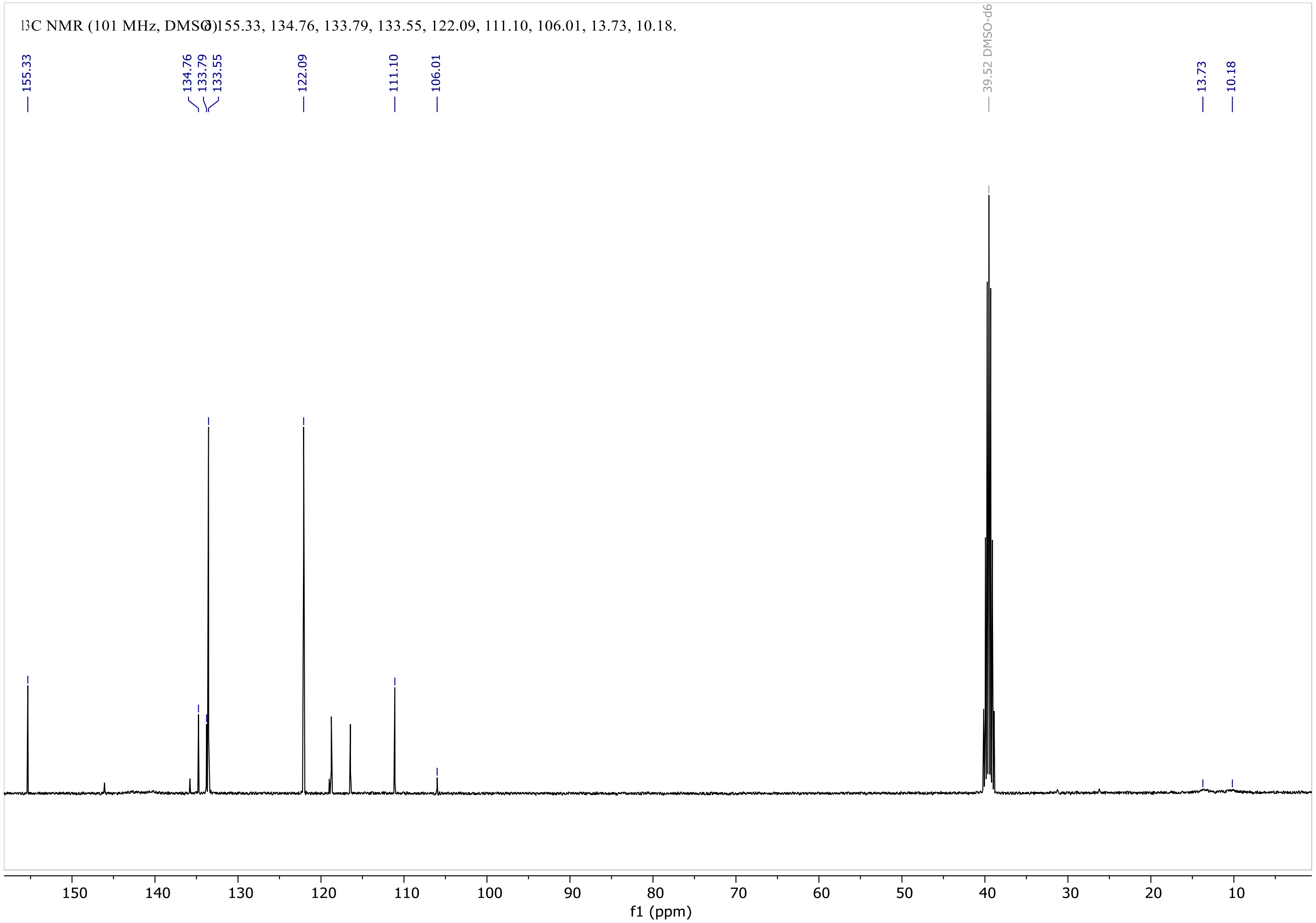

Figure S184: ^{13}C NMR spectrum of (*E*)-4-((3,5-dimethyl-1*H*-pyrazol-4-yl)diazenyl)benzonitrile in DMSO-d_6.

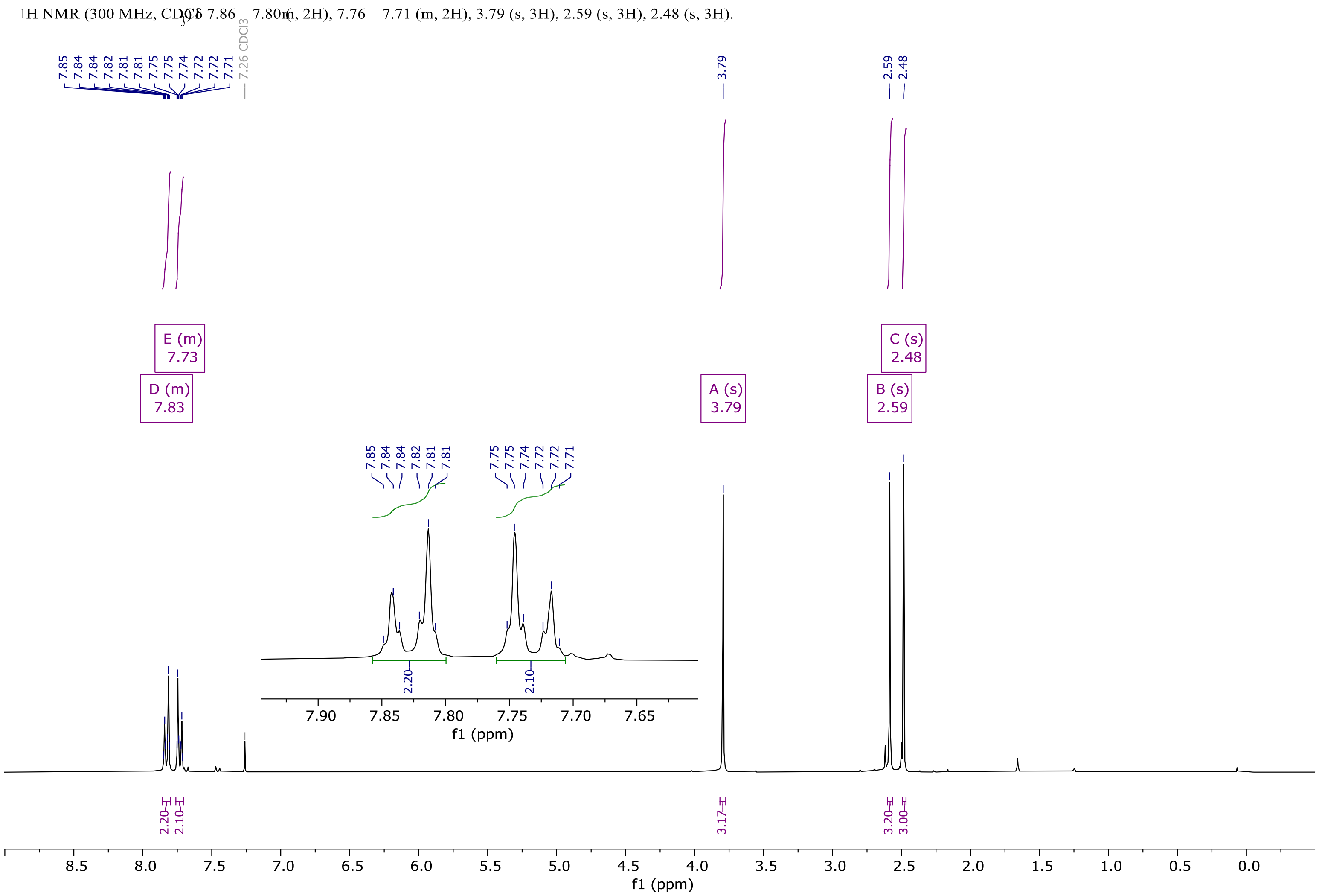

Figure S185: ^{1}H NMR spectrum of (*E*)-4-((1,3,5-trimethyl-1*H*-pyrazol-4-yl)diazenyl)benzonitrile in $CDCl_3$.

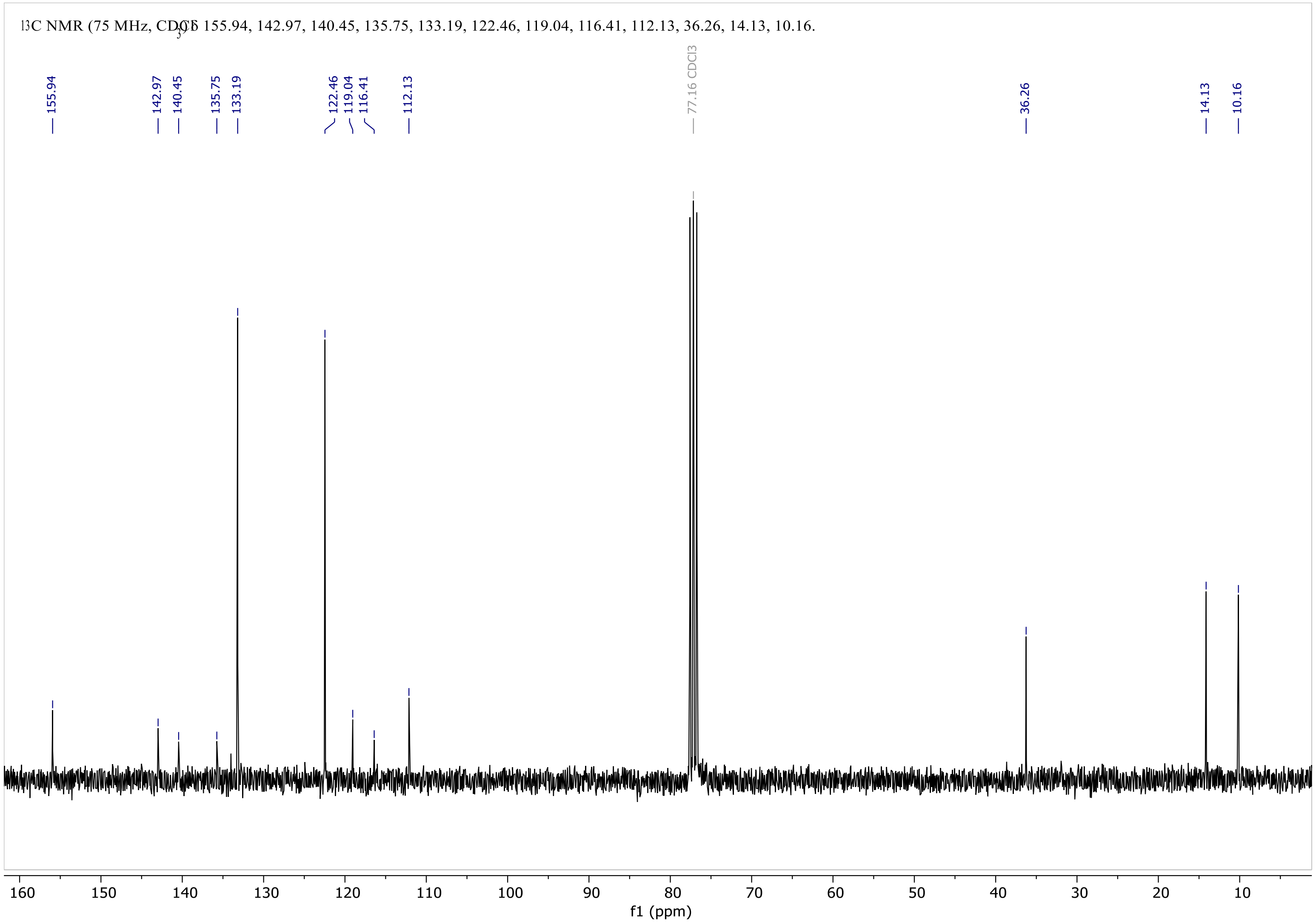

Figure S186: ^{13}C NMR spectrum of (*E*)-4-((1,3,5-trimethyl-1*H*-pyrazol-4-yl)diazenyl)benzonitrile in $CDCl_3$.

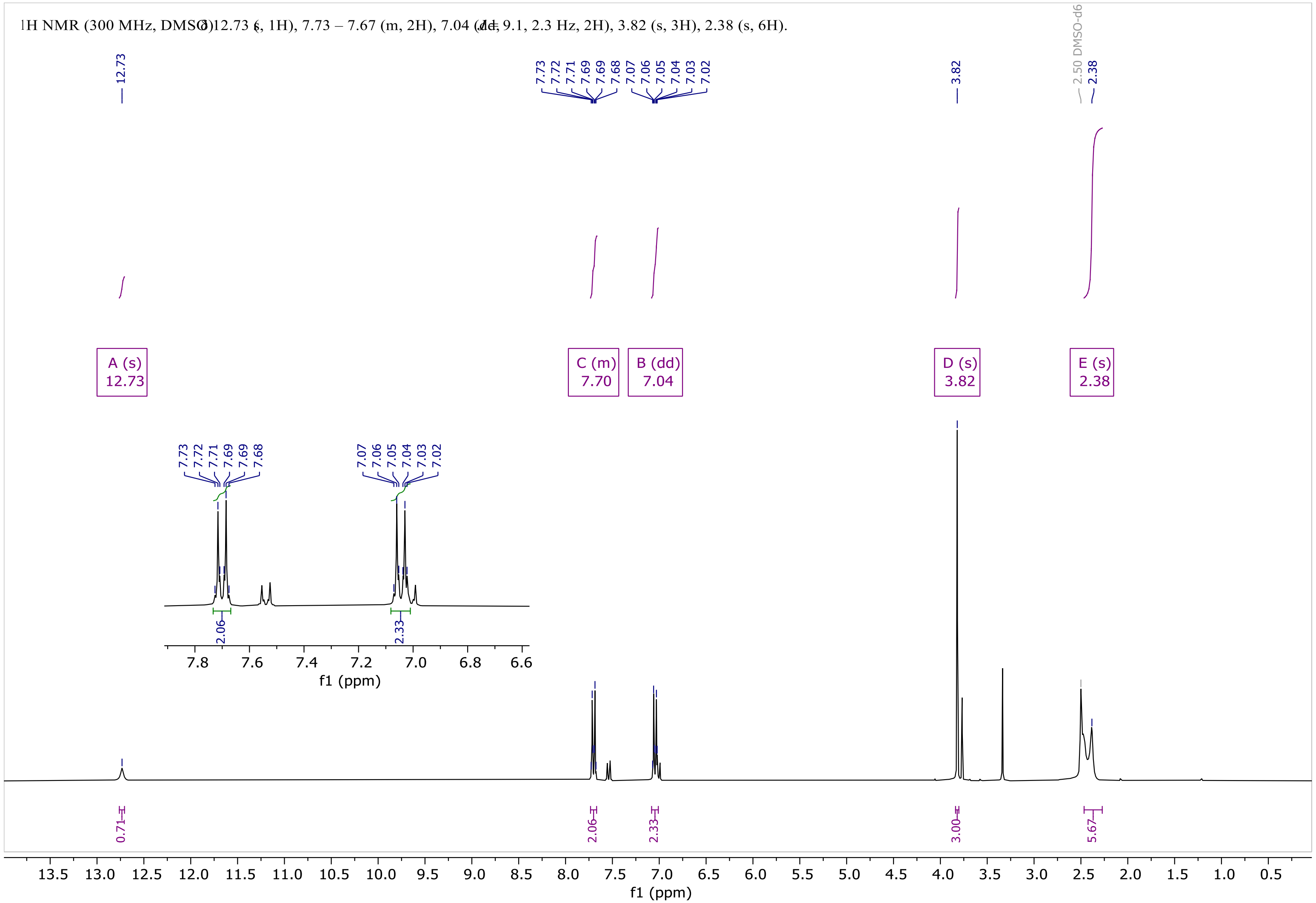

Figure S187: ^{1}H NMR spectrum of (*E*)-4-((4-methoxyphenyl)diazenyl)-3,5-dimethyl-1*H*-pyrazole in DMSO-d_6.

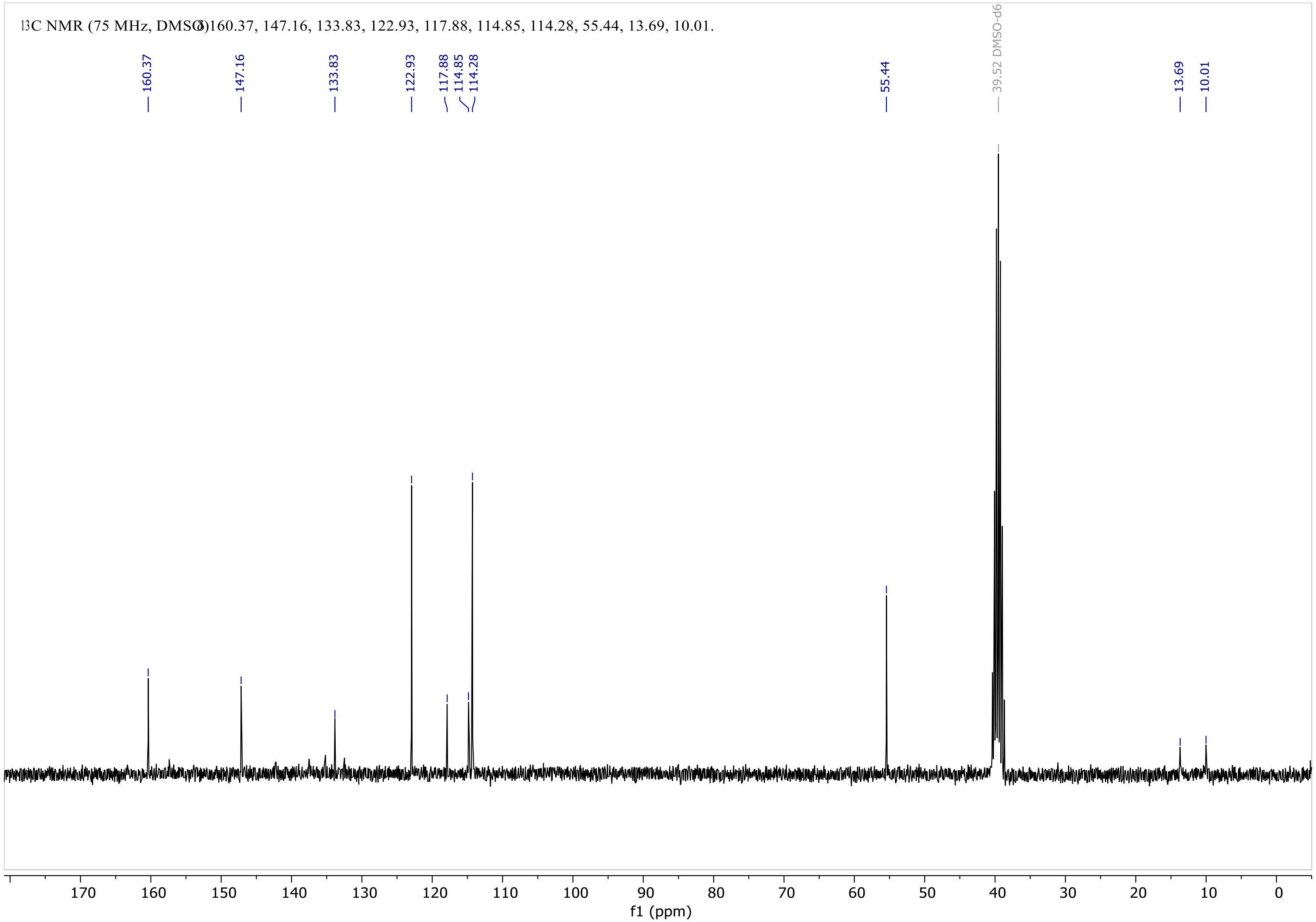

Figure S188: ^{13}C NMR spectrum of (*E*)-4-((4-methoxyphenyl)diazenyl)-3,5-dimethyl-1*H*-pyrazole in DMSO-d_6.

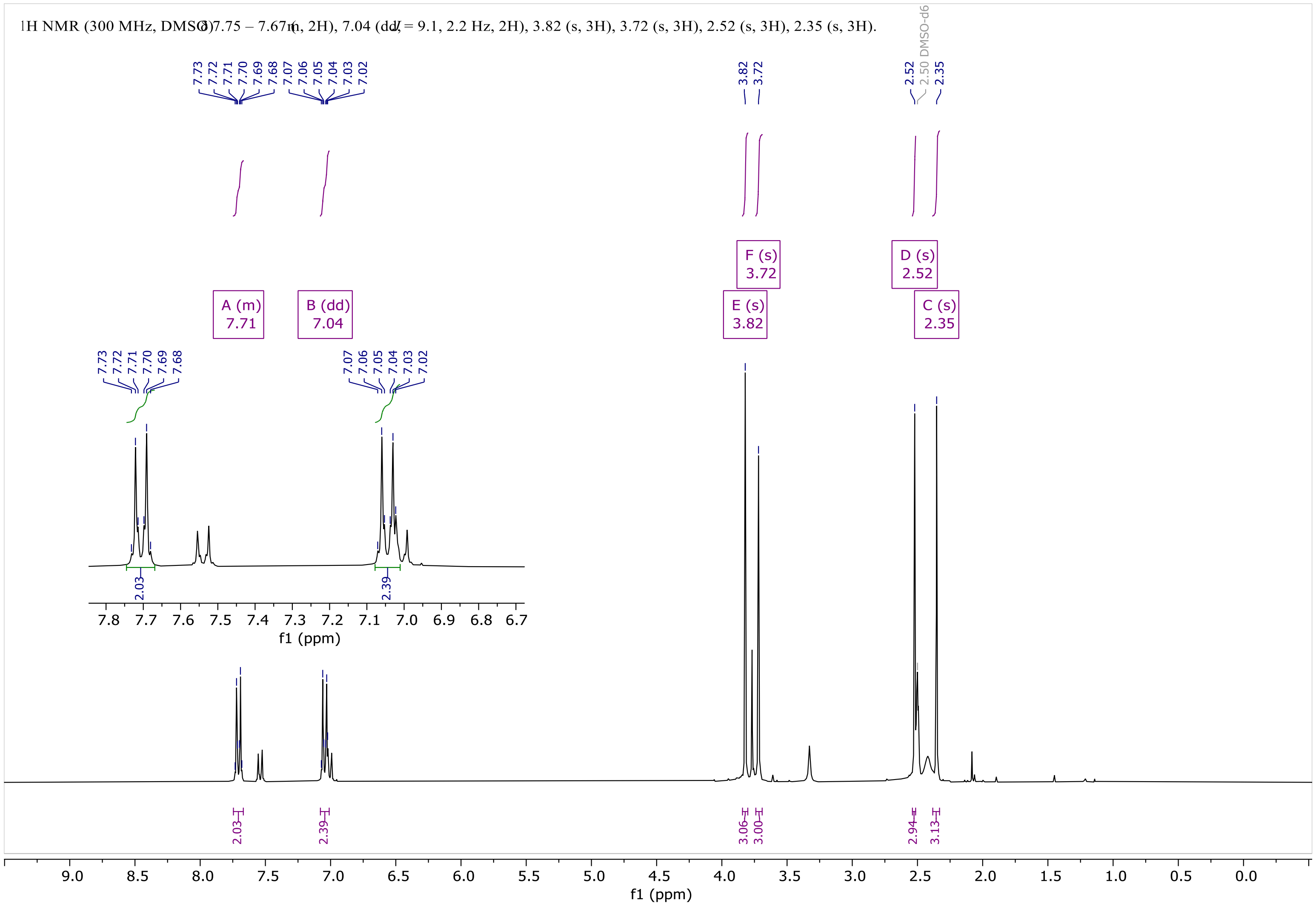

Figure S189: ^{1}H NMR spectrum of (*E*)-4-((4-methoxyphenyl)diazenyl)-1,3,5-trimethyl-1*H*-pyrazole in DMSO-d_6.

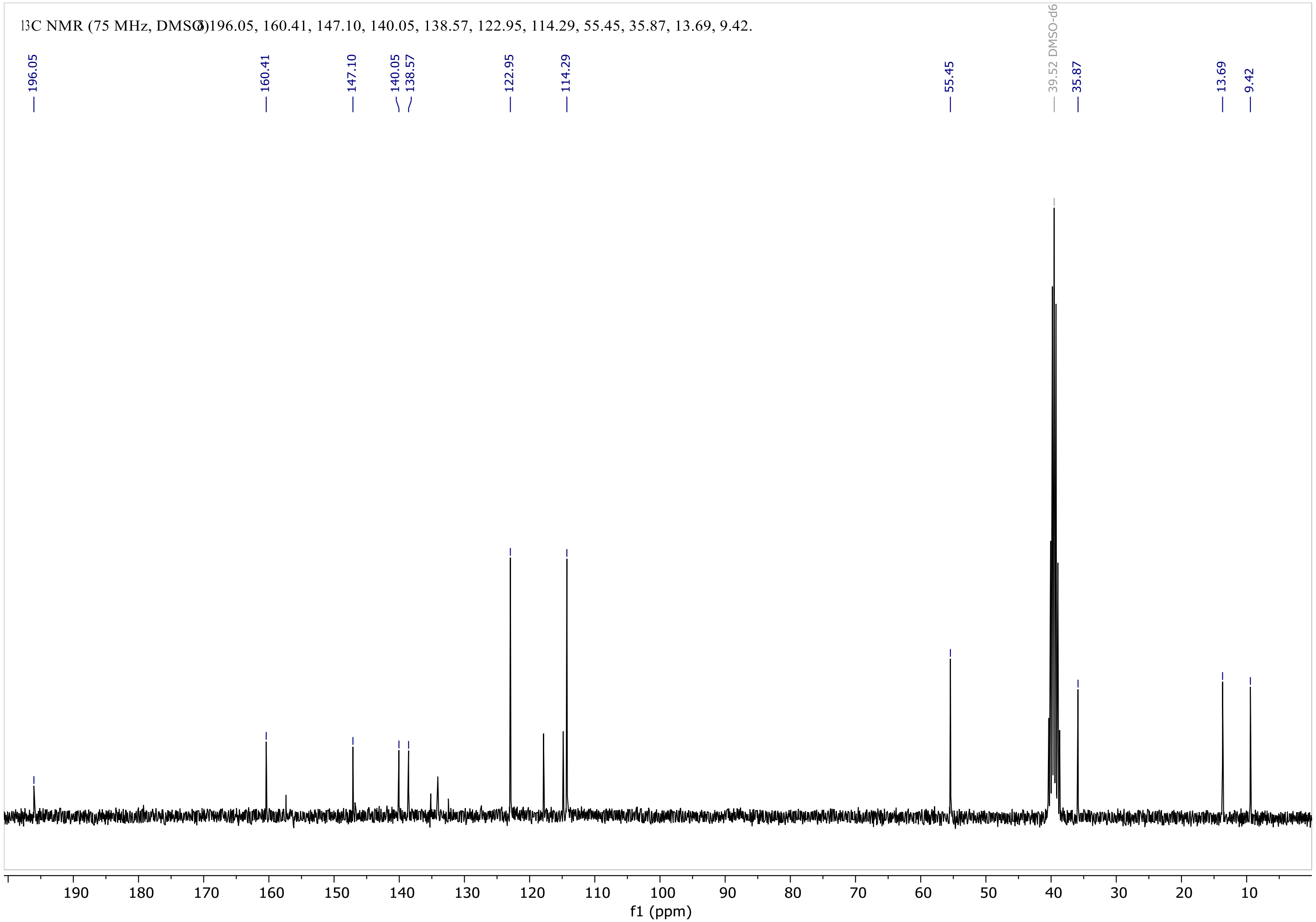

Figure S190: ^{13}C NMR spectrum of (*E*)-4-((4-methoxyphenyl)diazenyl)-1,3,5-trimethyl-1*H*-pyrazole in DMSO-d_6.

1H NMR (400 MHz, DMSO) δ 13.07 (s, 1H), 8.37 – 8.32 (m, 2H), 7.91 – 7.86 (m, 2H), 2.47 (s, 6H).

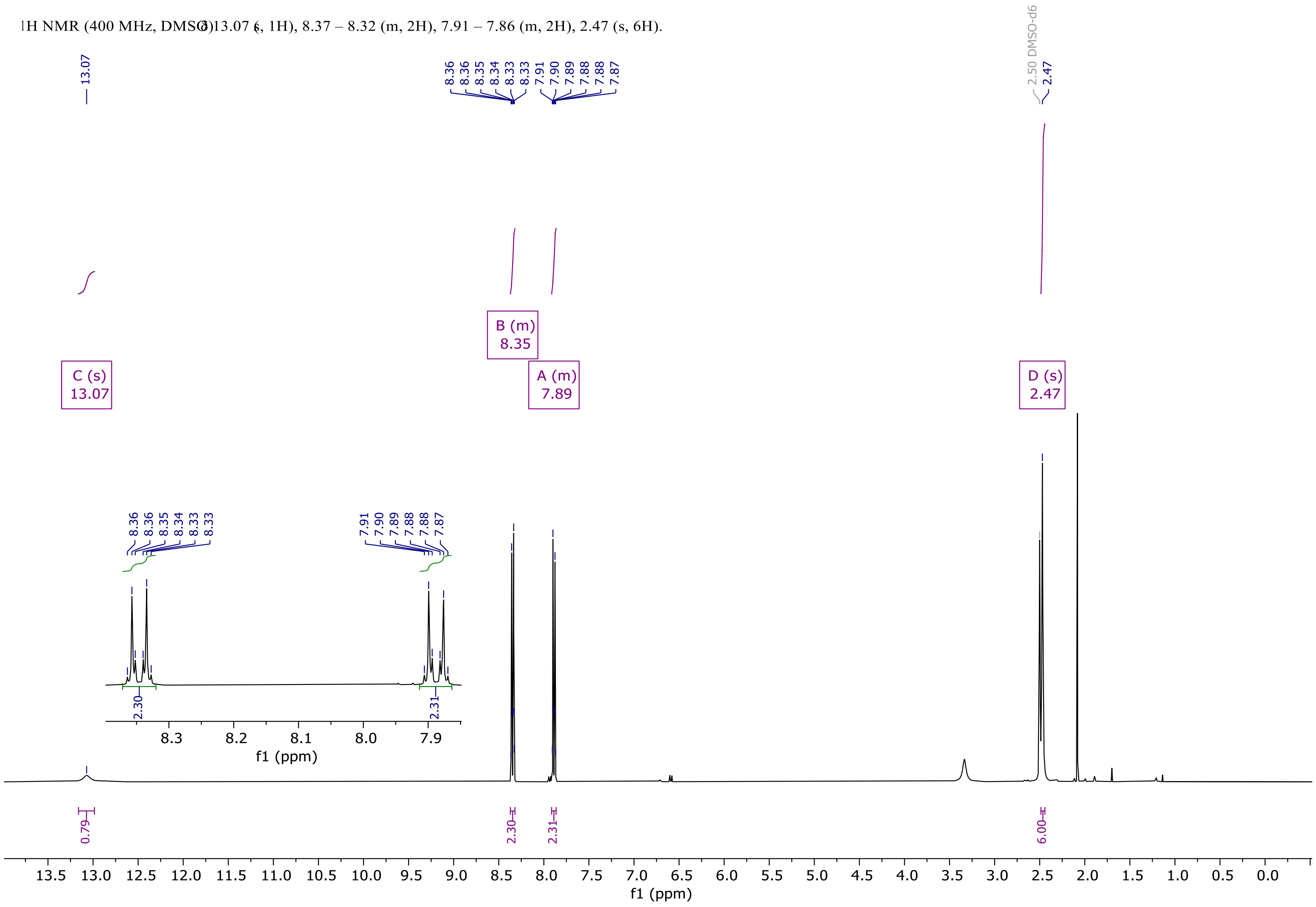

Figure S191: ^{1}H NMR spectrum of (*E*)-3,5-dimethyl-4-((4-nitrophenyl)diazenyl)-1*H*-pyrazole in DMSO-d_6.

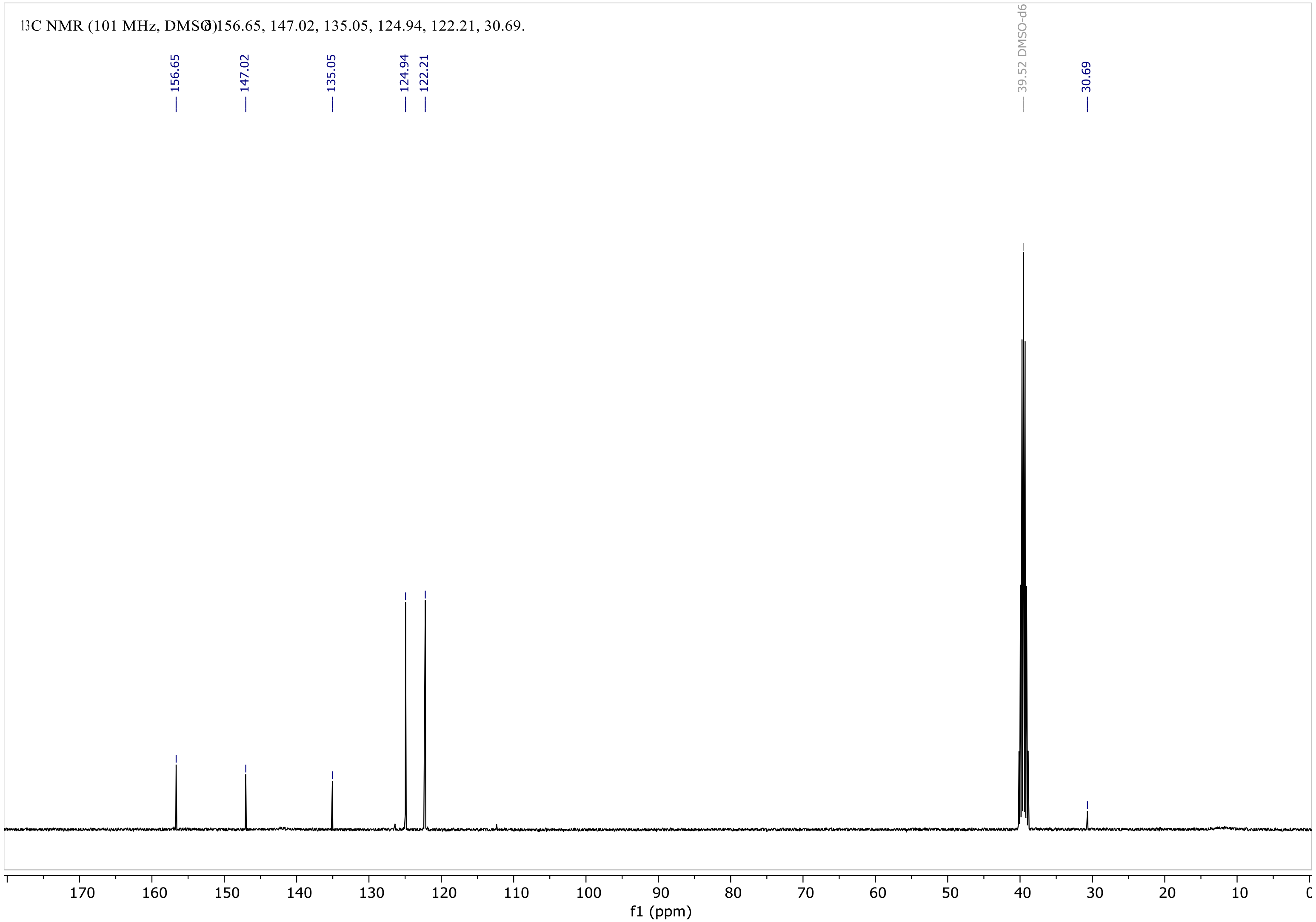

Figure S192: ^{13}C NMR spectrum of (*E*)-3,5-dimethyl-4-((4-nitrophenyl)diazenyl)-1*H*-pyrazole in DMSO-d_6.

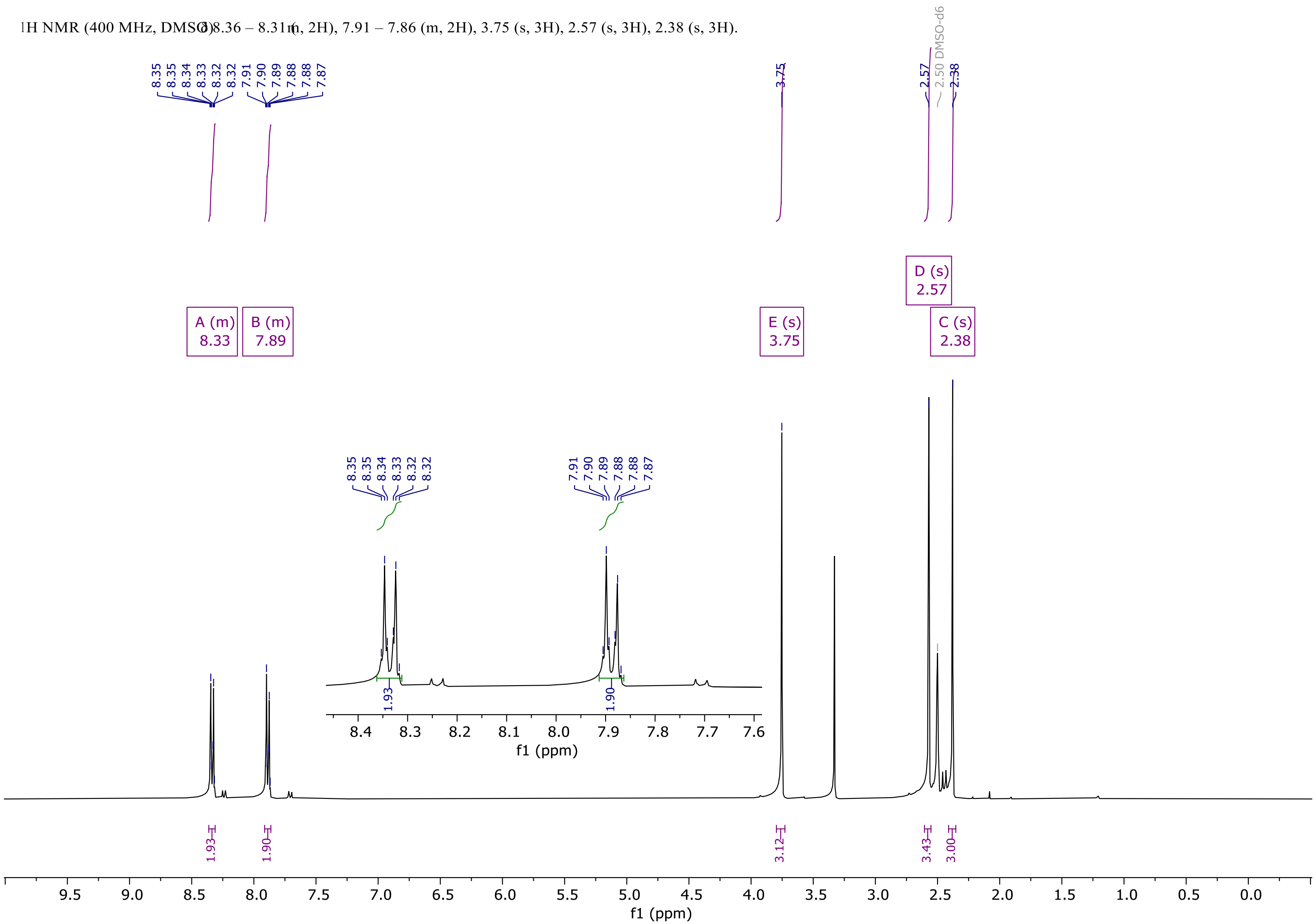

Figure S193: ^{1}H NMR spectrum of (*E*)-1,3,5-trimethyl-4-((4-nitrophenyl)diazenyl)-1*H*-pyrazole in DMSO-d_6.

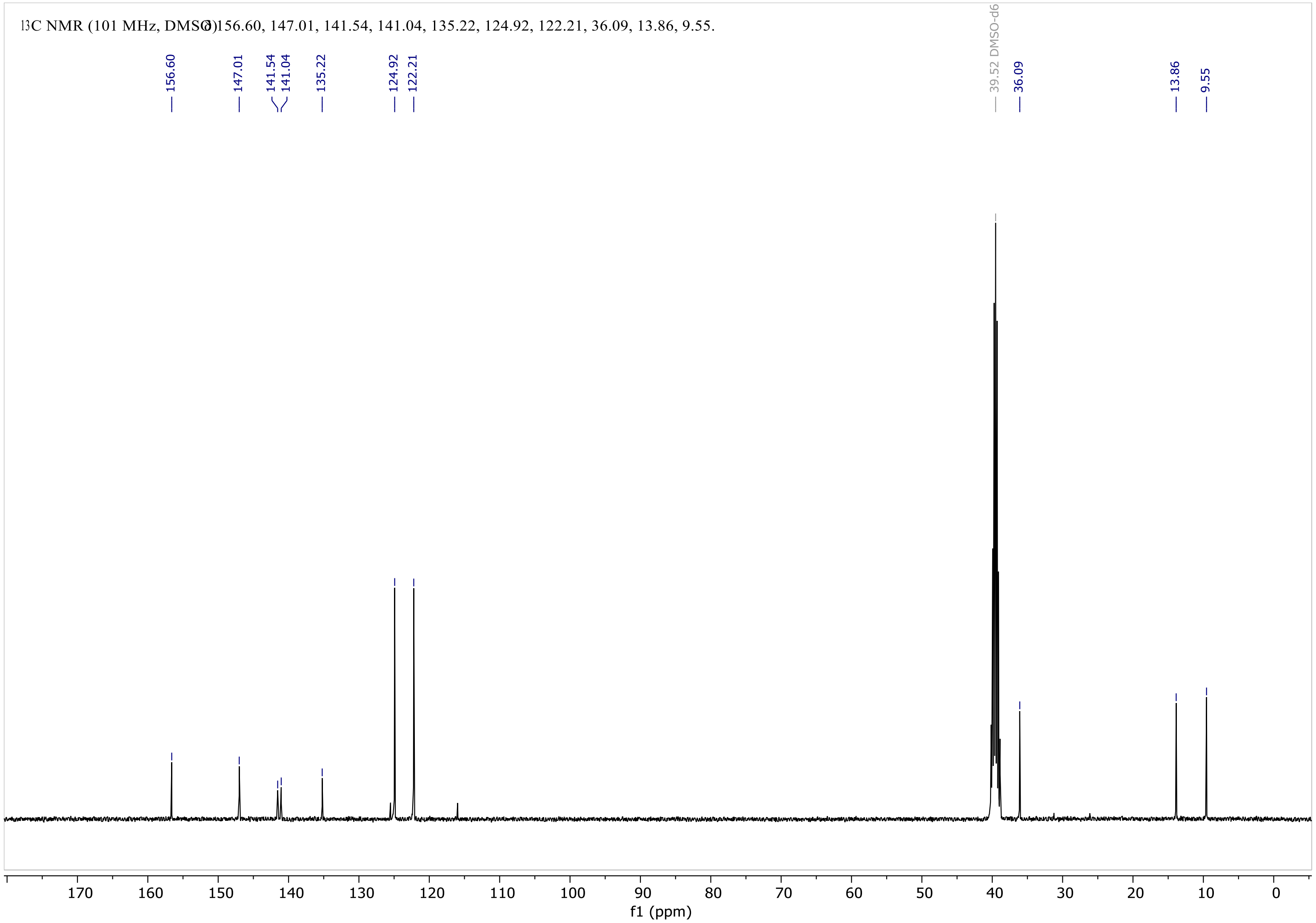

Figure S194: ^{13}C NMR spectrum of (*E*)-1,3,5-trimethyl-4-((4-nitrophenyl)diazenyl)-1*H*-pyrazole in DMSO-d_6.

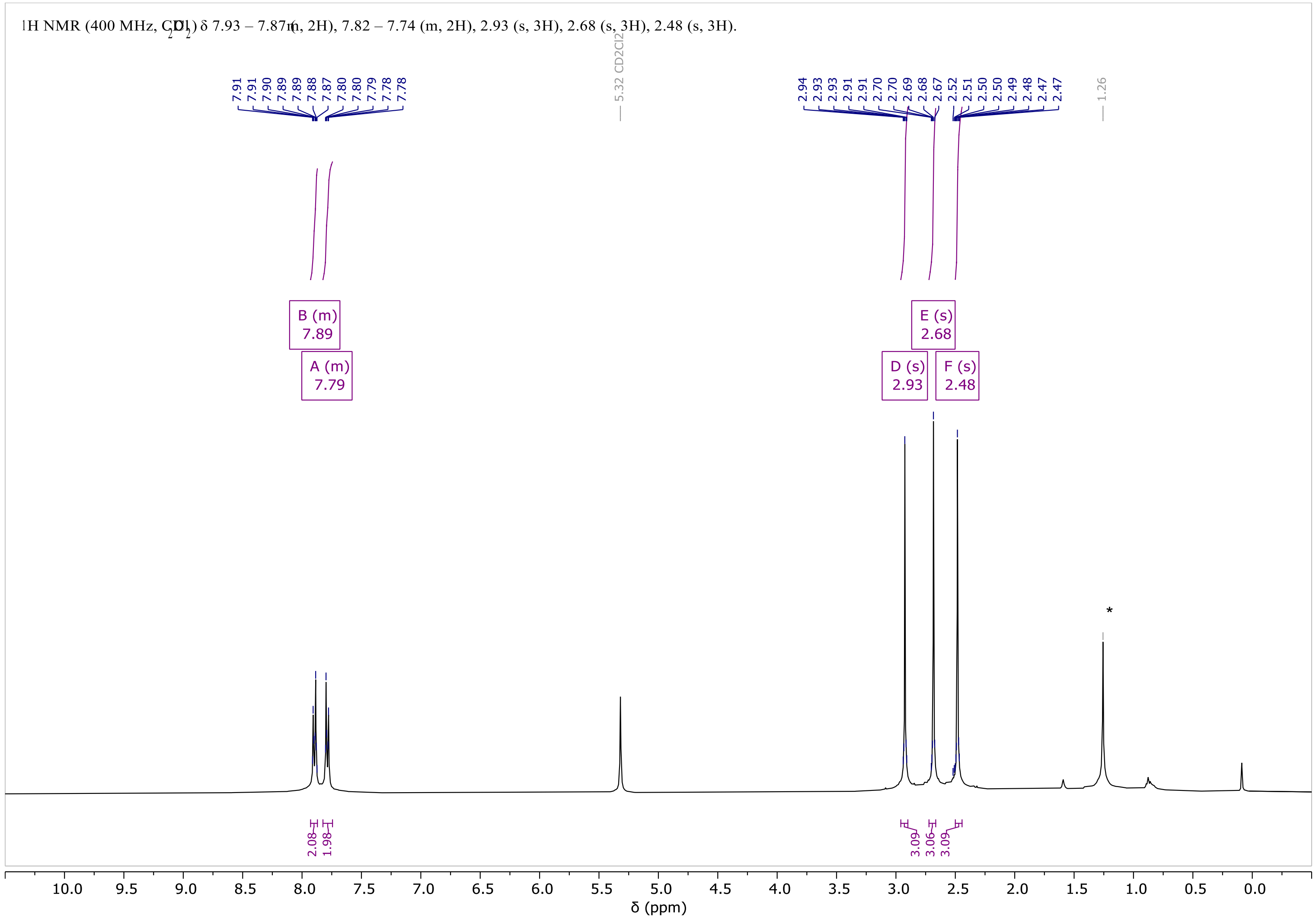

Figure S195: ^{1}H NMR spectrum of (*E*)-4-((1-acetyl-3,5-dimethyl-1*H*-pyrazol-4-yl)diazenyl)benzonitrile in CD_2Cl_2. Asterisk denotes grease.

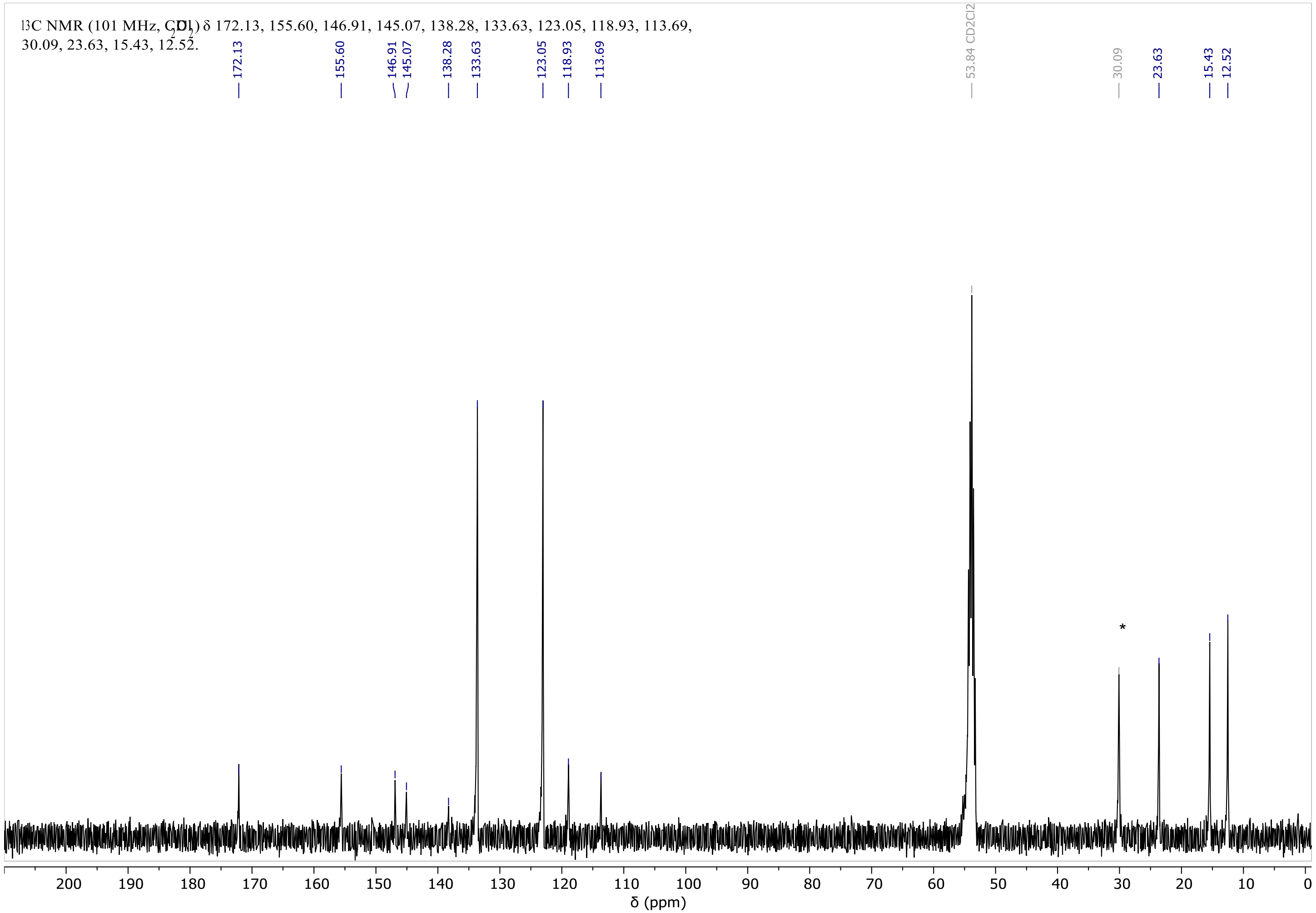

Figure S196: ^{13}C NMR spectrum of (*E*)-4-((1-acetyl-3,5-dimethyl-1*H*-pyrazol-4-yl)diazenyl)benzonitrile in CD_2Cl_2. Asterisk denotes grease.

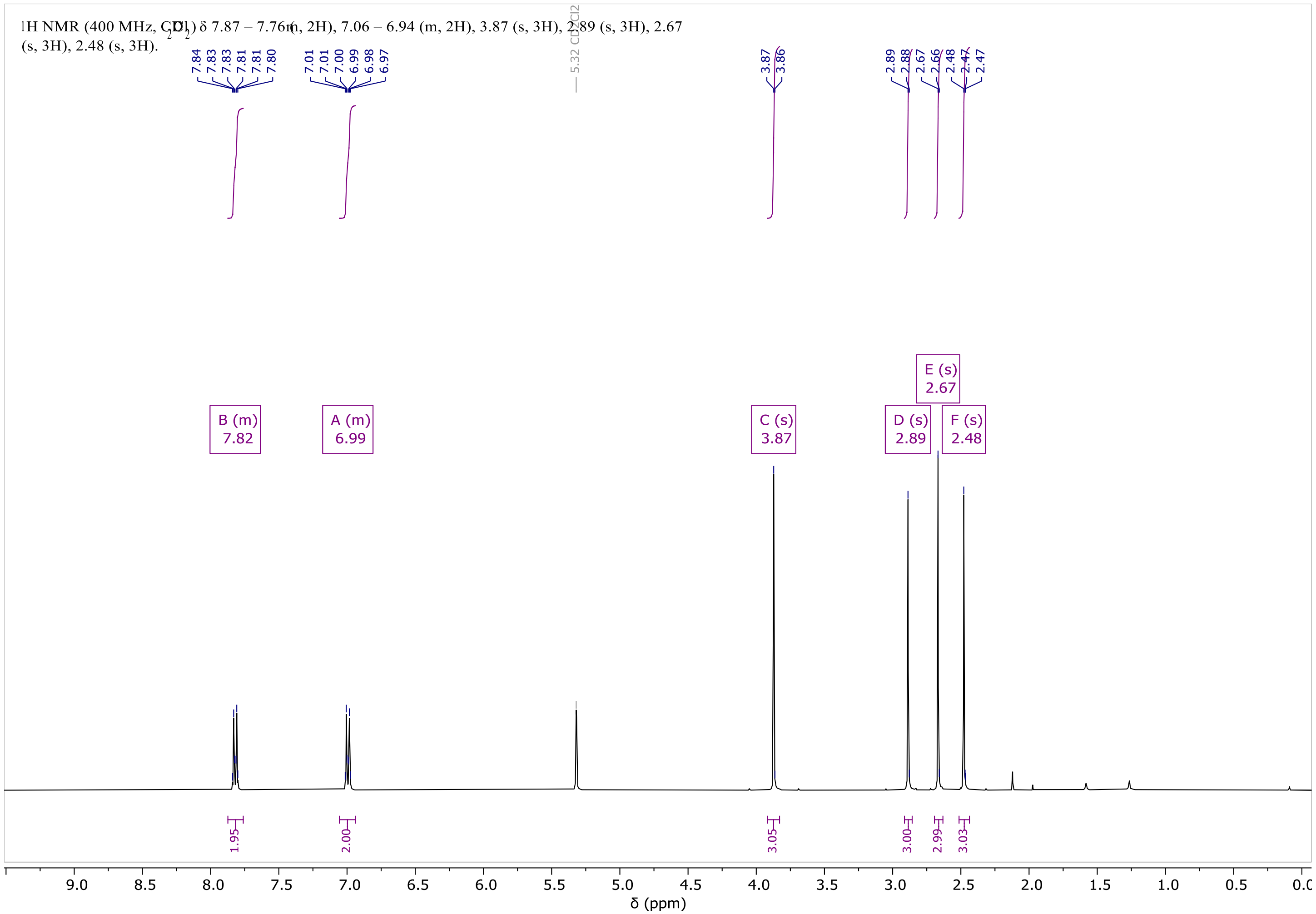

Figure S197: ^{1}H NMR spectrum of (*E*)-1-(4-((4-methoxyphenyl)diazenyl)-3,5-dimethyl-1*H*-pyrazol-1-yl)ethan-1-one in CD_2Cl_2.

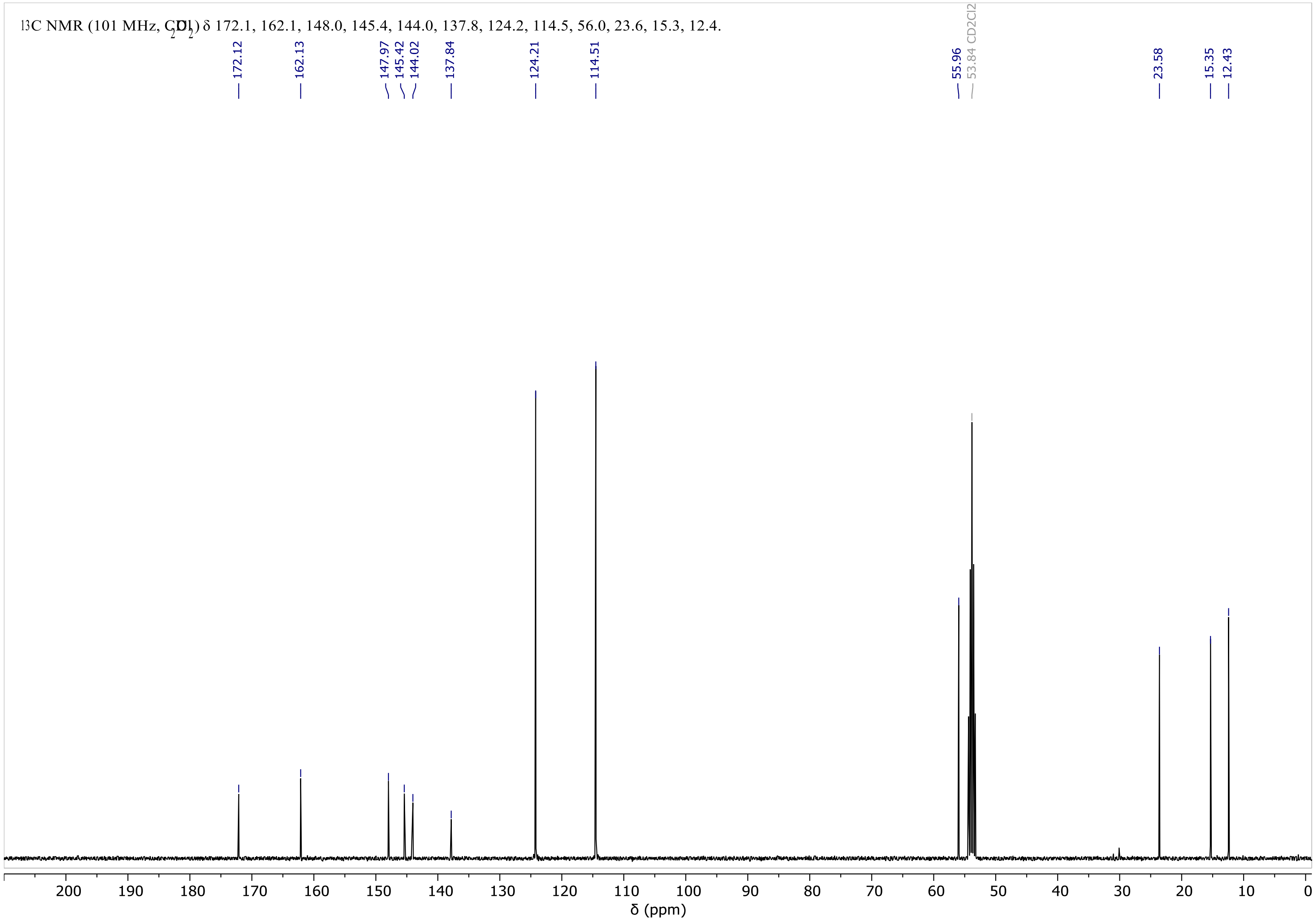

Figure S198: ^{13}C NMR spectrum of (*E*)-1-(4-((4-methoxyphenyl)diazenyl)-3,5-dimethyl-1*H*-pyrazol-1-yl)ethan-1-one in CD_2Cl_2.

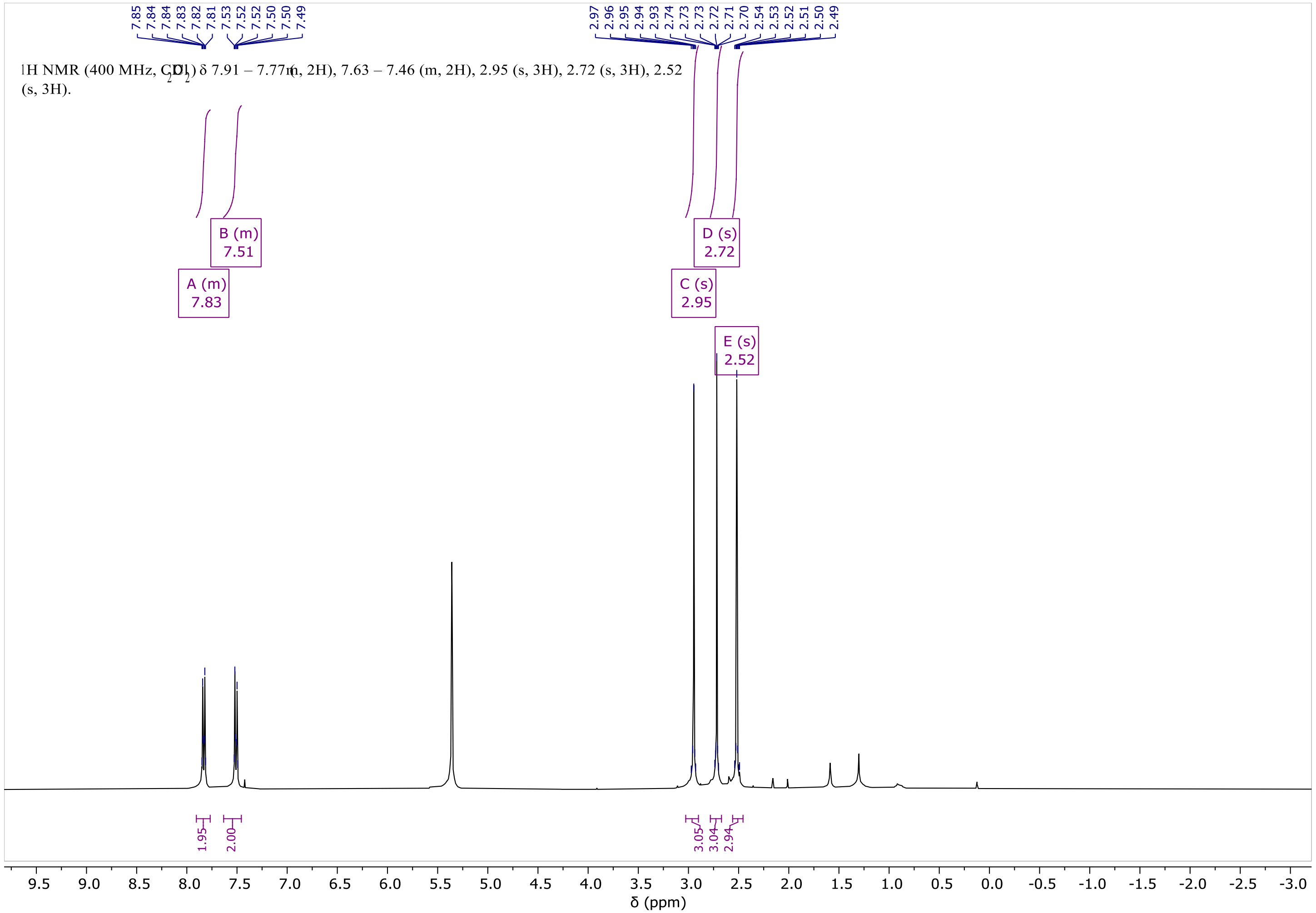

Figure S199: ^{1}H NMR spectrum of (*E*)-1-(4-((4-chlorophenyl)diazenyl)-3,5-dimethyl-1*H*-pyrazol-1-yl)ethan-1-one in CD_2Cl_2.

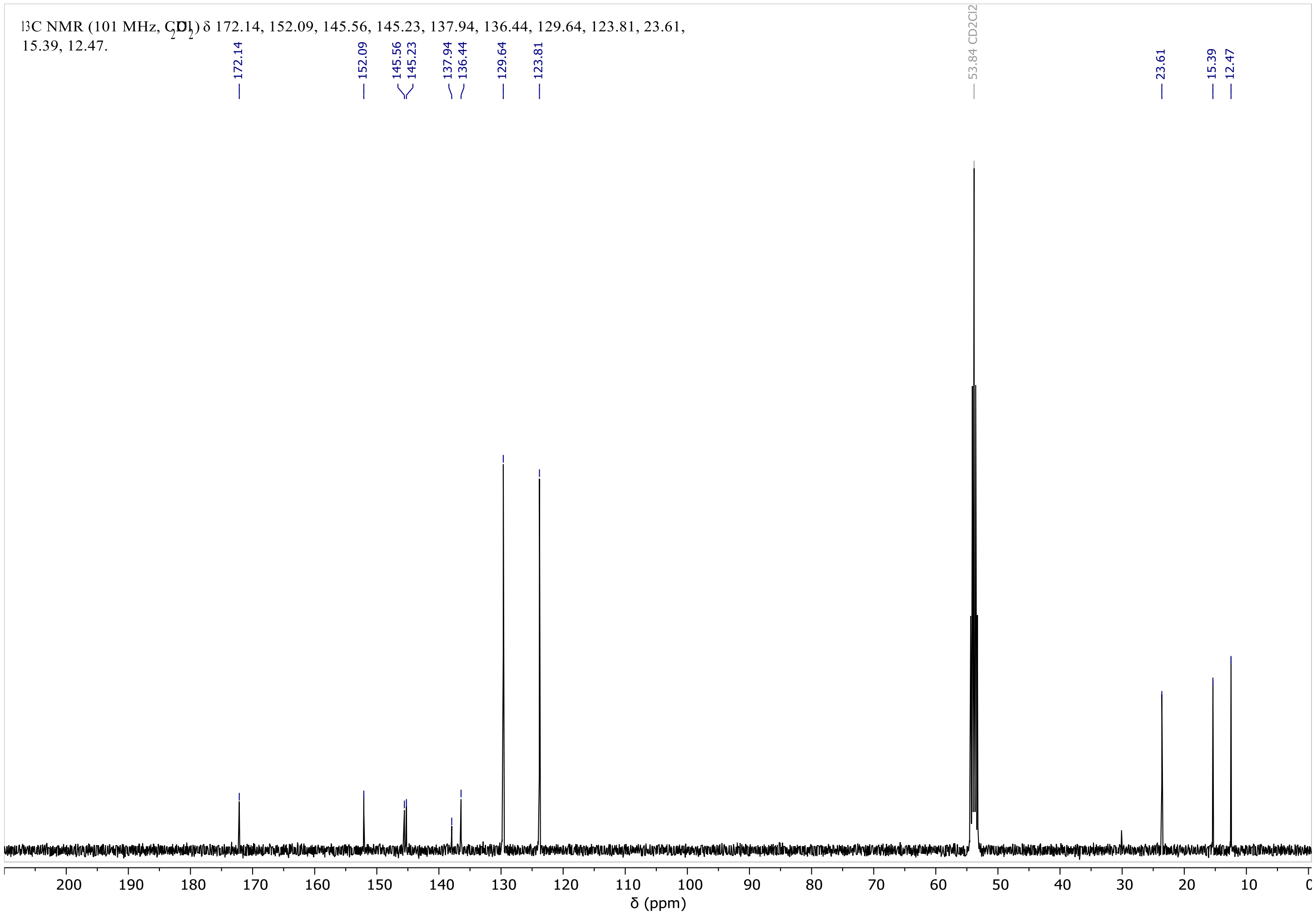

Figure S200: ^{13}C NMR spectrum of (*E*)-1-(4-((4-chlorophenyl)diazenyl)-3,5-dimethyl-1*H*-pyrazol-1-yl)ethan-1-one in CD_2Cl_2.

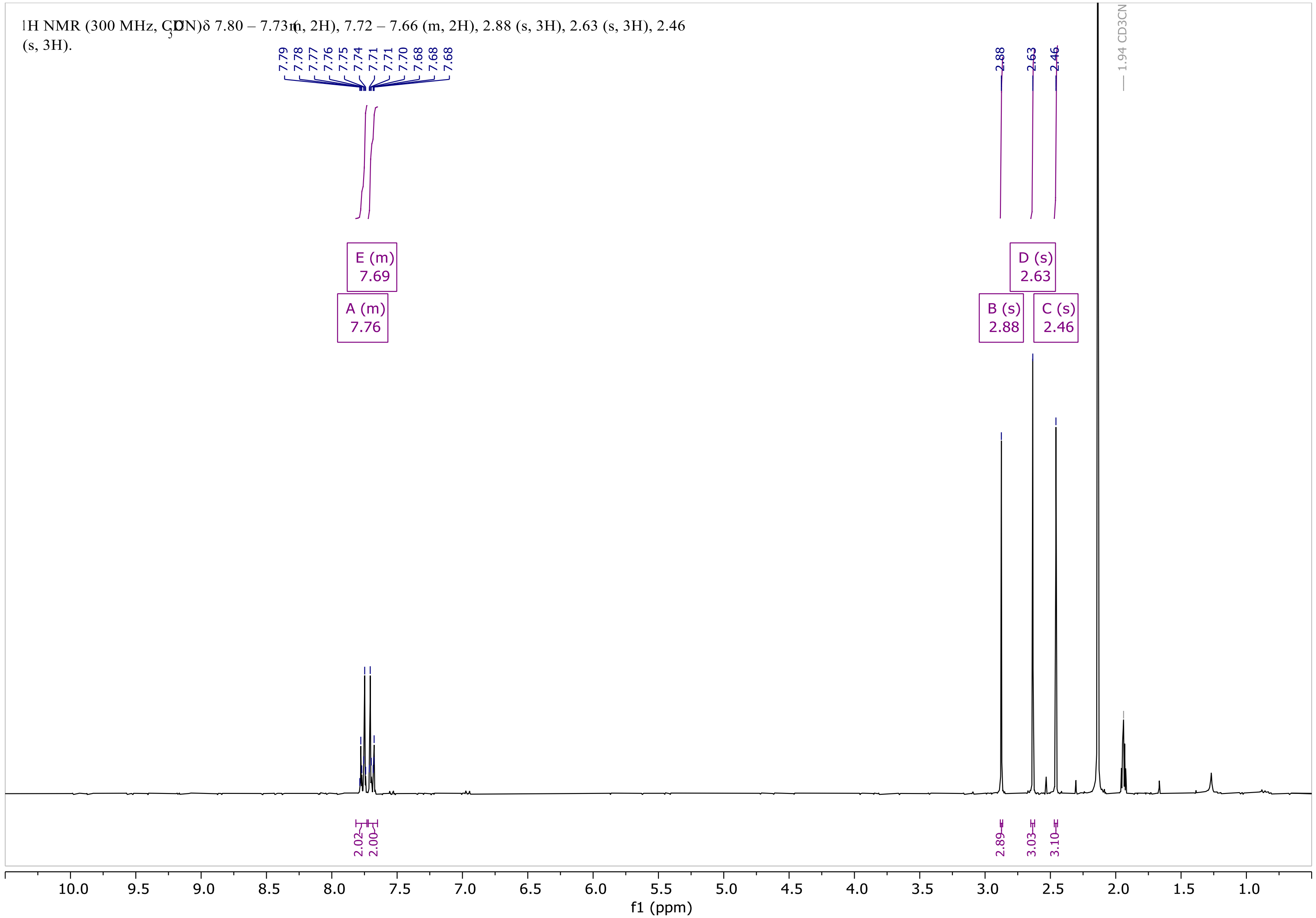

Figure S201: ^{1}H NMR spectrum of (*E*)-1-(4-((4-bromophenyl)diazenyl)-3,5-dimethyl-1*H*-pyrazol-1-yl)ethan-1-one in CD_2Cl_2.

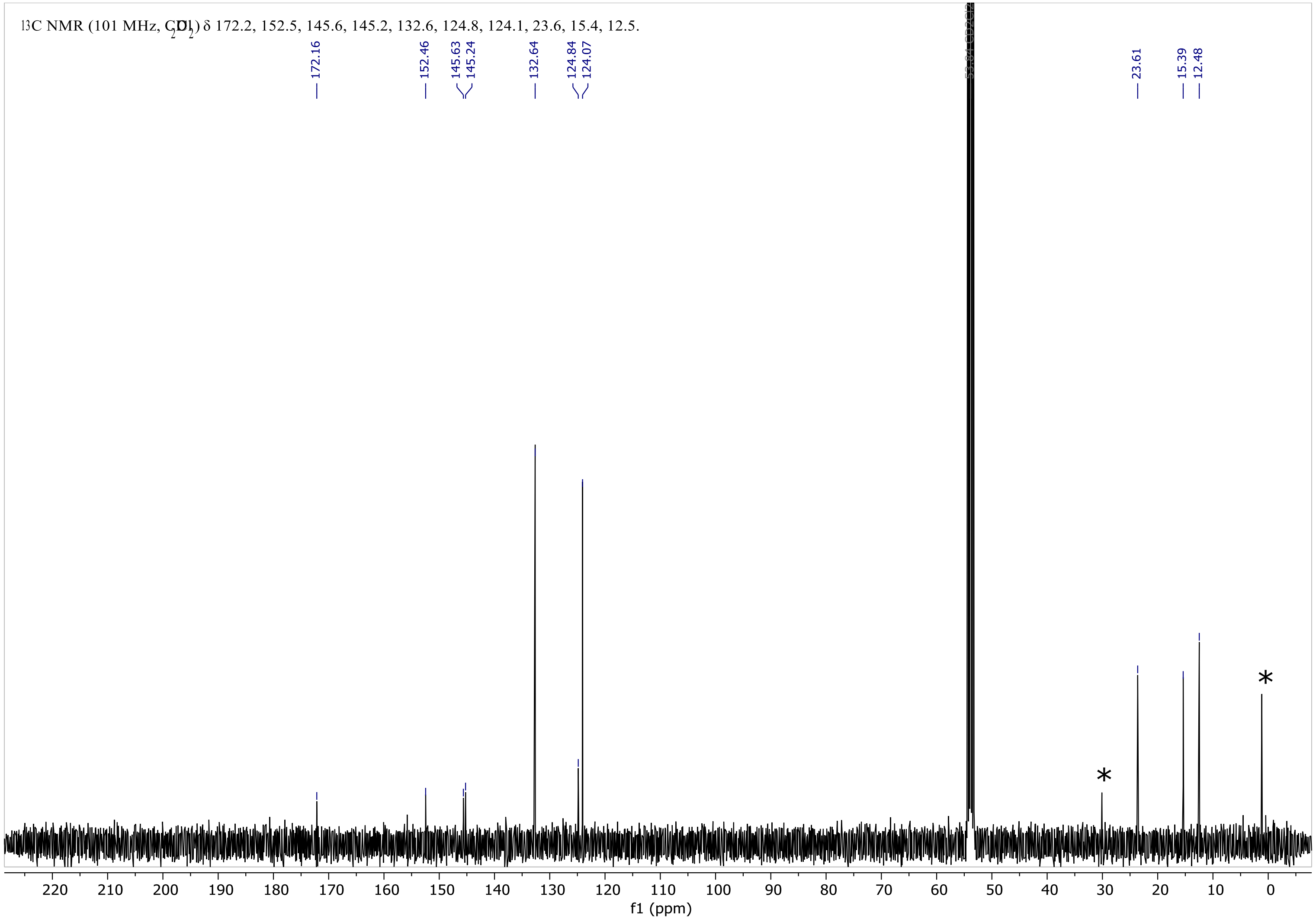

Figure S202: ^{13}C NMR spectrum of (*E*)-1-(4-((4-bromophenyl)diazenyl)-3,5-dimethyl-1*H*-pyrazol-1-yl)ethan-1-one in CD_2Cl_2. Asterisks denote grease.

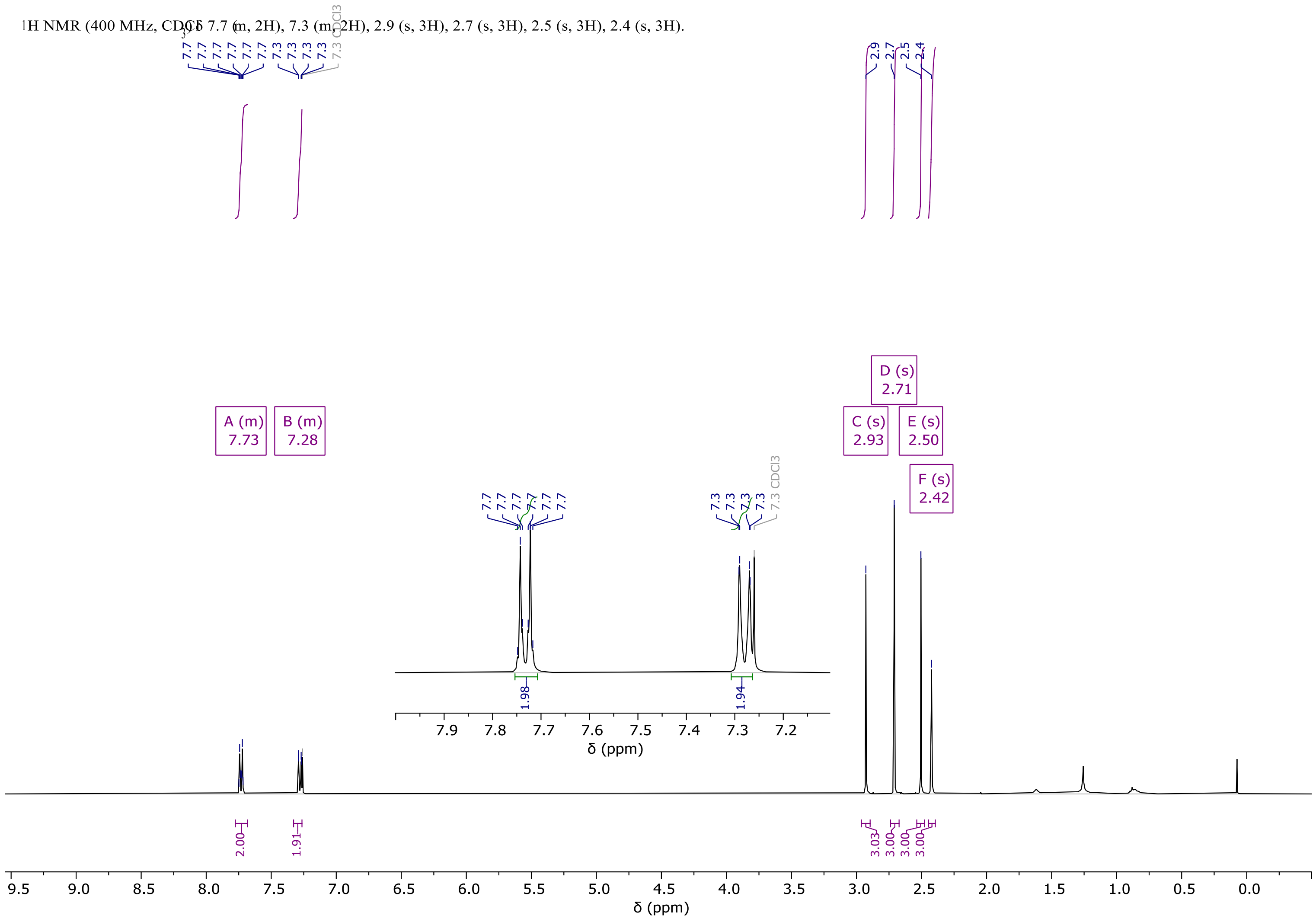

Figure S203: ^{1}H NMR spectrum of (*E*)-1-(3,5-dimethyl-4-(*p*-tolyldiazenyl)-1*H*-pyrazol-1-yl)ethan-1-one in $CDCl_3$.

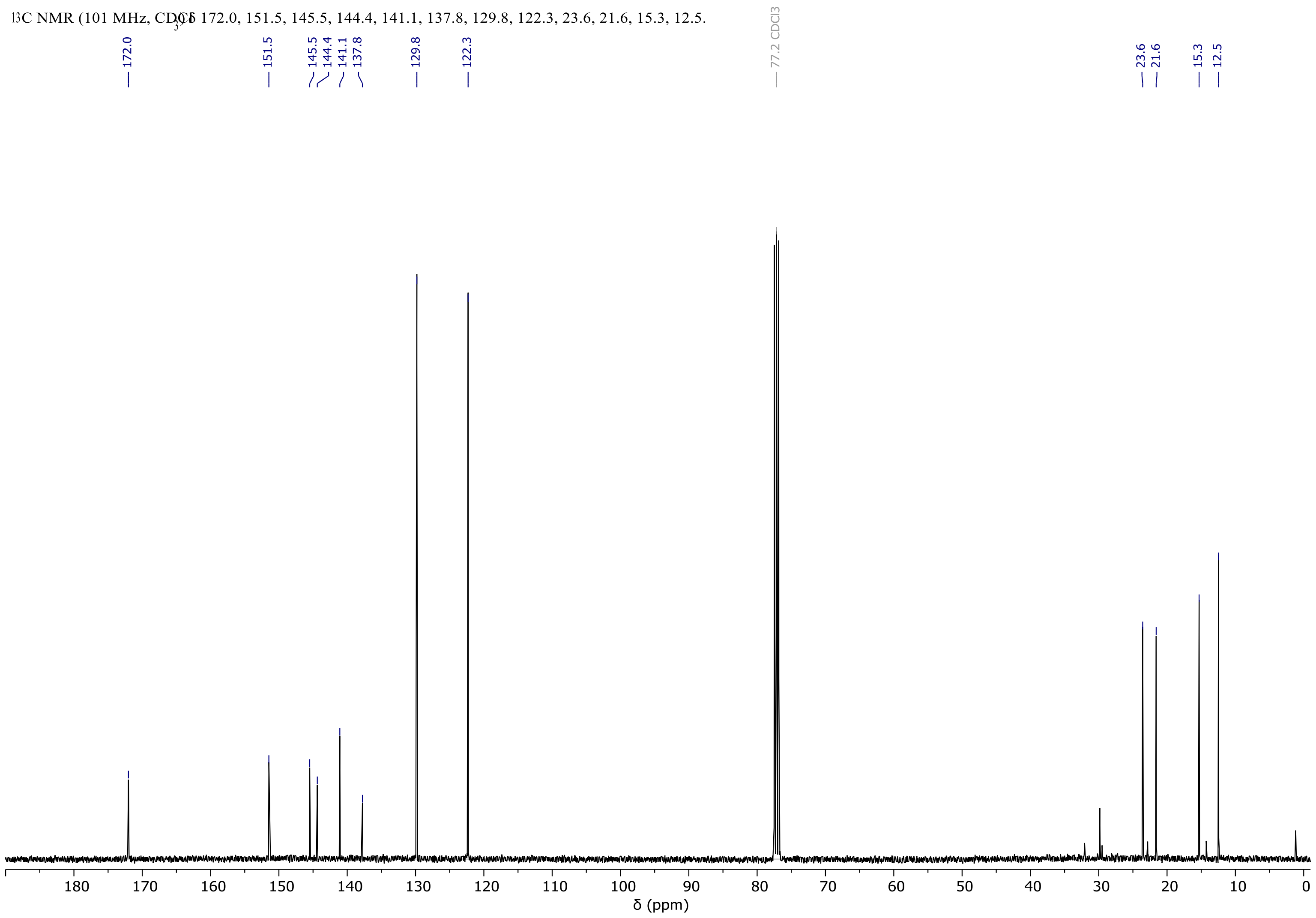

Figure S204: ^{13}C NMR spectrum of (*E*)-1-(3,5-dimethyl-4-(*p*-tolyldiazenyl)-1*H*-pyrazol-1-yl)ethan-1-one in $CDCl_3$.

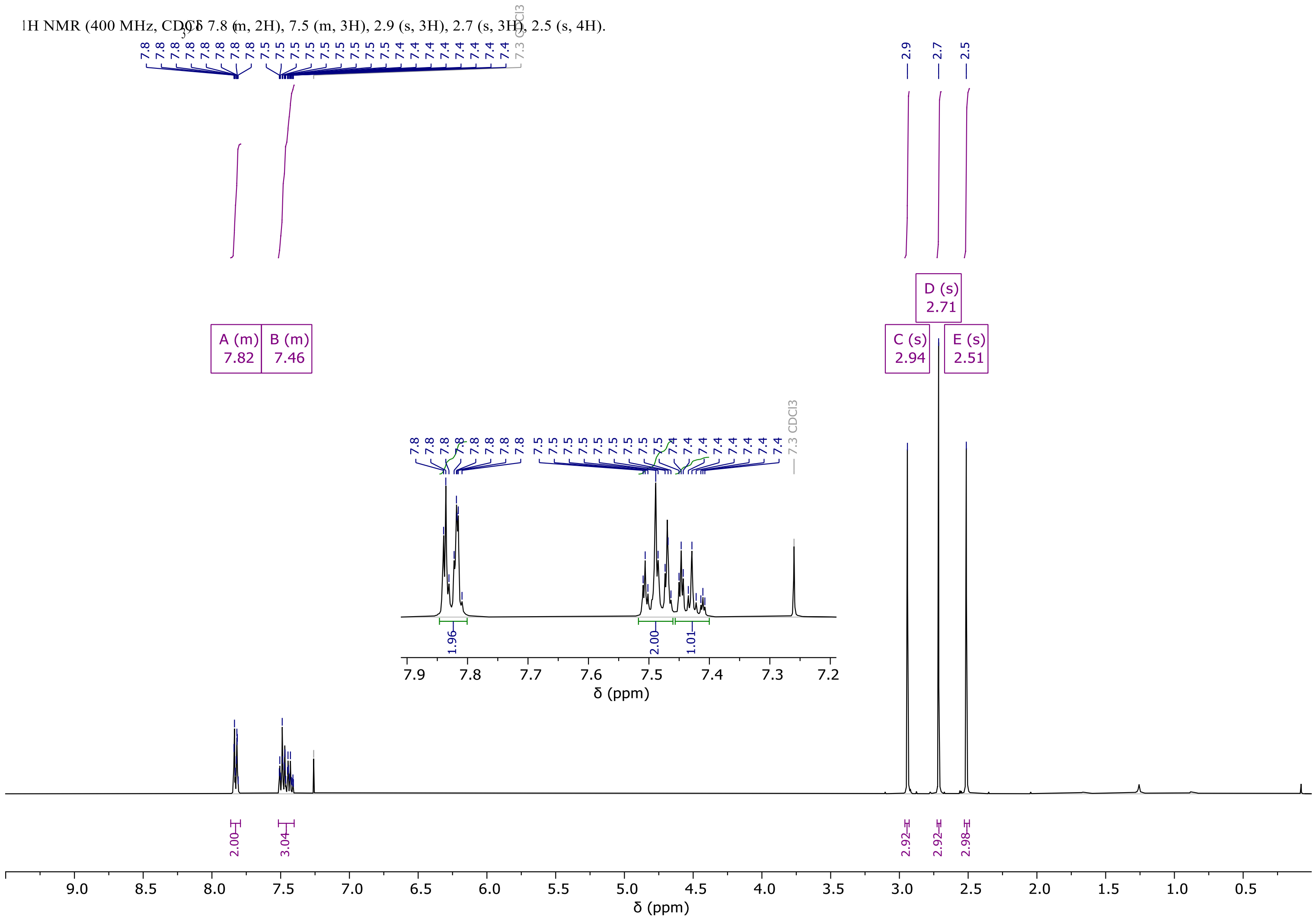

Figure S205: ^{1}H NMR spectrum of (*E*)-1-(3,5-dimethyl-4-(phenyldiazenyl)-1*H*-pyrazol-1-yl)ethan-1-one in $CDCl_3$.

13C NMR (101 MHz, CDCl3) δ 172.0, 153.3, 145.4, 144.9, 137.8, 130.6, 129.2, 122.3, 23.6, 15.3, 12.5.

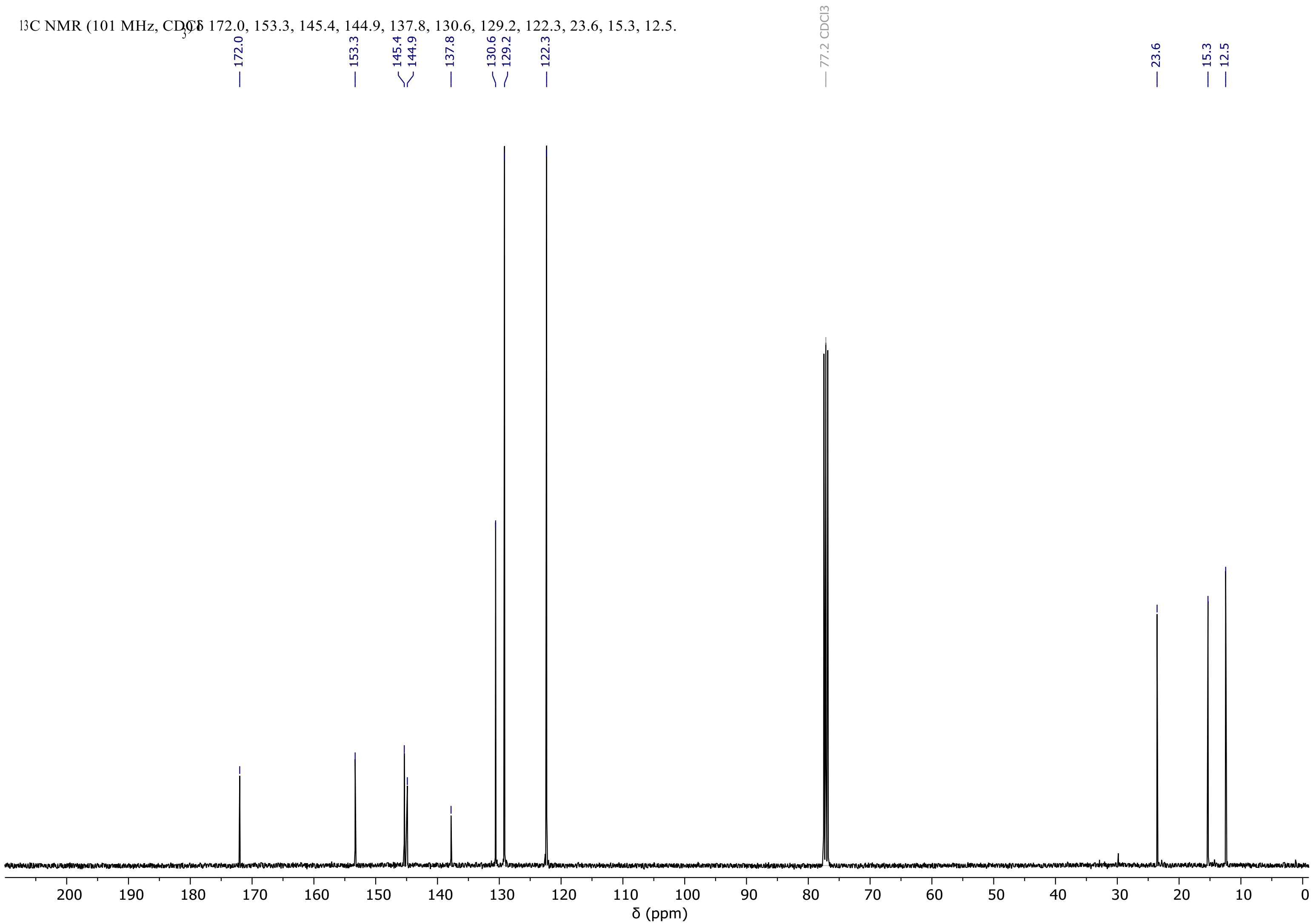

Figure S206: ^{13}C NMR spectrum of (*E*)-1-(3,5-dimethyl-4-(phenyldiazenyl)-1*H*-pyrazol-1-yl)ethan-1-one in $CDCl_3$.

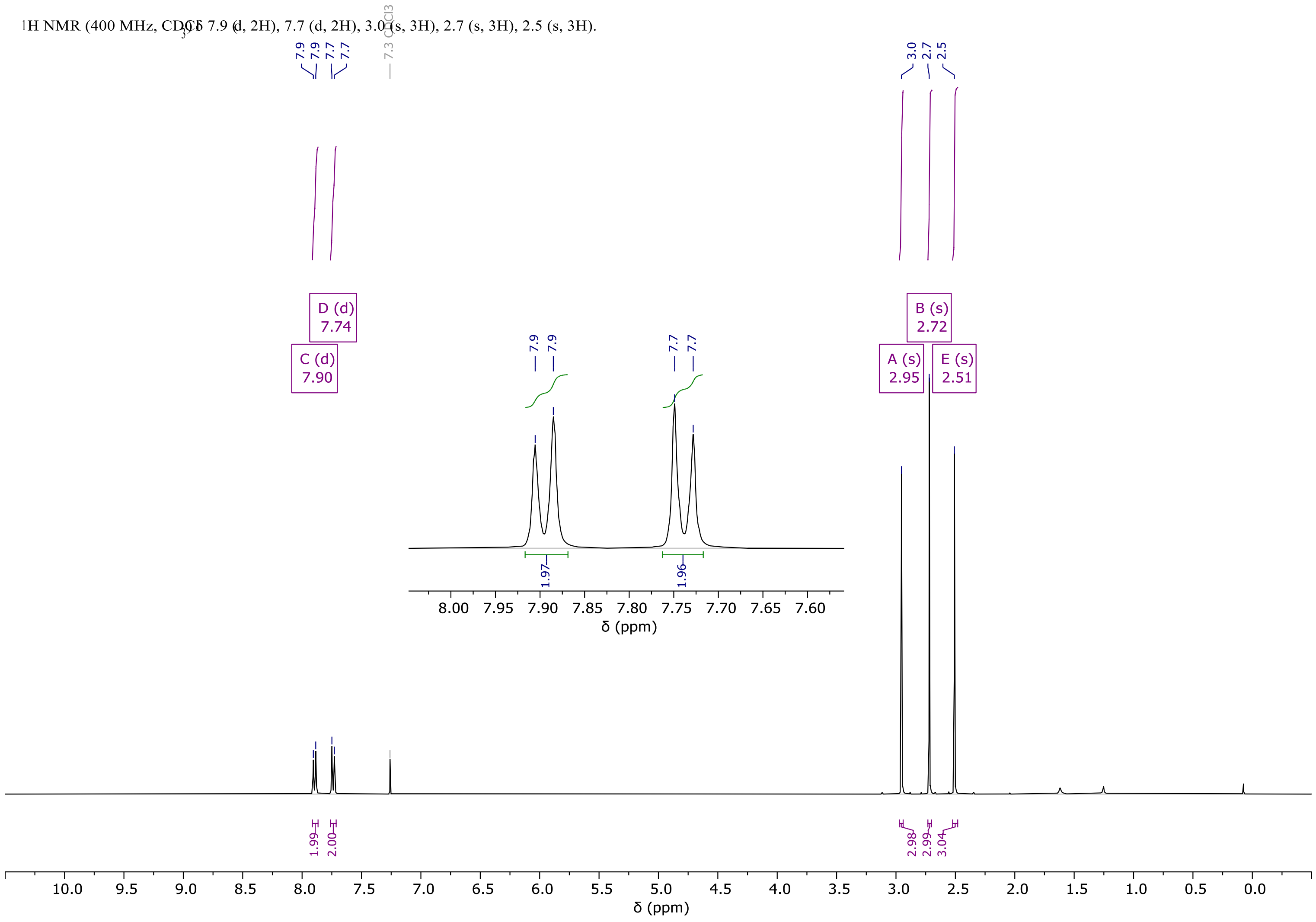

Figure S207: ^{1}H NMR spectrum of (*E*)-1-(3,5-dimethyl-4-((4-(trifluoromethyl)phenyl)diazenyl)-1*H*-pyrazol-1-yl)ethan-1-one in $CDCl_3$.

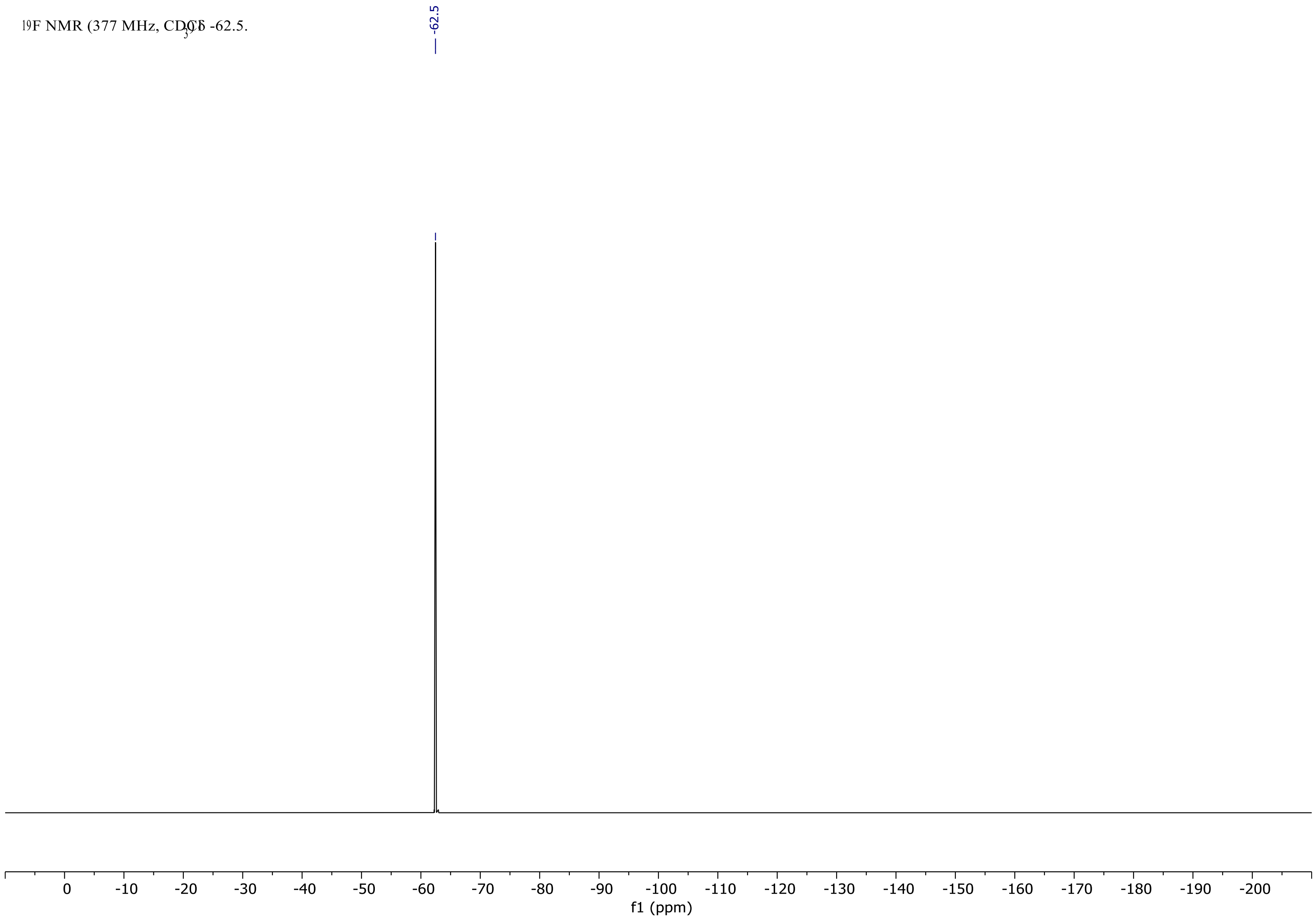

Figure S208: ^{19}F NMR spectrum of (*E*)-1-(3,5-dimethyl-4-((4-(trifluoromethyl)phenyl)diazenyl)-1*H*-pyrazol-1-yl)ethan-1-one in $CDCl_3$.

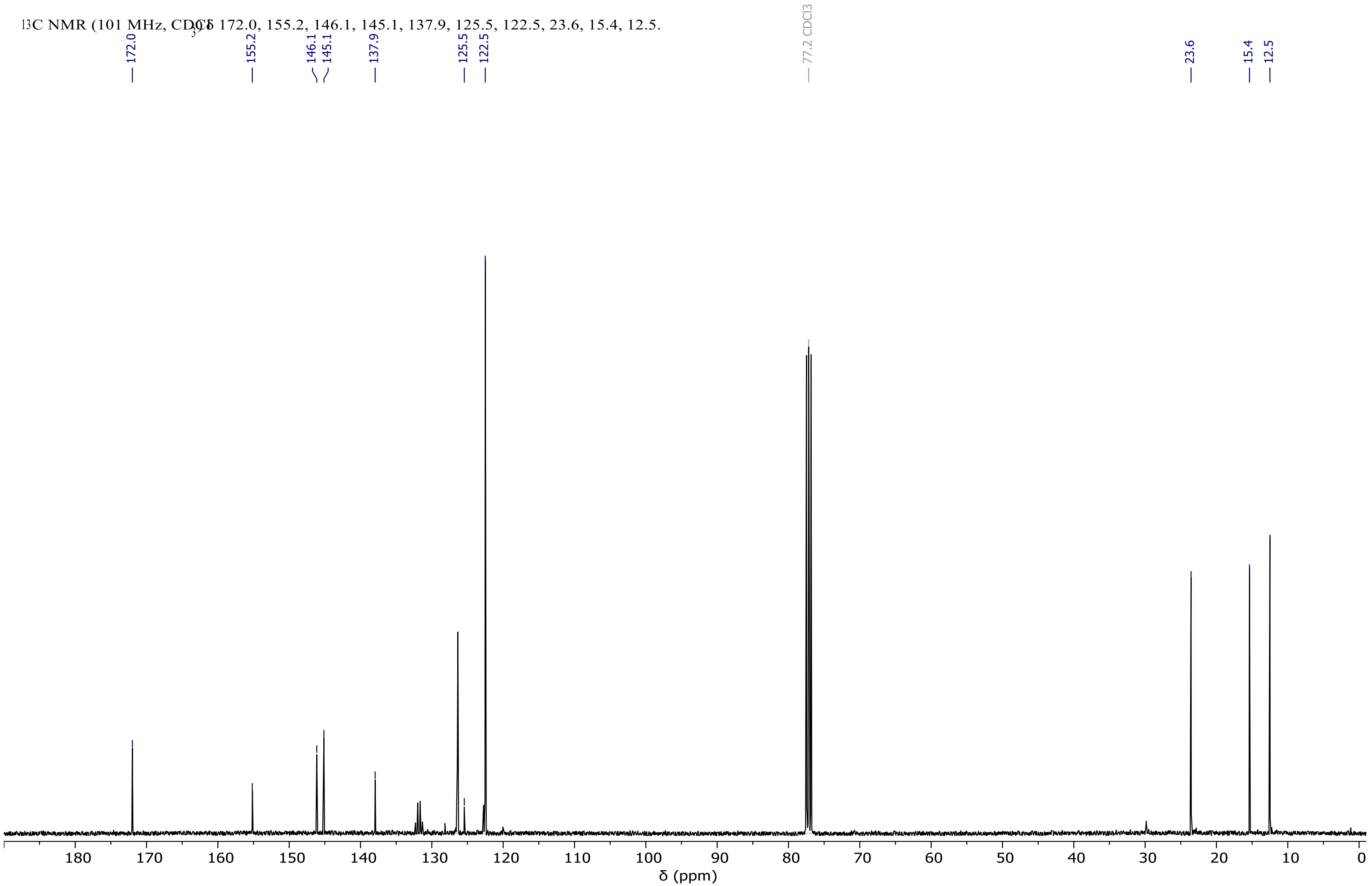

Figure S209: ^{13}C NMR spectrum of (*E*)-1-(3,5-dimethyl-4-((4-(trifluoromethyl)phenyl)diazenyl)-1*H*-pyrazol-1-yl)ethan-1-one in $CDCl_3$.

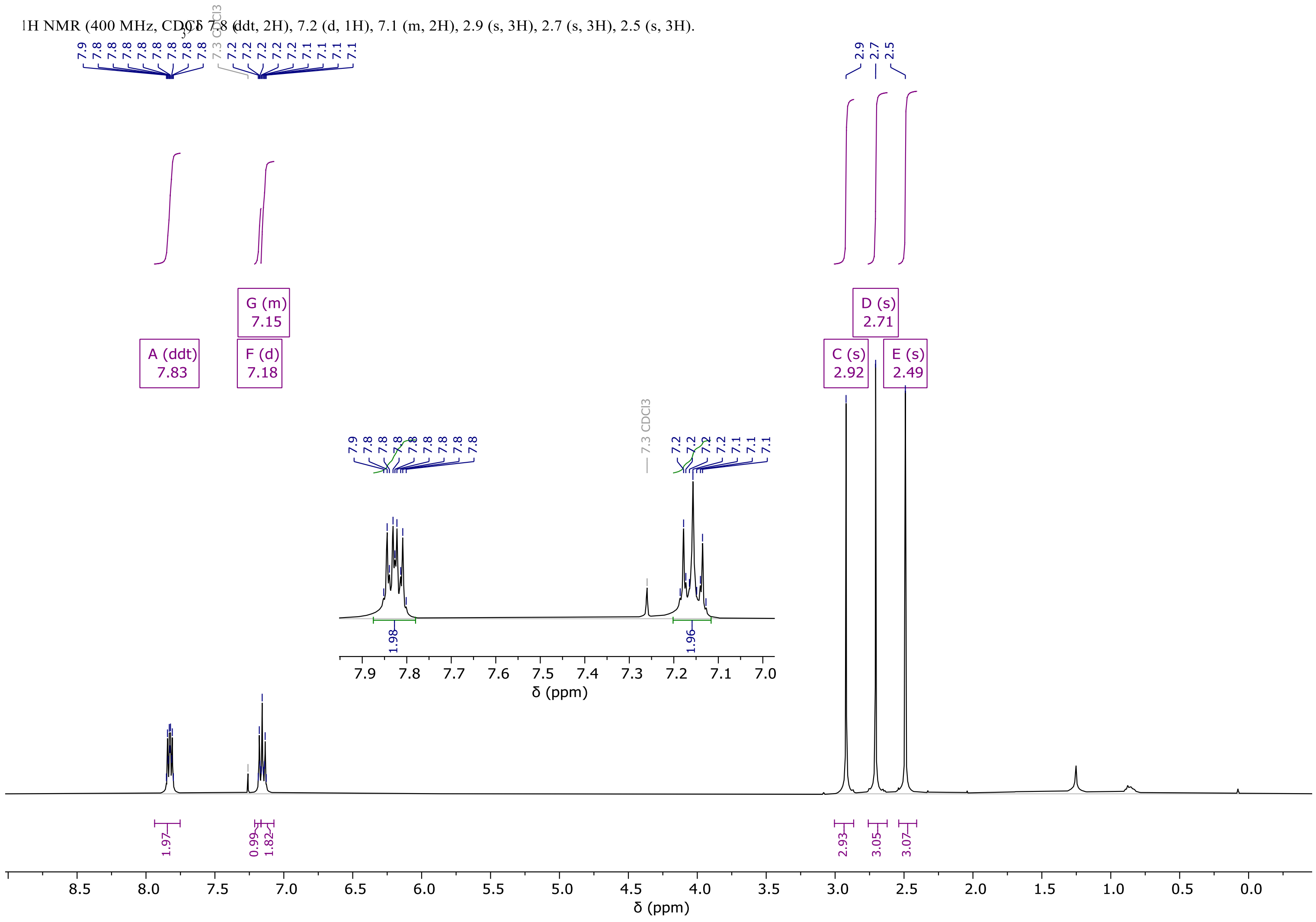

Figure S210: ^{1}H NMR spectrum of (*E*)-1-(4-((4-fluorophenyl)diazenyl)-3,5-dimethyl-1*H*-pyrazol-1-yl)ethan-1-one in $CDCl_3$.

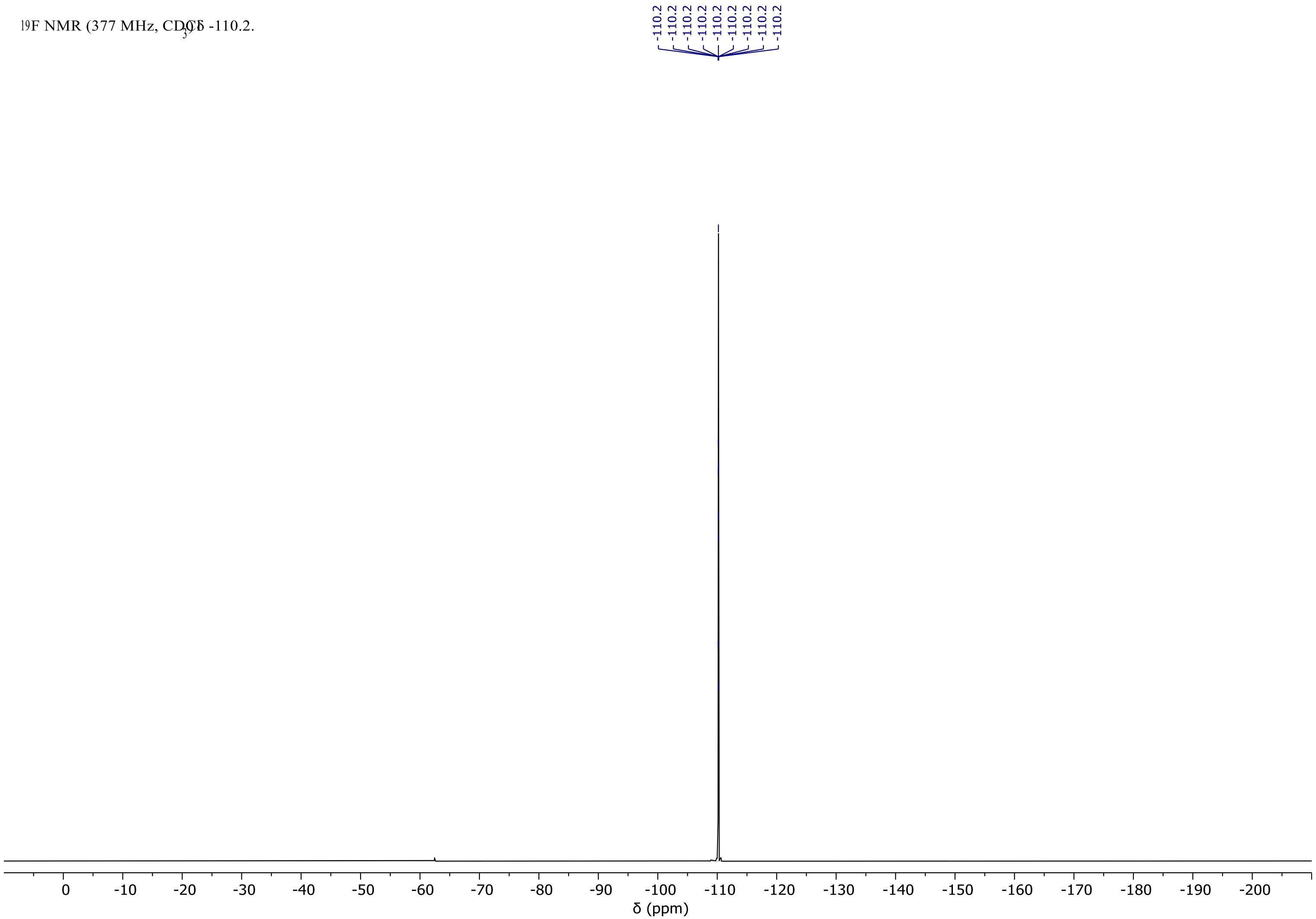

Figure S211: ^{19}F NMR spectrum of (*E*)-1-(4-((4-fluorophenyl)diazenyl)-3,5-dimethyl-1*H*-pyrazol-1-yl)ethan-1-one in $CDCl_3$.

13C NMR (101 MHz, $CDCl_3$) δ 172.0, 165.4, 162.9, 149.9, 145.3, 144.8, 137.6, 124.2, 124.2, 116.2, 116.0, 23.5, 15.3, 12.4.

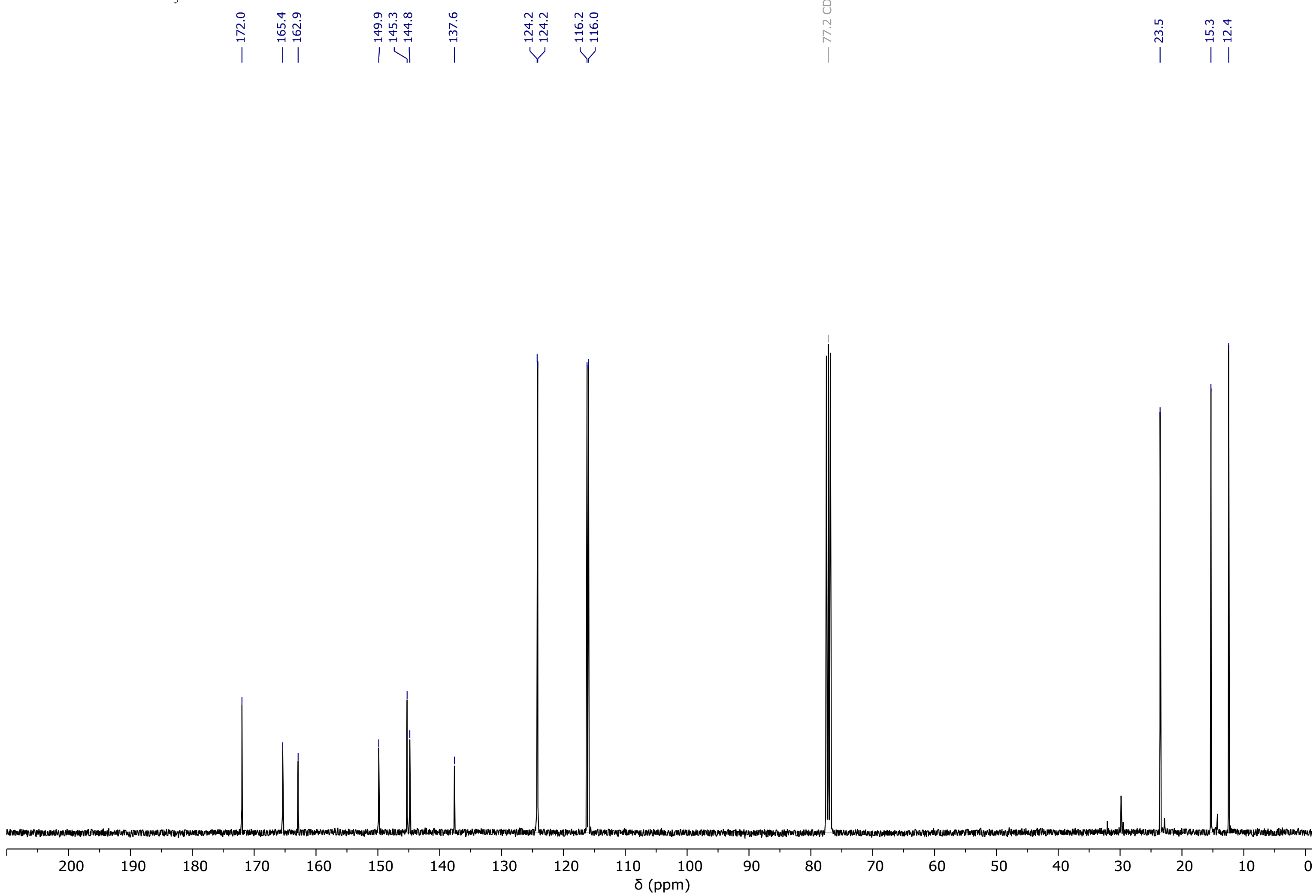

Figure S212: ^{13}C NMR spectrum of (*E*)-1-(4-((4-fluorophenyl)diazenyl)-3,5-dimethyl-1*H*-pyrazol-1-yl)ethan-1-one in $CDCl_3$.

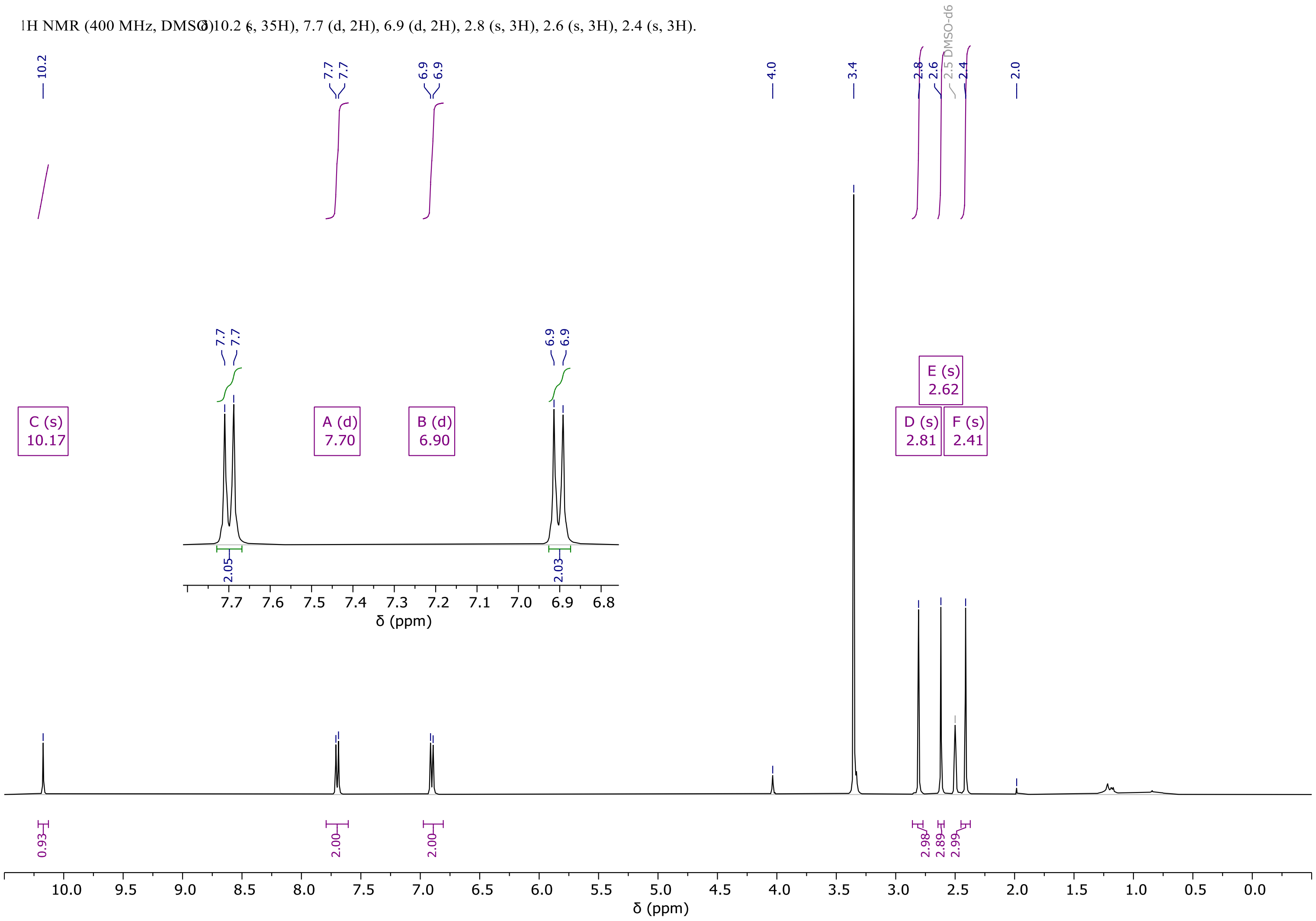

Figure S213: ^{1}H NMR spectrum of (*E*)-1-(4-((4-hydroxyphenyl)diazenyl)-3,5-dimethyl-1*H*-pyrazol-1-yl)ethan-1-one in DMSO-d_6.

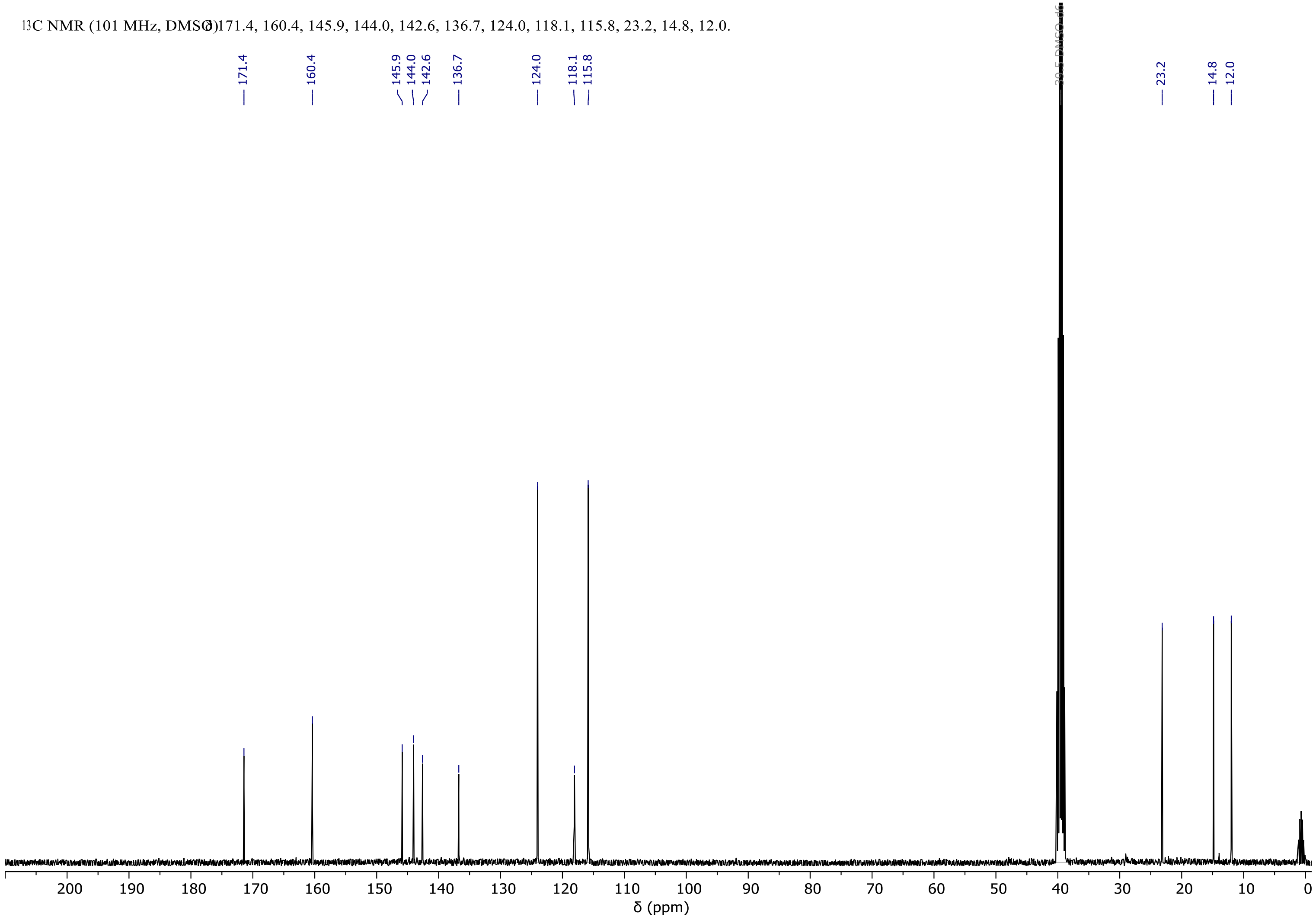

Figure S214: ^{13}C NMR spectrum of (*E*)-1-(4-((4-hydroxyphenyl)diazenyl)-3,5-dimethyl-1*H*-pyrazol-1-yl)ethan-1-one in DMSO-d_6.

5. References

(1) Weston, C. E.; Richardson, R. D.; Haycock, P. R.; White, A. J. P.; Fuchter, M. J. Arylazopyrazoles: Azoheteroarene Photoswitches Offering Quantitative Isomerization and Long Thermal Half-Lives. *J. Am. Chem. Soc.* **2014**, *136* (34), 11878–11881. https://doi.org/10.1021/ja505444d.

(2) Stricker, L.; Böckmann, M.; Kirse, T. M.; Doltsinis, N. L.; Ravoo, B. J. Arylazopyrazole Photoswitches in Aqueous Solution: Substituent Effects, Photophysical Properties, and Host–Guest Chemistry. *Chem. – Eur. J.* **2018**, *24* (34), 8639–8647. https://doi.org/10.1002/chem.201800587.

(3) Kumar, P.; Srivastava, A.; Sah, C.; Devi, S.; Venkataramani, S. Arylazo-3,5-dimethylisoxazoles: Azoheteroarene Photoswitches Exhibiting High *Z* -Isomer Stability, Solid-State Photochromism, and Reversible Light-Induced Phase Transition. *Chem. – Eur. J.* **2019**, *25* (51), 11924–11932. https://doi.org/10.1002/chem.201902150.

(4) Patel, H. V.; Vyas, K. A.; Pandey, S. P.; Fernandes, P. S. Reaction of 2, 3, 4-Pentantrione-3-Arylhydrazones with *N, N* -Dimethylhydrazine: Formation of Substituted 1 *H* -Pyrazoles *via* Demethylation. *Synth. Commun.* **1992**, *22* (21), 3081–3087. https://doi.org/10.1080/00397919209409257.

(5) Rustler, K.; Nitschke, P.; Zahnbrecher, S.; Zach, J.; Crespi, S.; König, B. Photochromic Evaluation of 3(5) -Arylazo-1 *H* -Pyrazoles. *J. Org. Chem.* **2020**, *85* (6), 4079–4088. https://doi.org/10.1021/acs.joc.9b03097.

(6) Devi, S.; Saraswat, M.; Grewal, S.; Venkataramani, S. Evaluation of Substituent Effect in *Z* -Isomer Stability of Arylazo-1 *H* -3,5-Dimethylpyrazoles: Interplay of Steric, Electronic Effects and Hydrogen Bonding. *J. Org. Chem.* **2018**, *83* (8), 4307–4322. https://doi.org/10.1021/acs.joc.7b02604.

(7) Stricker, L.; Fritz, E.-C.; Peterlechner, M.; Doltsinis, N. L.; Ravoo, B. J. Arylazopyrazoles as Light-Responsive Molecular Switches in Cyclodextrin-Based Supramolecular Systems. *J. Am. Chem. Soc.* **2016**, *138* (13), 4547–4554. https://doi.org/10.1021/jacs.6b00484.

(8) Kauth, A.-M.; Niebuhr, R.; Ravoo, B. J. Arylazopyrazoles for Conjugation by CuAAC Click Chemistry. *J. Org. Chem.* **2024**, *89* (9), 6371–6376. https://doi.org/10.1021/acs.joc.4c00354.

(9) Grebenovsky, N.; Goldau, T.; Bolte, M.; Heckel, A. Light Regulation of DNA Minicircle Dimerization by Utilizing Azobenzene *C* -Nucleosides. *Chem. – Eur. J.* **2018**, *24* (14), 3425–3428. https://doi.org/10.1002/chem.201706003.

(10) Weston, C. E.; Krämer, A.; Colin, F.; Yildiz, Ö.; Baud, M. G. J.; Meyer-Almes, F.-J.; Fuchter, M. J. Toward Photopharmacological Antimicrobial Chemotherapy Using Photoswitchable Amidohydrolase Inhibitors. *ACS Infect. Dis.* **2017**, *3* (2), 152–161. https://doi.org/10.1021/acsinfecdis.6b00148.

(11) Bland, R. D.; Clarke, T. L.; Harden, L. B. Rapid Infusion of Sodium Bicarbonate and Albumin into High-Risk Premature Infants Soon after Birth: A Controlled, Prospective Trial. *Am. J. Obstet. Gynecol.* **1976**, *124* (3), 263–267. https://doi.org/10.1016/0002-9378(76)90154-x.

(12) Stranius, K.; Börjesson, K. Determining the Photoisomerization Quantum Yield of Photoswitchable Molecules in Solution and in the Solid State. *Sci. Rep.* **2017**, *7* (1), 41145. https://doi.org/10.1038/srep41145.
